# Bromopyrrole Alkaloids with the Inhibitory Effects against the Biofilm Formation of Gram Negative Bacteria

**DOI:** 10.3390/md16010009

**Published:** 2018-01-02

**Authors:** Jingyuan Sun, Jiru Wu, Bang An, Nicole J. de Voogd, Wei Cheng, Wenhan Lin

**Affiliations:** 1 State Key Laboratory of Natural and Biomimetic Drugs, Peking University, Beijing 100191, China; jysun@bjmu.edu.cn (J.S.); 13478333545@163.com (J.W.); anbangsps@pku.edu.cn (B.A.); 2 The Netherlands Centre for Biodiversity Naturalis, P.O. Box 9517, 2300 RA Leiden, The Netherlands; nicole.devoogd@naturalis.nl

**Keywords:** marine sponge, *Stylissa massa*, antimicrobial activity, antibiofilm, structure elucidation

## Abstract

Anti-biofilm assay guided fractionation of the marine sponge *Stylissa massa* revealed the butanol soluble fraction that was possessing the inhibitory activity toward the biofilm formation of bacterium *E. coli*. Chromatographic separation of the bioactive fraction resulted in the isolation of 32 bromopyrrole alkaloids, including six new alkaloids, named stylisines A–F (**1**–**6**). The structures of new alkaloids were established by comprehensive analyses of the two-dimensional (2D) NMR (COSY, HMQC, and HMBC) and the high resolution electrospray ionization mass spectroscopy (HRESIMS) data, while the absolute configurations were determined by the X-ray diffraction and the electronic circular dichroism (ECD) data. Bioassay results indicated that phakellin-based alkaloids, including dibromoisophakellin and dibromophakellin, significantly reduced the biofilm formation of the bacterium *E. coli*. Present work provided a group of new natural scaffolds for the inhibitory effects against the biofilm formation of *E. coli*.

## 1. Introduction

**Figure 1:**
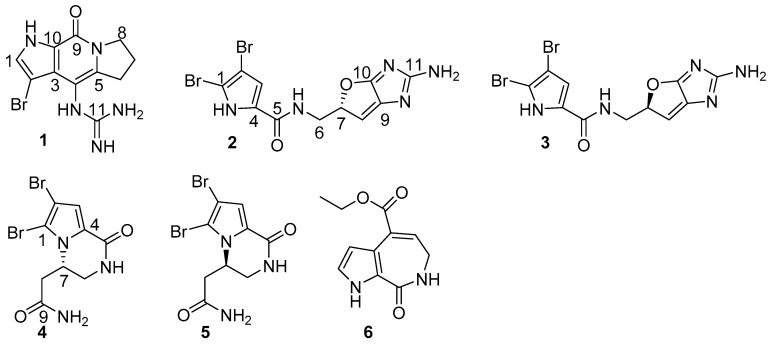
Structures of stylisines A–F (**1**–**6**).

Planktonic bacteria attached onto solid surfaces, creating a complex community of bacteria to form a biofilm, which protects bacteria against antibiotics and causes many recalcitrant infections, as well as resistance to antibiotics [1,2,3,4]. Biofilms account for >80% of human bacterial infections, and a biofilm matrix resists antibiotics at concentrations over 1000 times higher than conventionally used doses [5,6,7]. In nature, most bacteria are likely to form surface-attached biofilm communities as a survival strategy. Pathogenic biofilms pose a challenge because they enhance resistance not only to conventional antibiotics, but also to host defenses and external stresses. Thus, they are difficult to control in medical and industrial settings. So far, no specific biofilm inhibitor is commercially available, while bacterial biofilms remain a frontier of microbiology as they exhibit important tolerance and resistance in many aspects. Currently, an explosive amount of biofilm research is being conducted to discover novel compounds that are capable of inhibiting biofilms, without allowing bacteria to develop drug resistance. Marine benthic invertebrates have been proved a rich source of bioactive compounds with multiple ecological functions. Among the sessile organisms, sponges are the marine sessile filter feeders that are exposed to large amounts of bacteria and also viruses in the surrounding seawater. Because bacteria play a key role in microfilm formation [8], it becomes vital for sponges to regulate bacterial epibiosis on their surfaces. One epibacterial defense strategy for sponge-symbiont community is to produce bioactive secondary metabolites in order to outcompete other bacteria in the water column from causing infection or biofouling [9,10,11,12,13,14]. Among the sponge-derived active metabolites, pyrrole-imidazole alkaloids are a class of structurally unique natural products with a range of ecological and significant pharmacological bioactivities [15,16,17]. For instance, oroidin and stevensine were demonstrated to possess antifeedant properties, while oroidin and its analogues have been extensively studied as biofilm inhibitors [18,19,20,21]. In our research program for the discovery of bioactive natural products that are derived from marine sponges, HPLC-DAD-MS data and bioassay guided examination of the sponge extracts revealed that the *n*-BuOH extracts of the sponge *Stylissa massa* contain a profile of brominated alkaloids, and possess an inhibitory effect against the biofilm formation of *E. coli*. Chromatographic separation of the alkaloid-containing extracts resulted in the isolation of 32 bromopyrrole alkaloids, including six new compounds, namely stylisines A–F (**1**–**6**) (Figure 1). The antibacterial and anti-biofilm effects of the isolated alkaloids were evaluated.

## 2. Results

The sponge tissue was extracted with 95% alcohol, and the crude extract was partitioned between 40% MeOH-H_2_O and CH_2_Cl_2_ to remove lipids. The water soluble extract was then partitioned between H_2_O and *n*-BuOH. The ^1^H NMR spectrum in association with the LC-ESIMS data of *n*-BuOH extract characterized a profile of brominated alkaloids. Chromatographic separation, including the semi-preparative HPLC purification, conduced the isolation of 32 bromopyrrole derivatives, including six new alkaloids (Figure 1).

**Figure 2:**
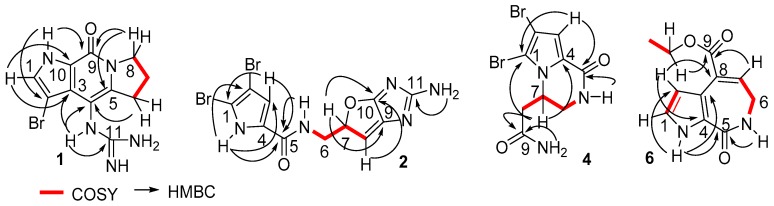
Key COSY and HMBC correlations of **1**, **2**, **4** and **6**.

Stylisine A (**1**) was obtained as a light yellow amorphous solid. Its molecular formula was determined as C_11_H_13_N_5_OBr on the basis of the high resolution electrospray ionization mass spectroscopy (HRESIMS) and NMR data. The pseudomolecular ion peaks at *m*/*z* 310 and 312, with a ratio of 1:1 in the ESIMS spectrum also supported a bromine atom in the molecule. The ^13^C NMR and distortionless enhancement by polarization transfer (DEPT) spectra exhibited 11 carbon resonances, which were classified into three methylenes, one methine, and seven quaternary carbons. The COSY in association with the HMBC correlations established a tetrahydropyrroloindolizinone nucleus, as evident from the COSY relationships for an alkyl spin system from H_2_-6 (*δ*_H_ 2.87, 2.99) to H_2_-8 (*δ*_H_ 3.98, 4.10), in addition to the HMBC correlations from the pyrrole proton H-1 (*δ*_H_ 7.46, brs) to C-3 (*δ*_C_ 123.1) and C-10 (*δ*_C_ 126.4), NH (*δ*_H_ 12.54, brs, pyrrole) to C-2 (*δ*_C_ 86.8), C-3, and C-9 (*δ_C_* 152.6), and between H_2_-8/C-9 and H_2_-6/C-4 (*δ*_C_ 102.8). The remaining moiety involved a quaternary carbon at *δ*_C_ 157.5 (C-11) and three nitrogen atoms, which was characteristic of a guanidine unit that was substituted at C-4 due to the HMBC correlations from a NH (9.16, s) to C-3, C-4, C-5 (*δ*_C_ 142.2), and C-11 (Figure 2). In addition, the significantly shielded carbon C-2 (*δ*_C_ 86.8) was attributed to a bromine atom substitution.

**Figure 3:**
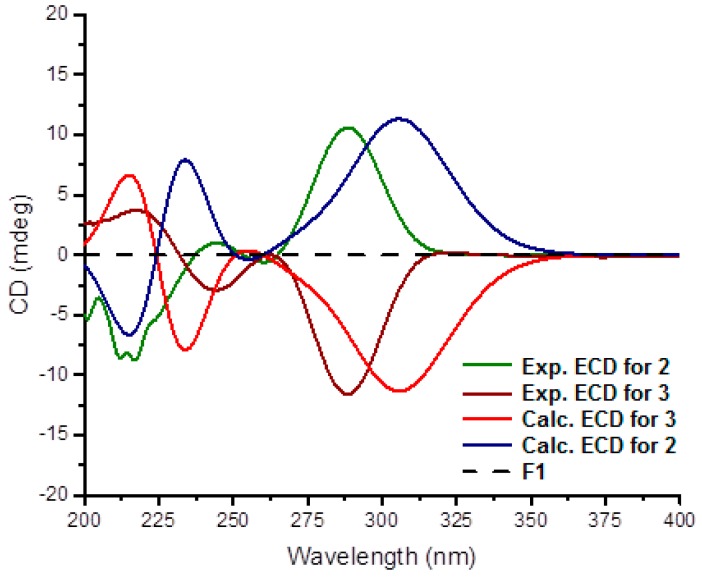
Experimental and calculated electronic circular dichroism (ECD) data for **2** and **3** in MeOH.

Stylisine B (**2**) was obtained as a yellow amorphous solid. Its ESIMS data presented the ion peaks at *m*/*z* 401.9, 403.9, and 405.9 [M + H]^+^ with the ratio of 1:2:1, indicating two bromine atoms in the molecule, while the HRESIMS and NMR data provided a molecular formula of C_11_H_10_N_5_O_2_Br_2_. Analyses of the two-dimensional (2D) NMR data revealed that compound **2** consists of two units of substructures. The first unit was determined to be a pyrrole-2-carboxamide, based on the HMBC correlations from H-3 (*δ*_H_ 6.95, s) to C-1 (*δ*_C_ 105.2) and a carbonyl carbon C-5 (*δ*_C_ 159.5), and from NH (*δ*_H_ 12.68, s, pyrrole) to C-2 (*δ*_C_ 98.4), C-3 (*δ*_C_ 113.5), and C-5. The second unit was assigned to a furoimidazole nucleus, according to additional HMBC correlations from H-7 (*δ*_H_ 4.37, m) to C-9 (*δ*_C_ 128.6) and C-10 (*δ*_C_ 153.5), and H-8 (*δ*_H_ 5.76, s) to C-7 (*δ*_C_ 74.0) and C-10. An amine group to be positioned at C-11 (*δ*_C_ 159.5) was ascribed by the HMBC correlation between NH_2_ (*δ*_H_ 9.23, s) and C-11. The connection of two units across a methylene group to form an amide was clarified by the COSY relationships from H_2_-6 (*δ*_H_ 3.43, m) to an amide NH (*δ*_H_ 8.15, t, *J* = 6.0 Hz) and H-7, along with the HMBC correlations from C-5 to the amide proton and H_2_-6. The significantly shielded quaternary carbons C-1 and C-2 were indicative of the substitution of bromine atoms at C-1 and C-2, respectively [22]. The absolute configuration at C-7 was determined on the basis of the comparison of the experimental and calculated electronic circular dichroism (ECD) data. The experimental ECD spectrum of **2** exhibited the positive Cotton effects (CE) at 240 and 280 nm, and negative CE at 230 nm, which were in accordance with the calculated ECD data for the model compound of **2** with 7*S* configuration, using the time-dependent density functional theory (TDDFT) method (Figure 3) [23,24]. Thus, C-7 of **2** was in agreement with *S* configuration.

The planer structure of stylisine C (**3**) was the same as that of **2**, as determined by the duplicated NMR (Table 1) and MS data. However, the specific rotation of **2** ([α]${}_{D}^{25}$ +30.8, MeOH) showed the opposite phase to that of **3** ([α]${}_{D}^{25}$ − 31.2, MeOH), suggesting **3** to be a stereoisomer of **2**. This assumption was confirmed by the opposite Cotton effects of **3** in comparison with those of **2** in the ECD spectra (Figure 3). The comparable Cotton effects of the experimental ECD of **3** and the calculated ECD for the 7*R* isomer of **2**, indicated the absolute configuration of **3** to be 7*R*. 

**Figure 4:**
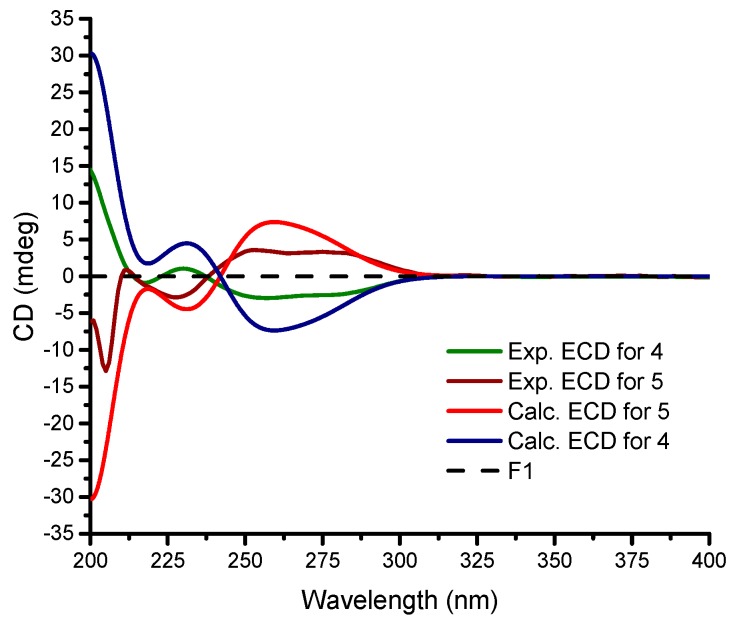
Experimental and calculated ECD data for **4** and **5** in MeOH.

Stylisine D (**4**) was obtained as yellow amorphous solid. Its HRESIMS and NMR data established a molecular formula of C_9_H_10_N_3_O_2_Br_2_, containing two bromine atoms. A comparison of the NMR data (Table 1) indicated that **4** shared the partial structure (1,2-dibromopyrrole-4-carboxamide) of **2**. The COSY relationships established an alkyl spin system from H_2_-6 (*δ*_H_ 3.34, 3.77) to H_2_-8 (*δ*_H_ 2.26, 2.63), while H_2_-6 extended the COSY correlation to an NH proton (*δ*_H_ 7.87, t). The HMBC correlations from a carbonyl carbon C-9 (*δ*_C_ 170.3) to H_2_-8, H-7 (*δ*_H_ 4.64), and NH_2_ (*δ*_H_ 7.13, 7.54) allowed for the linkage of a terminal amide to C-8 (*δ*_C_ 36.2). The connection of C-7 (*δ*_C_ 50.6) to the nitrogen atom of the pyrrole ring was deduced by the HMBC correlations from H-7 to C-1 (*δ*_C_ 105.7) and C-4 (*δ*_C_ 125.8). In addition, the HMBC correlations from NH (*δ*_H_ 7.87) to C-4 and the carbonyl carbon C-5 (*δ*_C_ 157.6) and between H-3 (*δ*_H_ 6.84, s) and C-5, resulted in the formation of a *δ*-lactam. The experimental ECD data showed positive CE at 228 nm and negative CE at 260 nm, which were in accordance with the calculated ECD data for a model compound of **4** with 7*S* configuration (Figure 4).

The identical NMR data of stylisine E (**5**) and **4** (Table 1), in association with the NMR and the HRESIMS data, assigned the same planar structure of both **5** and **4**. The distinction was attributed to the absolute configuration at C-7, which was determined to be 7*R*, according to the Cotton effects of **5** showing opposite phases to those of **4,** and to be similar with those calculated for 7*R* isomer of **4** (Figure 4).

Stylisine F (**6**) was obtained as a yellow amorphous solid. Its molecular formula was established as C_11_H_12_N_2_O_3_ on the basis of the HRESIMS and NMR data, requiring seven degrees of unsaturation. The ^13^C NMR and DEPT spectra exhibited 11 carbon resonances, of which, eight sp^2^ carbons and three sp^3^ carbons were defined. The COSY correlations from H-1 (*δ*_H_ 6.98) to H-2 (*δ*_H_ 6.52) and NH (*δ*_H_ 11.85), in association with the HMBC correlations from H-1 and H-2 to the quaternary carbons C-3 (*δ*_C_ 120.0) and C-4 (*δ*_C_ 126.2), disclosed a 3,4-disubstituted pyrrole ring. The HMBC correlations from NH to the carbons in the pyrrole ring and a carbonyl carbon C-5 (*δ*_C_ 163.3) ascertained a carbonyl group at C-4. A spin system coupling H_2_-6 (*δ*_H_ 3.48, dd) with NH (*δ*_H_ 7.73, t) and H-7 (*δ*_H_ 6.84, t) in the COSY spectrum, in addition to the HMBC correlations from H_2_-6 to C-5, and the olefinic carbons C-7 (*δ*_C_ 132.5) and C-8 (*δ*_C_ 131.6) and between H-7 and C-3, established a pyrroloazepine nucleus. Additional HMBC correlations from H-7 and the ethoxy methylene protons (*δ*_H_ 4.21, t) to the carbonyl carbon C-9 (*δ*_C_ 166.0) confirmed an ethoxycarbonyl group at C-8. Compound **6** was suggested to be an artifact derived during the process of the EtOH extraction, while 8-carboxylic analogue was considered to be the natural origin of **6**.

**Figure 5:**
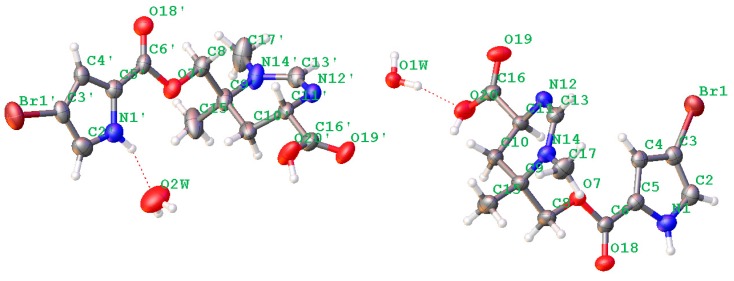
Plot of the X-ray diffraction of *N*-methylmanzacidin C.

In addition, 26 known alkaloids including pyrrole-2-carbamide type derivatives (1,2-dibromopyrrole-4-carbamide (**7**) [25], 2-bromopyrrole-4-carbamide (**8**), 1,2-dibromopyrrole-4-ethylate (**9**) [26], oroidin (**10**) [27], hymenidin (**11**) [28], dispacamide 1 (**12**) [29], keramadine (**13**) [30], laughine (**14**) [31], taurodispacamide A (**15**) [32]); aldizine type analogues (aldizine (**16**), 1-bromoaldizine (**17**) [33], 10*Z*-hymenialdisine (**18**), 10*E*-hymenialdisine (**19**) [34], hymenin (**20**) [35], stevensine (**21**) [36], (10*E*)-3-bromohymenialdisine (**22**), (10*Z*)-3-bromohymenialdisine (**23**) [37] and (10*Z*)-debromohymenialdisine (**24**)); phakellin type analogues (dibromoisophakellin (**25**), dibromophakellin (**26**) [38], monobromophakellin (**27**), monobromoisophakellin (**28**)); mamzacidin type analogues (manzacidins B-C (**29**, **30**) [39], *N*-methylmanzacidin C (**31**) [40]); in addition to longamide B (**32**) (Supporting Information, Figure S41) [41], were identified by the comprehensive analyses and the comparison of one-dimensional (1D) and 2D NMR, and HRESIMS data, in association with the specific rotations. The absolute configurations of *N*-methylmanzacidin C were confirmed by the X-ray diffraction of a single crystal using the Flack parameter for the first time (Figure 5).

**Scheme 1:**
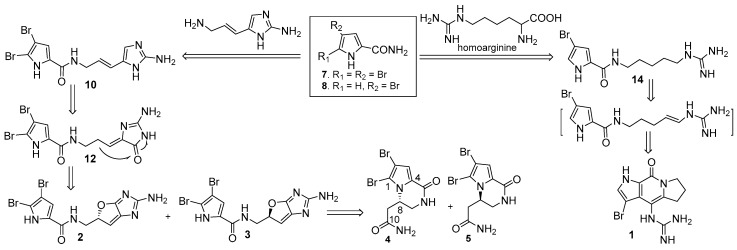
Biogenetic postulation to derive **1**–**5**.

Biogenetically, pyrrole-2-carbamide and brominated pyrrole-2-carbamides, such as **7** and **8,** were postulated to be the precursors to derive the isolated alkaloids [42,43,44]. Condensation of **8** with homoarginine yielded laughine (**14**), while the latter compound underwent dehydrogenation and cyclization to generate **1**. Condensation of **7** with 3-amino-l-(2-aminoimidazolyl)-prop-l-ene generated oroidin (**10**), which followed oxygenation and olefinic migration to afford dispacamide 1 (**12**). Heterocyclization of **12** through the formation of an ether bridge to derive the stereoisomers **2** and **3**. Compounds **4** and **5** were considered to be generated from **2** and **3** by ring rearrangement and oxygenation (Scheme 1). 

Hymenidin, taurodiscapamide A, and oroidin have been reported to display significant growth inhibition against the Gram positive bacteria *Staphylococcus aureus* and *Bacillus subtilis*, and the Gram negative bacteria *Escherichia coli* and *Pseudomonas aeruginosa*, as well as the fungus *C. albicans* [22]. Oroidin was also reported to inhibit the growth of *Rhodospirillum salexigens*, a marine bacterium that is known to form biofilms [19]. 1,2-Dibromopyrrole-4-carbamide promoted larval metamorphosis of the ascidian *Ciona savignyi* [25]. In addition, oroidin-based derivatives incorporating a 2-aminoimidazole motif served as molecules with a unique scaffold to inhibit and disperse bacterial biofilms [45,46].

The literature data stimulated us to examine the isolated alkaloids individually for growth inhibition against pathogenic bacteria, including the Gram positive bacteria *S. aureus* ATCC 25923, *S. haemolyticus* ATCC 29970, and *B. subtilis* ATCC 11562, and the Gram negative bacteria *E. coli* ATCC 25922, *Xanthomanes vesicatoria* ATCC 11633, *P. lachrymans* ATCC 11921, *Agrobaterium tumefaciens* No. 8 ATCC 11158, *Ralstonia solanacearum* ATCC 11696. Unexpectedly, most compounds showed weak or no activity against the panel of bacteria with MIC ≥ 128 μg/mL (Supporting Information, Table S1), with the exception of oroidin that showed MIC values ranging from 32–128 μg/mL. 

**Figure 6:**
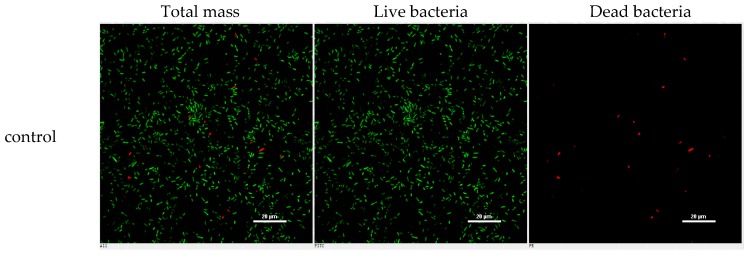

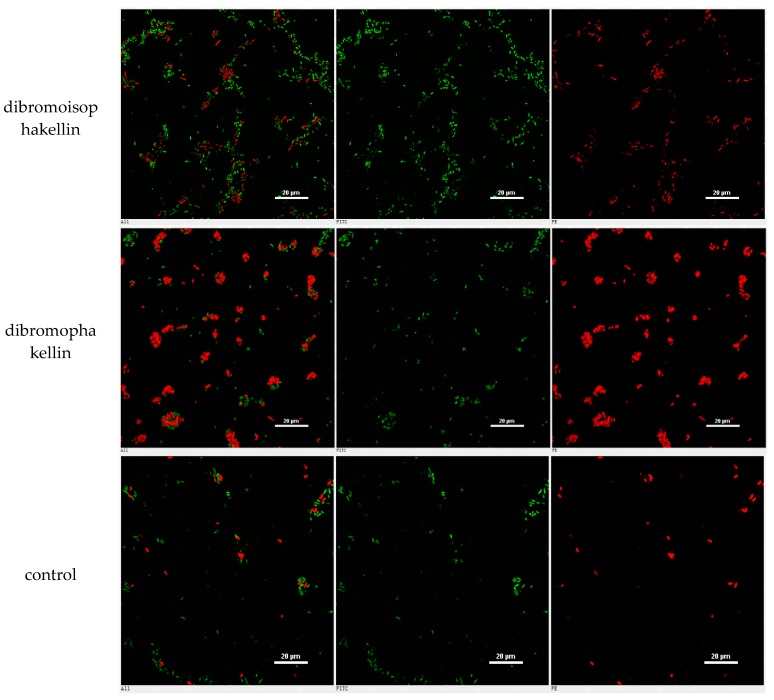
Microscopy images of dibromoisophakellin and dibromophakellin showing inhibition toward *Escherichia coli* biofilms by LIVE/DEAD-stained after 24 h.

However, the initial bioassay indicated that the *n*-BuOH extract (an extract of total alkaloids) exerted inhibitory effects against the biofilm formation of *E. coli.* The bioassay guided fractionation of *n*-BuOH extract revealed three fractions (F2 to F4) to be active (Table 2), while the most active fraction was attributed to F2, in which dibromoisophakellin and dibromophakellin were isolated as the main components. Both of the compounds exerted significant inhibition against the biofilm formation of *E. coli* with IC_50_ value of 50.9 and 31.3 μg/mL, respectively. In order to understand the bioactivities of the relative analogues, we extended the anti-biofilm assay of remaining alkaloids. However, those alkaloid derivatives showed weak or no inhibition toward the biofilm formation (Table 3). Preliminary analyses of the structure-activity relationship indicated that phakellin-type scaffold with an aminoimidazole ring is essential to induce the inhibitory activity, while analogues with dibromo substitution showed more active than those with monobromo substitution. In parallel, confocal laser scanning microscopy (CLSM) was used to detect the ability of dibromophakellin and dibromoisophakellin to interfere with the biofilm formation of *E. coli*. In this experiment, the biofilm-embedded cells were LIVE/DEAD stained. The viable bacteria were stained with intact membranes fluorescing green (SYTO9) to distinguish the dead bacteria that were stained with damaged membrane fluorescing red (propidium iodine) [47,48]. As shown in Figure 6, the number of the viable bacteria of *E. coli* in the biofilm and the total biofilm thickness were greatly reduced after the treatment with dibromoisophakellin and dibromophakellin individually at a dose of 100 μg/mL in comparison with the compact and condensed biofilm control. These images provided additional evidence to support the inhibitory effects of both compounds against the biofilm formation. In parallel experiment, the untreated biofilms of *E. coli* in the vehicle group (DMSO) showed an inhomogeneous distribution with visible cell clusters and clearly rod shaped, whereas the *E. coli* cells treated by dibromoisophakellin and dibromophakellin changed their cell morphology to a more spherical shape, and tended to be aggregated.

A–C = DMSO control, D–F = treated with dibromoisophakellin (100 μg/mL), G–I = treated with dibromophakellin (100 μg/mL), and J–L = treated with chloramphenicol (20 μg/mL). Viable bacteria are visible in green and dead bacteria in red. Left panels (A, D, G, J) for integrated red and green flourescent channels, middle panels (B, E, H, K) for green (live bacteria) and right panels (C, F, I, L) for red (dead bacteria).

## 3. Discussion

To the best of our knowledge, this study uncovered the largest numbers of brominated alkaloids with diverse scaffolds that were derived from marine sponge *S. massa*, implying more complexity of ecological environments, such as high biodiversity of predators, competitors, and overgrowths, as well as natural sponge-microbial associations in tropical sea water [49]. In addition to the postulation of the biogenetic relationships for new compounds in Scheme 1, complex hetercycles of pyrrole-imidazole alkaloids, such as aldizine-type and phakellin-type alkaloids, were supposed to be generated biogenetically from oroidin as a key intermediate through different manners of rearrangement and cyclization [44,50]. Although sponges have large symbiotic bacterial populations, oroidin and its analogues have been proved to be derived from sponge origin producing in its spherulous cells [51]. The structure variation is suggested to be an active enzymatic process involving the induction by symbiotic microorganisms. The prominent alkaloids, including oroidin (**10**), hymenidin (**11**), 10*Z* or 10*E* hymenialdisine (**18** or **19**), and debromohymenialdisine (**24**) from the sponge distributed at various biogeographic locations across Indo-Pacific reefs, suggesting that these typical alkaloids could be employed as chemical marks for taxonomy. 

In the naturally occurring pyrrole-imidazole alkaloids, as derived from marine sponges, very few have been shown to modulate biofilm formation without killing the bacteria or disrupting their growth [8]. Oroidin and bromoageliferin were able to interfere with the bacterial attachment of *Rhodospirillum salexigens* and *Vibrio vulnificus* [52,53], where *R. salexigens* is a marine bacterium that is known to form biofilms. Oroidin exhibited modest anti-biofilm activity against two strains (PAO1 and PA14) of *Pseudomonas aeruginosa,* with IC_50_ values of 190 and 166 μM, whereas its analogue, dihydrosventrin, showed stronger inhibition against PAO1 and PA14 with IC_50_ values of 52 and 111 μM, respectively [54]. In our work, oroidin was inactive toward the anti-biofilm formation of *E. coli*, but dispacamide 1 (**12**) showed weak activity, with 10.4% inhibition rate at 100 μg/mL without microbicidal effect. These findings suggested that different pyrrole-imidazole alkaloids could play separate roles in ecological defense. Phakellin-based alkaloids dibromoisophakellin and dibromophakellin exerted significant reduction against the biofilm formation of *E. coli* with weak inhibition against the growth inhibition of planktonic bacteria, indicating the inhibitory effects via non-microbicidal mechanism. The molecular mechanism of the active compounds to eliminate biofilm formation remains to be assessed.

## 4. Materials and Methods 

### 4.1. General Experimental Procedures 

Optical rotations were measured on a Rudolph IV Autopol automatic polarimeter. IR spectra were determined on a Thermo Nicolet Nexus 470 FT-IR spectrometer. 1D and 2D NMR spectra were recorded on a Bruker Avance 400 NMR spectrometer (400 MHz for ^1^H and 100 MHz for ^13^C, respectively). Chemical shifts were referenced to the solvent peaks at *δ*_H_ 2.50 and *δ*_C_ 39.8 for dimethyl sulfoxide (DMSO-*d*_6_), respectively, and coupling constants were in Hz. ESIMS and HRESIMS spectra were obtained from a Thermo Scientific LTQ Orbitrap XL instrument. Column chromatography was carried out with silica gel (160–200 mesh, 200–300 mesh), and HF_254_ silica gel for TLC was obtained from Qingdao Marine Chemistry Co. Ltd. (Qingdao, China), ODS gel (50 μm), and Sephadex LH-20 (18–110 μm) were obtained from YMC (Kyoto, Japan) and Amersham Pharmacia Biotech AB, Uppsala, Sweden. HPLC chromatography was performed on an Alltech instrument (426-HPLC pump), equipped with an Alltech UVIS-200 detector and semipreparative reversed-phase columns (Grace Prevail C_18_, 5 μm, 250 mm × 10 mm). Analytical HPLC chromatography was performed on Prominence LC-20AD (Shimadzu, Kyoto, Japan) equipped with pump LC-20AD, detector Prominence SPD-M20A PDA and Shimadzu VP-ODS column (150 mm × 4.6 mm), Thermo BDS Hypersil C_18_ column (150 mm × 4.6 mm, 5 μm).

### 4.2. Animal Material 

Sponge *Stylissa massa* was collected from the inner coral reef at a depth of around 8 m in Hainan Island of China, in May 2013, and the samples were frozen immediately after collection. The specimen was identified by Dr. Nicole J. de Voogd (Naturalis Biodiversity Center (Leiden, The Netherlands)). The voucher specimens (XSA-24) are deposited at the State Key Laboratory of Natural and Biomimetic Drugs, Peking University, China.

### 4.3. Isolation and Purification 

Sponge tissue (3.5 kg, wet weight) was extracted with 95% EtOH for three times, and the crude extract was partitioned between MeOH and *n*-hexane to remove lipids. The MeOH layer was further partitioned between H_2_O and *n*-BuOH to afford *n*-BuOH soluble extract (11.4 g). The DAD-HPLC-ESIMS and the NMR data revealed that *n*-BuOH fraction contains a profile of brominated alkaloids. The silica gel chromatography of *n*-BuOH-fraction (4.9 g) was eluted with CH_2_Cl_2_-MeOH (a gradient from 6:1 to 2:1) to afford five fractions (F1 to F5). F1 (336 mg) was chromatographed on a silica gel column eluting with petroleum ether (PE)-acetone (from 3:1 to 1:1, gradient), to yield **7** (6.0 mg), **17** (11.3 mg), **13** (8.6 mg), and **16** (29.3 mg). Part of F2 (140 mg) was subjected to a silica gel column using CH_2_Cl_2_-MeOH (13:1) as a mobile phase followed by HPLC purification using a mobile phase of CH_3_CN-H_2_O (28:72, 1‰ CF_3_COOH) to afford **25** (11.7 mg) and **26** (117.5 mg). F3 (795 mg) was separated on ODS column eluting with MeOH-H_2_O (1:5) to afford six subfractions (SF31–SF36). SF31 (146 mg) was purified by a semi-preparative HPLC eluting with CH_3_CN-H_2_O (14:86, 3‰ CF_3_COOH) to afford **1** (21.1 mg) and **27** (30.8 mg). Following the same separation protocol by the semi-preparative HPLC eluting with MeOH-H_2_O (3:1), **28** (1.2 mg) was purified from SF32 (82.8 mg). SF33 (248 mg) was separated on Sephadex LH-20 column eluting with MeOH to afford **11** (26 mg). SF34 (28 mg) was purified by the semi-preparative HPLC eluting with CH_3_CN-H_2_O (1:4, 1‰ CF_3_COOH) to afford **18** (3.3 mg), **12** (7.2 mg), and **10** (99.2 mg). F4 (515 mg) was separated on ODS column and eluted with a gradient of MeOH-H_2_O (25–50%) to afford five subfractions (SF41–SF45). Following the semi-preparative HPLC separation, **19** (6.7 mg) was obtained from SF42 (177 mg) (CH_3_CN-H_2_O 12:88, 1‰ CF_3_COOH), **15** (17.5 mg), **20** (13.5 mg), **8** (3.1 mg), and **21** (7.6 mg) were obtained from SF43 (120 mg). SF44 (337 mg) was separated by semi-preparative HPLC eluting with 32% CH_3_CN-H_2_O (1‰ CF_3_COOH) to yield **22** (2.9 mg), **23** (17.5 mg), and **32** (3.6 mg). F5 (2.9 g) was subsequently subjected to MPLC, and then purified by semi-preparative HPLC eluting with 36% MeOH-H_2_O to afford **24** (9.9 mg), **31** (2.0 mg), **30** (39.7 mg), **29** (11.9 mg), **9** (39.1 mg), **15** (9.6 mg), **2**/**3** mixture (5.2 mg), **4**/**5** (4.1 mg) mixture, and **6** (4.7 mg). The separation of **2**/**3** (a ratio of 1:1) and **4**/**5** (a ratio of 5:4) was achieved by the chiral HPLC column (Daciel CHIRALPAK IC column) eluting with *n*-hexane-isopropanol (78:22) to obtain **2** (2.4 mg), **3** (2.5 mg), **4** (2.0 mg), and **5** (1.8 mg). 

*Stylisine A* (**1**): light yellow solid, [α]${}_{D}^{25}$ − 9.4 (*c* 0.14, MeOH), IR (KBr film) *ν*_max_ 3322, 3149, 3079, 2925, 1679, 1657, 1585, 1205, 1136 cm^−1^, ^1^H and ^13^C NMR (DMSO-*d*_6_), see Table 1, ESIMS *m*/*z* 310.03 [M + H]^+^ and HRTOFMS *m*/*z* 310.0311 [M + H]^+^ (calcd. for C_11_H_13_N_5_OBr, 310.0303).

*Stylisine B* (**2**): light yellow amorphous, [α]${}_{D}^{25}$ + 30.8 (*c* 0.05, MeOH), IR (KBr film) *ν*_max_ 3424, 1677, 1205, 1138 cm^−1^; ^1^H and ^13^C NMR (DMSO-*d*_6_), see Table 1, ESIMS *m*/*z* 401.92 [M + H]^+^ and HRTOFMS *m*/*z* 401.9199 [M + H]^+^ (calcd. for C_11_H_10_N_5_O_2_Br_2_, 401.9201).

*Stylisine C* (**3**): light yellow amorphous, [α]${}_{D}^{25}$ − 31.2 (*c* 0.05, MeOH), IR (KBr film) *ν*_max_3424, 2853, 1677, 1439, 1206, 1138 cm^−1^, ^1^H and ^13^C NMR (DMSO-*d*_6_), see Table 1, ESIMS *m*/*z* 401.92 [M + H]^+^ and HRTOFMS *m*/*z* 401.9199 [M + H]^+^ (calcd. for C_11_H_10_N_5_O_2_Br_2_, 401.9201).

*Stylisine D* (**4**): light yellow amorphous, [α]${}_{D}^{25}$ − 4.0 (*c* 0.05, MeOH), IR (KBr film) *ν*_max_ 3452, 1735, 1719, 1674, 1653, 1381, 1367, 1205, 1139 cm^−1^; ^1^H and ^13^C NMR (DMSO-*d*_6_), see Table 1, ESIMS *m*/*z* 349.91 [M + H]^+^ and HRTOFMS *m*/*z* 349.9146 [M + H]^+^ (calcd. for C_9_H_10_N_3_O_2_Br_2_, 349.9140).

*Stylisine E* (**5**): light yellow amorphous, [α]${}_{D}^{25}$ + 4.0 (*c* 0.05, MeOH); IR (KBr film) *ν*_max_ 3452, 1735, 1720, 1675, 1654, 1382, 1368, 1206, 1140 cm^−1^; ^1^H and ^13^C NMR (DMSO-*d*_6_), see Table 1, ESIMS *m*/*z* 349.91 [M + H]^+^ and HRTOFMS *m*/*z* 349.9146 [M + H]^+^ (calcd. for C_9_H_10_N_3_O_2_Br_2_, 349.9140).

*Stylisine F* (**6**): yellow solid, [α]${}_{D}^{25}$ − 19.0 (*c* 0.16, MeOH); IR (KBr film) *ν*_max_ 3351, 2927, 1713, 1680, 1624, 1470, 1205, 1138 cm^−1^; ^1^H and ^13^C NMR (DMSO-*d*_6_), see Table 1, ESIMS *m*/*z* 221.09 [M + H]^+^ and HRTOFMS *m*/*z* 221.0931 [M + H]^+^ (calcd. for C_11_H_13_N_2_O_3_, 221.0926).

### 4.4. X-ray Crystallographic Analysis 

Crystal data were obtained on a RigakuMicroMax 002+ single-crystal X-ray diffractometer. Cell parameter measurements and data collection were performed with a Bruker APEX2 CCD diffractometer using the wavelength for Cu K*α* (*λ* = 1.54184 A) radiation. The crystal structure of *N*-methylmanzacidin C was solved by direct methods (SHELXS-97), and subsequent Fourier difference techniques (SHELEX-97, version 6.10, Bruker AXS Inc. (Hong Kong, China)). The crystallographic data for the structures of *N*-methylmanzacidin C has been deposited in the Cambridge Crystallographic Data Center (CCDC number: 1543679). 

Light yellow crystals of *N*-methylmanzacidin C were obtained from MeOH/H_2_O (1:1) using the vapor diffusion method. Crystal data for *N*-methylmanzacidin C, colorless needles, mp: 200.5–201.5 °C, C_26_H_36_N_6_O_10_Br_2_ (2 C_13_H_16_O_4_N_3_Br + 2 H_2_O), M = 752.43, orthorhombic, space group P2_1_2_1_2_1_, *a* = 7.17211(6) A, *b* = 18.32553(14) A, *c* = 25.1534(2) A, *α* = 90°, *β* = 90°, *γ* = 90°, *V* = 3305.98 (**5**) A^3^, *Z* = 4, *T* = 160.0 K, *μ*(Cu Kα) = 3.635 mm^−1^, *D*_calc_ = 1.512 g/cm^3^, 28511 reflections measured (5.968 ≤ 2*θ* ≤ 137.558), 60,196 unique (*R*_int_ = 0.0334, *R*_sigma_ = 0.0202), which were used in all of the calculations. The final R_1_ was 0.0301 (I > 2*σ* (*I*)) and *w*R_2_ was 0.0745. Flack parameter = −0.019 (**6**).

### 4.5. ECD Calculation 

ECD data were calculated in the B3LYP/6-31+G(d) level in MeOH. Conformational analyses were carried out via random searching in the Sybyl-X 2.0 version using the MMFF94S force field with an energy cutoff of 3.0 kcal/mol. The ECD spectra were simulated by the overlapping Gaussian function (half the bandwidth at 1/e peak height, 0.16–0.3 eV). The simulated spectra of the two lowest energy conformers for each structure were averaged according to the Boltzmann distribution theory and their relative Gibbs free energy (ΔG). 

### 4.6. Antibacterial Assay 

The Gram positive bacteria involved *Staphylococcus aureus* ATCC 25923, *Bacillus subtilis* ATCC 11562, *Staphylococcus haemolyticus* ATCC 29970, while the Gram negative bacteria included *Pseudomonas aeruginosa* ATCC 9027, *Escherichia coli* ATCC 25922, *Pseudomonas lachrymans* ATCC 11921, *Agrobacterium tumefaciens* No. 8 ATCC 11158, *Xanthomonas vesicatoria* ATCC 11633, and *Ralstonia solanacearum* ATCC 11696. Each bacterium strain that was to be tested was streaked onto a lysogeny broth agar or Mueller-Hinton Broth agar plate, and was incubated at 37 °C for 24 h. One colony was then transferred to fresh lysogeny broth or Mueller-Hinton broth, and the cell density was adjusted to 10^4^–10^5^ cfu/mL. Compounds were dissolved in DMSO with 12.8 mg/mL, and then were serially diluted with freshly prepared microbial broth to give gradient concentrations of 128, 64, 32, 16, 8, and 4 μg/mL. An aliquot (160 μL) of each dilution was transferred to a 96-well plate. Each sample was repeated for four times with a final DMSO concentration of 0.03% in each well. The plates were incubated at 37 °C for 24 h and shaken at 200 rpm, the optical density of each well was measured at 620 nm. Each compound was screened against the Gram positive and negative bacteria, while chloramphenicol was used as positive control and 0.03% DMSO, 1% DMSO were used as negative control.

### 4.7. Inhibition of Biofilm Formation Assay

Inhibition assays were performed by taking an overnight culture of bacterial strain (*Escherichia coli* with Mueller-Hinton Broth, *Pseudomonas aeruginosa* with lysogeny broth) to an OD_620_ of 0.8–1.0 (1 × 10^8^ CFU/mL), the bacterial suspension was diluted by the broth to the cell density with an OD_620_ of 0.02–0.05. Compounds were dissolved in DMSO with 10 mg/mL, while the solution was then serially diluted with freshly prepared microbial broth to give final concentrations. Each solution was transferred (140 μL) into the wells of the 96-well plate by an incubation, under stationary conditions for 24 h at 30 °C. After incubation, the medium was discarded from the wells and the plates were washed thoroughly with water. Plates were then stained with 160 μL of 0.1% solution of crystal violet (CV), and then incubated at ambient temperature for 30 min. Plates were washed with water again and the remaining stain was solubilized with 200 μL of 95% ethanol. Biofilm inhibition was quantitated by measuring the OD_570_ of each well and calculated. Gentamicin was used as positive control against *Pseudomonas aeruginosa*, and chloramphenicol was used as positive control against *Escherichia coli*.

Dispersion assays were performed by taking an overnight culture of bacterial strain (*E. coli*) to an OD_620_ of 0.8–1.0 (1 × 10^8^ CFU/mL), the bacterial suspension was diluted by broth to the cell density, with an OD_620_ of 0.02–0.05. The bacterial suspension was transferred (130 μL) into the wells of a 96-well plate followed by an incubation under stationary conditions at 30 °C to establish the biofilms. After 24 h, the medium was discarded from the wells and the plates were washed thoroughly with water. Compounds were dissolved in DMSO with 10 mg/mL, while the solution was then serially diluted with freshly prepared microbial broth to give final concentrations. The solutions were aliquoted (140 μL) into the wells of the 96 well plate with the established biofilms. Medium alone was added to a subset of the wells to serve as a control. Plates were then incubated for 24 h at 30 °C. After incubation, the medium was discarded from the wells and the plates were washed thoroughly with water. Plates were then stained with 160 μL of 0.1% solution of crystal violet (CV), and then incubated at ambient temperature for 30 min. Plates were washed with water and the remaining stain was solubilized with 200 μL of 95% ethanol. Biofilm inhibition was quantitated by measuring the OD_570_ of each well and was calculated as a percentage of the blank control without the formation of biofilm.

### 4.8. Confocal Laser Scanning Microscope (CLSM) Assay

Inhibition assays were performed by taking an overnight culture of bacterial strain (*Escherichia coli* with Mueller-Hinton Broth) to an OD_620nm_ of 0.8–1.0 (1 × 10^8^ CFU/mL), the bacterial suspension was diluted by the broth to the cell density with an OD_620_ of 0.1. Compounds were dissolved in DMSO with 10 mg/mL. The compound solution (dibromophakellin and dibromoisophakellin) was then diluted with freshly prepared microbial broth to give a concentration of 200 μg/mL (positive control chloramphenicol of 40 μg/mL). Compound solution (1 mL) and bacterial solution (1 mL) were transferred into petri dishes to give final concentrations of 100 μg/mL (positive control chloramphenicol of 20 μg/mL, negative control of 100% DMSO). The dishes were incubated under stationary conditions for 24 h at 30 °C. After incubation, the medium was carefully discarded from the dishes, while the dishes were washed with 0.9% NaCl solution. Compound-treated biofilms were used for CLSM analysis. Biofilms were subsequently stained with the Filmtracer^TM^ LIVE/DEAD^®^ Biofilm Viability Kit for microscopy (invitrogen, molecular probes), according to the manufacturer’s instructions, and were observed by confocal laser scanning microscopy (Nikon A1plus) using 100× objective lens. The excitation/emission wavelength of SYTO9 stain was 488/525 nm and 561/595 nm for propidium iodide. The images were analyzed using NIS Elements software. This experiment was repeated for three times.

## 5. Conclusions

In summary, a comprehensively chromatographic separation of marine sponge *Stylissa massa* resulted in the isolation of 32 alkaloids with diverse scaffolds, including six new alkaloids. Bioassay results revealed that oroidin exhibited the inhibitory effects against a panel of bacteria. In addition, phakellin-based alkaloids dibromoisophakellin and dibromophakellin showed significant reduction against the biofilm formation of *E. coli* with weak inhibition toward planktonic bacteria, validating that the alkaloids were selectively killing biofilm bacteria to be acting via non-microbicidal mechanism. Present work firstly reported phakellin-based alkaloids to be new natural scaffold for biofilm inhibition.

## Figures and Tables

**Table 1:** ^1^H and ^13^C NMR data of **1**–**6** in dimethyl sulfoxide (DMSO-*d*_6_).

	1		2/3		4/5		6	
1	7.46, d (2.8)	127.38, CH		105.20, C		105.67, C	6.98, dd (2.4, 2.8)	121.52, CH
2		86.79, C		98.36, C		99.24, C	6.52, dd (2.0, 2.4)	109.32, CH
3		123.14, C	6.95, s	113.46,CH	6.84, s	113.86, CH		119.98, C
4		102.79, C		128.36, C		125.81, C		126.22, C
5		142.18, C		159.51, C		157.56, C		163.29, C
6	2.99, m; 2.87, m	28.42, CH_2_	3.43, m	42.36, CH_2_	3.77, dd (2.0, 13.20) 3.34, dd (5.2, 13.2)	42.06, CH_2_	3.48, dd (4.8, 7.2)	37.60, CH_2_
7	2.15, m	21.56, CH_2_	4.37, m	74.01, CH	4.64, m	50.59, CH	6.84, t (7.2)	132.54, CH
8	4.10, m; 3.98, m	48.27, CH_2_	5.76, brs	115.41, CH	2.63, dd (11.2, 12.8) 2.26, dd (1.5, 12.8)	36.16, CH_2_		131.59, C
9		152.56, C		128.58, C		170.25, C		165.98, C
10		126.39, C		153.49, C			4.21, q (7.0)	60.56, CH_2_
11		157.51, C		159.50, C			1.26, t (7.0)	14.08, CH_3_
NH	12.54, d (2.8)		12.68, s		7.87, brd (5.2)		11.85, dd (2.0, 2.8)	
NH			8.15, t (5.0)				7.73, t (4.8)	
NH (CN_3_H_4_)	9.16, s							
NH_2_ (CN_3_H_4_)	7.48, br		9.23, brs					
NH (CN_3_H_4_)	7.24, br							
CONH_2_					7.13, brs; 7.54, brs			

**Table 2:** Inhibitory effects of fractions against *E. coli* biofilm formation.

Sample	*E. coli* Biofilm Formation Inhibition Rate (%) ^a^
Positive control ^b^	98.4
*n*-BuOH fraction	7.7
F1	na
F2	84.4
F3	26.4
F4	18.2
F5	na

^a^ Measured in a dose of 200 μg/mL. ^b^ Positive control: chloramphenicol. Measured in a dose of 100 μg/mL.

**Table 3:** Inhibitory effects of alkaloids against *E. coli* biofilm formation.

Sample	*E. coli* Biofilm Formation Inhibition Rate (%) ^a^	IC_50_ (μg/mL)
Positive control	98.4 ± 0.8	
12	10.4 ± 1.7	>100
25	72.1 ± 2.8	50.9
26	76.5 ± 1.1	31.3
27	25.9 ± 2.4	>100

^a^ Measured in a dose of 100 μg/mL. Positive control: chloramphenicol.

## Supplementary Materials

The following are available online at www.mdpi.com/1660-3397/16/1/9/s1, HRESIMS, ^1^H, ^13^C NMR, HSQC, HMBC, ROESY, and IR spectra of the new alkaloids. Physical and spectroscopic data, HPLC chromatographs, ^1^H and ^13^C NMR, and 2D NMR spectra for known alkaloids.

## References

[1] Vaccari L., Allan D.B., Sharifi-Mood N., Singh A.R., Leheny R.L., Stebe K.J. (2015). Films of bacteria at interfaces: Three stages of behavior. Soft Matter.

[2] Kolter R. (2010). Biofilms in lab and nature: A molecular geneticist’s voyage to microbial ecology. Int. Microbiol..

[3] Koerdt A., Godeke J., Berger J., Thormann K.M., Albers S.J. (2010). Crenarchaeal biofilm formation under extreme conditions. PLoS ONE.

[4] Barros J., Grenho L., Manuel C.M., Ferreira C., Melo L.F., Nunes O.C., Monteiro F.J., Ferraz M.P. (2013). A modular reactor to simulate biofilm development in orthopedic materials. Int. Microbiol..

[5] Stewart P.S., Franklin M.J., Williamson K.S., Folsom J.P., Boegli L., James G.A. (2015). Contribution of stress responses to antibiotic tolerance in *Pseudomonas aeruginosa* biofilms. Antimicrob. Agents Chemother..

[6] Lebeaux D., Chauhan A., Rendueles O., Beloin C. (2013). From in vitro to in vivo models of bacterial biofilm-related infections. Pathogens.

[7] Stewart P.S. (2015). Antimicrobial tolerance in biofilms. Microbiol. Spectr..

[8] Stowe S.D., Richards J.J., Tucker A.T., Thompson R., Melander C., Cavanagh J. (2011). Anti-biofilm compounds derived from marine sponges. Mar. Drugs.

[9] Balskus E.P. (2014). Sponge symbionts play defense. Nat. Chem. Biol..

[10] Davies D. (2003). Understanding biofilm resistance to antibacterial agents. Nat. Rev. Drug Discov..

[11] Fusetani N. (2004). Biofouling and antifouling. Nat. Prod. Rep..

[12] Laport M.S., Santos O.C.S., Muricy G. (2009). Marine sponges: Potential sources of new antimicrobial drugs. Curr. Pharm. Biotechnol..

[13] Hertiani T., Edrada-Ebel R., Ortlepp S., van Soest R.W., de Voogd N.J., Wray V., Hentschel U., Kozytska S., Müller W.E.G., Proksch P. (2010). From anti-fouling to biofilm inhibition: New cytotoxic secondary metabolites from two Indonesian *Agelas* sponges. Bioorg. Med. Chem..

[14] Tsukamoto S., Kato H., Hirota H., Fusetani N. (1996). Ceratinamides A and B: New antifouling dibromotyrosine derivatives from the marine sponge *Pseudoceratina purpurea*. Tetrahedron.

[15] Shalmali N., Ali M.R., Bawa S. (2017). Imidazole: An essential edifice for the identification of new lead compounds and drug development. Mini Rev. Med. Chem..

[16] Lindel T. (2017). Chemistry and biology of the pyrrole-imidazole alkaloids. Alkaloids Chem. Biol..

[17] Cychon C., Lichte E., Köck M. (2015). The marine sponge *Agelas citrina* as a source of the new pyrrole-imidazole alkaloids citrinamines A–D and *N*-methylagelongine. Beilstein J. Org. Chem..

[18] Assmann M., Soest R.W.M., Kock M. (2001). New antifeedant bromopyrrole alkaloid from the Caribbean sponge *Stylissa caribica*. J. Nat. Prod..

[19] Melander R.J., Liu H., Stephens M.D., Bewley C.A., Melander C. (2016). Marine sponge alkaloids as a source of anti-bacterial adjuvants. Bioorg. Med. Chem. Lett..

[20] Hodnik Z., Łos J.M., Zula A., Zidar N., Jakopin Z., Łos M., Dolenc M.S., Ilaš J., Wegrzyn G., Mašic L.P. (2014). Inhibition of biofilm formation by conformationally constrained indole-based analogues of the marine alkaloid oroidin. Bioorg. Med. Chem. Lett..

[21] Forte B., Malgesini B., Piutti C., Quartieri F., Scolaro A., Papeo G. (2009). A submarine journey: The pyrrole-imidazole alkaloids. Mar. Drugs.

[22] Zhang H., Khalil Z., Conte M.M., Plisson F., Capon R.J. (2012). A search for kinase inhibitors and antibacterial agents: Bromopyrrolo-2-aminoimidazoles from a deep-water Great Australian Bight sponge, *Axinella* sp.. Tetrahedron Lett..

[23] Autschbach J., Nitsch-Velasquez L., Rudolph M. (2011). Time-dependent density functional response theory for electronic chiroptical properties of chiral molecules. Top. Curr. Chem..

[24] Pescitelli G., Di Pietro S., Cardellicchio C., Capozzi M.A., Di Bari L. (2010). Systematic investigation of CD spectra of aryl benzyl sulfoxides interpreted by means of TDDFT calculations. J. Org. Chem..

[25] Tsukamoto S., Kato H., Hirota H., Fusetani N. (1996). Mauritiamine, a new antifouling oroidin dimer from the marine sponge *Agelas mauritiana*. J. Nat. Prod..

[26] Mancini I., Guella G., Amade P., Roussakis C., Pietra F. (1997). Hanishin, a semiracemic, bioactive C_9_ alkaloid of the axinellid sponge *Acanthella carteri* from the Hanish Islands. A shunt metabolite?. Tetrahedron Lett..

[27] Forenza S., Minale L., Riccio R., Fattorusso E. (1971). New bromopyrrole derivatives from the sponge *Agela soroides*. J. Chem. Soc. Chem. Commun..

[28] Kobayashi J., Ohizumi Y., Nakamura H., Hirata Y. (1986). A novel antagonist of serotonergic receptors, hymenidin, isolated from the Okinawan marine sponge *Hymeniacidon* sp.. Experientia.

[29] Cafieri F., Fattorusso E., Mangoni A., Taglialatela-Scafati O. (1996). Dispacamides, anti-histamine alkaloids from caribbean *Agelas* sponges. Tetrahedron Lett..

[30] Nakamura H., Ohizumi Y., Kobayashi J., Hirata Y. (1984). Keramadine, a novel antagonist of serotonergic receptors isolated from the okinawan sea sponge *Agelas* sp.. Tetrahedron Lett..

[31] Williams D.E., Patrick B.O., Behrisch H.W., Soest R.V., Roberge M., Andersen R.J. (2005). Dominicin, a cyclic octapeptide, and laughine, a bromopyrrole alkaloid, isolated from the Caribbean marine sponge *Eurypon laughlini*. J. Nat. Prod..

[32] Fattorusso E., Taglialatela-Scafati O. (2000). Two novel pyrrole-imidazole alkaloids from the Mediterranean sponge *Agela soroides*. Tetrahedron Lett..

[33] Fouad M.A., Debbab A., Wray V., Müller W.E.G., Proksch P. (2012). New bioactive alkaloids from the marine sponge *Stylissa* sp.. Tetrahedron.

[34] Tasdemir D., Mallon R., Greenstein M., Feldberg L.R., Kim S.C., Collins K., Wojciechowicz D., Mangalindan G.C., Concepción G.P., Harper M.K. (2002). Aldisine alkaloids from the Philippine sponge *Stylissa massa* are potent inhibitors of mitogen-activated protein kinase kinase-1 (MEK-1). J. Med. Chem..

[35] Kobayashi J., Ohizumi Y., Nakamura H., Hirata Y., Wakamatsu K., Miyazawa T. (1986). Hymenin, a novel α-adrenoceptor blocking agent from the Okinawan marine sponge *Hymeniacidon* sp.. Experientia.

[36] Albizatit K.F., Faulkner D.J. (1985). Stevensine, a novel alkaloid of an unidentified marine sponge. J. Org. Chem..

[37] Eder C., Proksch P., Wray V., Klaus Steube K., Bringmann G., Soest R.W.M., Ferdinandus E., Pattisina L.A., Wiryowidagdo S., Moka W. (1999). New alkaloids from the indopacific sponge *Stylissa carteri*. J. Nat. Prod..

[38] Meyer S.W., Köck M. (2008). NMR Studies of phakellins and isophakellins. J. Nat. Prod..

[39] Kobayashi J., Kanda F., Ishibashi M., Shigemori H. (1991). Manzacidins A–C, novel tetrahydropyrimidine alkaloids from the Okinawan marine sponge *Hymeniacidon* sp.. J. Org. Chem..

[40] Tsukamoto S., Tane K., Ohta T., Matsunaga S., Fusetani N., Soest R.W.M. (2001). Four new bioactive pyrrole-derived alkaloids from the marine sponge *Axinella brevistyla*. J. Nat. Prod..

[41] Zhu Y., Wang Y., Gu B., Yang F., Jiao W., Hu G., Yu H., Han B., Zhang W., Shen Y. (2016). Antifungal bromopyrrole alkaloids from the South China Sea sponge *Agelas* sp.. Tetrahedron.

[42] Andrade P., Willoughby R., Pomponi S.A., Kerr R.G. (1999). Biosynthetic studies of the alkaloid, stevensine, in a cell culture of the marine sponge *Teichaxinella morchella*. Tetrahedron Lett..

[43] Stout E.P., Wang Y., Romo D., Molinski T.F. (2012). Pyrrole aminoimidazole alkaloid metabiosynthesis with marine sponges *Agelas conifera* and *Stylissa caribica*. Angew. Chem. Int. Ed..

[44] Genta-Jouve G., Cachet N., Holderith S., Oberhansli F., Teyssie J.L., Jeffree R., Mourabit A.A., Thomas O.P. (2011). New insight into marine alkaloid metabolic pathways: Revisiting oroidin biosynthesis. ChemBioChem.

[45] Richards J.J., Reed C.S., Christian Melander C. (2008). Effects of *N*-pyrrole substitution on the anti-biofilm activities of oroidin derivatives against *Acinetobacter baumannii*. Bioorg. Med. Chem. Lett..

[46] Ballard T.E., Richards J.J., Aquino A., Reed C.S., Melander C. (2009). Antibiofilm activity of a diverse oroidin library generated through reductive acylation. J. Org. Chem..

[47] Stiefel P., Rosenberg U., Schneider J., Mauerhofer S., Maniura-Weber K., Ren Q. (2016). Is biofilm removal properly assessed? Comparison of different quantification methods in a 96-well plate system. Appl. Microbiol. Biotechnol..

[48] Shen Y., Stojicic S., Haapasalo M. (2010). Bacterial viability in starved and revitalized biofilms: Comparison of viability staining and direct culture. J. Endod..

[49] Sauleau P., Moriou C., Mourabit A.A. (2017). Metabolomics approach to chemical diversity of the Mediterranean marine sponge *Agelas oroides*. Nat. Prod. Res..

[50] Pina I.C., White K.N., Cabrera G., Rivero E., Crews P. (2007). Bromopyrrole carboxamide biosynthetic products from the Caribbean sponge *Agelas dispar*. J. Nat. Prod..

[51] Richelle-Maurer E., De Kluijver M.J., Feio S., Gaudencio S., Gaspar H., Gomez R., Tavares R., Van de Vyver G., Van Soest R.W.M. (2003). Localization and ecological significance of oroidin and sceptrin in the Caribbean sponge *Agelas conifer*. Biochem. Syst. Ecol..

[52] Kelly S.R., Jensen P.R., Henkel T.P., Fenical W., Pawlik J.R. (2003). Effects of Caribbean sponge extracts on bacterial attachment. Aquat. Microb. Ecol..

[53] Yamada A., Kitamura H., Yamaguchi K., Fukuzawa S., Kamijima C., Yazawa K., Kuramoto M., Wang G.-Y.-S., Fujitani Y., Uemura D. (1997). Development of chemical substances regulating biofilm formation. Bull. Chem. Soc. Jpn..

[54] Richards J.J.T., Ballard E.B., Huigens R.W., Melander C. (2008). Synthesis and screening of an oroidin library against *Pseudomonas aeruginosa* biofilms. ChemBioChem.

