# Supplementary material for: Bromopyrrole Alkaloids with the Inhibitory Effects against the Biofilm Formation of Gram Negative Bacteria
Source: Mar Drugs. 2018 Jan 2;16(1):9. doi: 10.3390/md16010009 (PMC5793057; doi:10.3390/md16010009)
Supplement: Supplementary file 1 [file marinedrugs-16-00009-s001.pdf]

Bromopyrrole Alkaloids with the Inhibitory Effects against the Biofilm Formation of Gram Negative Bacteria

Supporting Information

Table S1 Yield of each alkaloid isolated from the sponge

Figure S1. ^{1}H-NMR spectrum of **1** (DMSO-d_6, 400 MHz)

Figure S2. ^{13}C-NMR spectrum of **1** (DMSO-d_6, 100 MHz)

Figure S3. ^{1}H-^{1}H COSY spectrum of **1** (DMSO-d_6, 400 MHz)

Figure S4. HSQC spectrum of **1** (DMSO-d_6, 400 MHz)

Figure S5. HMBC spectrum of **1** (DMSO-d_6, 400 MHz)

Figure S6. Enlarged HMBC spectrum of **1** (DMSO-d_6, 400MHz)

Figure S7. Enlarged HMBC spectrum of **1** (DMSO-d_6, 400 MHz)

Figure S8. Enlarged HMBC spectrum of **1** (DMSO-d_6, 400 MHz)

Figure S9. HR-ESIMS spectrum of **1**

Figure S10. IR spectrum of **1**

Figure S11. ^{1}H-NMR spectrum of **2/3** (DMSO-d_6, 400 MHz)

Figure S12. ^{13}C-NMR spectrum of **2/3** (DMSO-d_6, 100 MHz)

Figure S13. ^{1}H-^{1}H COSY spectrum of **2/3** (DMSO-d_6, 400 MHz)

Figure S14. Enlarged ^{1}H-^{1}H COSY spectrum of **2/3** (DMSO-d_6, 400 MHz)

Figure S15. HSQC spectrum of **2/3** (DMSO-d_6, 400 MHz)

Figure S16. HMBC spectrum of **2/3** (DMSO-d_6, 400 MHz)

Figure S17. Enlarged HMBC spectrum of **2/3** (DMSO-d_6, 400 MHz)

Figure S18. Enlarged HMBC spectrum of **2/3** (DMSO-d_6, 400 MHz)

Figure S19. HR-ESIMS spectrum of **2/3**

Figure S20. IR spectrum of **2/3**

Figure S21. ^{1}H-NMR spectrum of **4/5** (DMSO-d_6, 400 MHz)

Figure S22. ^{13}C-NMR spectrum of **4/5** (DMSO-d_6, 100 MHz)

Figure S23. ^{1}H-^{1}H COSY spectrum of **4/5** (DMSO-d_6, 400 MHz)

Figure S24. HSQC spectrum of **4/5** (DMSO-d_6, 400 MHz)

Figure S25. HMBC spectrum of **4/5** (DMSO-d_6, 400 MHz)

Figure S26. HR-ESIMS spectrum of **4/5**

Figure S27. IR spectrum of **4/5**

Figure S28. ^{1}H-NMR spectrum of **6** (DMSO-d_6, 400MHz)

Figure S29. ^{13}C-NMR spectrum of **6** (DMSO-d_6, 100 MHz)

Figure S30. ^{1}H-^{1}H COSY spectrum of **6** (DMSO-d_6, 400 MHz)

Figure S31. Enlarged ^{1}H-^{1}H COSY spectrum of **6** (DMSO-d_6, 400 MHz)

Figure S32. Enlarged ^{1}H-^{1}H COSY spectrum of **6** (DMSO-d_6, 400 MHz)

Figure S33. HSQC spectrum of **6** (DMSO-d_6, 400 MHz)

Figure S34. HMBC spectrum of **6** (DMSO-d_6, 400MHz)

Figure S35. Enlarged HMBC spectrum of **6** (DMSO-d_6, 400MHz)

Figure S36. HR-ESIMS spectrum of **6**

Figure S37. IR spectrum of **6**

Figure S38. Chiral HPLC separation of **2** and **3**

Figure S39. Chiral HPLC separation of **4** and **5**

Physical and spectroscopic data for known compounds

HPLC chromatograph spectra, 1D and 2D NMR spectra, ESIMS spectra of known compounds

Table S1 Yield of each alkaloid isolated from the sponge

compounds	Amount (mg)	Yield (%)
**1**	21.1	0.60
**2**	2.4	0.08
**3**	2.5	0.08
**4**	2.0	0.06
**5**	1.8	0.05
**6**	4.7	0.13
**7**	6.0	0.17
**8**	3.1	0.09
**9**	39.1	1.12
**10**	99.2	2.83
**11**	26	0.74
**12**	7.2	0.21
**13**	8.6	0.25
**14**	17.5	0.50
**15**	9.6	0.27
**16**	29.3	0.84
**17**	11.3	0.32
**18**	3.3	0.10
**19**	6.7	0.19
**20**	13.5	0.39
**21**	7.6	0.22
**22**	2.9	0.08
**23**	17.5	0.50
**24**	9.9	0.28
**25**	11.7	0.33
**26**	117.5	3.36
**27**	30.8	0.88
**28**	1.2	0.03
**29**	11.9	0.34
**30**	39.7	1.13
**31**	2.0	0.06
**32**	3.6	0.10

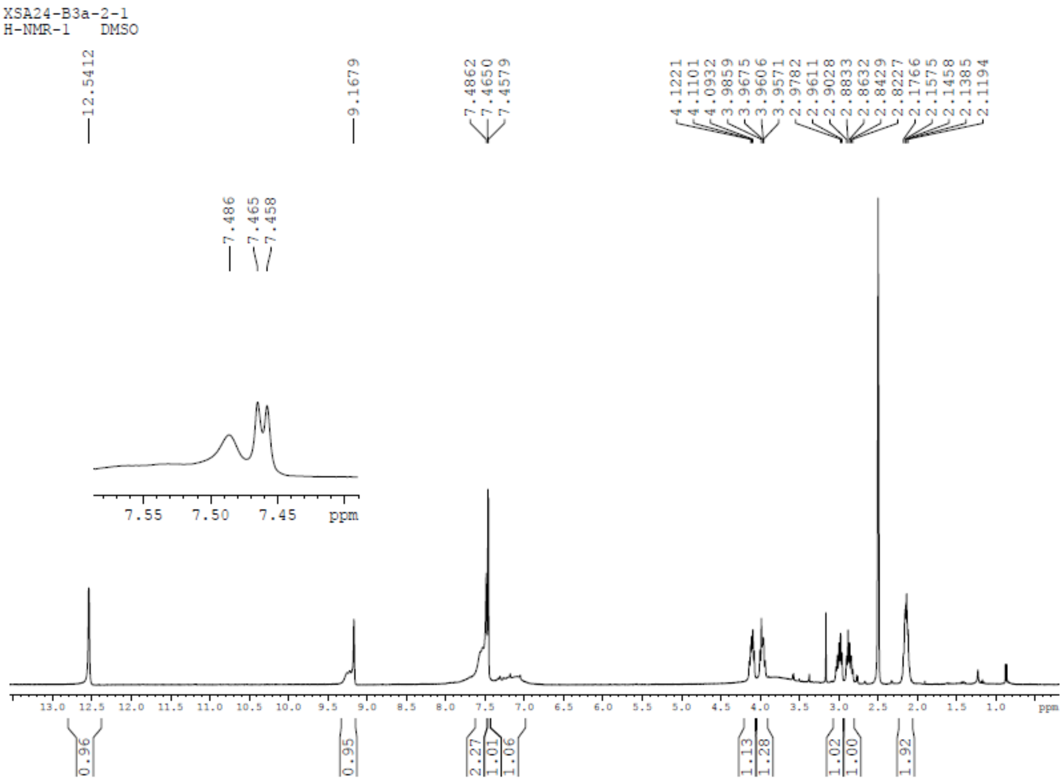

Figure S1. ¹H-NMR spectrum of **1** (DMSO-d_6, 400MHz)

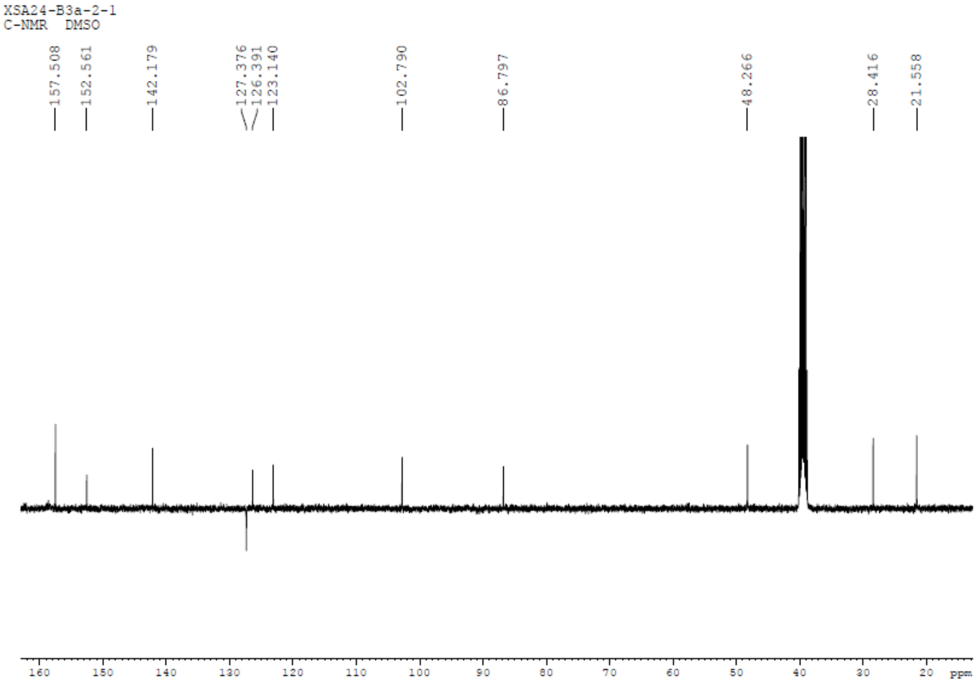

Figure S2. ¹³C-NMR spectrum of **1** (DMSO-d_6, 100MHz)

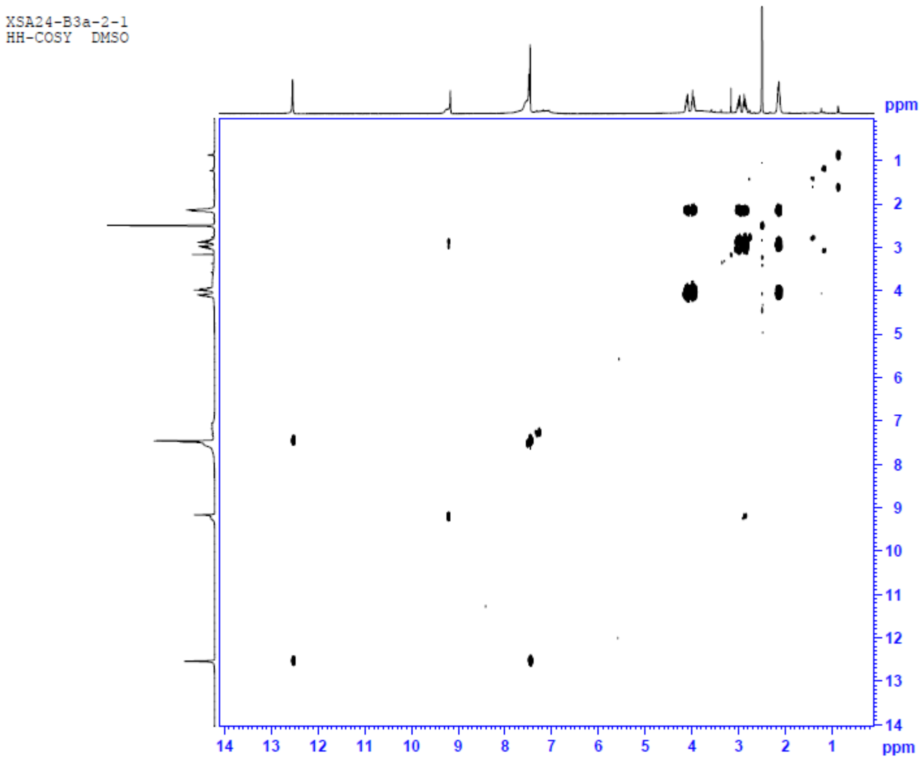

Figure S3. ^{1}H-^{1}H COSY spectrum of **1** (DMSO-d_6, 400MHz)

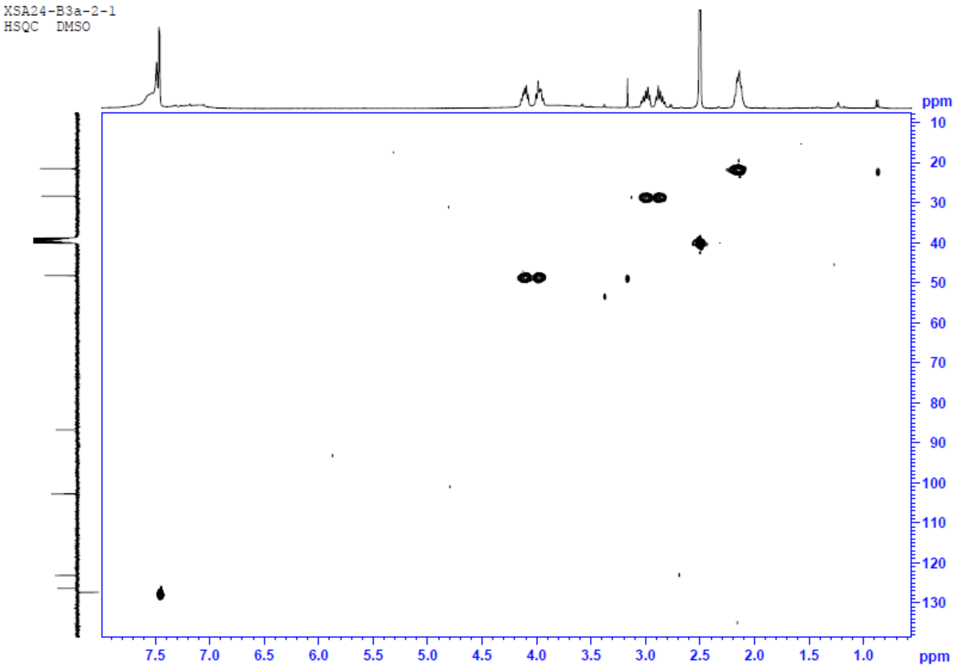

Figure S4. HSQC spectrum of **1** (DMSO-d_6, 400MHz)

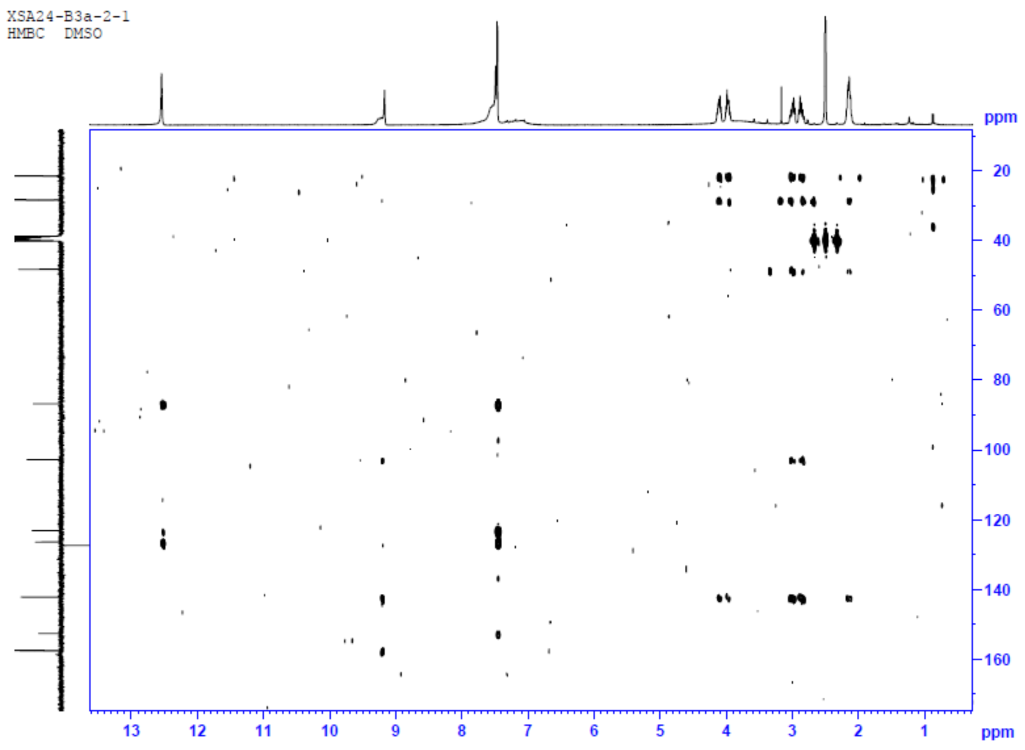

Figure S5. HMBC spectrum of **1** (DMSO-d_6, 400MHz)

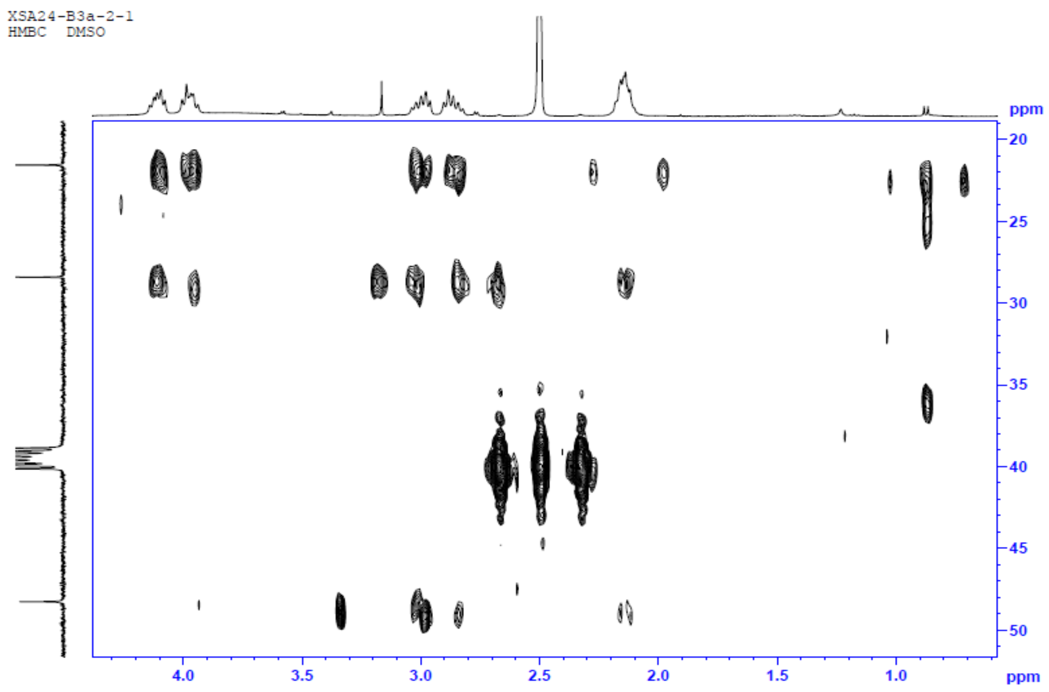

Figure S6. Enlarged HMBC spectrum of **1** (DMSO-d_6, 400MHz)

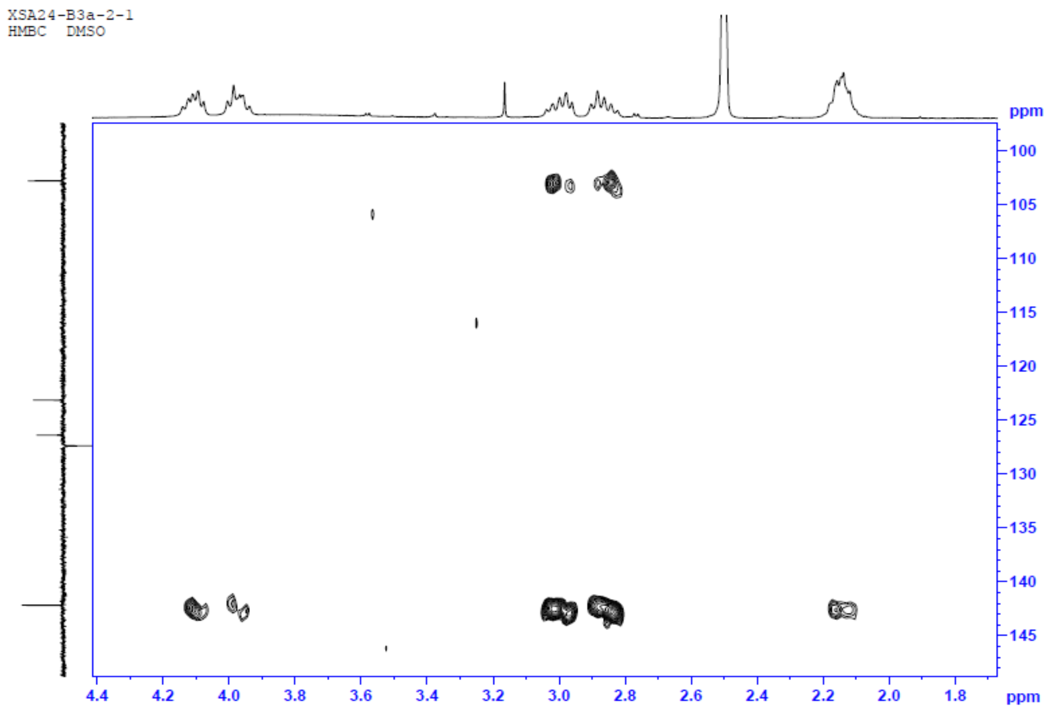

Figure S7. Enlarged HMBC spectrum of **1** (DMSO-d_6, 400MHz)

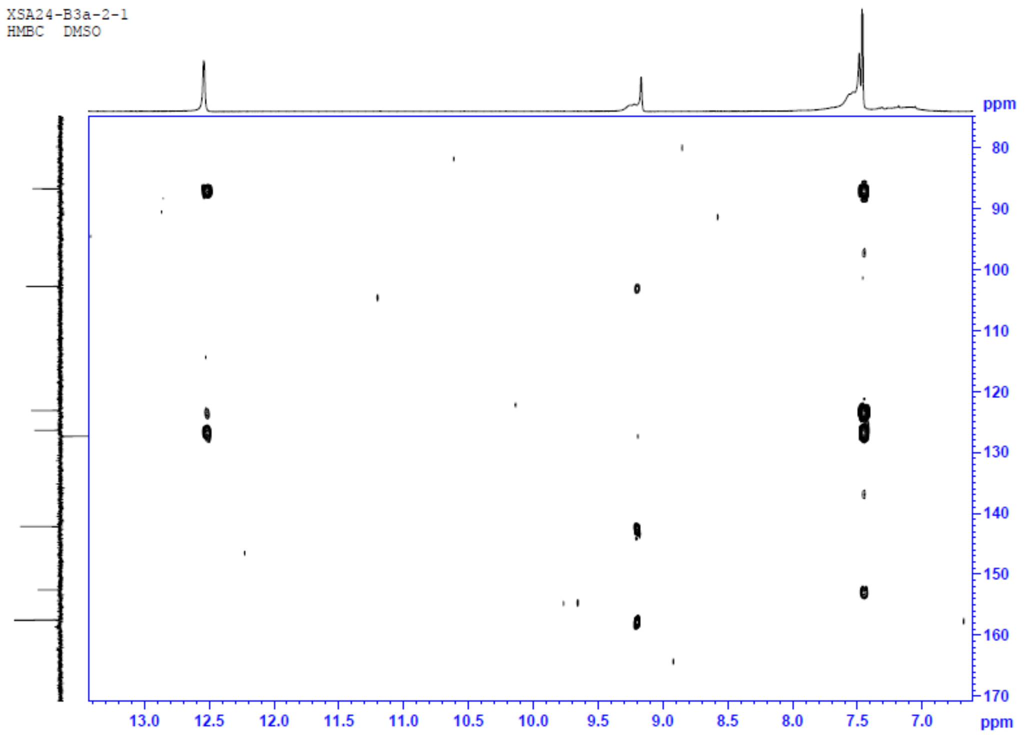

Figure S8. Enlarged HMBC spectrum of **1** (DMSO-d_6, 400MHz)

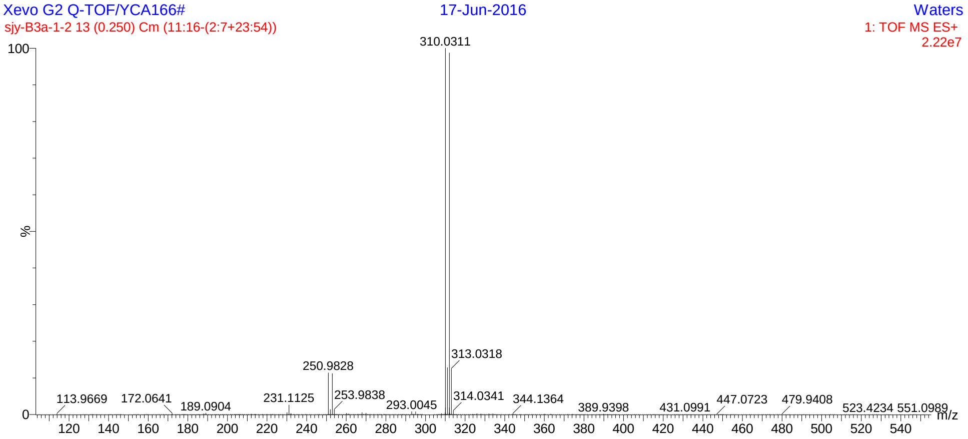

Mass	Calc. Mass	mDa	PPM	DBE	i-FIT	Norm	Conf(%)	Formula
310.0311	310.0303	0.8	2.6	7.5	637.5	0.000	100.00	$C_{11}H_{13}N_5OBr$

Figure S9. HR-ESIMS spectrum of **1**

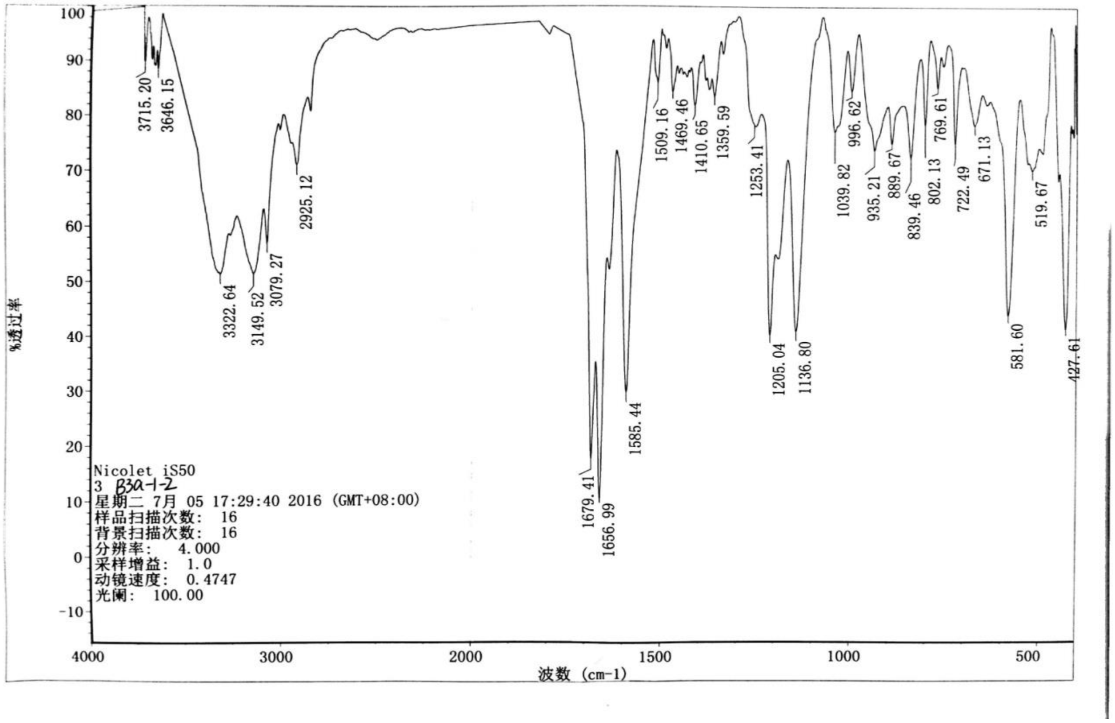

Figure S10. IR spectrum of **1**

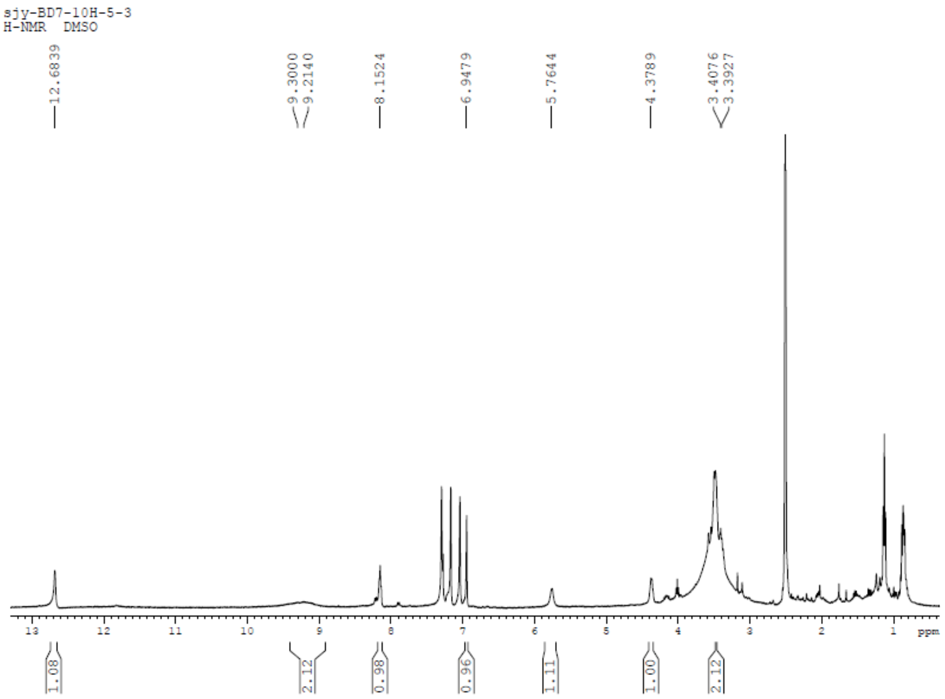

Figure S11. ^{1}H-NMR spectrum of **2/3** (DMSO-d_6, 400MHz)

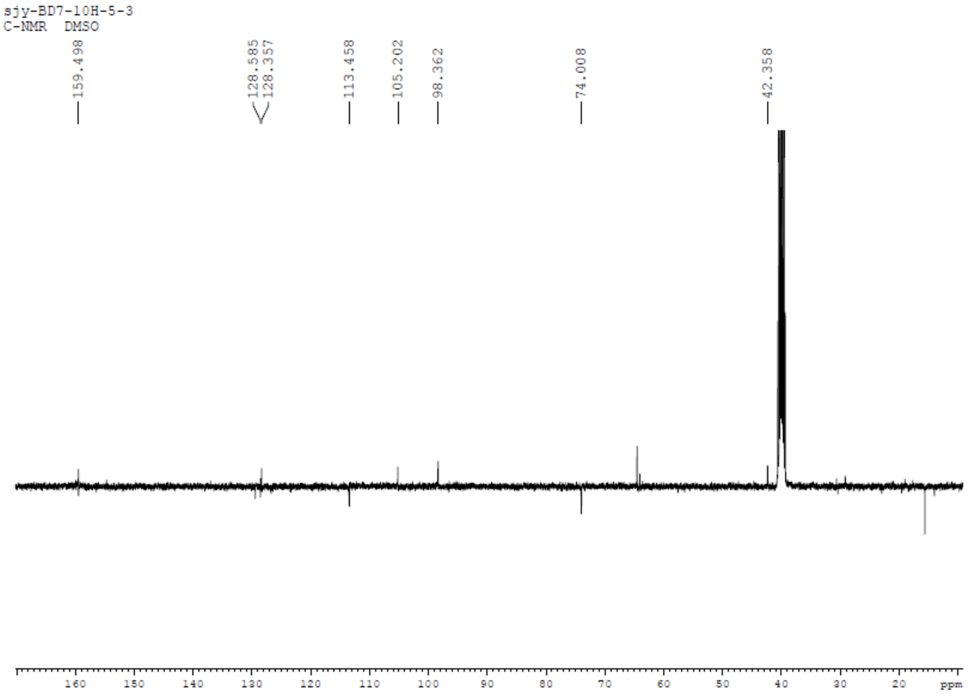

Figure S12. ^{13}C-NMR spectrum of **2/3** (DMSO-d_6, 100MHz)

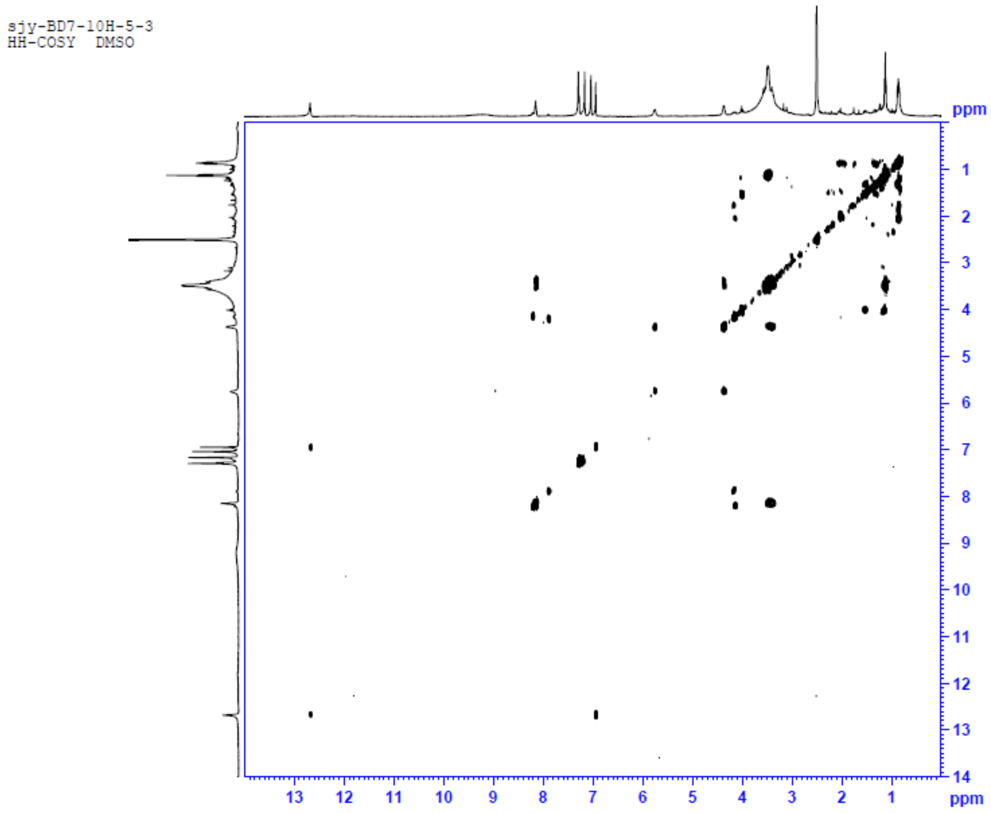

Figure S13. ^{1}H-^{1}H COSY spectrum of **2/3** (DMSO-d_6, 400MHz)

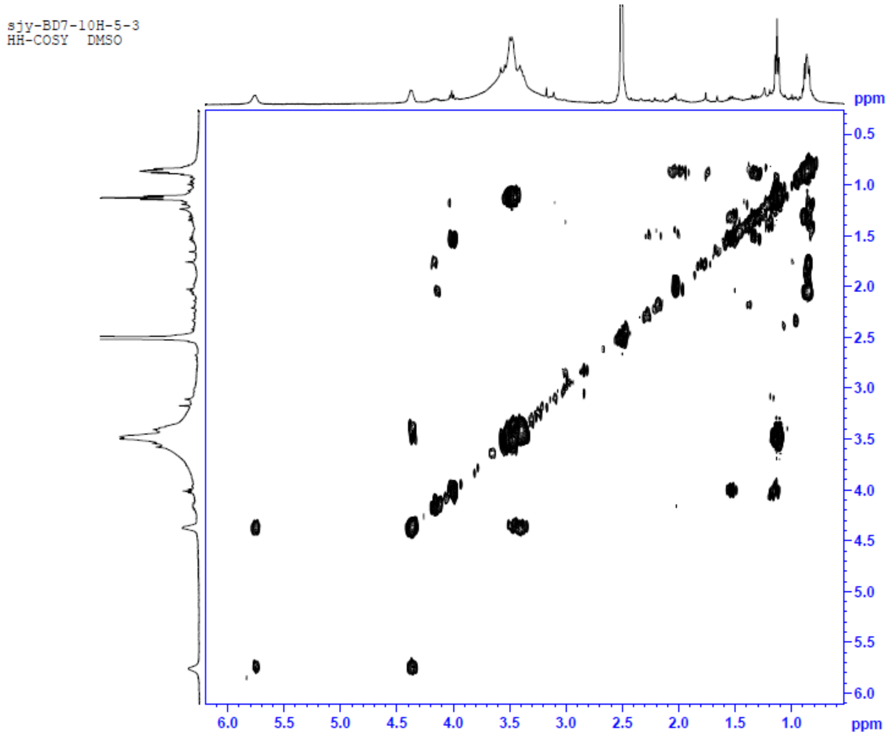

Figure S14. Enlarged ^{1}H-^{1}H COSY spectrum of **2/3** (DMSO-d_6, 400MHz)

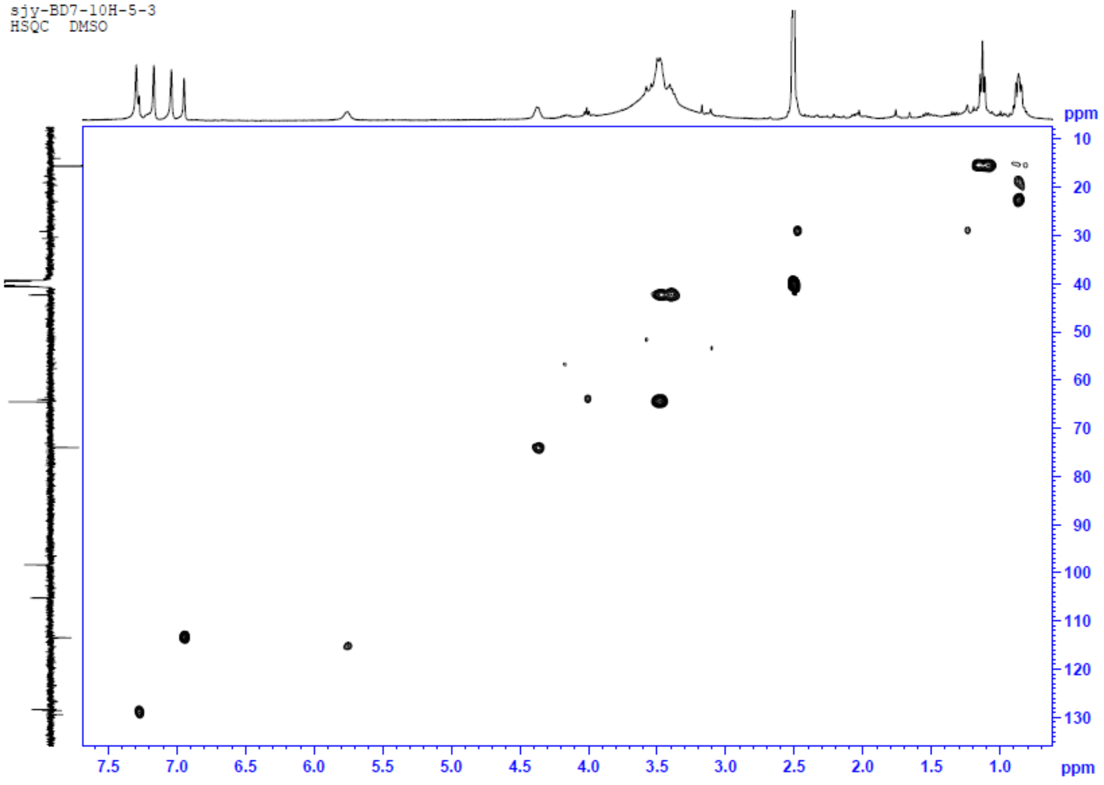

Figure S15. HSQC spectrum of **2/3** (DMSO-d_6, 400MHz)

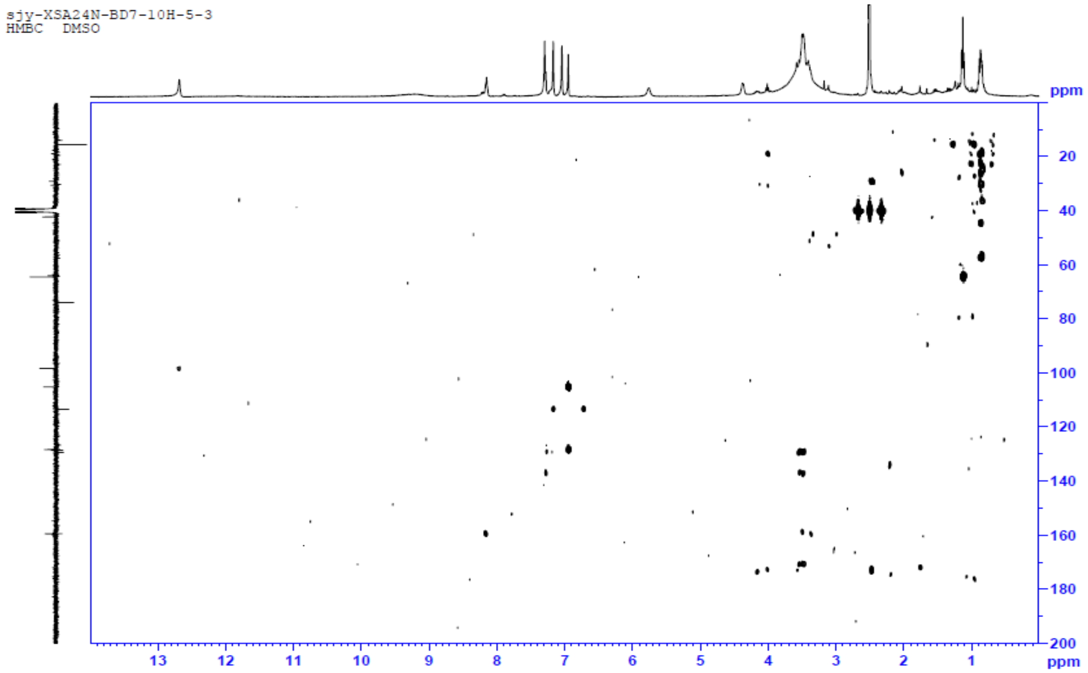

Figure S16. HMBC spectrum of **2/3** (DMSO-d_6, 400MHz)

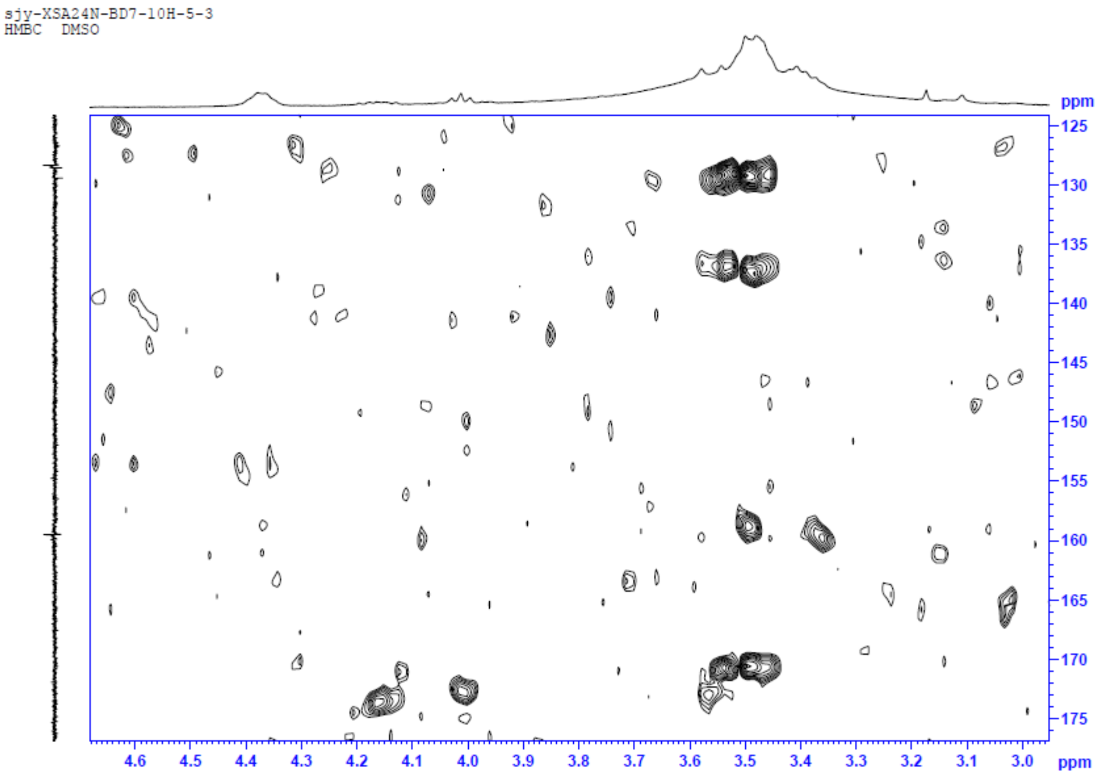

Figure S17. Enlarged HMBC spectrum of **2/3** (DMSO-d_6, 400MHz)

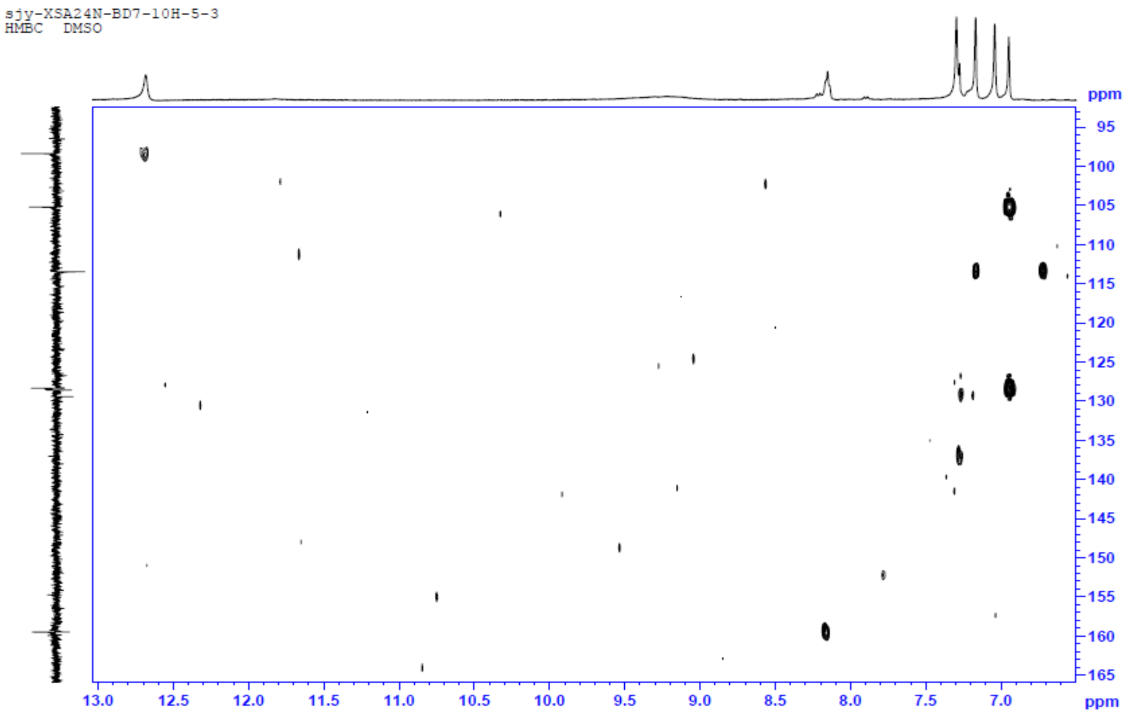

Figure S18. Enlarged HMBC spectrum of **2/3** (DMSO-d_6, 400MHz)

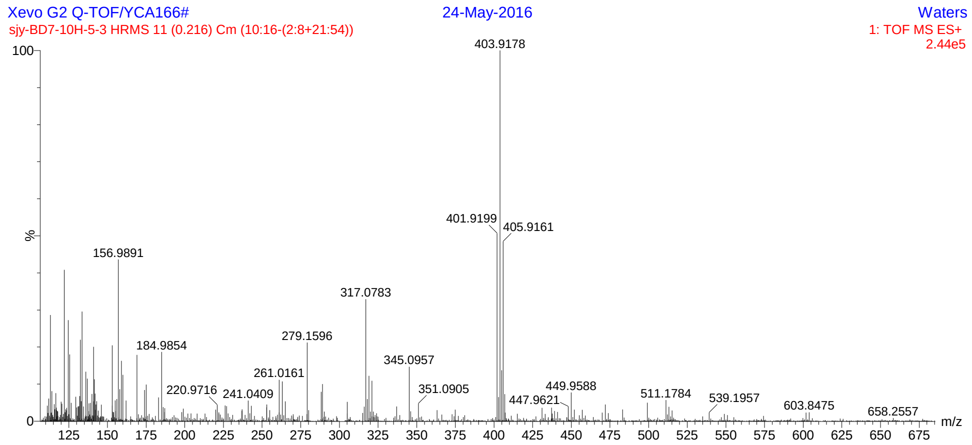

Mass	Calc. Mass	mDa	PPM	DBE	i-FIT	Norm	Conf(%)	Formula
401.9199	401.9201	-0.2	-0.5	8.5	274.7	0.250	77.86	$C_{11}H_{10}N_5O_2Br_2$

Figure S19. HR-ESIMS spectrum of **2/3**

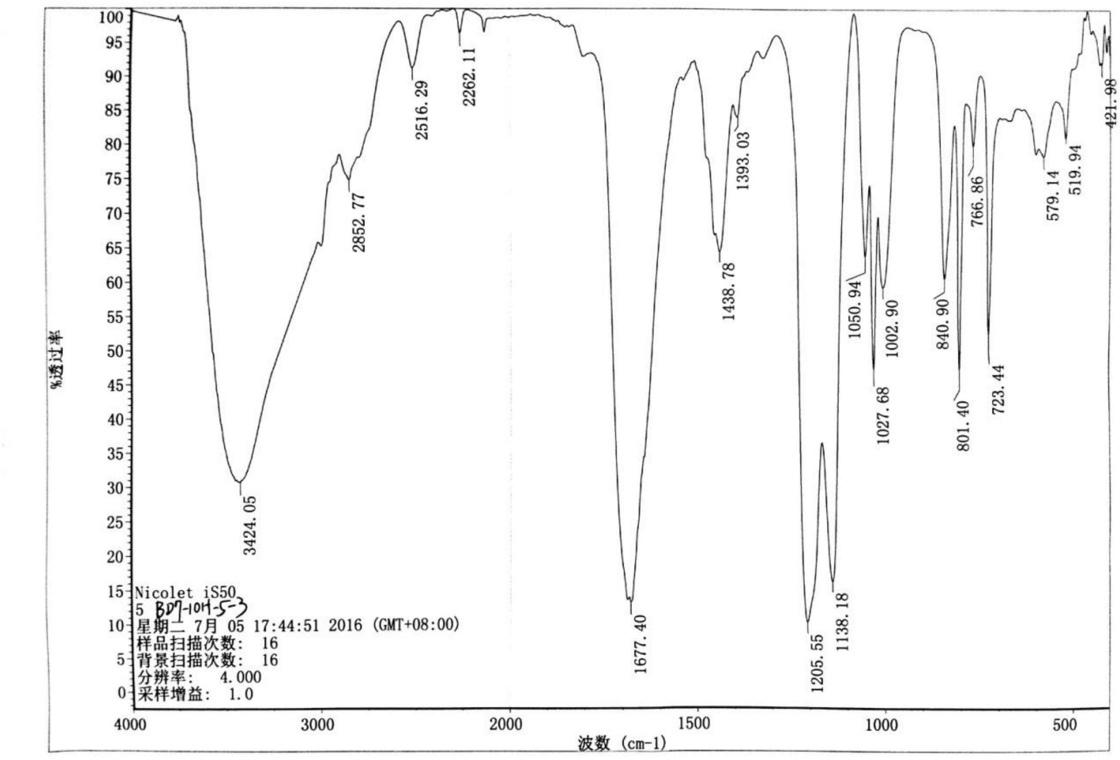

Figure S20. IR spectrum of **2/3**

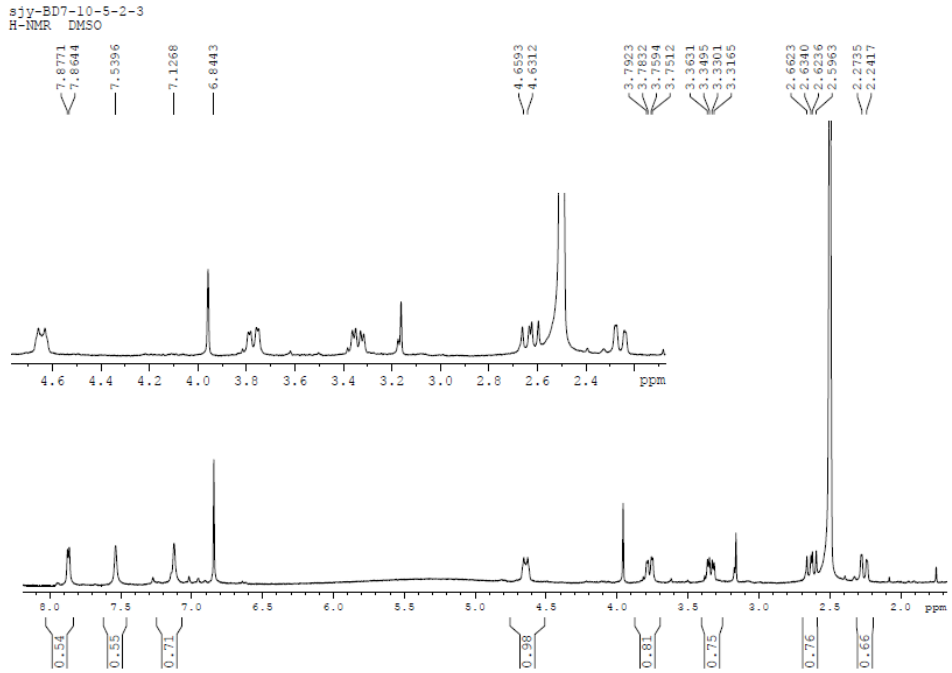

Figure S21. ^{1}H-NMR spectrum of **4/5** (DMSO-d_6, 400MHz)

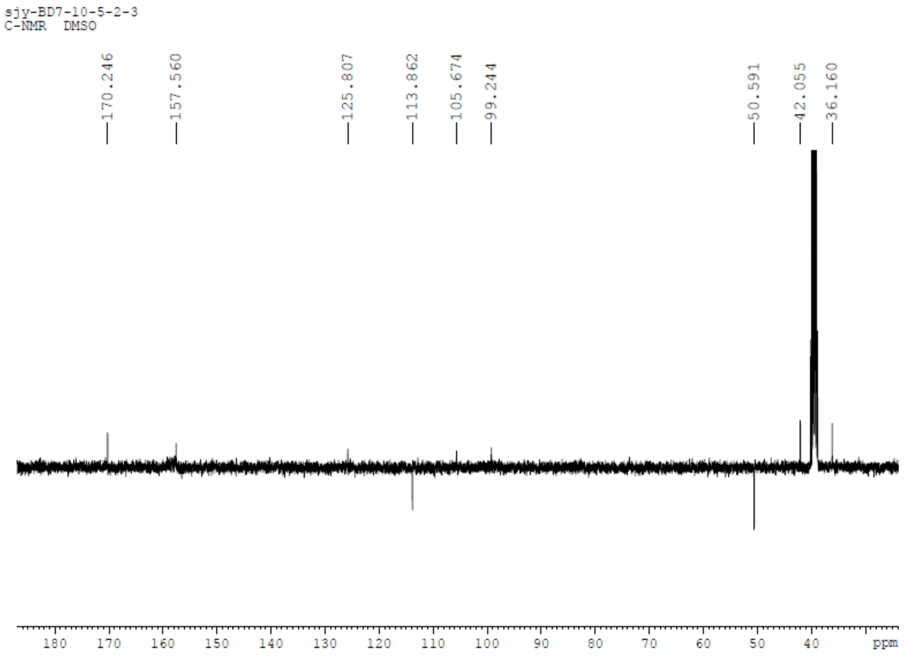

Figure S22. ^{13}C-NMR spectrum of **4/5** (DMSO-d_6, 100MHz)

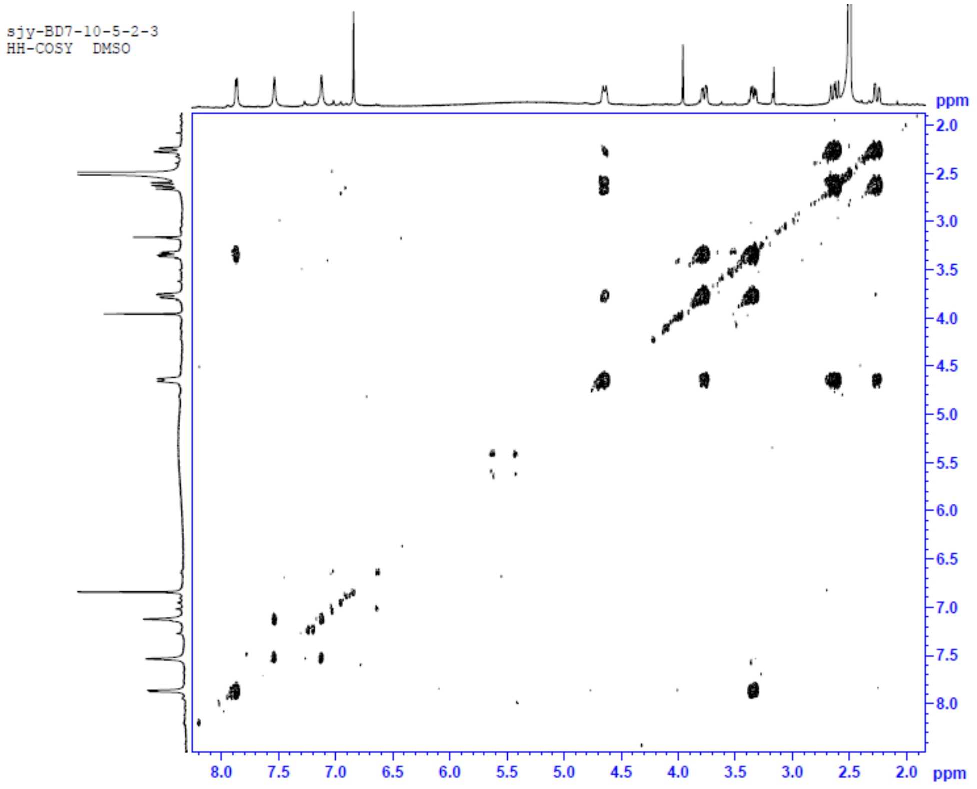

Figure S23. ^{1}H-^{1}H COSY spectrum of **4/5** (DMSO-d_6, 400MHz)

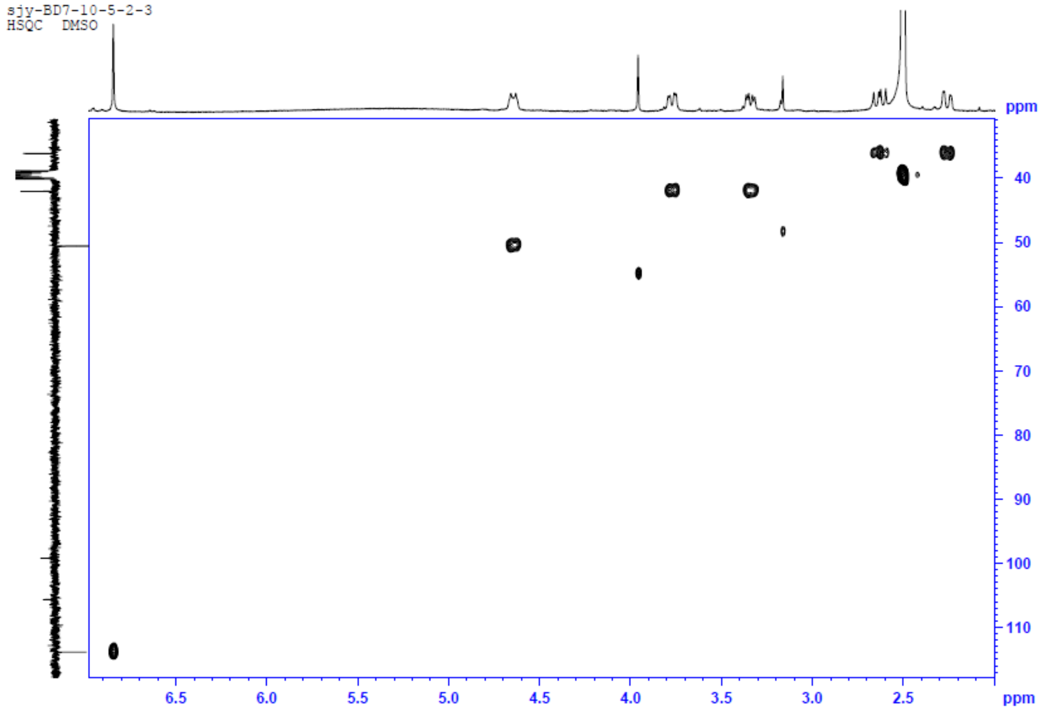

Figure S24. HSQC spectrum of **4/5** (DMSO-d_6, 400MHz)

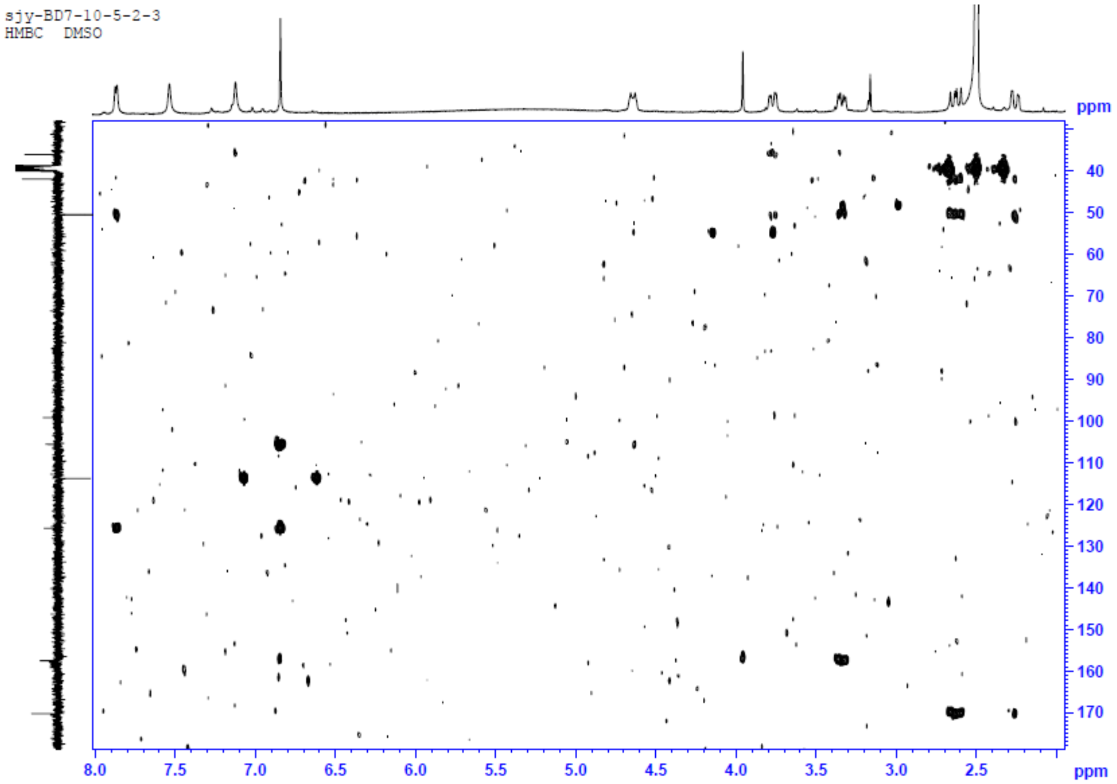

Figure S25. HMBC spectrum of **4/5** (DMSO-d_6, 400 MHz)

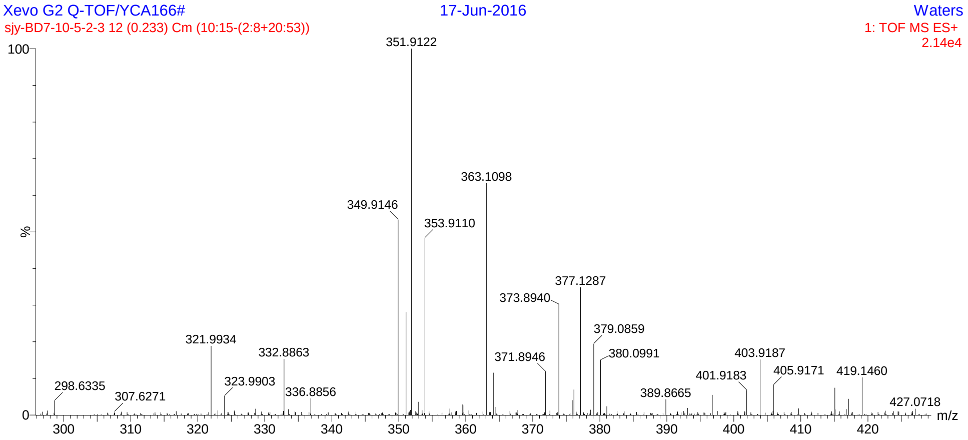

Mass	Calc. Mass	mDa	PPM	DBE	i-FIT	Norm	Conf(%)	Formula
349.9146	349.9140	0.6	1.7	5.5	117.1	0.490	61.25	$C_9H_{10}N_3O_2Br_2$

Figure S26. HR-ESIMS spectrum of **4/5**

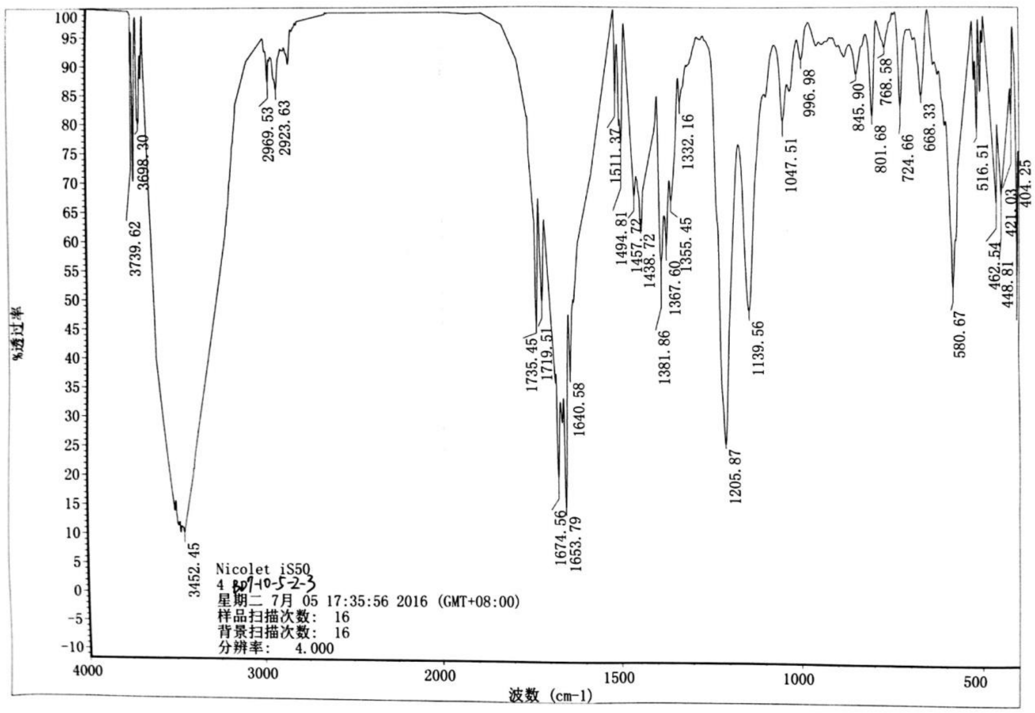

Figure S27. IR spectrum of **4/5**

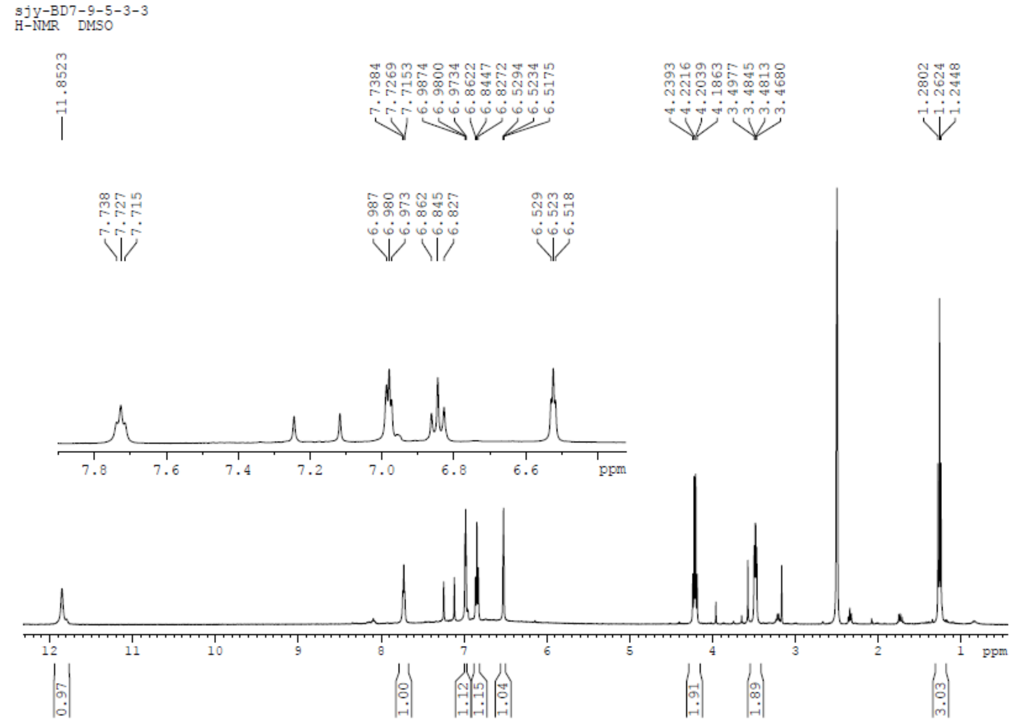

Figure S28. ^{1}H-NMR spectrum of **6**(DMSO-d_6, 400MHz)

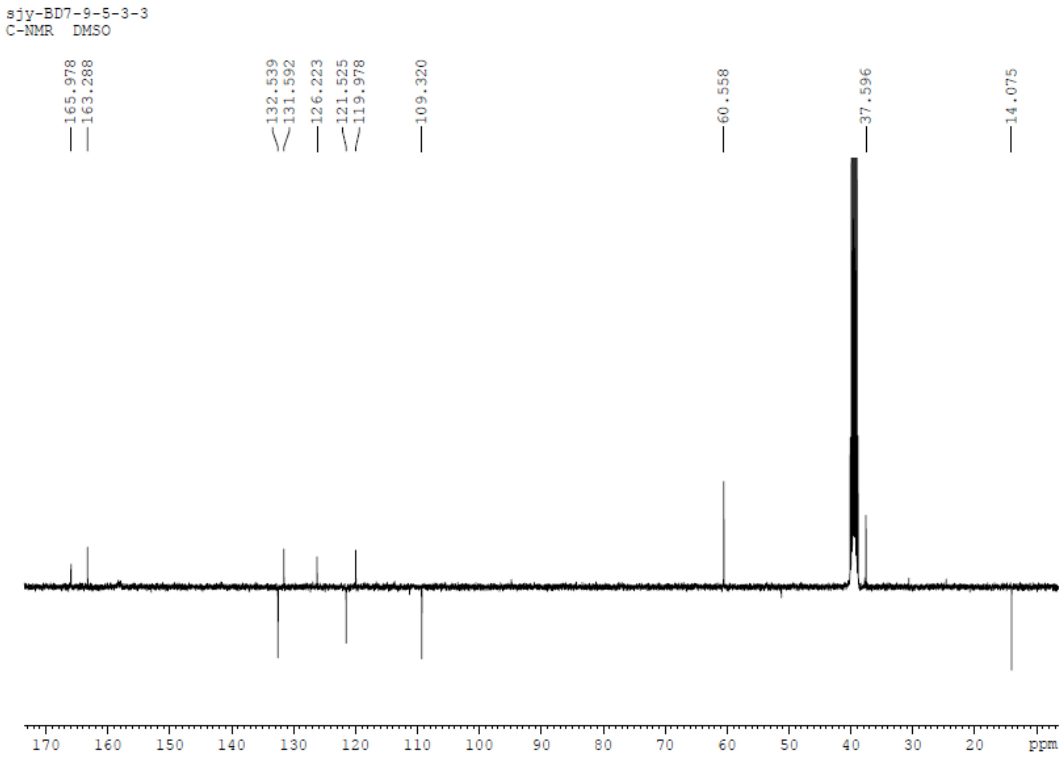

Figure S29. ^{13}C-NMR spectrum of **6** (DMSO-d_6, 100MHz)

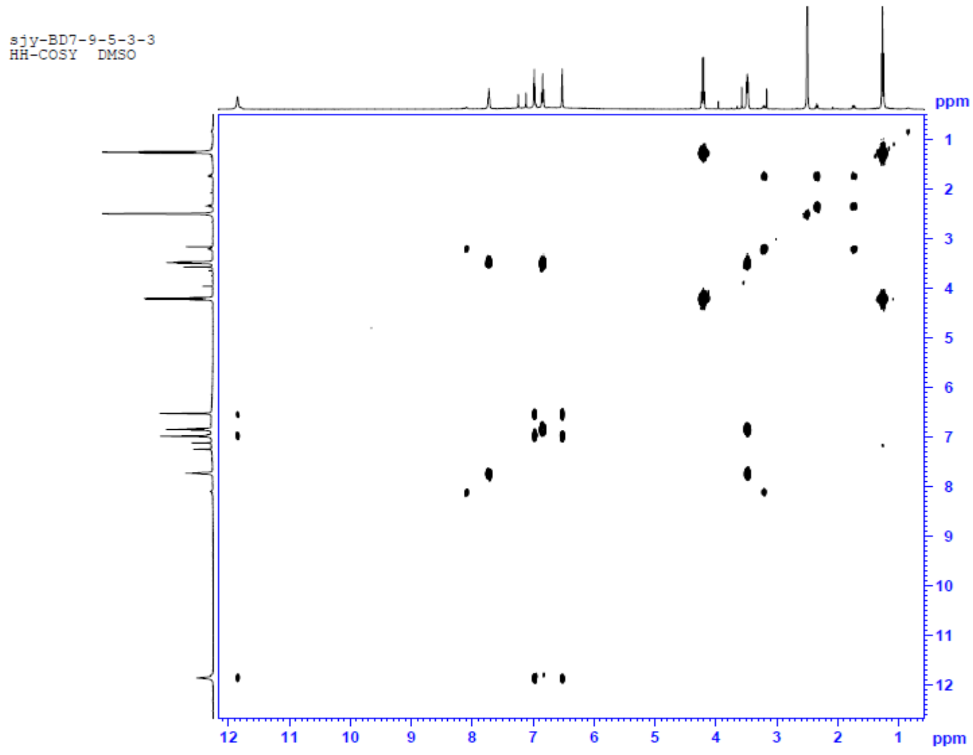

Figure S30. ^{1}H-^{1}H COSY spectrum of **6** (DMSO-d_6, 400MHz)

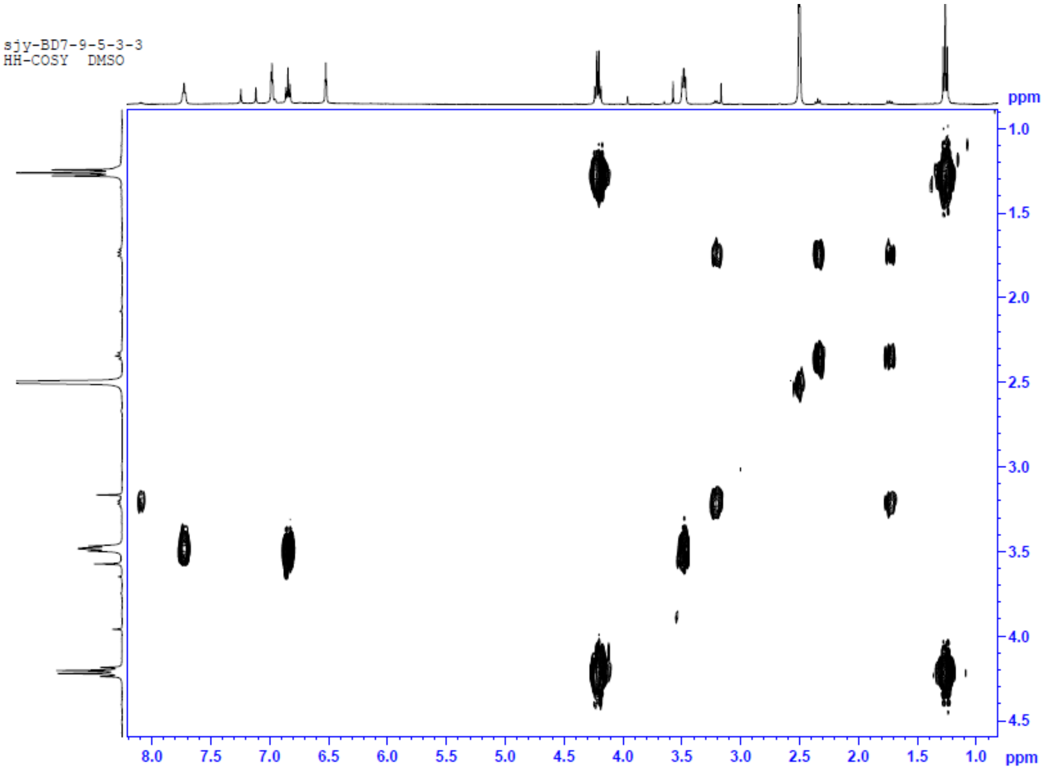

Figure S31. Enlarged ^{1}H-^{1}H COSY spectrum of **6** (DMSO-d_6, 400MHz)

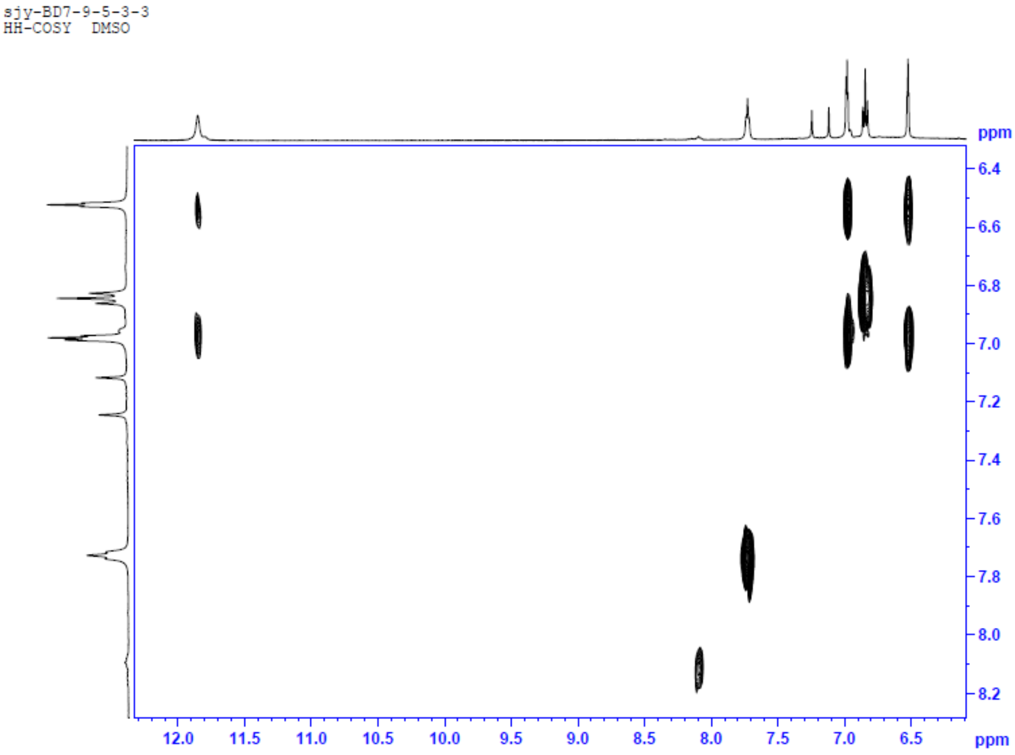

Figure S32. Enlarged ^{1}H-^{1}H COSY spectrum of **6** (DMSO-d_6, 400MHz)

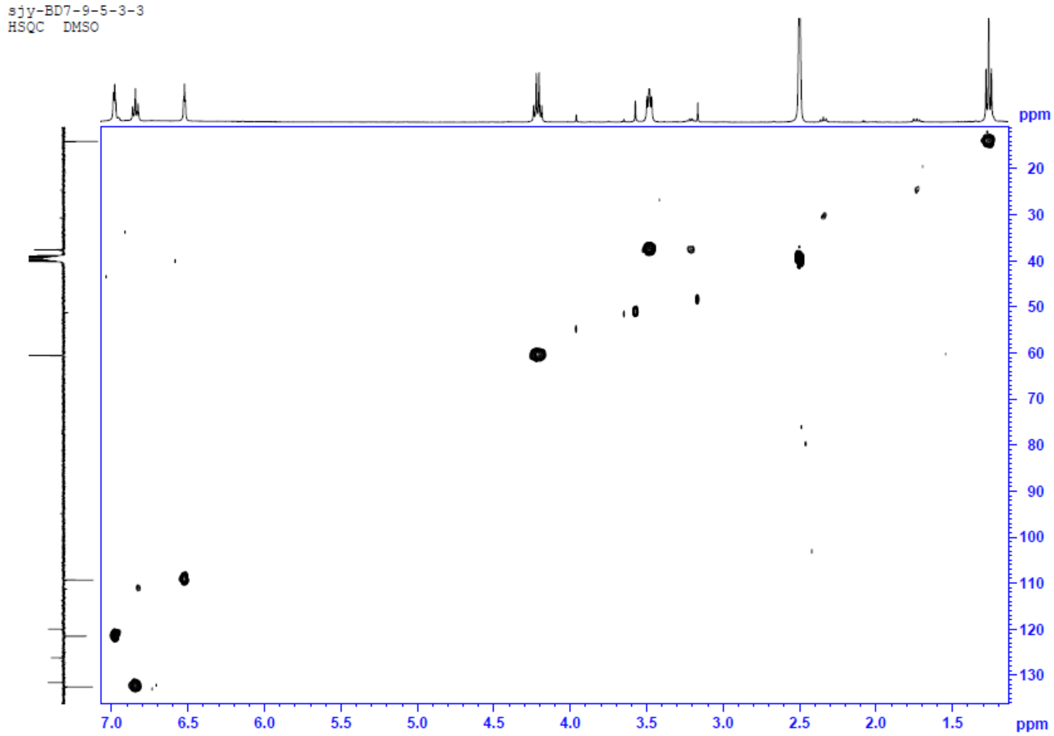

Figure S33. HSQC spectrum of **6** (DMSO-d_6, 400MHz)

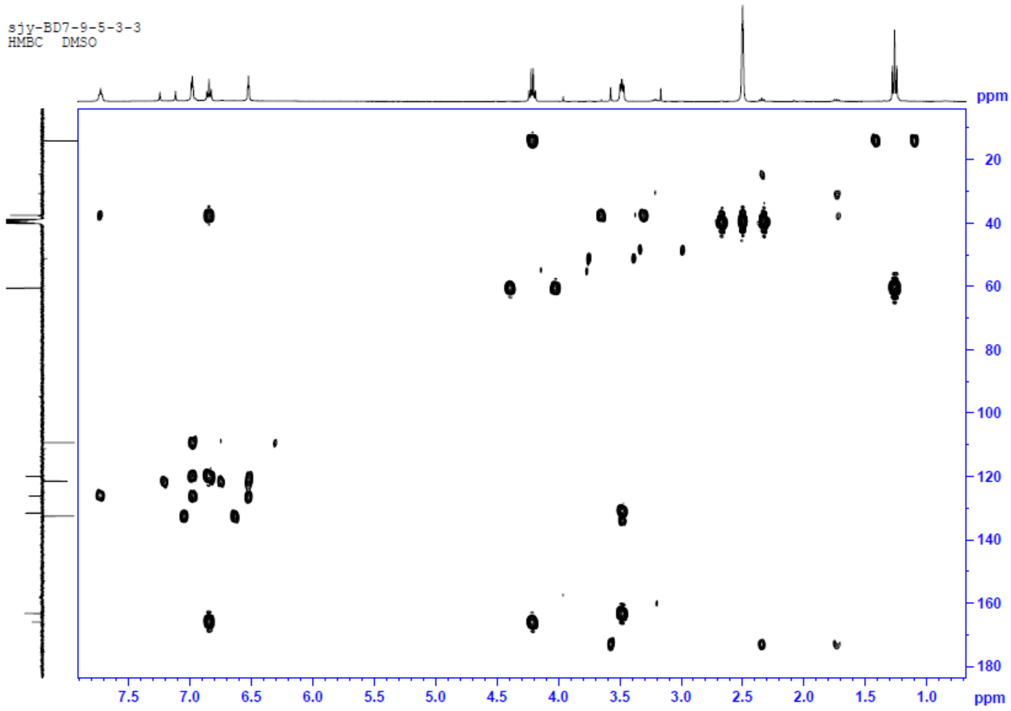

Figure S34. HMBC spectrum of **6** (DMSO-d_6, 400MHz)

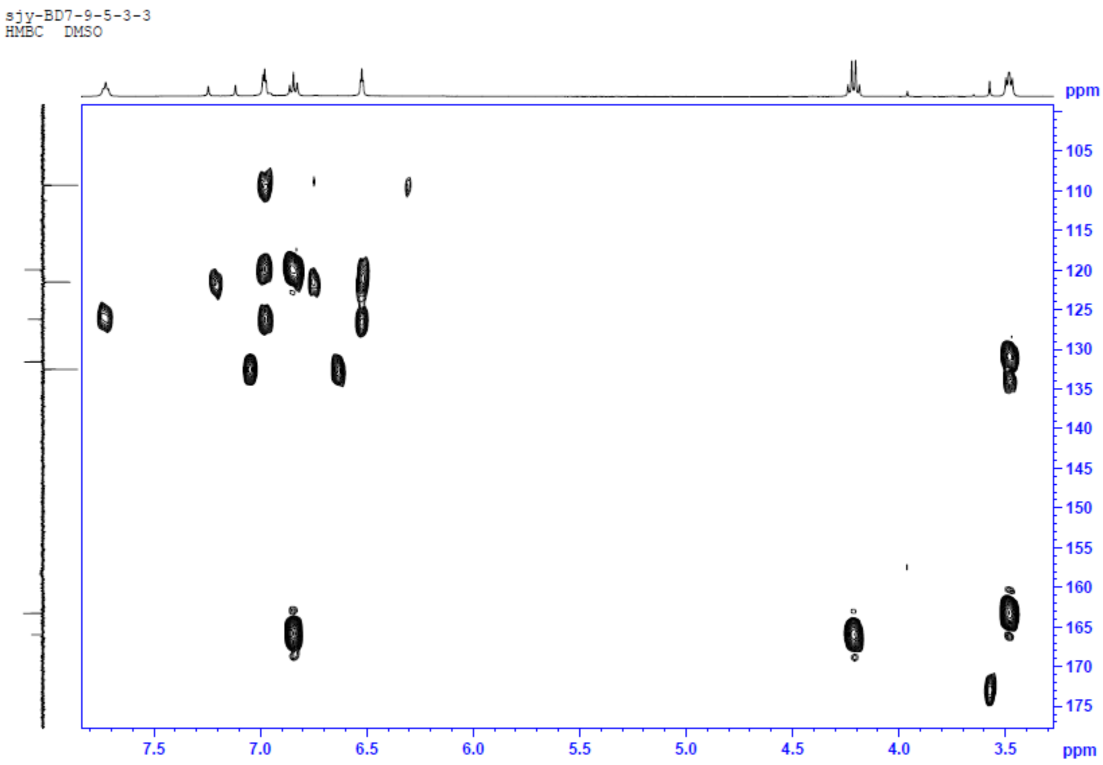

Figure S35. Enlarged HMBC spectrum of **6** (DMSO-d_6, 400MHz)

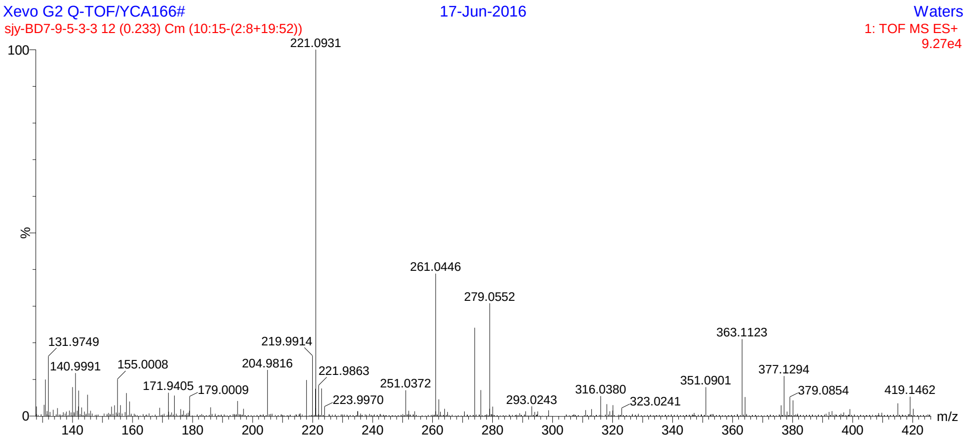

Mass	Calc. Mass	mDa	PPM	DBE	i-FIT	Norm	Conf(%)	Formula
**221.0931**	**221.0926**	**0.5**	**2.3**	**6.5**	**191.7**	**n/a**	**n/a**	$C_{11}H_{13}N_2O_3$

Figure S36. HRESIMS spectrum of **6**

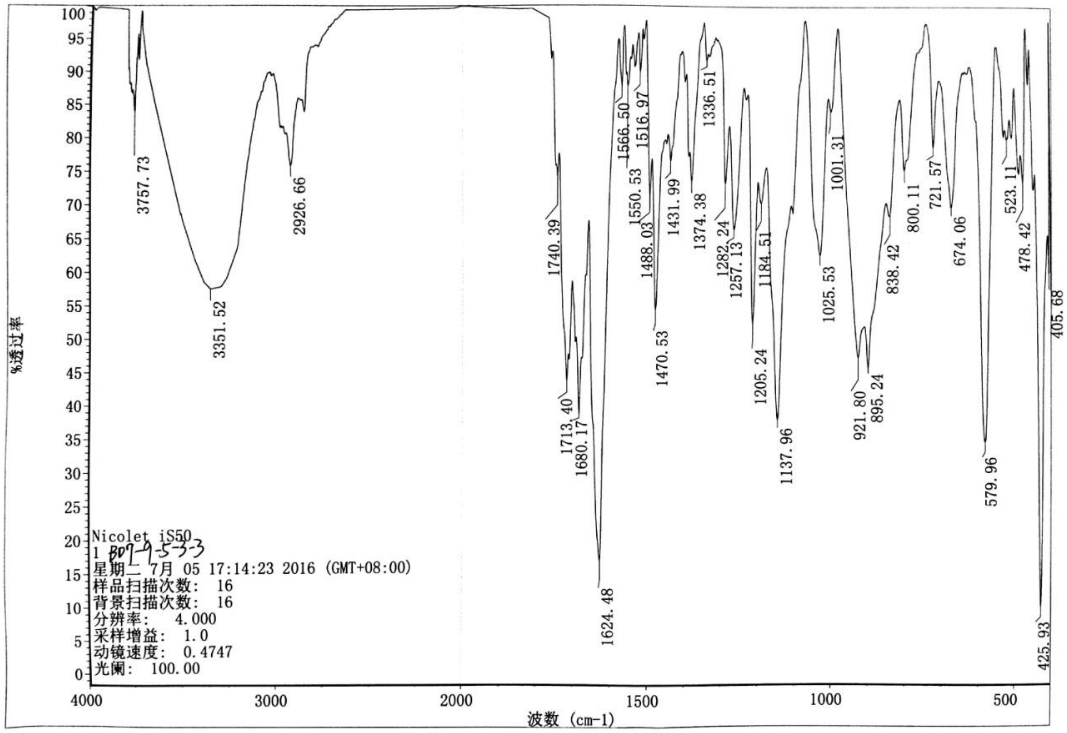

Figure S37. IR spectrum of **6**

The mixture of **2/3** was separated by chiral HPLC (Daciel CHIRALPAK IC column, 0.46cmI.D.*15cmL, *n*-hexane: isopropanol= 78: 22, 1mL/min, 254nm) with ratio of 1:1. [peak a, **2**: $[\alpha]_D^{25}$ +30.80 (c 0.05, MeOH); peak b, **3**: $[\alpha]_D^{25}$-31.20 (c 0.05, MeOH)].

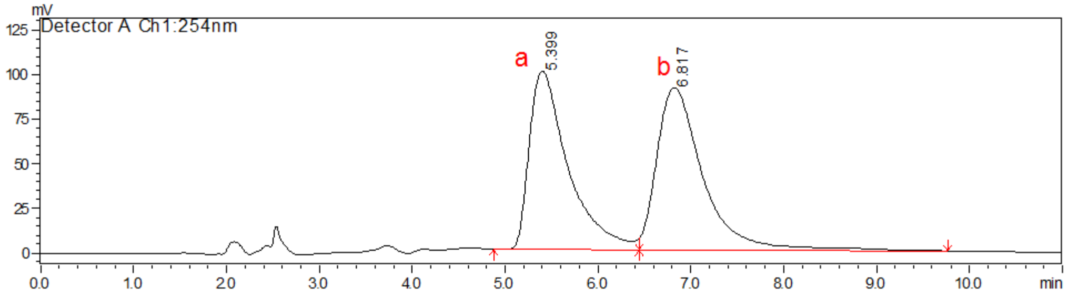

Figure S39. Chiral HPLC separation of **2 (a)** and **3 (b)**

The mixture of **4/5** was separated by chiral HPLC analysis (Daciel CHIRALPAK IC column, 0.46cmI.D.*15cmL, *n*-hexane: ethanol= 75: 25, 1mL/min, 254nm) with ratio of 5:4 [peak a, **4**: $[\alpha]_D^{25}$ -4.00 (c 0.05, MeOH); peak b, **5**: $[\alpha]_D^{25}$+4.00 (c 0.05, MeOH)].

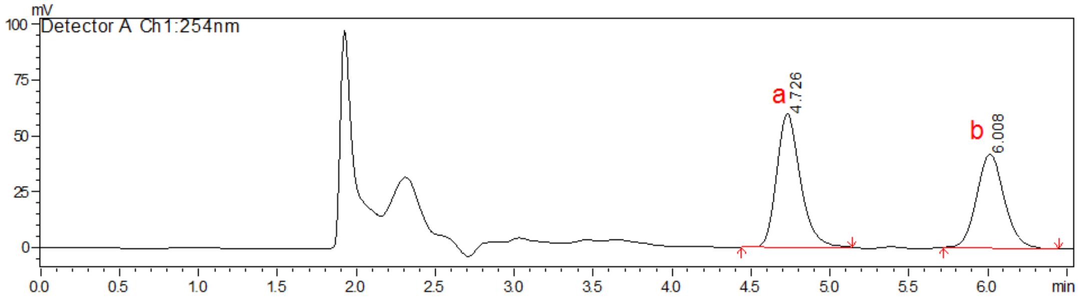

Figure S40. Chiral HPLC separation of **4 (a)** and **5 (b)**

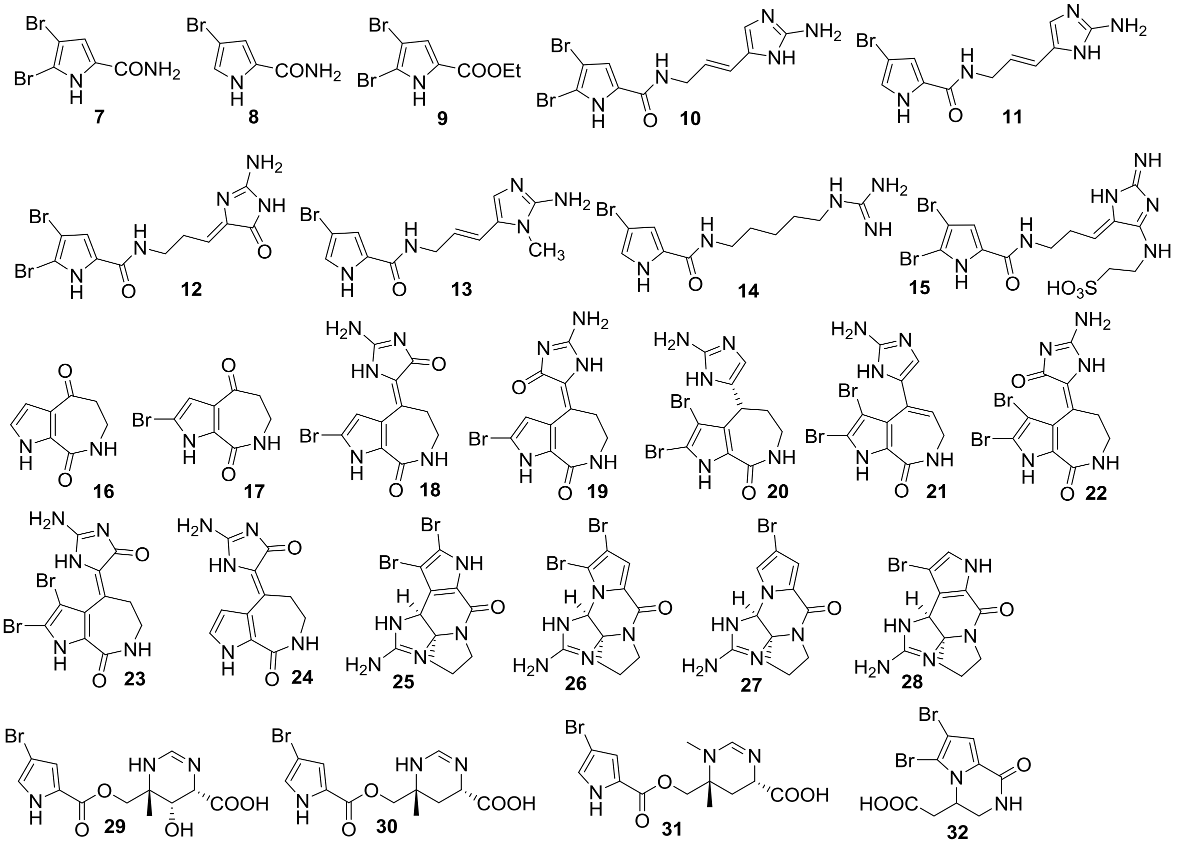

Figure S41. Known alkaloids derived from marine sponge *S. massa*

Physical and spectroscopic data for known compounds

Compound 7 (4,5-dibromopyrrole-2-carbamide): light yellow amorphous; ^{1}H NMR (DMSO-d_6, 400 MHz) δ 12.64 (1H, brs, NH-1), 7.60 (1H, brs, NH), 7.18 (1H, brs, NH), 6.92 (1H, s, H-3) ppm; ^{13}C NMR (DMSO-d_6, 100 MHz) δ 160.4 (C, C-5), 128.2 (C, C-4), 113.1 (CH, C-3), 104.6 (C, C-1), 97.7 (C, C-2) ppm; ESIMS *m/z* 267[M+H]$^+$.

Compound 8 (4-bromopyrrole-2-carbamide): light yellow amorphous; ^{1}H NMR (DMSO-d_6, 400 MHz) δ 11.76 (1H, brs, NH-1), 7.56 (1H, brs, NH), 7.09 (1H, brs, NH), 6.96 (1H, m, H-1), 6.84 (1H, m, H-3) ppm; ^{13}C NMR (DMSO-d_6, 100 MHz) δ 161.1 (C, C-5), 126.9 (C, C-4), 121.2 (CH, C-1), 112.0 (CH, C-3), 94.8 (C, C-2) ppm; ESIMS *m/z* 189[M+H]$^+$.

Compound 9 (ethyl 4,5-dibromopyrrole-2-carboxylate): yellow solid; ^{1}H NMR (DMSO-d_6, 400 MHz) δ 13.11 (1H, s, NH), 6.88 (1H, s, H-3), 4.23 (2H, q, J = 7.00 Hz, H-6), 1.27 (3H, t, J = 7.00 Hz, H-7) ppm; ^{13}C NMR (DMSO-d_6, 100 MHz) δ 158.8 (C, C-5), 124.0 (C, C-4), 117.0 (CH, C-3), 107.4 (C, C-1), 98,9 (C, C-2), 60.2 (CH_2, C-6), 14.2 (CH_3, C-7) ppm; ESIMS *m/z* 296[M+H]$^+$.

Compound 10 (oroidin): yellow amorphous; ^{1}H NMR (DMSO-d_6, 400 MHz) δ 12.86 (1H, brs, NH), 12.72 (1H, brs, NH), 12.20 (1H, brs, NH-1), 8.45 (1H, t, J = 5.66 Hz, NH-2), 7.72 (2H, s, NH_2), 6.98 (1H, d, J = 2.24 Hz, H-3), 6.90 (1H, s, H-10), 6.22 (1H, d, J = 16.1 Hz, H-8), 6.09 (1H, dt, J = 16.1, 5.2 Hz, H-7), 3.96 (2H, m, H-6) ppm; ^{13}C NMR (DMSO-d_6, 100 MHz) δ 158.7 (C, C-5), 147.9 (C, C-11), 128.0 (C, C-4), 126.5 (CH, C-7), 124.9 (C, C-9), 116.3 (CH, C-8), 112.8 (CH, C-3), 111.2 (CH, C-10), 104.7 (C, C-1), 97.9 (C, C-2), 39.9 (CH_2, C-6) ppm; ESIMS *m/z* 388[M+H]$^+$.

Compound 11 (hymenidin): yellow amorphous; ^{1}H NMR (DMSO-d_6, 400 MHz) δ 12.76 (1H, brs, NH), 12.12 (1H, brs, NH), 11.85 (1H, s, NH-1), 8.42 (1H, t, J = 5.7 Hz, NH-2), 7.67 (2H, s, NH_2), 6.98 (1H, m, C-1), 6.91 (1H, s, H-10), 6.89 (1H, m, C-3), 6.22 (1H, d, J = 16.4 Hz, H-8), 6.10 (1H, dt, J = 16.4, 5.4 Hz, H-7), 3.96 (2H, m, H-6) ppm; ^{13}C NMR (DMSO-d_6, 100 MHz) δ 159.4 (C, C-5), 147.8 (C, C-11), 126.8 (CH, C-7), 126.7 (C, C-4), 124.9 (C, C-9), 121.3 (CH, C-1), 116.2 (CH, C-8), 111.6 (CH, C-3), 111.1 (CH, C-10), 94.9 (C, C-2), 39.8 (CH_2, C-6) ppm; ESIMS *m/z* 310[M+H]$^+$.

Compound 12 (dispacamide 1): light yellow amorphous; ^{1}H NMR (DMSO-d_6, 400

MHz) δ 12.70 (1H, s, NH-1), 9.57 (2H, brs, NH_2), 8.30 (1H, t, J = 5.7 Hz, NH-2), 6.90 (1H, d, J = 2.3 Hz, H-3), 5.99 (1H, t, J = 7.6 Hz, H-8), 3.38 (2H, m, H-6), 2.52 (2H, m, H-7) ppm; ^{13}C NMR (DMSO-d_6, 100 MHz) δ 162.9 (C, C-10), 158.9 (C, C-5), 155.7 (C, C-11), 129.2 (C, C-9), 128.0 (C, C-4), 117.1 (CH, C-8), 112.6 (CH, C-3), 104.6 (C, C-1), 97.8 (C, C-2), 37.3 (CH_2, C-6), 27.3 (CH_2, C-7) ppm; ESIMS *m/z* 404$[M+H]^+$.

Compound 13 (keramadine): light yellow amorphous; ^{1}H NMR(DMSO-d_6, 400 MHz) δ 12.69 (1H, s, NH-3), 11.85 (1H, s, NH-1), 8.46 (1H, t, J = 5.5 Hz, NH-2), 7.80 (1H, s, NH-4), 7.11 (1H, s, H-10), 6.98 (1H, m, C-1), 6.84 (1H, m, C-3), 6.25 (1H, d, J = 11.7 Hz, H-8), 5.85 (1H, m, H-7), 4.01 (2H, m, H-6), 3.39 (3H, s, CH_3) ppm; ^{13}C NMR (DMSO-d_6, 100 MHz) δ 159.5 (C, C-5), 146.6 (C, C-11), 133.3 (CH, C-7), 126.6 (C, C-4), 123.7 (C, C-9), 121.3 (CH, C-1), 113.7 (CH, C-8), 111.9 (CH, C-10), 111.5 (CH, C-3), 94.9 (C, C-2), 37.7 (CH_2, H-6), 29.2 (CH_3) ppm; ESIMS *m/z* 324$[M+H]^+$.

Compound 14 (laughine): light yellow amorphous; ^{1}H NMR (DMSO-d_6, 400 MHz) δ 11.77 (1H, s, NH-1), 8.08 (1H, t, J = 5.6 Hz, NH-3), 7.80 (2H, s, NH_2), 7.66 (1H, t, J = 5.3 Hz, NH-2), 6.95 (1H, m, H-1), 6.82 (1H, m, H-3), 3.19 (2H, m, H-10), 3.08 (2H, m, H-6), 1.49 (2H, m, H-7), 1.48 (2H, m, H-9), 1.30 (2H, m, H-8) ppm; ^{13}C NMR (DMSO-d_6, 100 MHz) δ 159.4 (C, C-11), 156.7 (C, C-5), 127.0 (C, C-4), 120.9 (CH, C-1), 111.2 (CH, C-3), 94.8 (C, C-2), 40.6 (CH_2, C-6), 38.2 (CH_2, C-10), 28.8 (CH_2, C-7), 28.1 (CH_2, C-9), 23.4 (CH_2, C-8) ppm； ESIMS *m/z* 316$[M+H]^+$.

Compound 15 (taurodispacamide A): light yellow amorphous; ^{1}H NMR (DMSO-d_6, 400 MHz) δ 12.72 (1H, s, NH-1), 11.27 (1H, s, NH-5), 9.60 (1H, t, NH-6), 9.20 (1H, s, NH), 8.48 (1H, s, NH), 8.27 (1H, t, NH-2), 6.92 (1H, s, H-3), 6.14 (1H, t, J = 7.5 Hz, H-8), 3.67 (2H, m, H-12), 3.35 (2H, m, H-6), 2.78 (2H, m, H-13), 2.53 (2H, m, H-7) ppm; ^{13}C NMR (DMSO-d_6, 100 MHz) δ 167.1 (C, C-11), 166.0 (C, C-10), 159.0 (C, C-5), 132.8 (C, C-9), 128.0 (C, C-4), 114.3 (CH, C-8), 112.6 (CH, C-3), 104.6 (C, C-1), 97.8 (C, C-2), 49.2 (CH_2, C-13), 39.9 (CH_2, C-12), 37.7 (CH_2, C-6), 27.8 (CH_2, C-7) ppm; ESIMS *m/z* 511$[M+H]^+$.

Compound 16 (aldizine): yellow solid; ^{1}H NMR (DMSO-d_6, 400 MHz) δ 12.16 (1H, brs, NH-1), 8.32 (1H, t, J = 5.1 Hz, NH-2), 6.98 (1H, t, J = 2.7 Hz, H-1), 6.55 (1H, t, J = 2.7 Hz, H-2), 3.35 (2H, m, H-6), 2.70 (2H, m, H-7) ppm; ^{13}C NMR (DMSO-d_6, 100 MHz) δ 194.4 (C, C-8), 162.2 (C, C-5), 127.9 (C, C-4), 123.6 (C, C-3), 122.4 (CH, C-1), 109.5

(CH, C-2), 43.5 (CH_2, C-7), 36.6 (CH_2, C-6) ppm; ESIMS *m/z* 265$[M+H]^+$.

Compound 17 (2-bromoaldizine): light yellow single crystal; ^{1}H NMR (DMSO-d_6, 400 MHz) δ 12.97 (1H, brs, NH-1), 8.40 (1H, t, J = 5.1 Hz, NH), 6.56 (1H, s, H-2), 3.34 (2H, m, H-6), 2.70 (2H, m, H-7) ppm; ^{13}C NMR (DMSO-d_6, 100 MHz) δ 193.5 (C, C-8), 161.3 (C, C-5), 129.4 (C, C-4), 124.6 (C, C-3), 111.2 (CH, C-2), 105.2 (C, C-1), 43.4 (CH_2, C-7), 36.3 (CH_2, C-6) ppm; ESIMS *m/z* 243$[M+H]^+$.

Compound 18 (10Z-hymenialdisine): light yellow single crystal; ^{1}H NMR (DMSO-d_6, 400 MHz) δ 12.84 (1H, brs, NH-1), 8.92 (2H, brs, NH_2), 8.09 (1H, s, NH), 6.59 (1H, s, H-2), 3.27 (2H, m, H-7) , 3.26 (2H, m, H-6) ppm; ^{13}C NMR (DMSO-d_6, 100 MHz) δ 164.3 (C, C-10), 162.3 (C, C-5), 155.1 (C, C-11), 128.1 (C, C-4), 127.9 (C, C-8), 121.9 (C, C-3), 111.4 (CH, C-2), 104.8 (C, C-1), 39.1 (CH_2, C-6), 31.9 (CH_2, C-7) ppm； ESIMS *m/z* 324$[M+H]^+$.

Compound 19 (10E-hymenialdisine): light yellow amorphous; ^{1}H NMR (DMSO-d_6, 400 MHz) δ 12.63 (1H, brs, NH-1), 9.26 (2H, brs, NH_2), 8.03 (1H, t, J = 5.1 Hz, NH-2), 6.73 (1H, d, J = 2.1 Hz, H-2), 3.26 (2H, m, H-6) , 2.83 (2H, m, H-7) ppm; ^{13}C NMR (DMSO-d_6, 100 MHz) δ 163.0 (C, C-10), 161.3 (C, C-5), 153.7 (C, C-11), 128.2 (C, C-4), 127.6 (C, C-8), 123.3 (C, C-9), 119.9 (C-3), 113.9 (CH, C-2), 102.2 (C, C-1), 38.0 (CH_2, C-6), 36.6 (CH_2, C-7) ppm； ESIMS *m/z* 324$[M+H]^+$.

Compound 20 ((-)-hymenin): light yellow amorphous; $[\alpha]_D^{25}$ -8.65 (*c* 1.62, MeOH); ^{1}H NMR (DMSO-d_6, 400 MHz) δ 12.62 (1H, s, NH-1), 12.39 (1H, s, NH), 11.94 (1H, s, NH), 7.96 (1H, m, NH-2), 7.45 (2H, s, NH_2), 6.23 (1H, s, H-10), 4.09 (1H, t, J = 3.9 Hz, H-8), 3.10 (2H, m, H-6), 2.15 (1H, m, H-7), 2.00 (1H, m, H-7) ppm; ^{13}C NMR (DMSO-d_6, 100 MHz) δ 161.1 (C, C-5), 147.1 (C, C-11), 128.9 (C, C-4), 125.2 (C, C-9), 123.2 (C, C-3), 111.1 (CH, C-10), 106.2 (C, C-1), 100.6 (C, C-2), 36.4 (CH_2, C-6), 34.2 (CH, C-8), 31.8 (CH_2, C-7) ppm； ESIMS *m/z* 388$[M+H]^+$.

Compound 21 (stevensine): light yellow amorphous; ^{1}H NMR (DMSO-d_6, 400 MHz) δ 13.29 (1H, s, NH-1), 12.44 (1H, brs, NH), 12.24 (1H, brs, NH), 8.12 (1H, t, J = 5.0 Hz, NH-2), 7.49 (2H, s, NH_2), 6.89 (1H, s, H-10), 6.20 (1H, t, J = 7.2 Hz, H-7), 3.44 (2H, m, H-6) ppm; ^{13}C NMR (DMSO-d_6, 100 MHz) δ 161.5 (C, C-5), 147.2 (C, C-11), 128.6 (C, C-4), 126.0 (CH, C-7), 125.9 (C, C-8), 124.7 (C, C-9), 120.9 (C, C-3), 111.7 (CH, C-10), 107.6 (C, C-1), 97.7 (C, C-2), 37.1 (CH_2, C-6) ppm； ESIMS *m/z* 386$[M+H]^+$.

Compound 22 ((10E)-3-bromohymenialdisine): light yellow amorphous; ^{1}H NMR (DMSO-d_6, 400 MHz) δ 13.16 (1H, s, NH-1), 9.36 (2H, brs, NH_2), 8.03 (1H, t, J = 5.2, 5.6 Hz, NH-2), 3.25 (2H, m, H-6), 2.85 (2H, m, H-7) ppm; ^{13}C NMR (DMSO-d_6, 100 MHz) δ 163.8 (C, C-10), 161.9 (C, C-5), 153.7 (C, C-11), 126.85.2 (C, C-4), 123.3 (C, C-8), 121.9 (C, C-9), 118.4 (C, C-3), 105.8 (C, C-1), 101.9 (C, C-2), 37.8 (CH_2, C-6) , 37.7 (CH_2, C-7) ppm； ESIMS m/z 402[M+H]$^+$.

Compound 23 ((10Z)-3-bromohymenialdisine): light yellow amorphous; ^{1}H NMR (DMSO-d_6, 400 MHz) δ 13.40 (1H, NH-1), 11.23 (1H, brs, NH), 9.53 (1H, brs, NH_2), 8.90 (1H, brs, NH_2), 8.07 (1H, t, J = 4.9 Hz, NH-2), 3.27 (2H, m, H-6) ppm; ^{13}C NMR (DMSO-d_6, 100 MHz) δ 163.3 (C, C-10), 162.8 (C, C-5), 154.4 (C, C-11), 127.2 (C-4), 125.7 (C-8), 124.0 (C-9), 120.8 (C-3), 107.4 (C, C-1), 98.6 (C, C-2), 38.7 (CH_2, C-6) , 35.2 (CH_2, C-7) ppm； ESIMS m/z 402[M+H]$^+$.

Compound 24 ((10Z)-debromohymenialdisine): light yellow amorphous; ^{1}H NMR (DMSO-d_6, 400 MHz) δ 12.06 (1H, NH-1), 8.03 (1H, t, J = 4.7 Hz, NH-2), 7.09 (1H, t, J = 2.7 Hz, H-1), 6.58 (1H, t, J = 2.2 Hz, H-2), 3.3 (2H, m, C-7) , 3.26 (2H, m, C-6) ppm; ^{13}C NMR (DMSO-d_6, 100 MHz) δ 163.0 (C, C-5), 155.3 (C, C-11), 129.2 (C, C-4), 126.5 (C, C-8), 122.6 (CH, C-1), 120.6 (C, C-3), 109.7 (CH, C-2), 39.4 (CH_2, C-6) , 31.3 (CH_2, C-7) ppm； ESIMS m/z 402[M+H]$^+$.

Compound 25 ((-)-dibromoisophakellin): light yellow amorphous; $[\alpha]_D^{25}$-59.1 (c 1.10 , MeOH); ^{1}H NMR (DMSO-d_6, 400 MHz) δ 13.34 (1H, s, NH-1), 9.79 (1H, s, NH), 8.84 (1H, s, NH), 7.96 (2H, brs, NH_2), 5.22 (1H, s, H-10), 3.55 (1H, m, H-6), 3.45 (1H, m, H-6), 2.21 (2H, m, H-8), 2.00 (2H, m, H-7) ppm; ^{13}C NMR (DMSO-d_6, 100 MHz) δ 156.8 (C, C-11), 154.7 (C, C-5), 122.8 (C, C-4), 122.4 (C, C-3), 108.4 (C, C-1), 96.3 (C, C-2), 84.1 (C, C-9), 54.0 (CH, C-10), 44.1 (CH_2, C-6), 39.1 (CH_2, C-8), 19.1 (CH_2, C-7) ppm； ESIMS m/z 388[M+H]$^+$.

Compound 26 ((-)-dibromophakellin): light yellow amorphous; $[\alpha]_D^{25}$-94.2 (c 1.54 , MeOH); ^{1}H NMR (DMSO-d_6, 400 MHz) δ 10.23 (1H, s, NH_2), 9.75 (1H, s, NH_2), 8.56 (1H, brs, NH), 8.19 (1H, brs, NH), 7.03 (H, s, H-3), 6.30 (1H, s, H-10), 3.67 (1H, m, H-6), 3.48 (1H, m, H-6), 2.40 (1H, m, H-8), 2.28 (1H, m, H-8) , 2.05 (2H, m, H-7) ppm; ^{13}C NMR (DMSO-d_6, 100 MHz) δ 156.4 (C, C-11), 153.7 (C, C-5), 125.0 (C, C-4), 114.8 (CH, C-3), 106.1 (C, C-1), 102.0 (C, C-2), 82.4 (C, C-9), 68.2 (CH, C-10), 44.7 (CH_2, C-6), 38.5

(CH_2, C-8), 19.0 (CH_2, C-7) ppm；ESIMS *m/z* 388$[M+H]^+$.

Compound 27 ((-)-monobromophakellin): light yellow amorphous; $[\alpha]_D^{25}$-63.1 (*c* 2.56 , MeOH); ^{1}H NMR (DMSO-d_6, 400 MHz) δ 10.26 (1H, brs, NH_2), 10.08 (1H, brs, NH_2), 8.65 (1H, s, NH), 7.29 (1H, d, *J* = 1.7 Hz, H-1), 6.80 (H, d, *J* = 1.7 Hz, H-3), 6.10 (1H, s, H-10), 3.64 (1H, m, H-6), 3.50 (1H, m, H-6), 2.35 (1H, m, H-8), 2.24 (1H, m, H-8), 2.06 (2H, m, H-7) ppm; ^{13}C NMR (DMSO-d_6, 100 MHz) δ 156.7 (C, C-11), 154.2 (C, C-5), 123.9 (C, C-4), 121.7 (CH, C-1), 113.3 (CH, C-3), 98.1 (C, C-2), 82.1 (C, C-9), 68.1 (CH, C-10), 45.0 (CH_2, C-6), 38.1 (CH_2, C-8), 19.3 (CH_2, C-7) ppm；ESIMS *m/z* 310$[M+H]^+$.

Compound 28 ((-)-monobromoisophakellin): light yellow amorphous; $[\alpha]_D^{25}$-31.0 (*c* 0.48 , MeOH); ^{1}H NMR (DMSO-d_6, 400 MHz) δ 12.46 (1H, s, NH-1), 9.67 (1H, s, NH_2), 8.68 (1H, s, NH_2), 7.86 (1H, brs, NH), 7.23 (1H, d, *J* = 2.8 Hz, H-1), 5.23 (1H, s, H-10), 3.58 (1H, m, H-6), 3.46 (1H, m, H-6), 2.22 (2H, m, H-8), 2.01 (2H, m, H-7) ppm; ^{13}C NMR (DMSO-d_6, 100 MHz) δ 156.7 (C, C-11), 155.5 (C, C-5), 124.4 (CH, C-1), 121.6 (C, C-4), 121.4 (C, C-3), 93.2 (C, C-2), 84.2 (C, C-9), 54.1 (CH, C-10), 48.3 (CH_2, C-6), 44.0 (CH_2, C-8), 19.2 (CH_2, C-7) ppm；ESIMS *m/z* 310$[M+H]^+$.

Compound 29 ((-)-manzacidins B): light yellow amorphous; $[\alpha]_D^{25}$-38.2 (*c* 2.32, MeOH); ^{1}H NMR (DMSO-d_6, 400 MHz) δ 12.47 (1H, brs, NH-1), 10.22 (1H, brs, NH), 8.05 (1H, s, H-10), 7.25 (1H, s, H-1), 6.99 (1H, s, H-3), 4.30 (1H, m, H-6), 4.28 (1H, m, H-8), 4.21 (1H, m, H-6), 4.18 (1H, m, H-9), 1.32 (3H, s, CH_3) ppm; ^{13}C NMR (DMSO-d_6, 100 MHz) δ 168.5 (C, COOH), 158.9 (C, C-5), 149.9 (CH, C-10), 124.2 (CH, C-1), 122.3 (C, C-4), 117.0 (CH, C-3), 96.1 (C, C-2), 65.4 (CH_2, C-6), 63.8 (CH, C-8), 56.1 (C, C-7), 54.1 (CH, C-9), 23.2 (CH_3) ppm；ESIMS *m/z* 360$[M+H]^+$.

Compound 30 (manzacidins C): light yellow amorphous; $[\alpha]_D^{25}$+ 27.3 (*c* 3.26, MeOH); ^{1}H NMR (DMSO-d_6, 400 MHz) δ 12.63 (1H, brs, NH-1), 10.59 (1H, brs, NH), 8.11 (1H, s, H-10), 7.22 (1H, s, H-1), 6.95 (1H, s, H-3), 4.33 (1H, d, *J* = 11.0 Hz, H-6), 4.08 (1H, d, *J* = 11.0 Hz, H-6), 4.01 (1H, m, H-9), 2.22 (1H, dd, *J* = 13.7, 4.1 Hz, H-8), 1.88 (1H, m, H-8), 1.31 (3H, s, CH_3) ppm; ^{13}C NMR (DMSO-d_6, 100 MHz) δ 170.5 (C, COOH), 158.8 (C, C-5), 150.0 (CH, C-10), 124.3 (CH, C-1), 122.1 (C, C-4), 117.0 (CH, C-3), 96.1 (C, C-2), 68.1 (CH_2, C-6), 52.1 (C, C-7), 49.0 (CH, C-9), 30.4 (CH_2, C-8), 23.5 (CH_3) ppm; ESIMS *m/z* 344$[M+H]^+$.

Compound 31 (N-methylmanzacidin C): light yellow single crystal; Mp: 200.5-201.5 ^{o}C; molecular formula $C_{13}H_{16}O_4N_3Br$.

Compound 32 (longamide B): light yellow amorphous; $[\alpha]_D^{25}$ 0 (*c* 0.05, MeOH); ^{1}H NMR (DMSO-d_6, 400 MHz) δ 7.84 (1H, NH), 6.85 (1H, s, H-3), 4.66 (1H, d, J = 10.5 Hz, H-7), 3.82 (1H, m, H-6), 3.39 (1H, m, H-6), 2.73 (2H, m, H-8), 2.46 (2H, m, H-8) ppm; ^{13}C NMR (DMSO-d_6, 100 MHz) δ 170.9 (C, C-9), 157.5 (C, C-5), 125.7 (C, C-4), 113.9 (CH, C-3), 105.8 (C, C-1), 99.4 (C, C-2), 50.2 (CH, C-7), 42.1 (CH_2, C-6), 35.7 (CH_2, C-8) ppm； ESIMS *m/z* 351$[M+H]^+$.

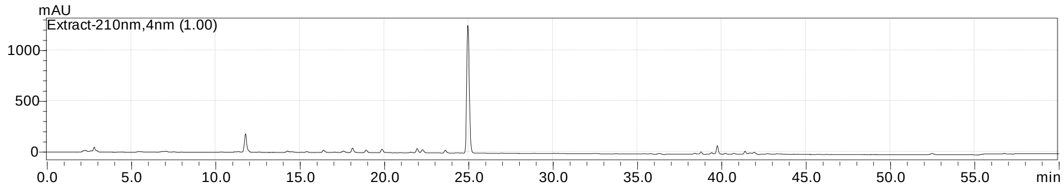

The DAD-HPLC of **7** (0-40min, 5%-100%MeOH-H_2O)

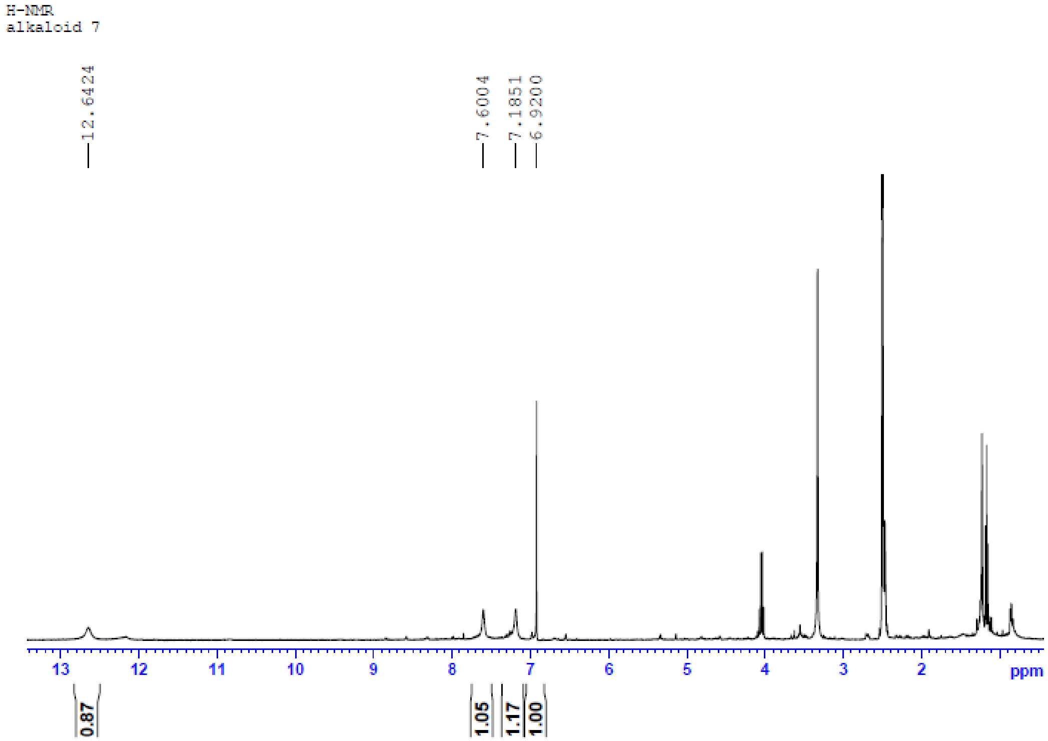

^{1}H-NMR spectrum of **7** (DMSO-d_6, 400MHz)

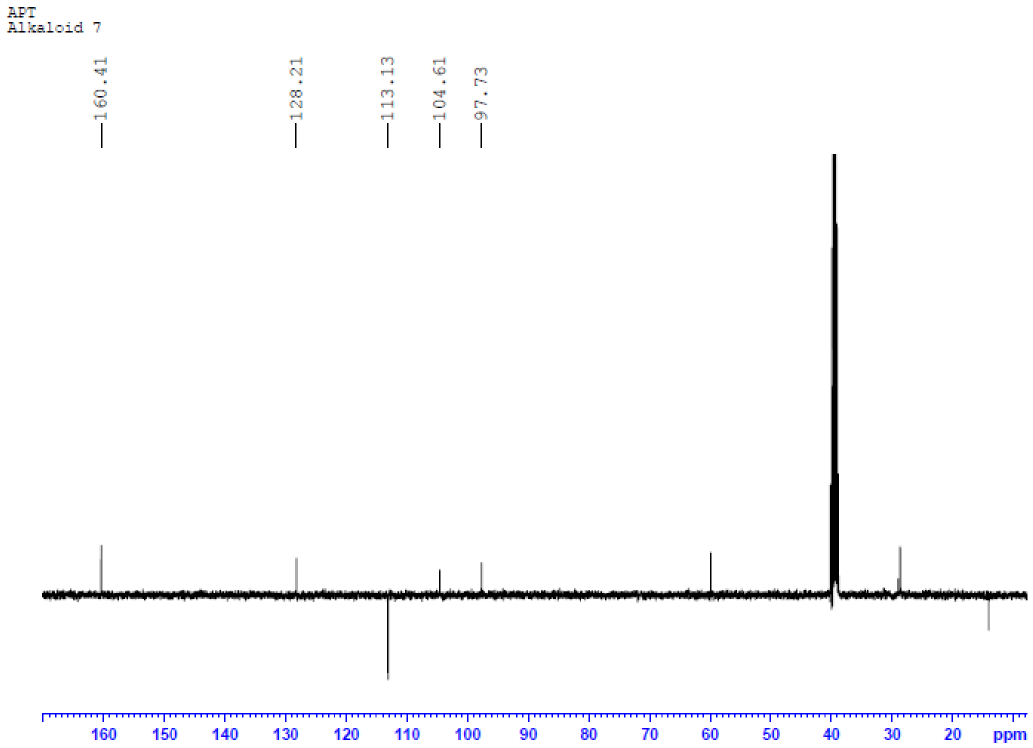

^{13}C-NMR spectrum of **7** (DMSO-d_6, 100MHz)

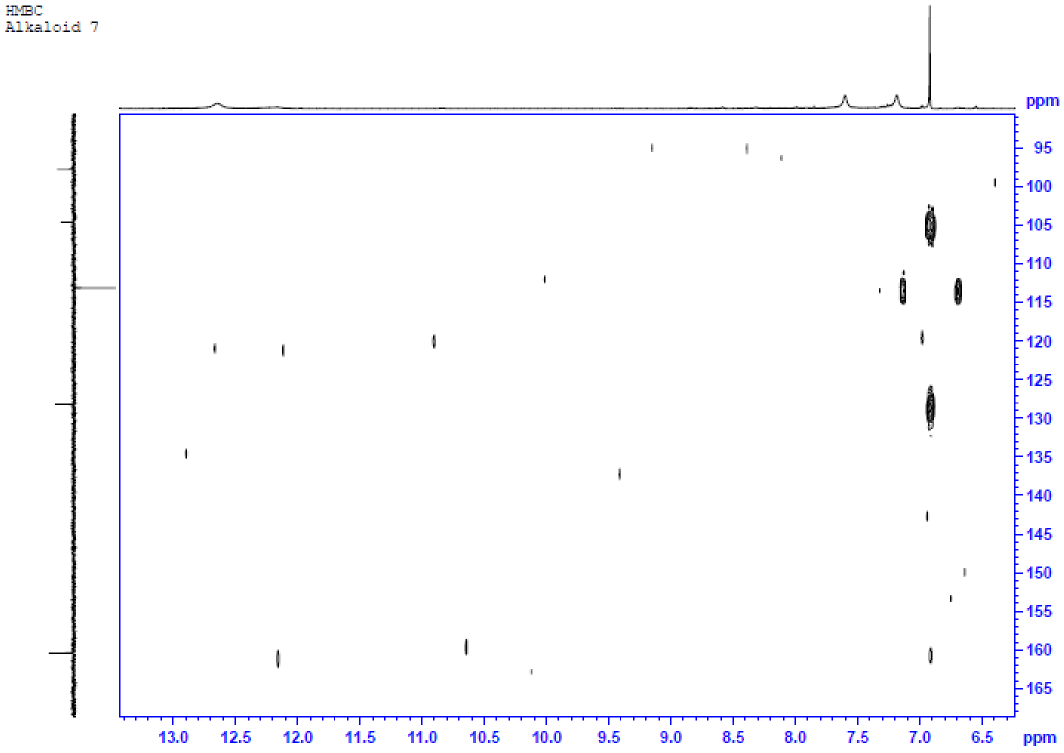

HMBC spectrum of **7** (DMSO-d_6, 400MHz)

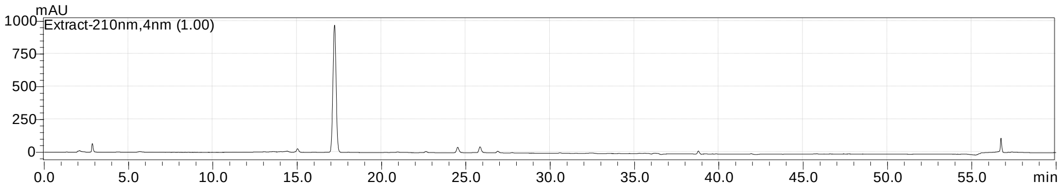

The DAD-HPLC of **8** (0-40min, 5%-100%MeOH-H_2O)

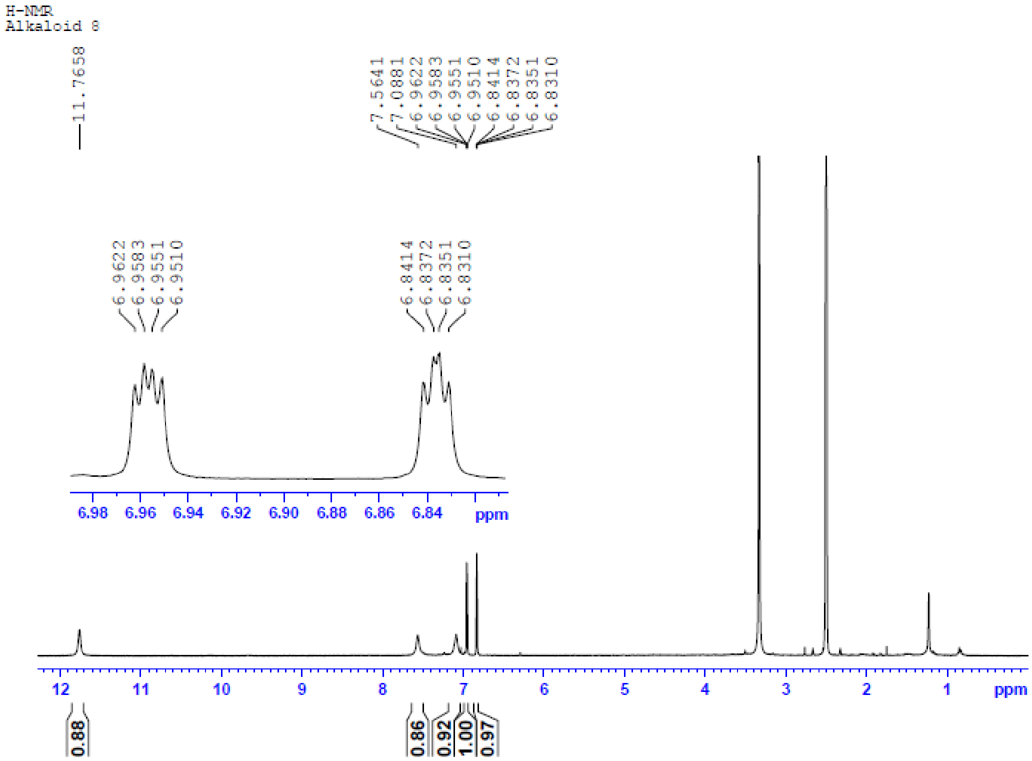

^{1}H-NMR spectrum of **8** (DMSO-d_6, 400MHz)

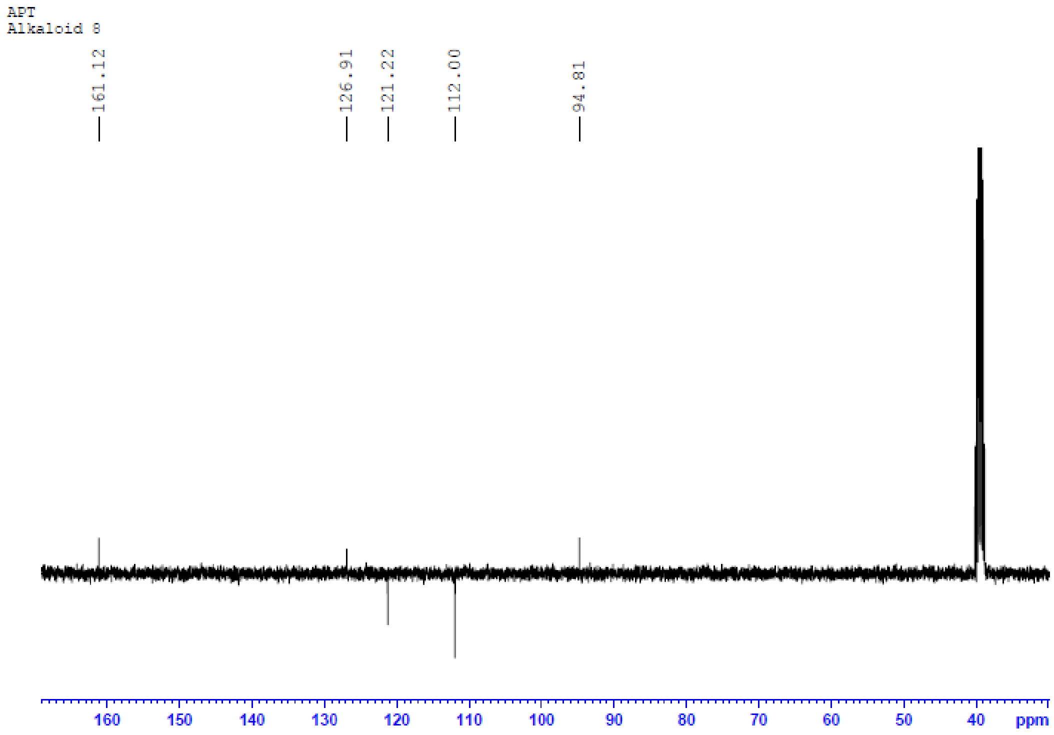

^{13}C-NMR spectrum of **8** (DMSO-d_6, 100MHz)

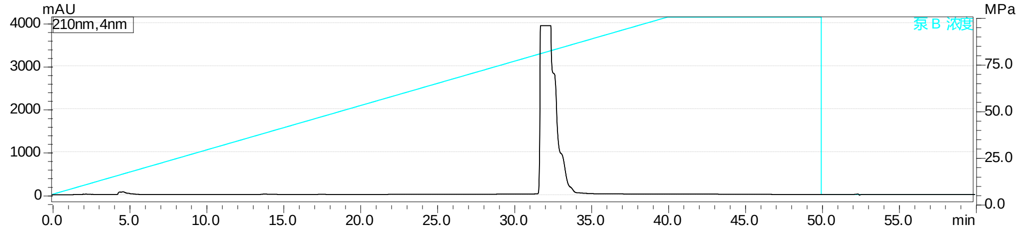

The DAD-HPLC of **9** (0-40min, 5%-100%MeOH-H_2O)

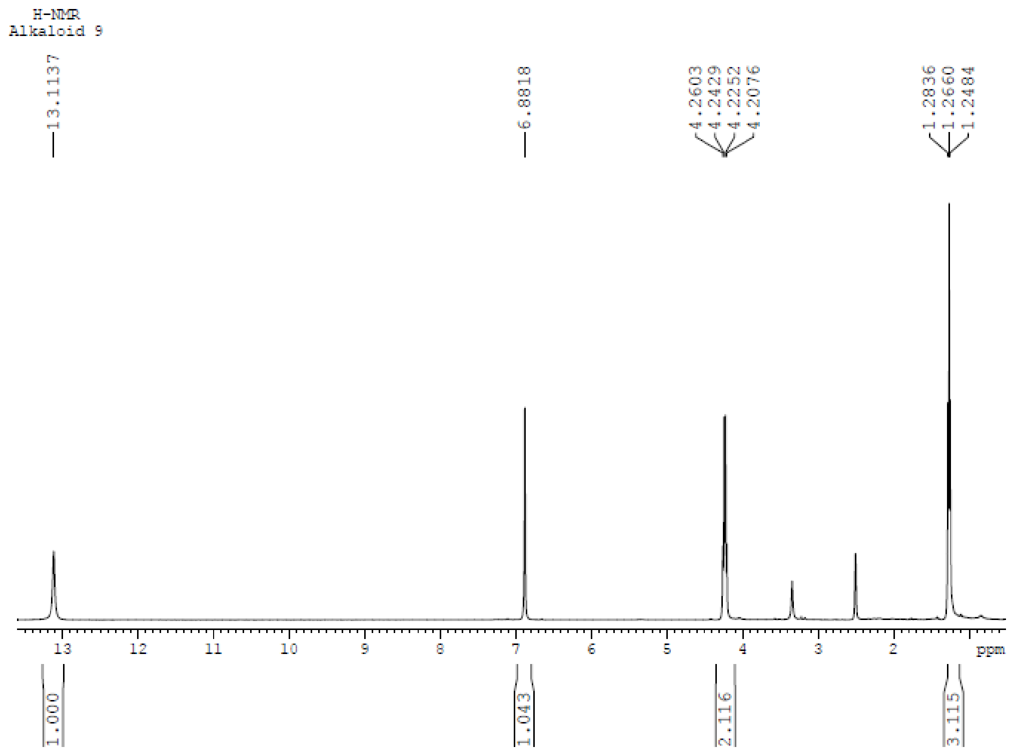

^{1}H-NMR spectrum of **9** (DMSO-d_6, 400MHz)

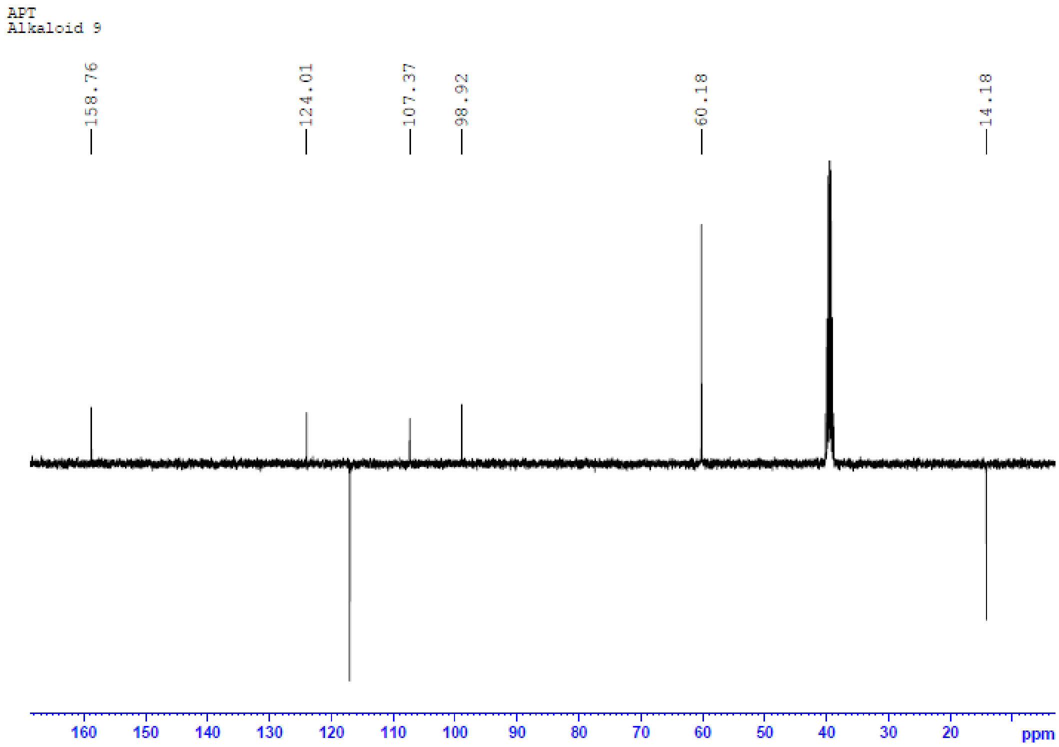

^{13}C-NMR spectrum of **9** (DMSO-d_6, 100MHz)

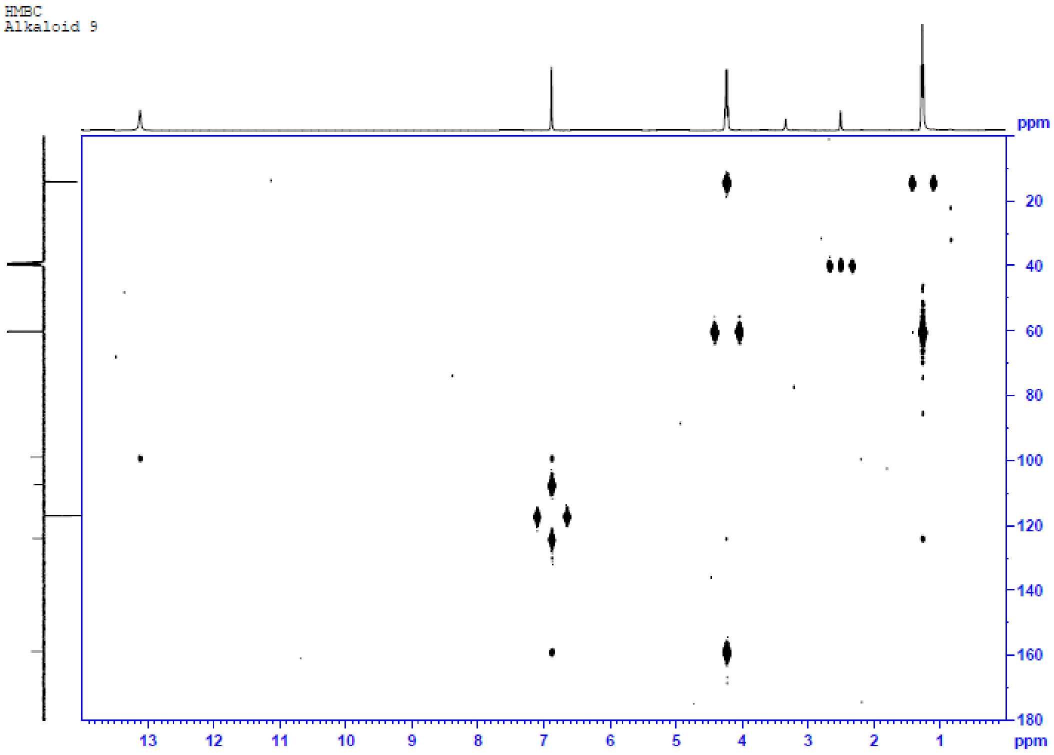

HMBC spectrum of **9** (DMSO-d_6, 400MHz)

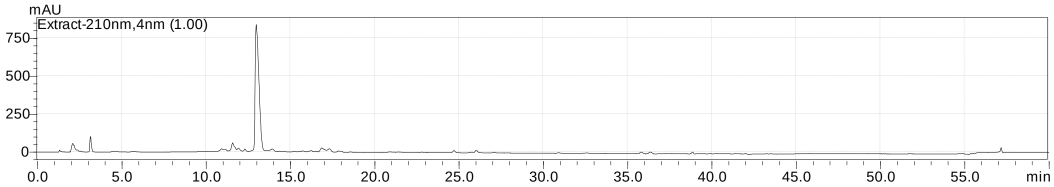

The DAD-HPLC of **10** (0-40min, 5%-100%MeOH-H_2O)

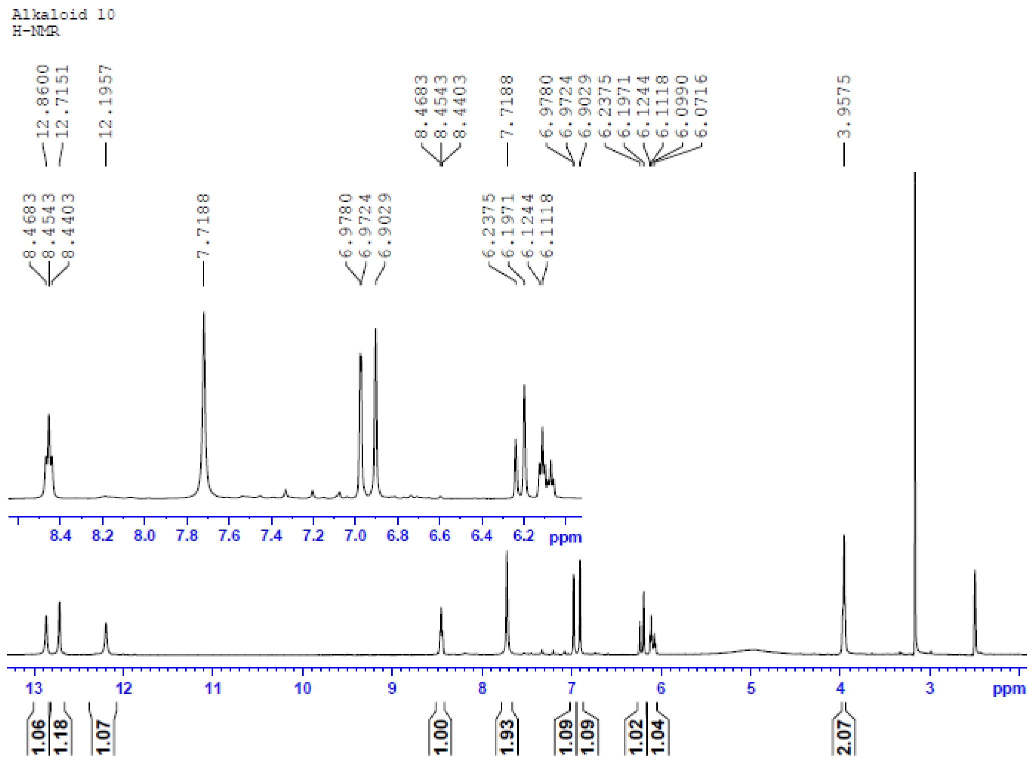

^{1}H-NMR spectrum of **10** (DMSO-d_6, 400MHz)

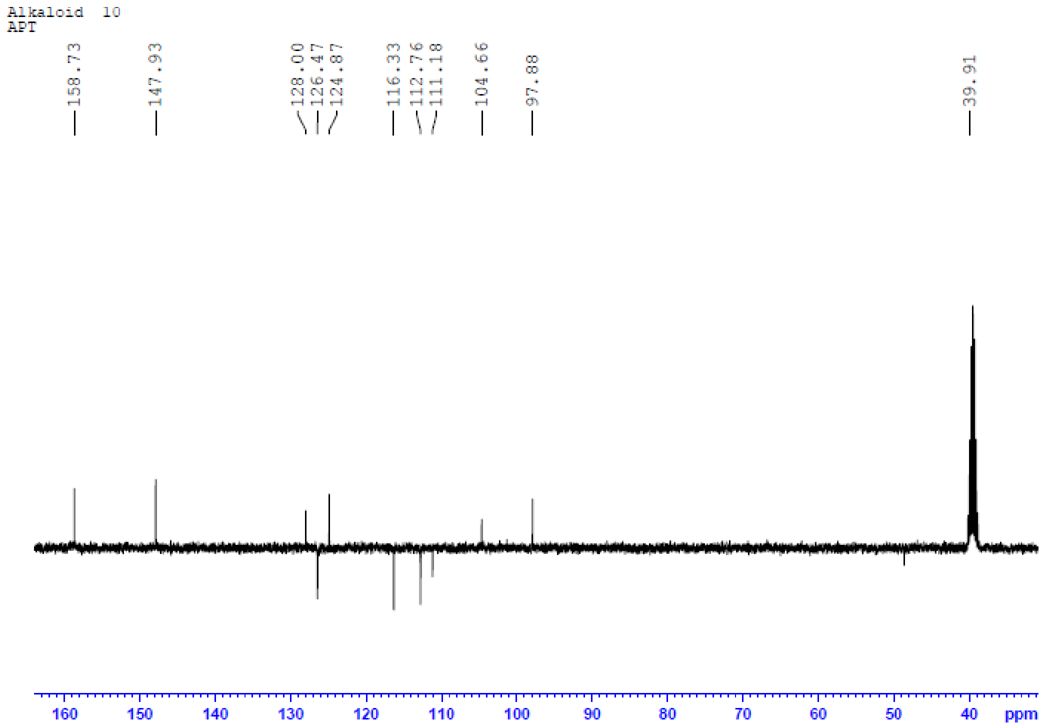

^{13}C-NMR spectrum of **10** (DMSO-d_6, 100MHz)

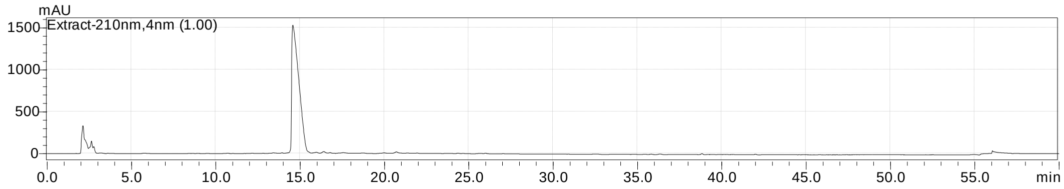

The DAD-HPLC of **11** (0-40min, 5%-100%MeOH-H_2O)

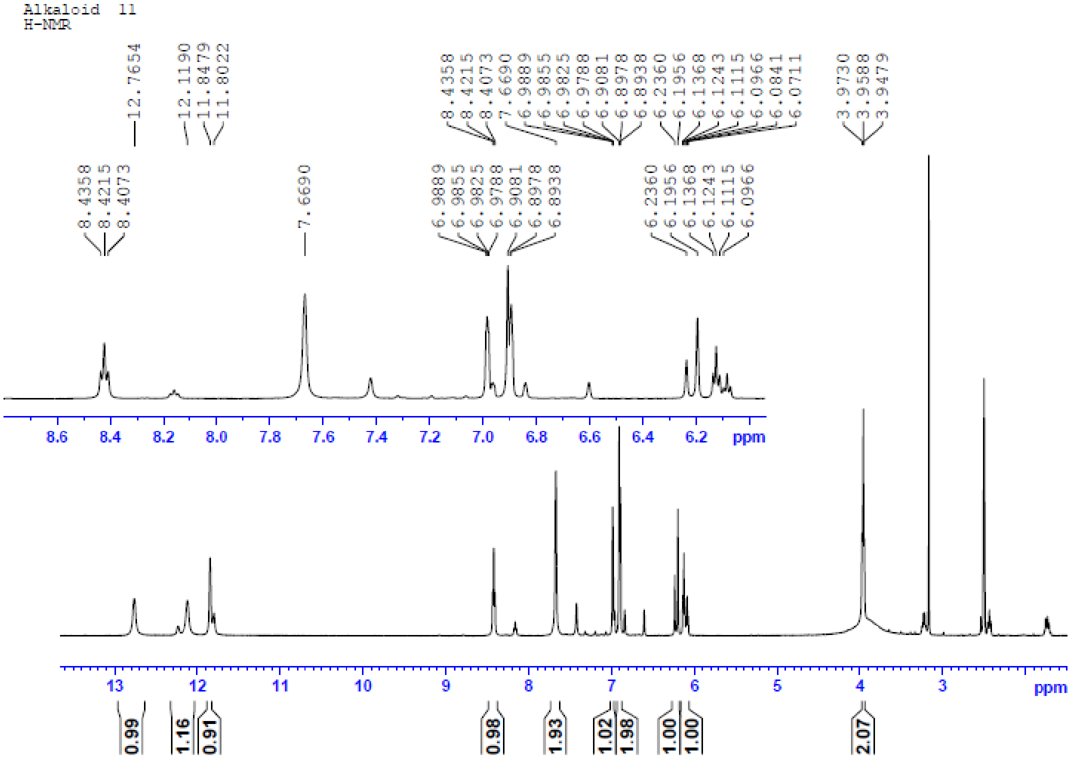

^{1}H-NMR spectrum of **11** (DMSO-d_6, 400MHz)

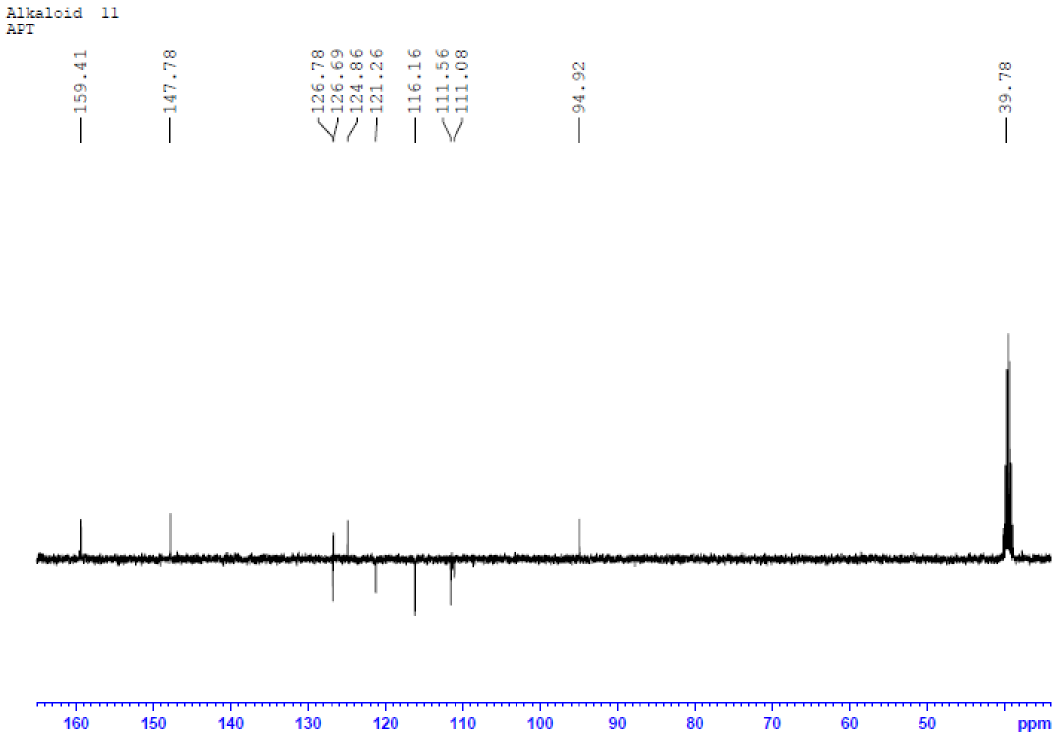

^{13}C-NMR spectrum of **11** (DMSO-d_6, 100MHz)

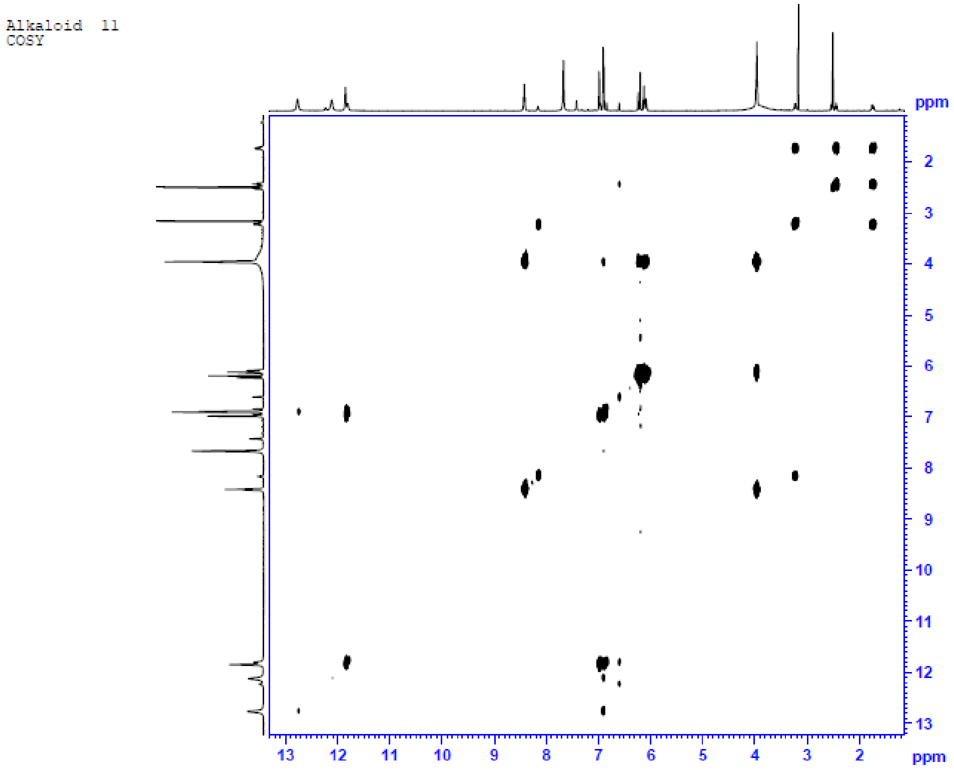

^{1}H-^{1}H COSY spectrum of **11** (DMSO-d_6, 400MHz)

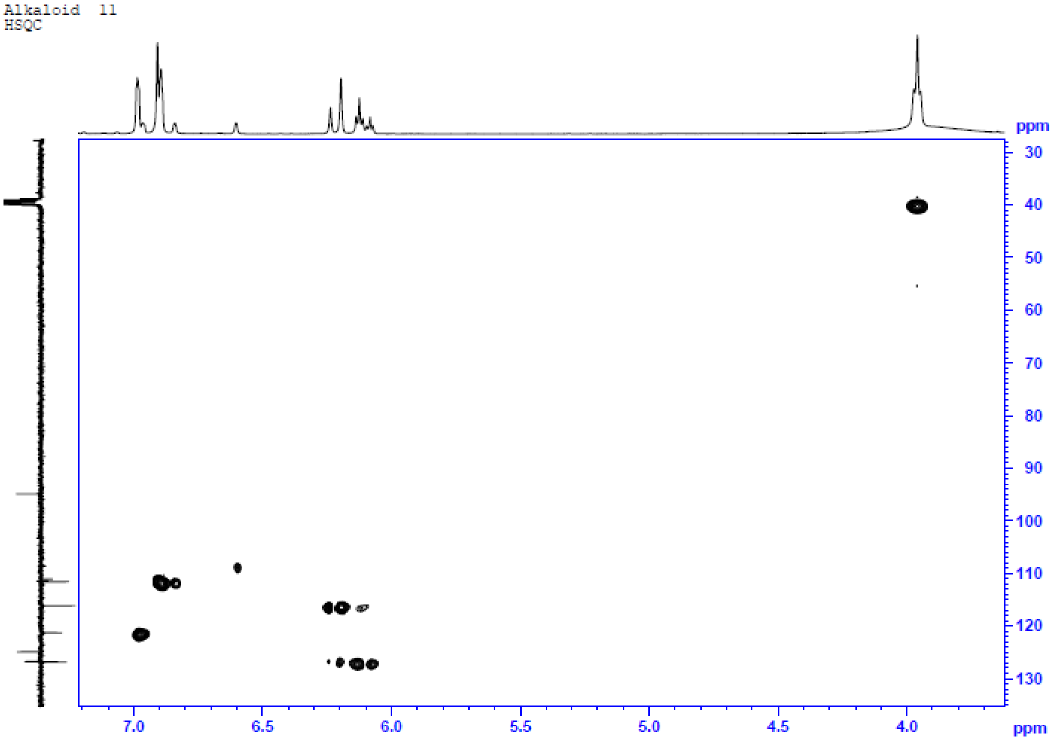

HSQC spectrum of **11** (DMSO-d_6, 400MHz)

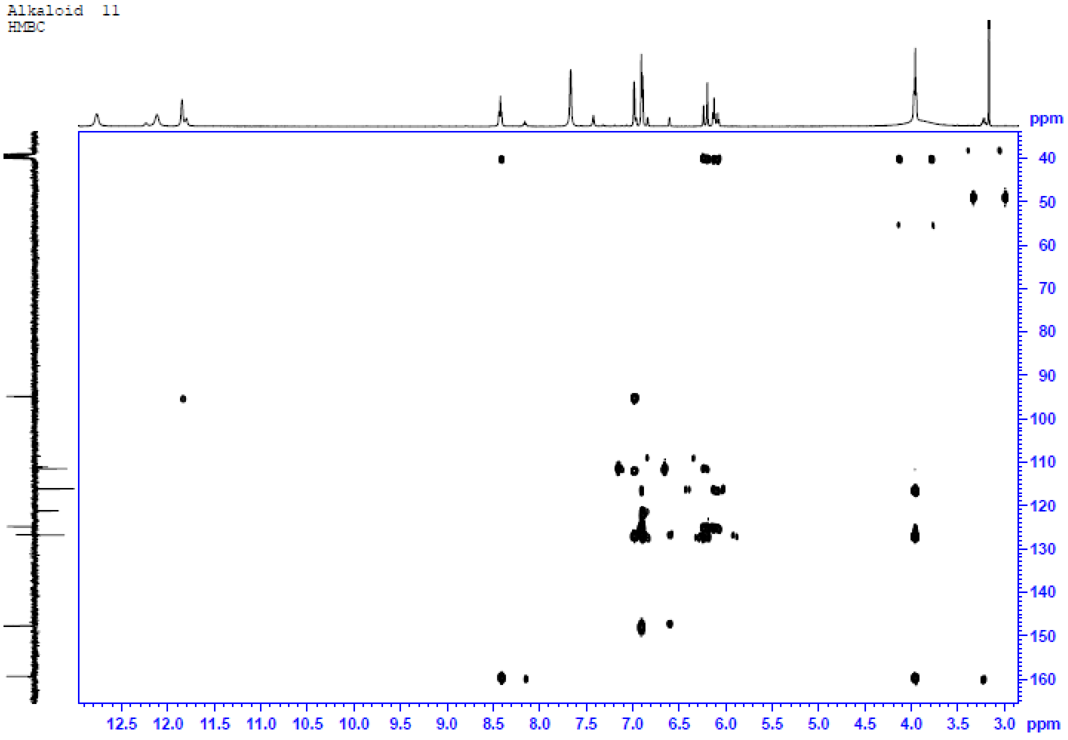

HMBC spectrum of **11** (DMSO-d_6, 400MHz)

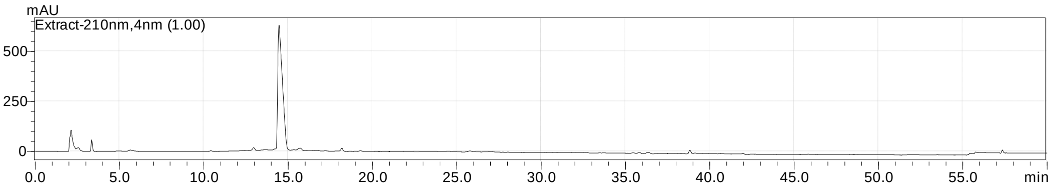

The DAD-HPLC of **12** (0-40min, 5%-100%MeOH-H_2O)

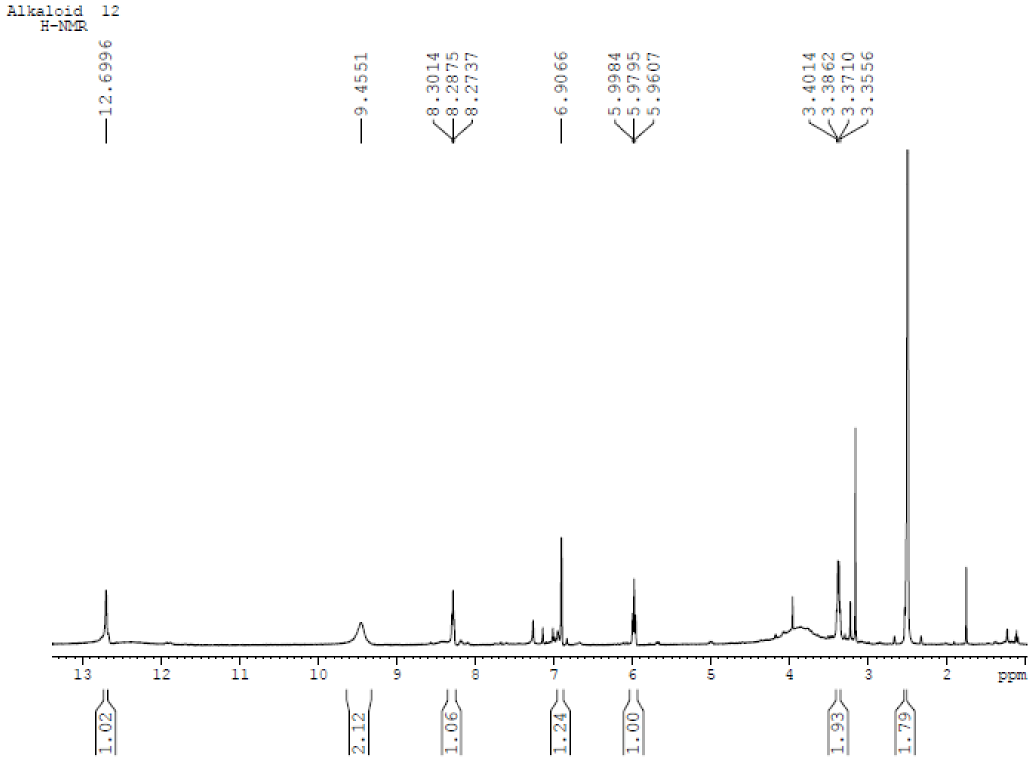

^{1}H-NMR spectrum of **12** (DMSO-d_6, 400MHz)

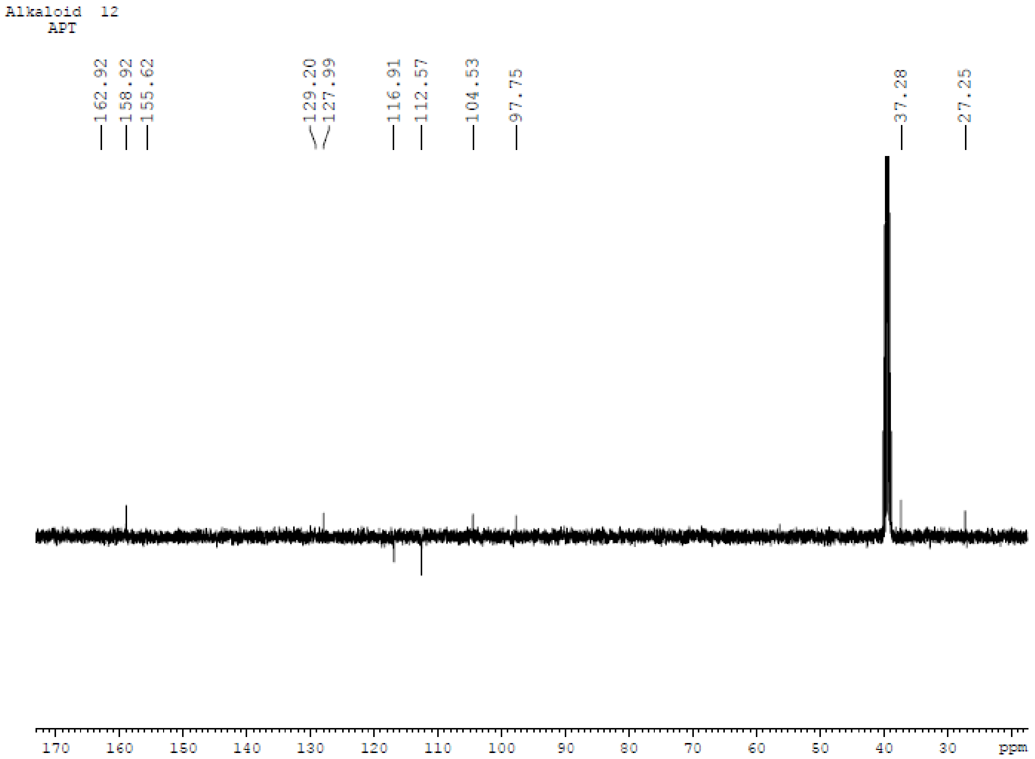

^{13}C-NMR spectrum of **12** (DMSO-d_6, 100MHz)

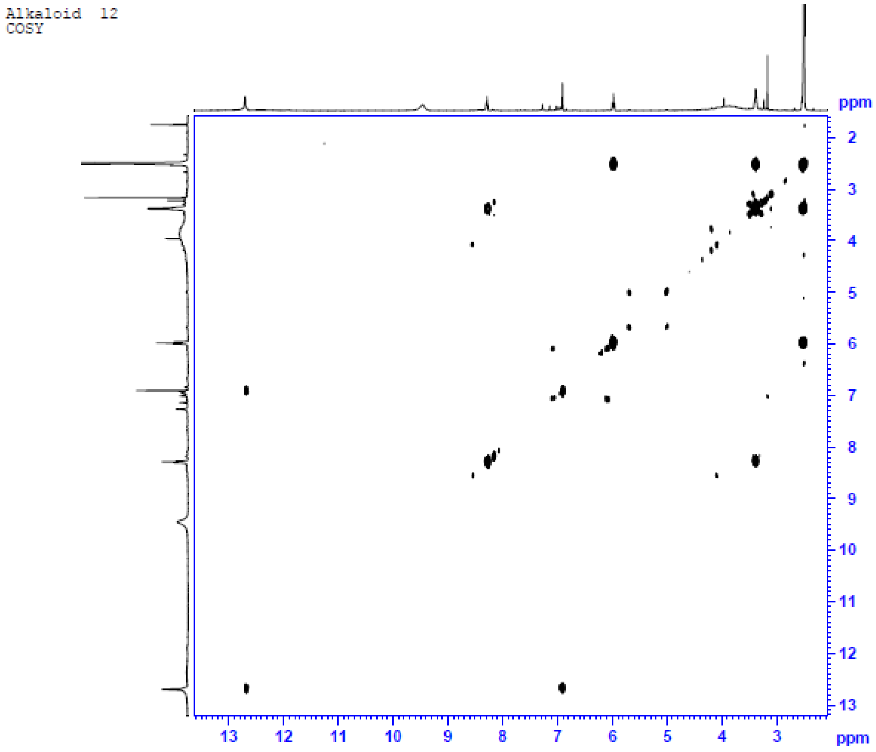

^{1}H-^{1}H COSY spectrum of **12** (DMSO-d_6, 400MHz)

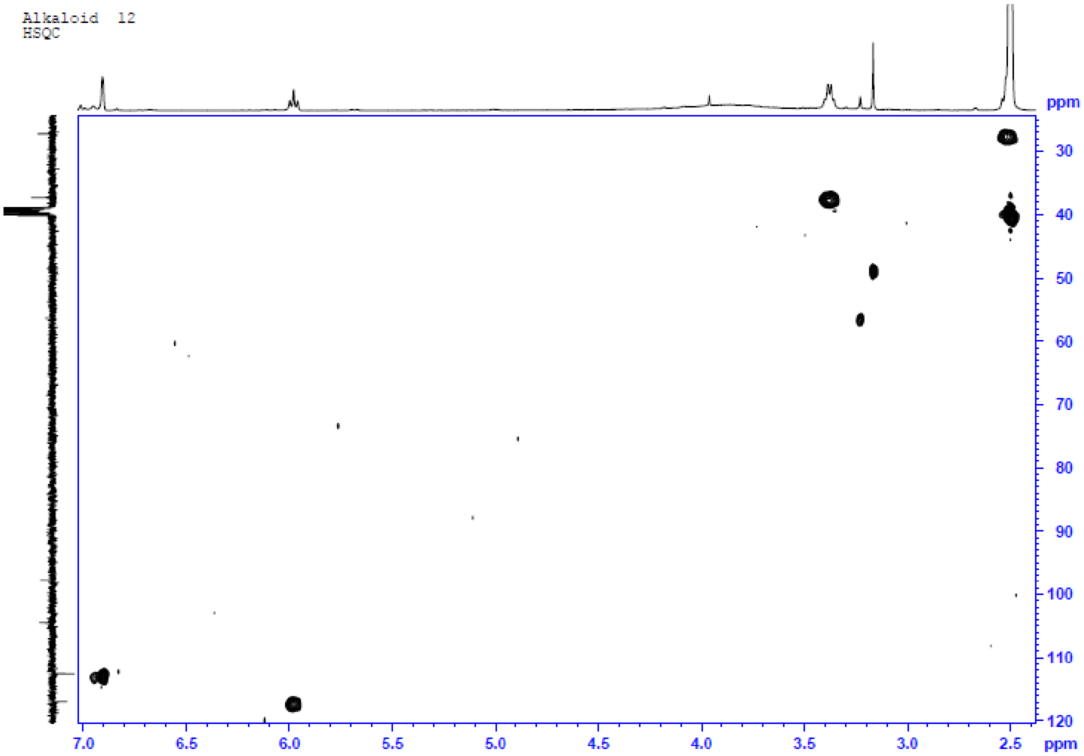

HSQC spectrum of **12** (DMSO-d_6, 400MHz)

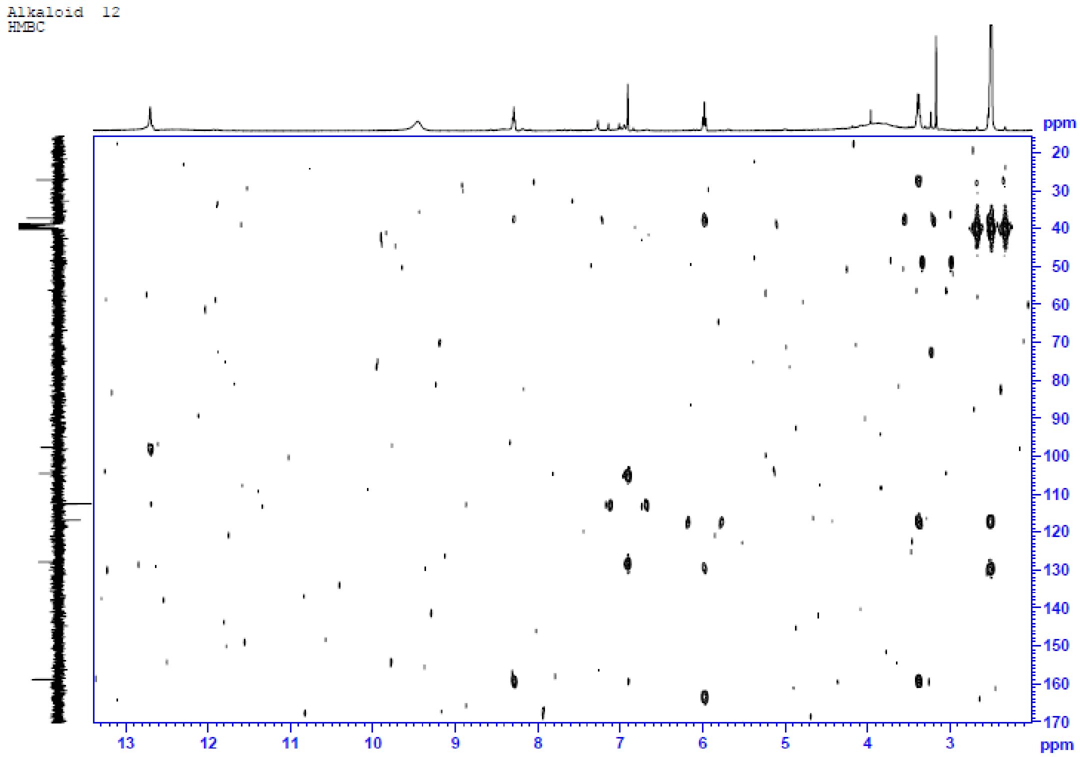

HMBC spectrum of **12** (DMSO-d_6, 400MHz)

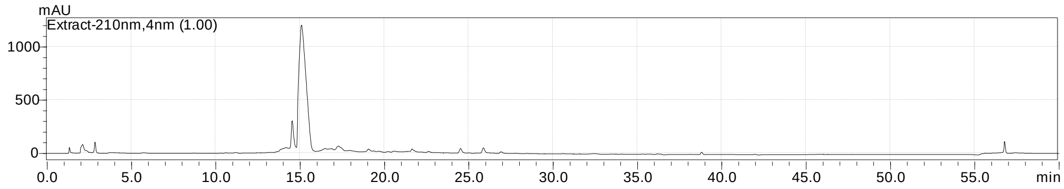

The DAD-HPLC of **13/14** (0-40min, 5%-100%MeOH-H_2O)

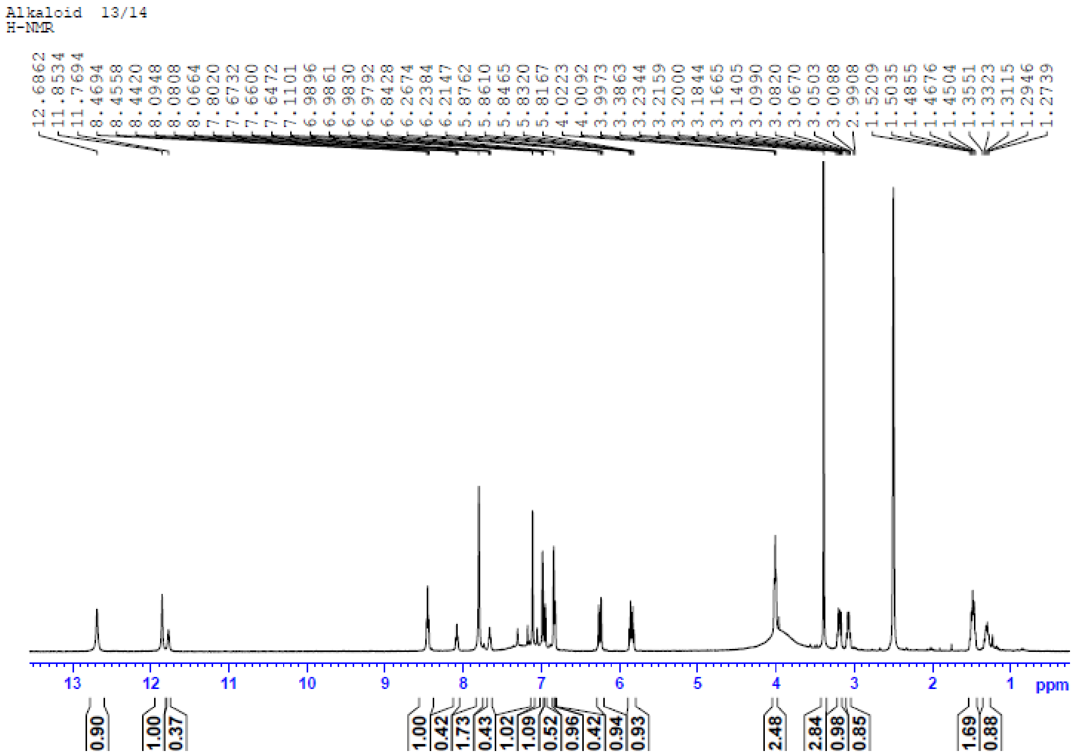

^{1}H-NMR spectrum of **13/14** (DMSO-d_6, 400MHz)

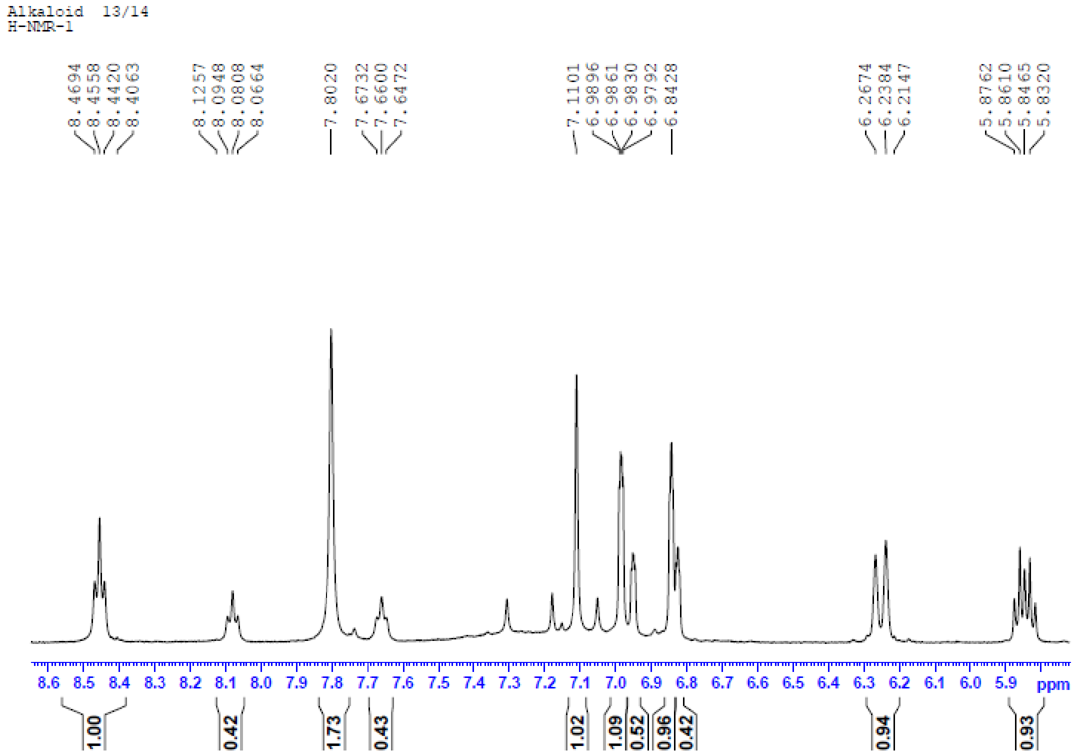

^{1}H-NMR spectrum of **13/14** (DMSO-d_6, 400MHz), expansion-1

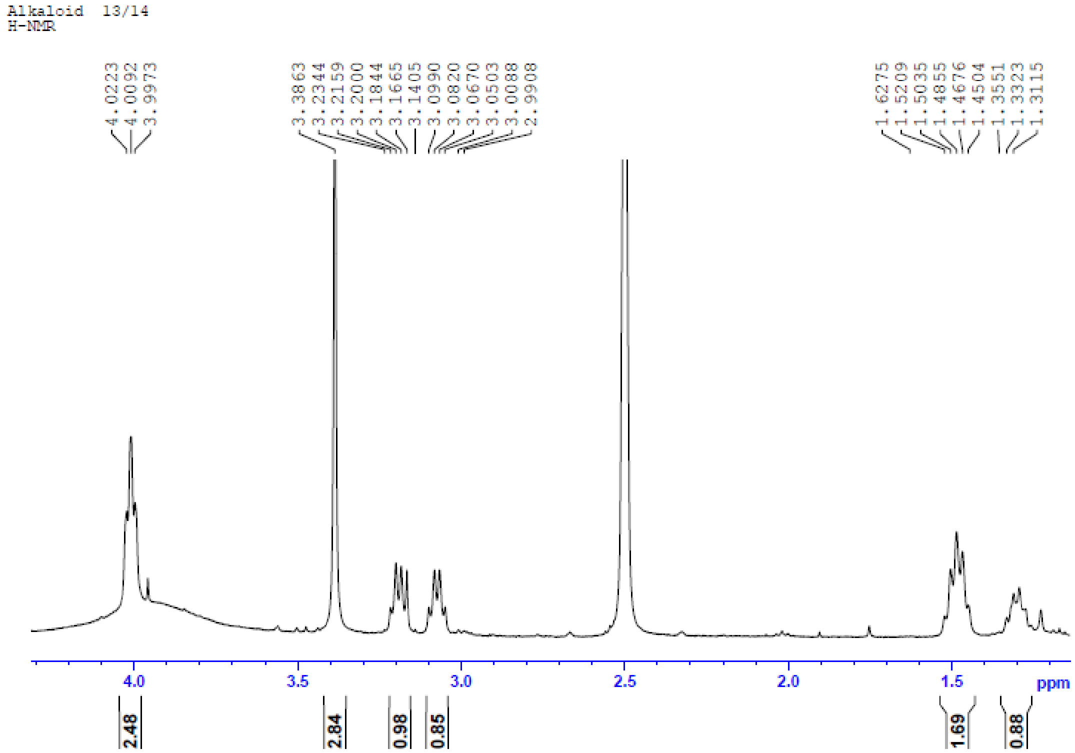

^{1}H-NMR spectrum of **13/14** (DMSO-d_6, 400MHz), expansion-2

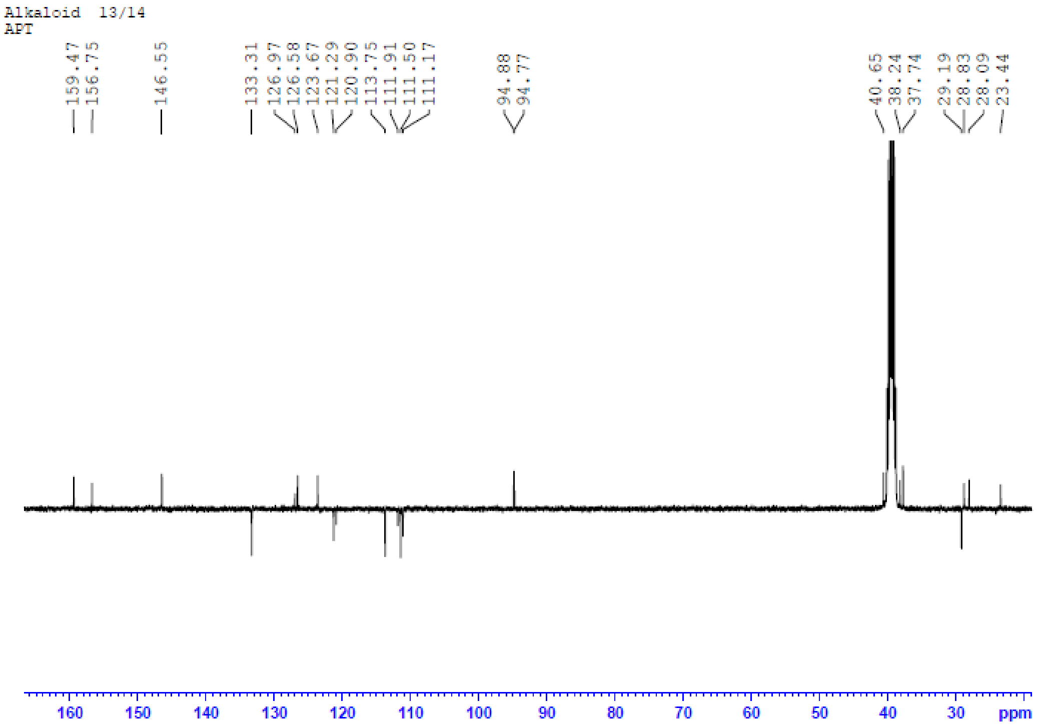

^{13}C-NMR spectrum of **13/14** (DMSO-d_6, 100MHz)

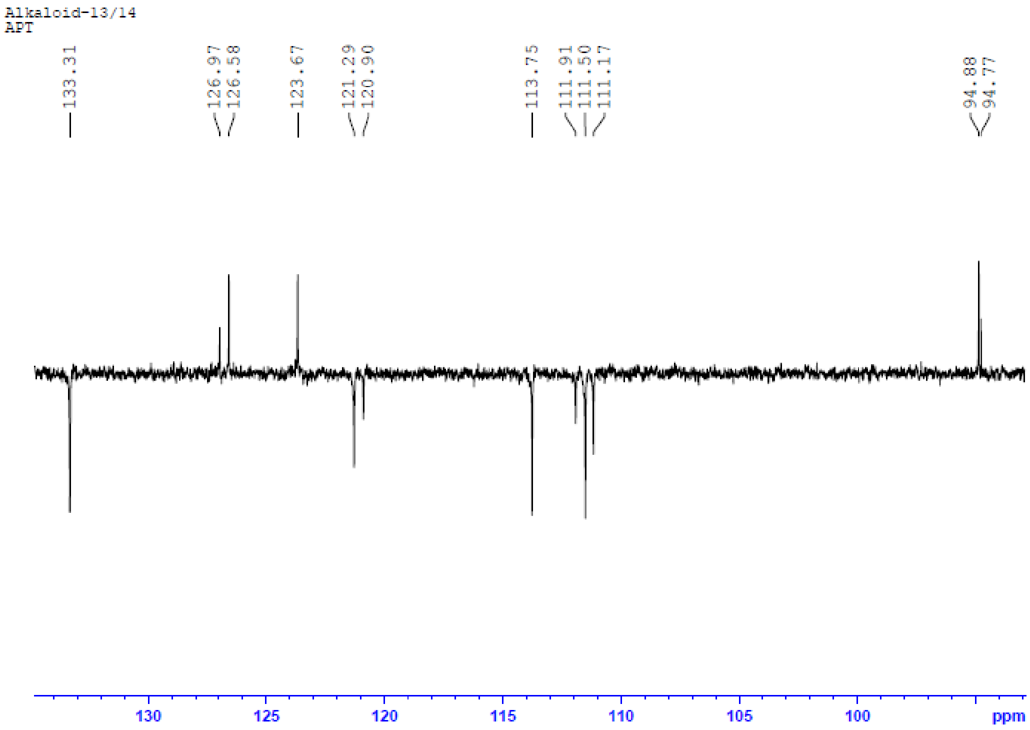

^{13}C-NMR spectrum of **13/14** (DMSO-d_6, 100MHz), expansion-1

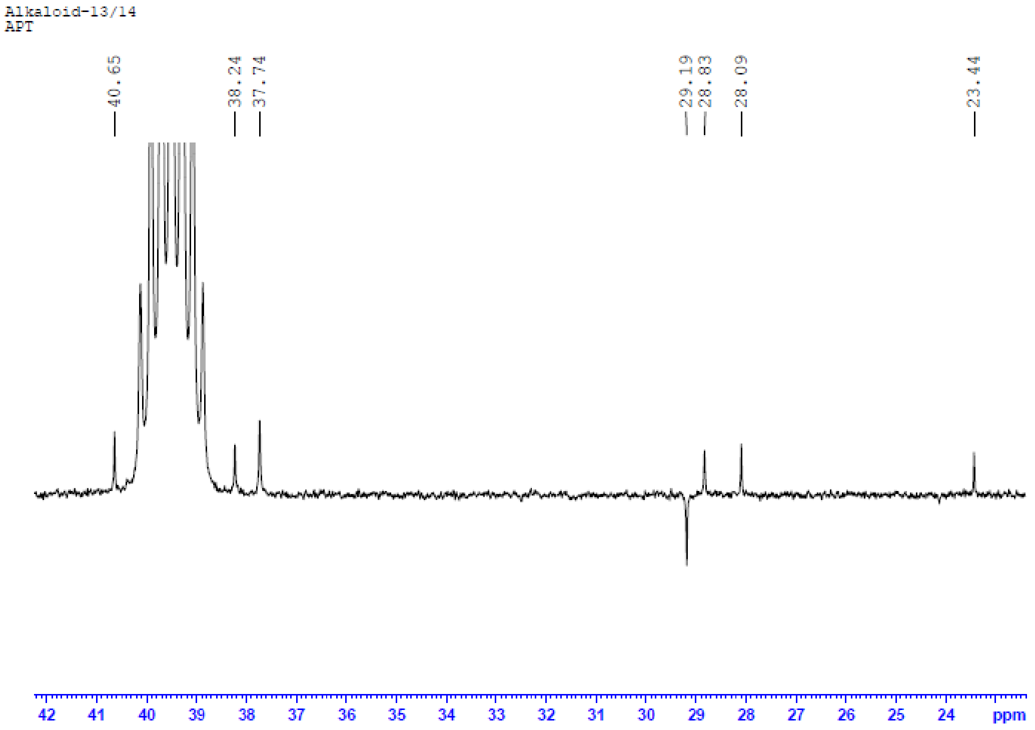

^{13}C-NMR spectrum of **13/14** (DMSO-d_6, 100MHz), expansion-2

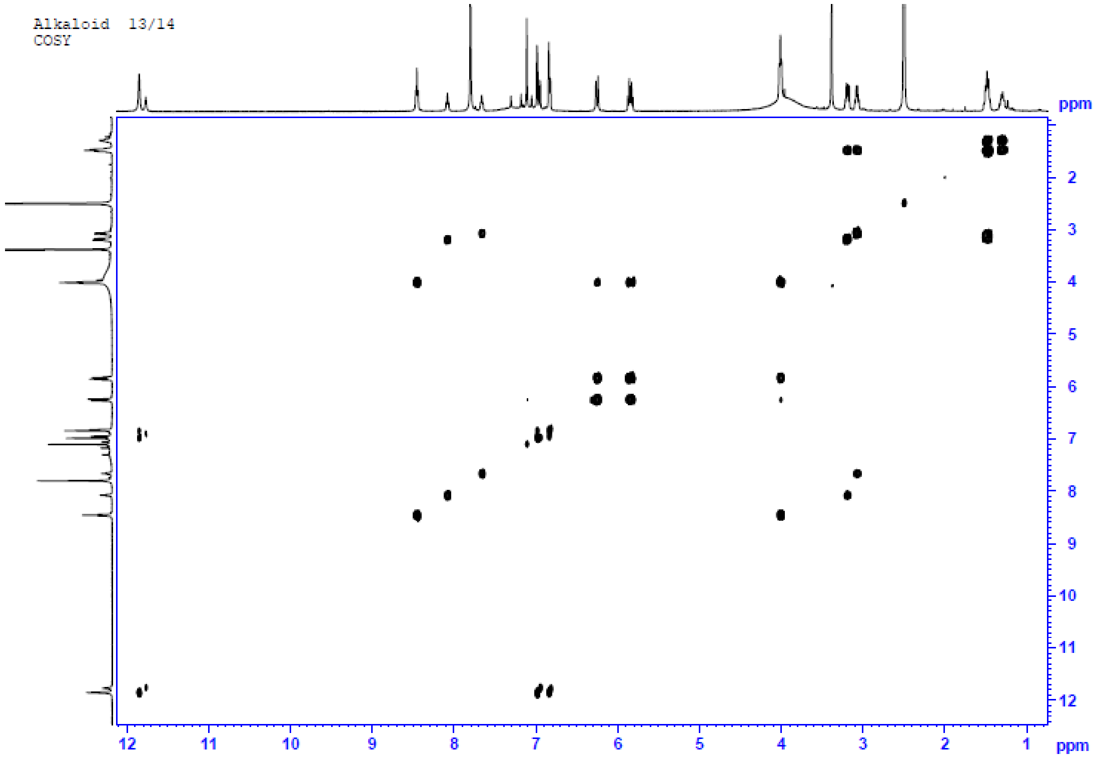

^{1}H-^{1}H COSY spectrum of **13/14** (DMSO-d_6, 400MHz)

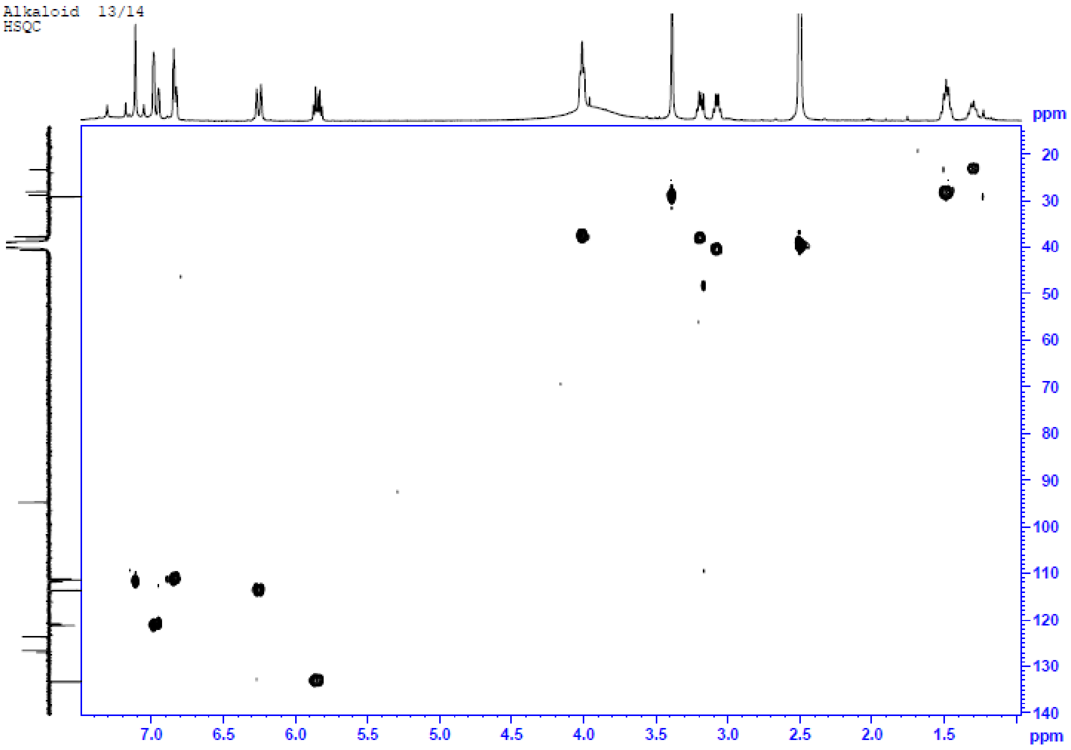

HSQC spectrum of **13/14** (DMSO-d_6, 400MHz)

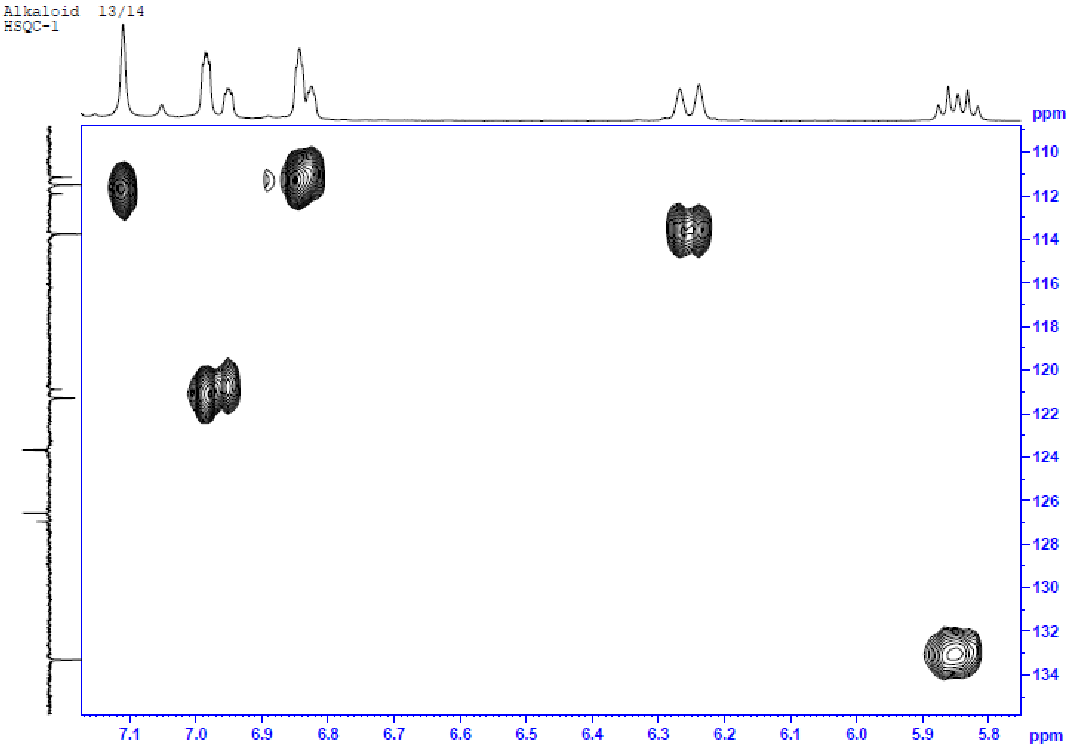

HSQC spectrum of **13/14** (DMSO-d_6, 400MHz), expansion-1

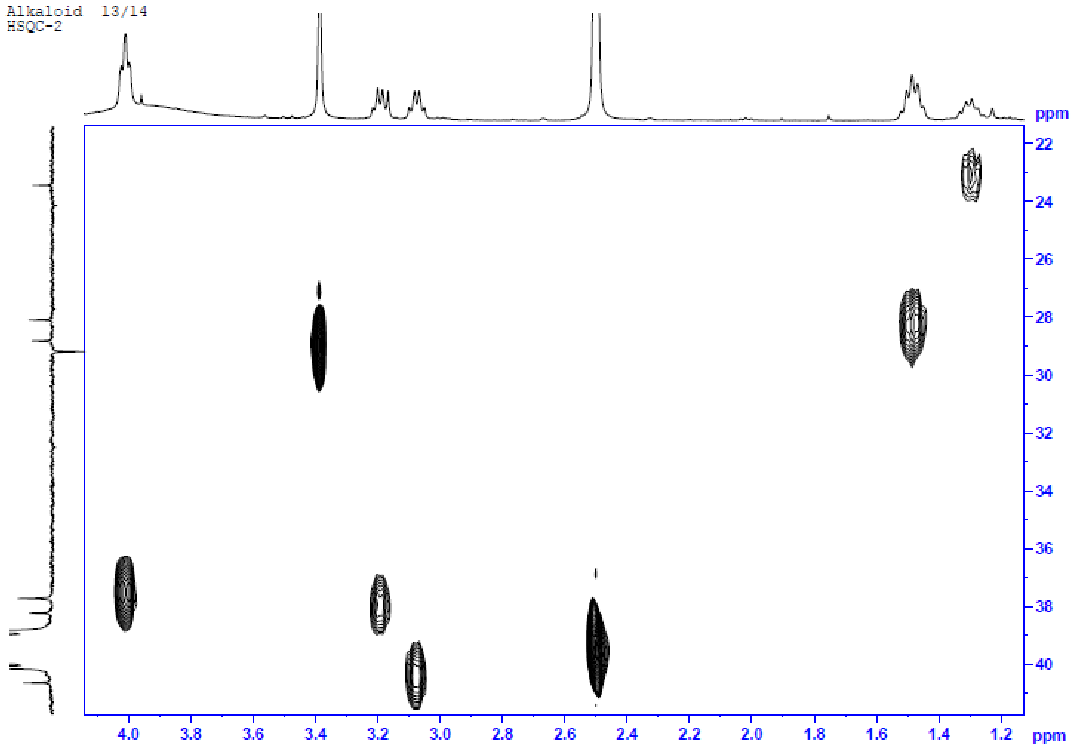

HSQC spectrum of **13/14** (DMSO-d_6, 400MHz), expansion-2

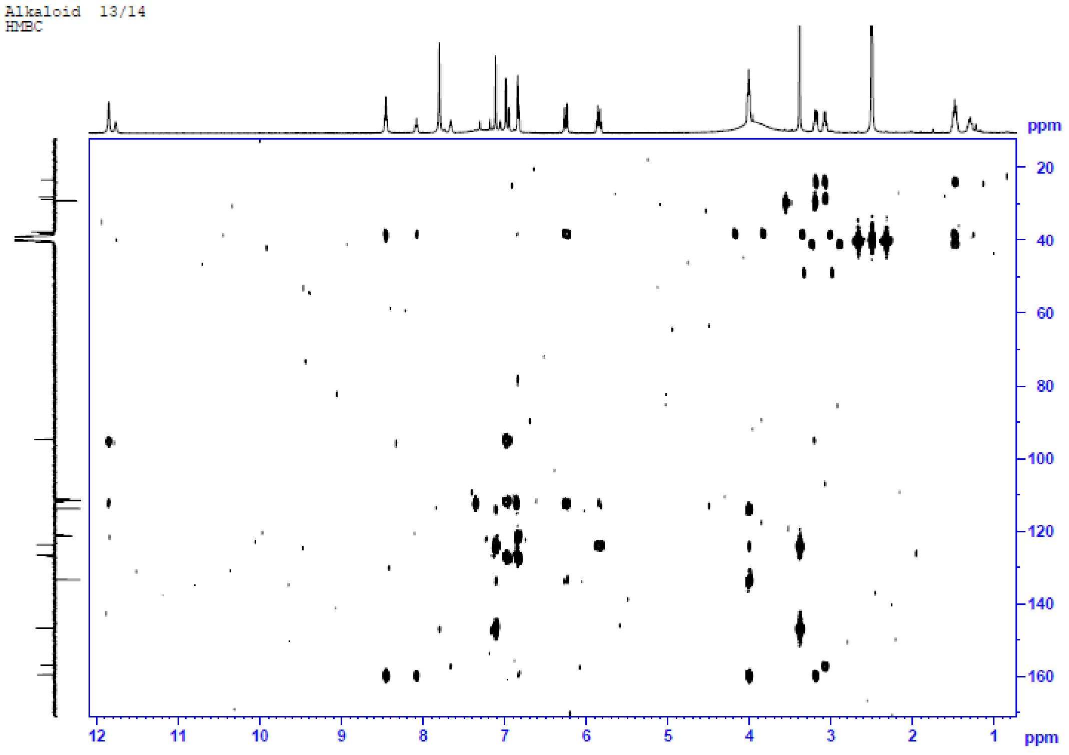

HMBC spectrum of **13/14** (DMSO-d_6, 400MHz)

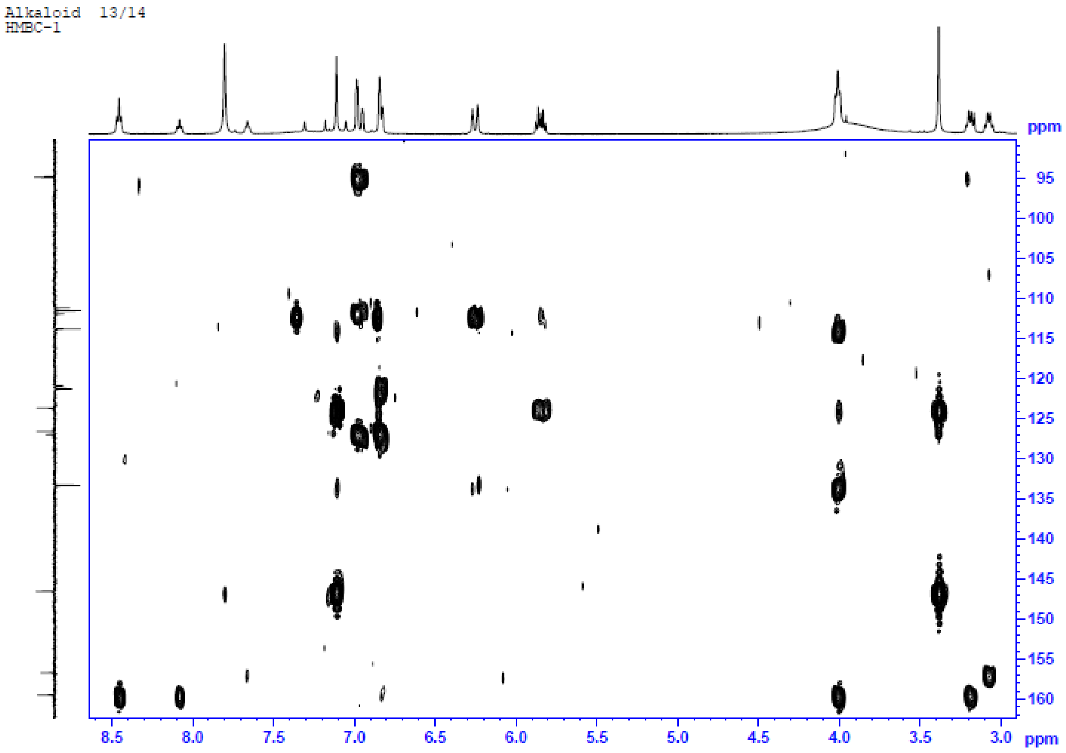

HMBC spectrum of **13/14** (DMSO-d_6, 400MHz), expansion-1

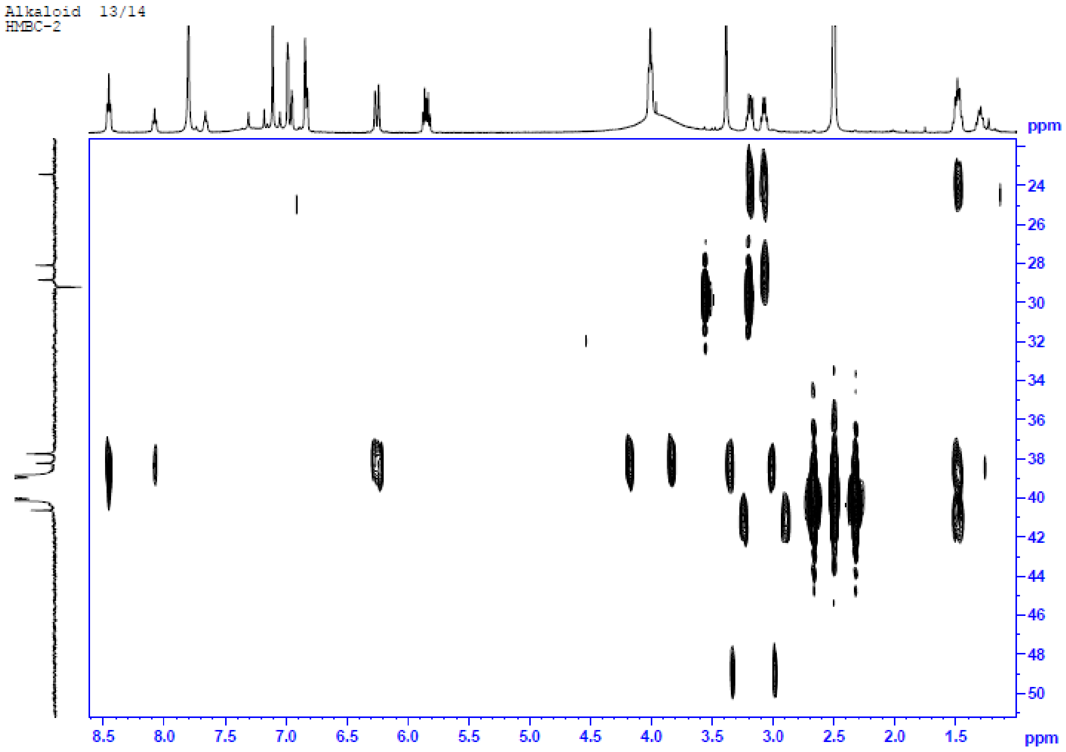

HMBC spectrum of **13/14** (DMSO-d_6, 400MHz), expansion-2

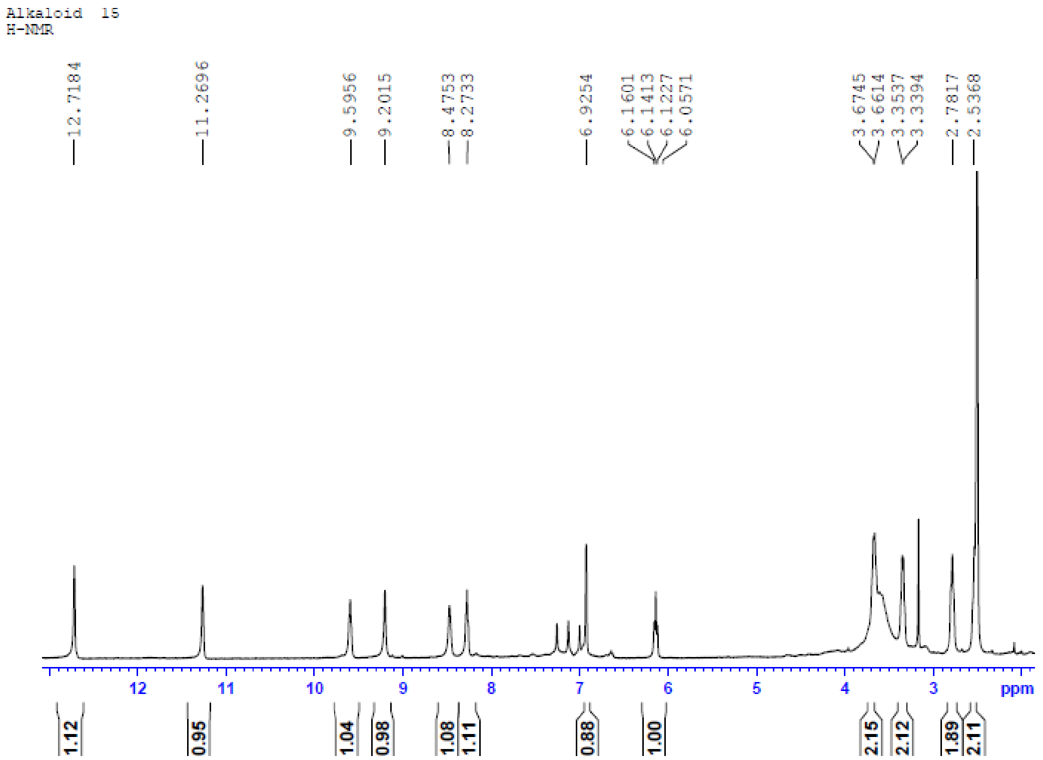

^{1}H-NMR spectrum of **15** (DMSO-d_6, 400MHz)

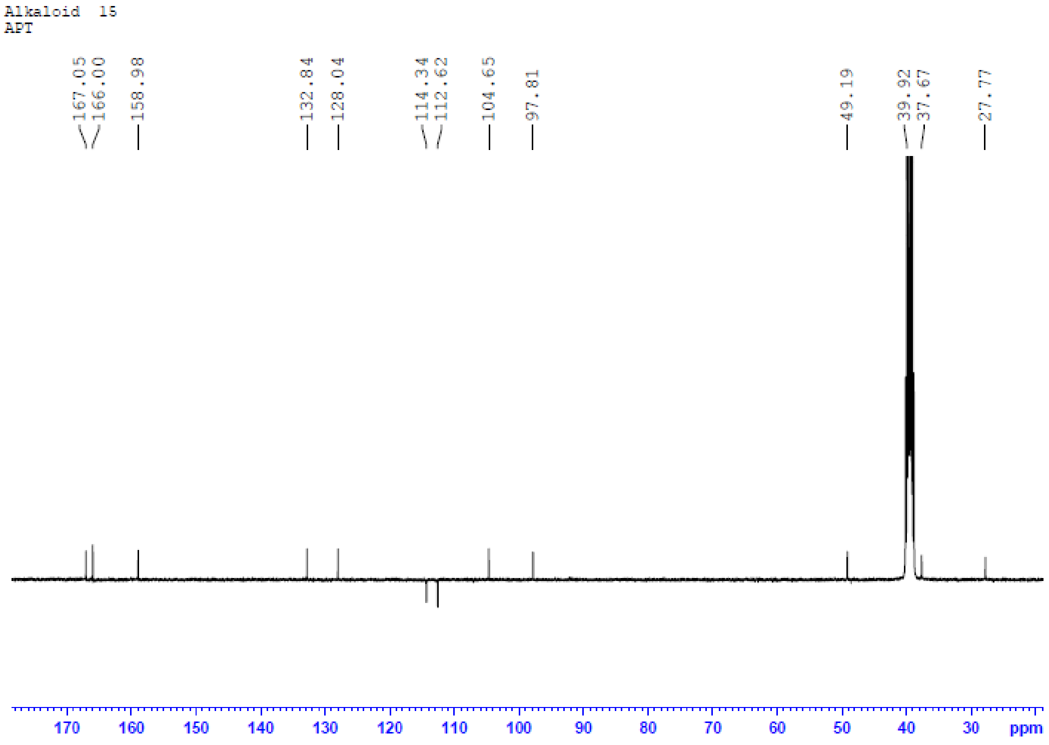

^{13}C-NMR spectrum of **15** (DMSO-d_6, 100MHz)

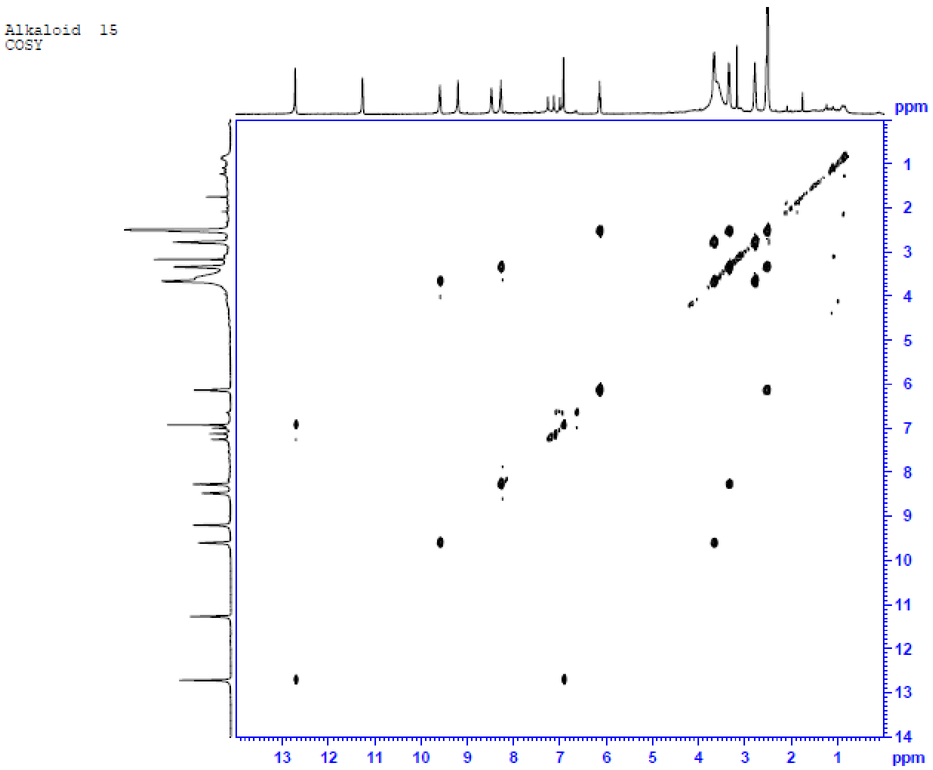

^{1}H-^{1}H COSY spectrum of **15** (DMSO-d_6, 400MHz)

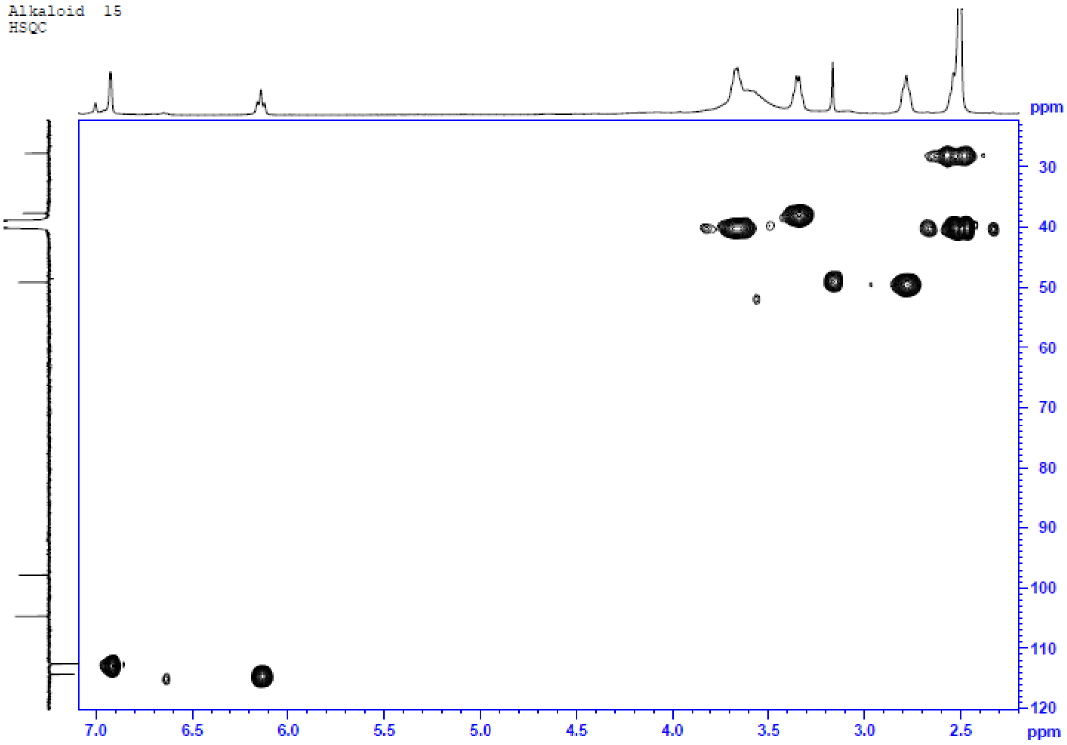

HSQC spectrum of **15** (DMSO-d_6, 400MHz)

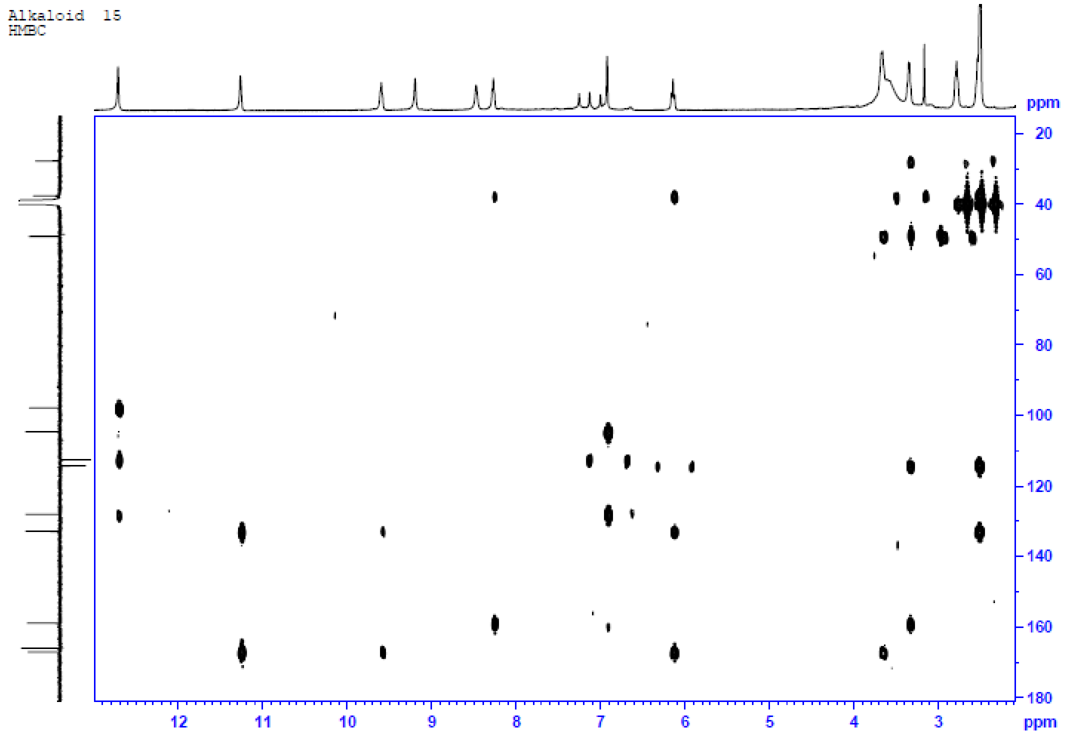

HMBC spectrum of **15** (DMSO-d_6, 400MHz)

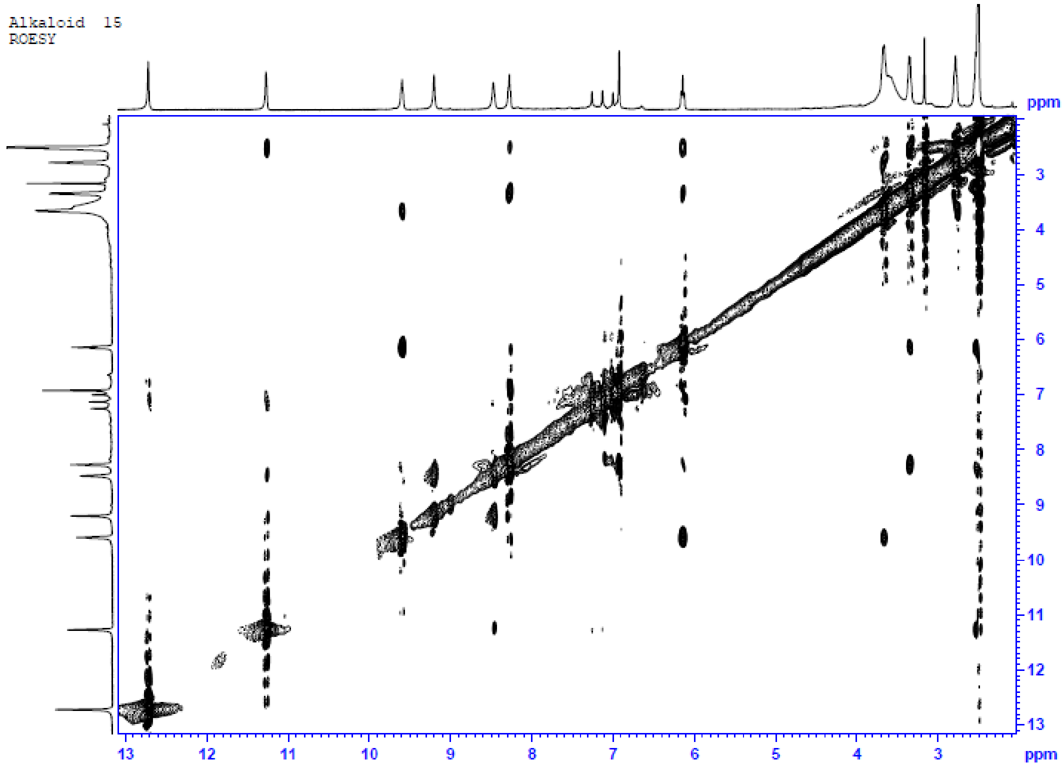

ROESY spectrum of **15** (DMSO-d_6, 400MHz)

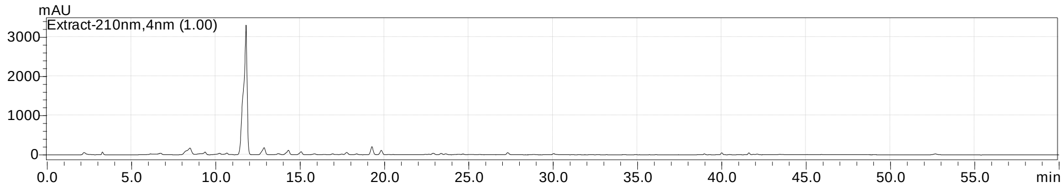

The DAD-HPLC of **16** (0-40min, 5%-100%MeOH-H_2O)

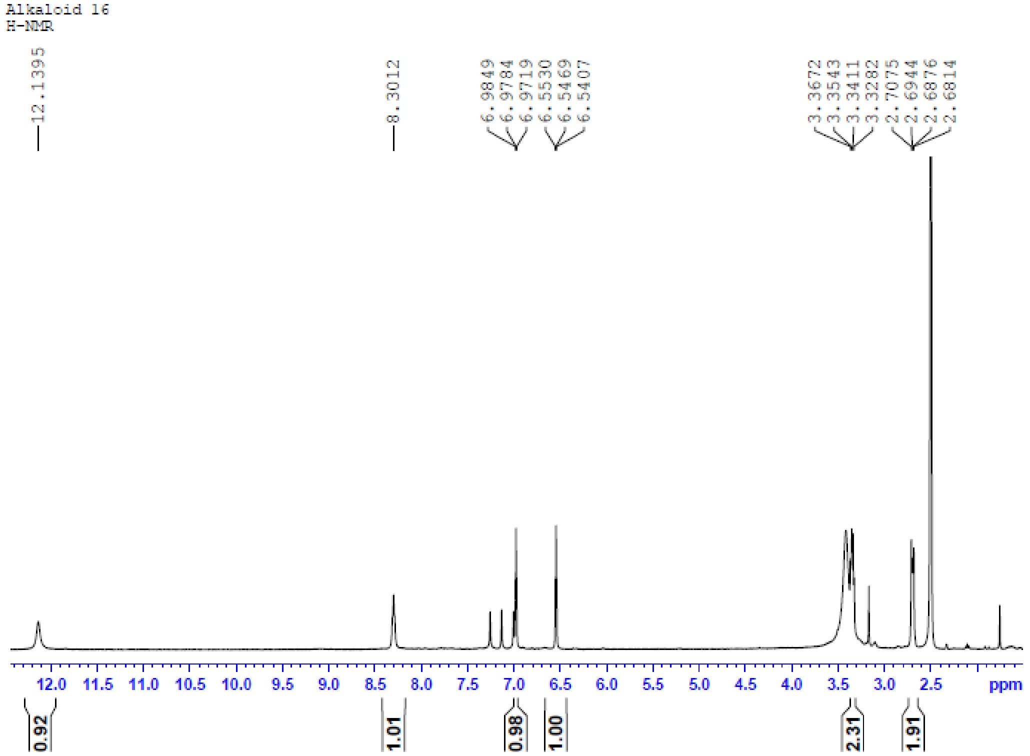

^{1}H-NMR spectrum of **16** (DMSO-d_6, 400MHz)

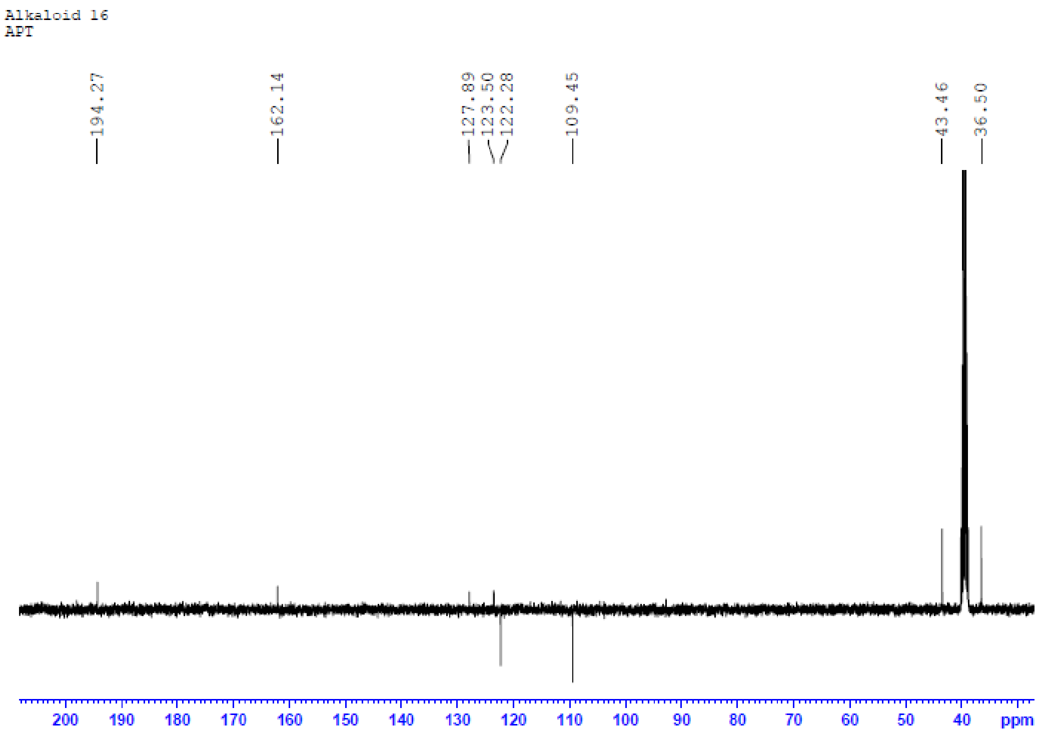

^{13}C-NMR spectrum of **16** (DMSO-d_6, 100MHz)

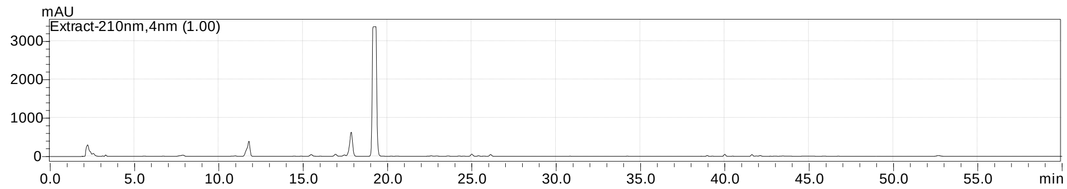

The DAD-HPLC of **17** (0-40min, 5%-100%MeOH-H_2O)

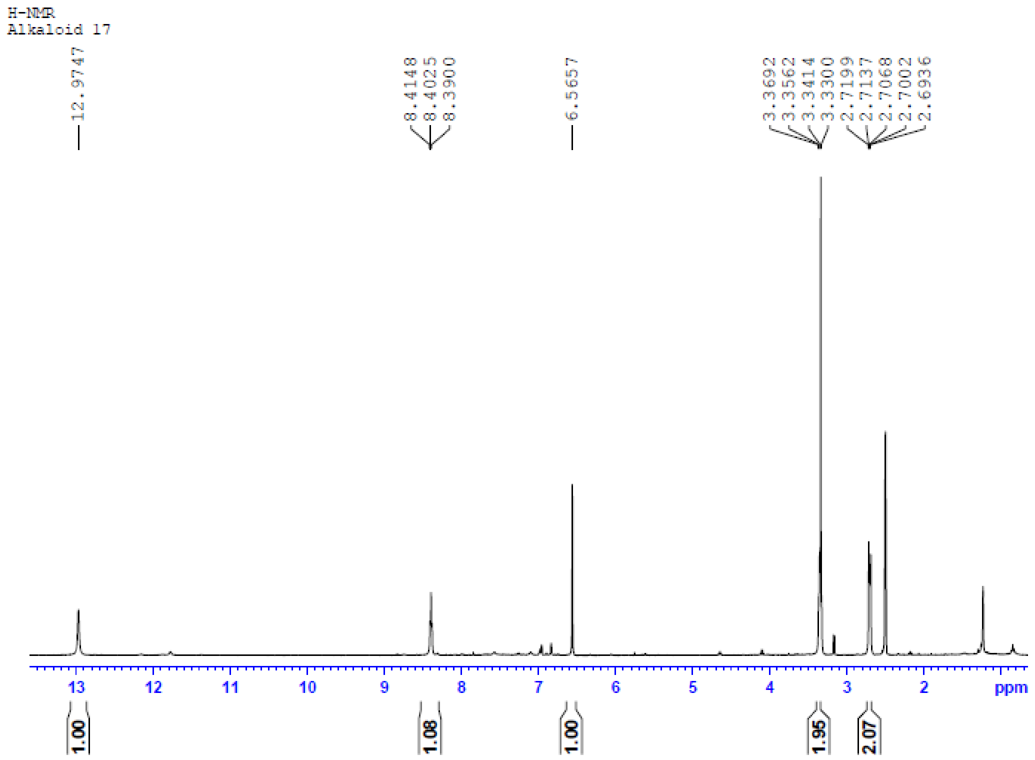

^{1}H-NMR spectrum of **17** (DMSO-d_6, 400MHz)

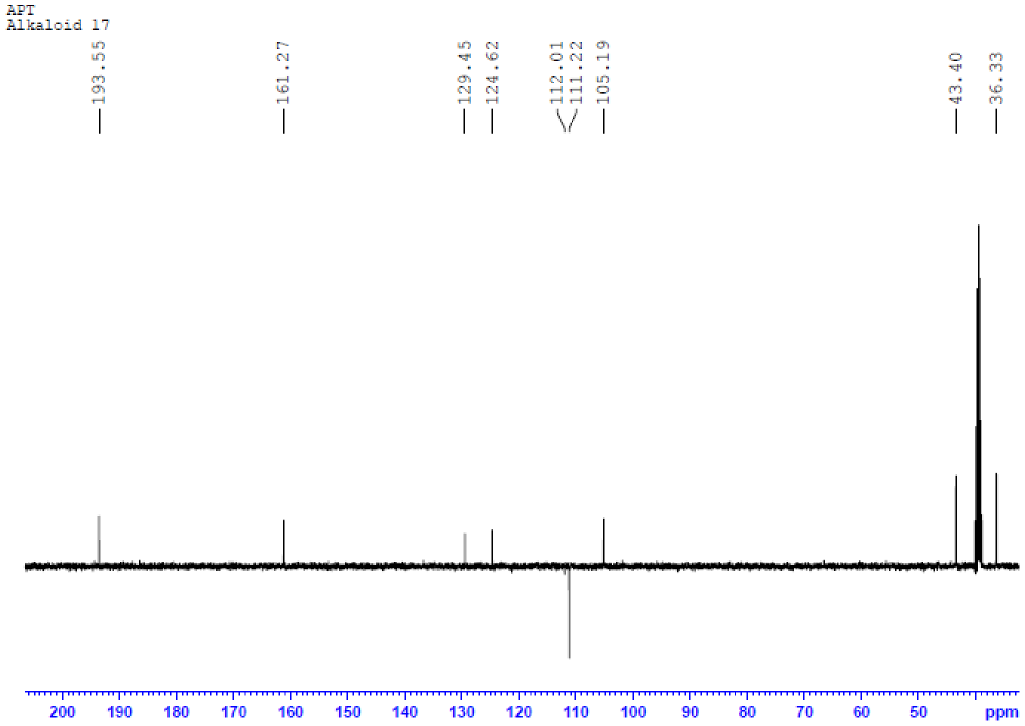

^{13}C-NMR spectrum of **17** (DMSO-d_6, 100MHz)

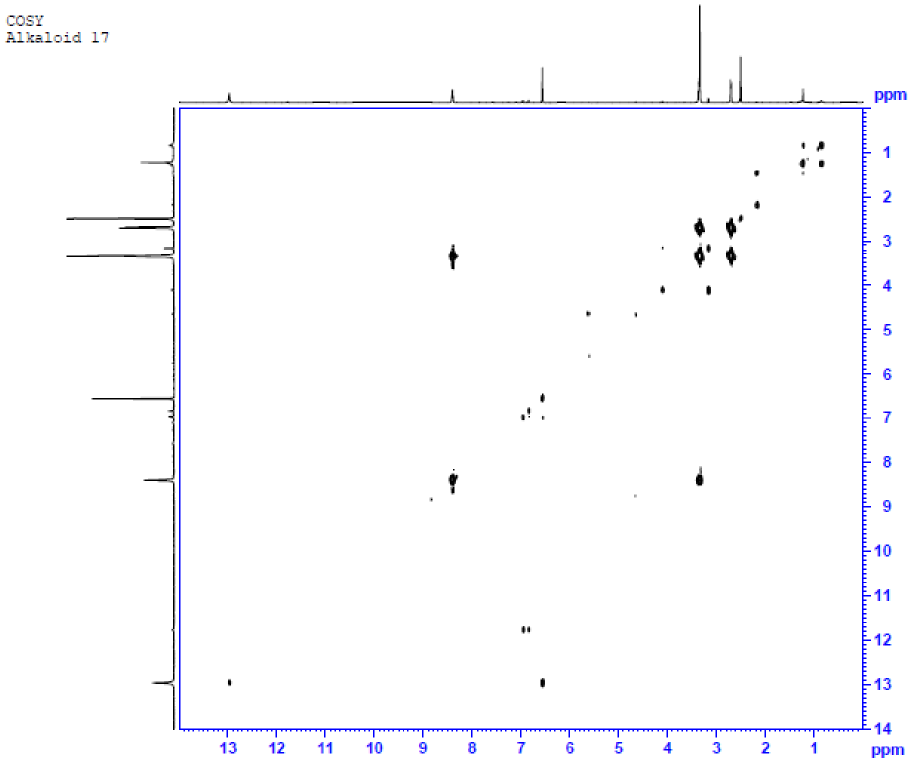

^{1}H-^{1}H COSY spectrum of **17** (DMSO-d_6, 400MHz)

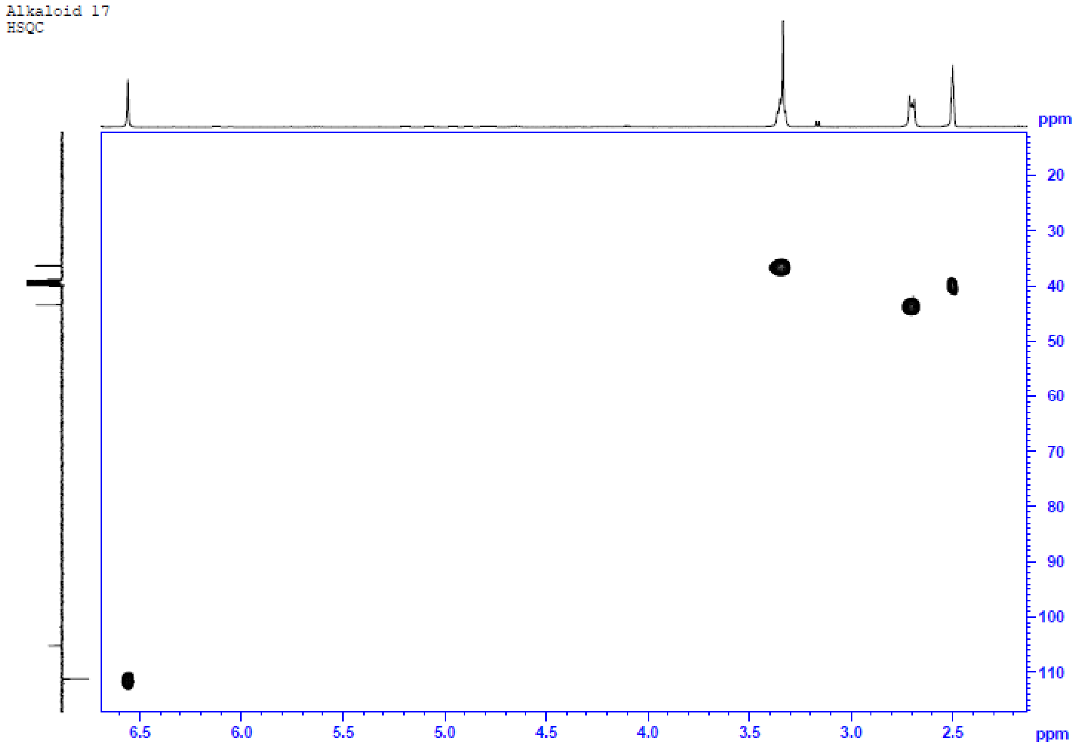

HSQC spectrum of **17** (DMSO-d_6, 400MHz)

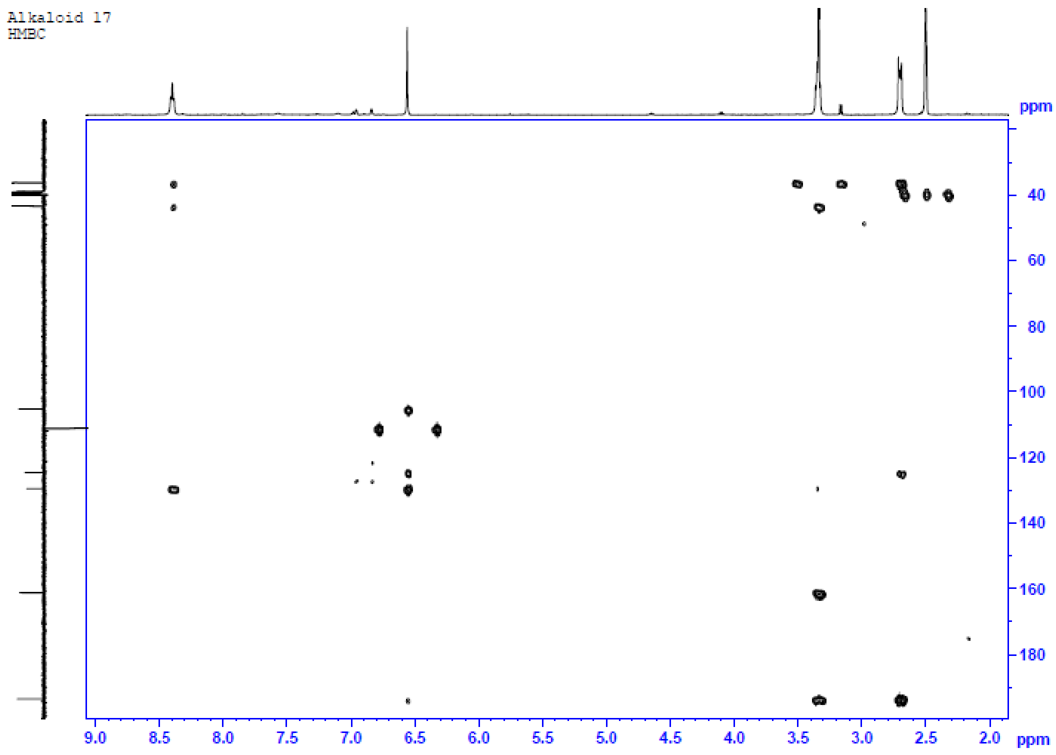

HMBC spectrum of **17** (DMSO-d_6, 400MHz)

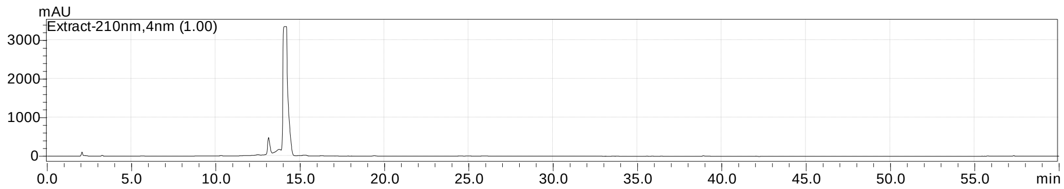

The DAD-HPLC of **18** (0-40min, 5%-100%MeOH-H_2O)

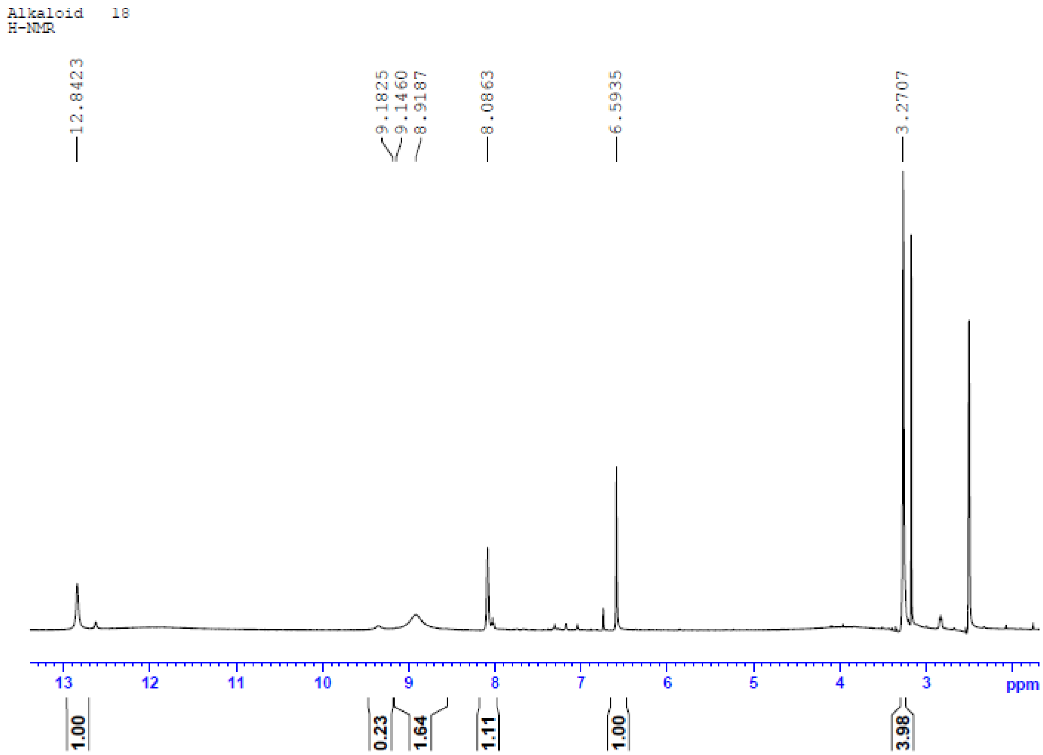

^{1}H-NMR spectrum of **18** (DMSO-d_6, 400MHz)

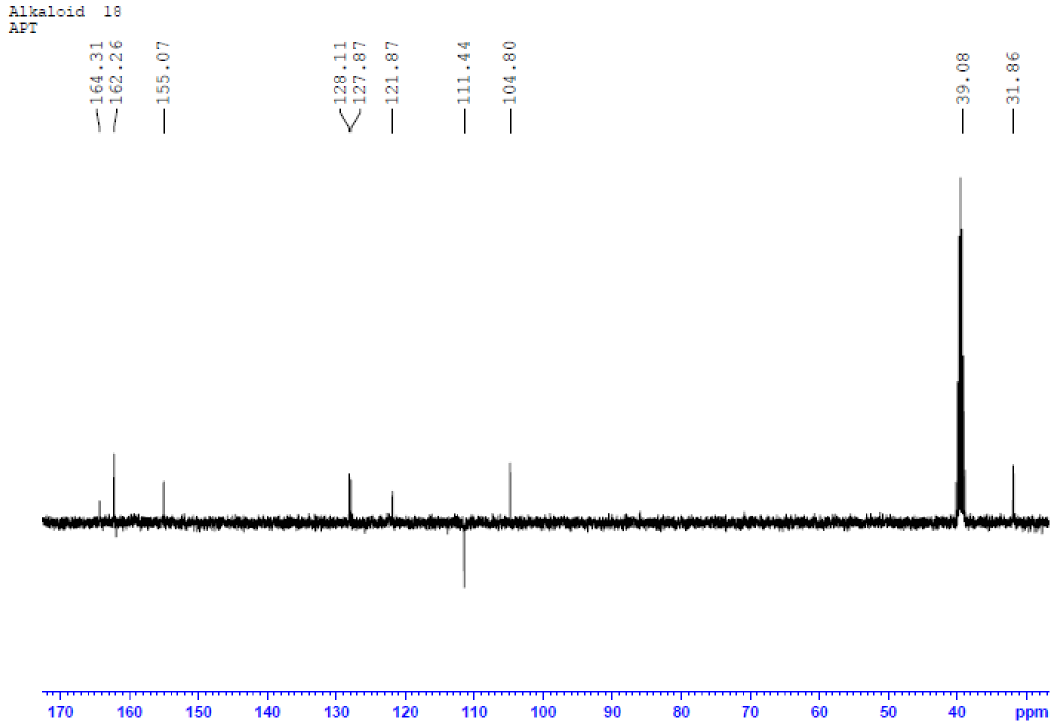

^{13}C-NMR spectrum of **18** (DMSO-d_6, 100MHz)

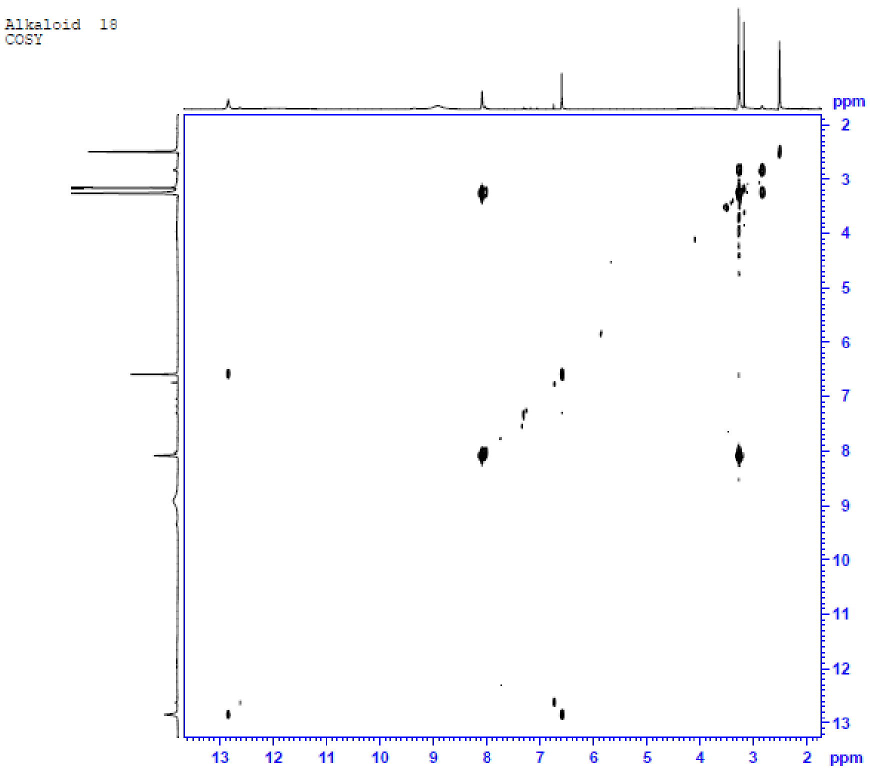

^{1}H-^{1}H COSY spectrum of **18** (DMSO-d_6, 400MHz)

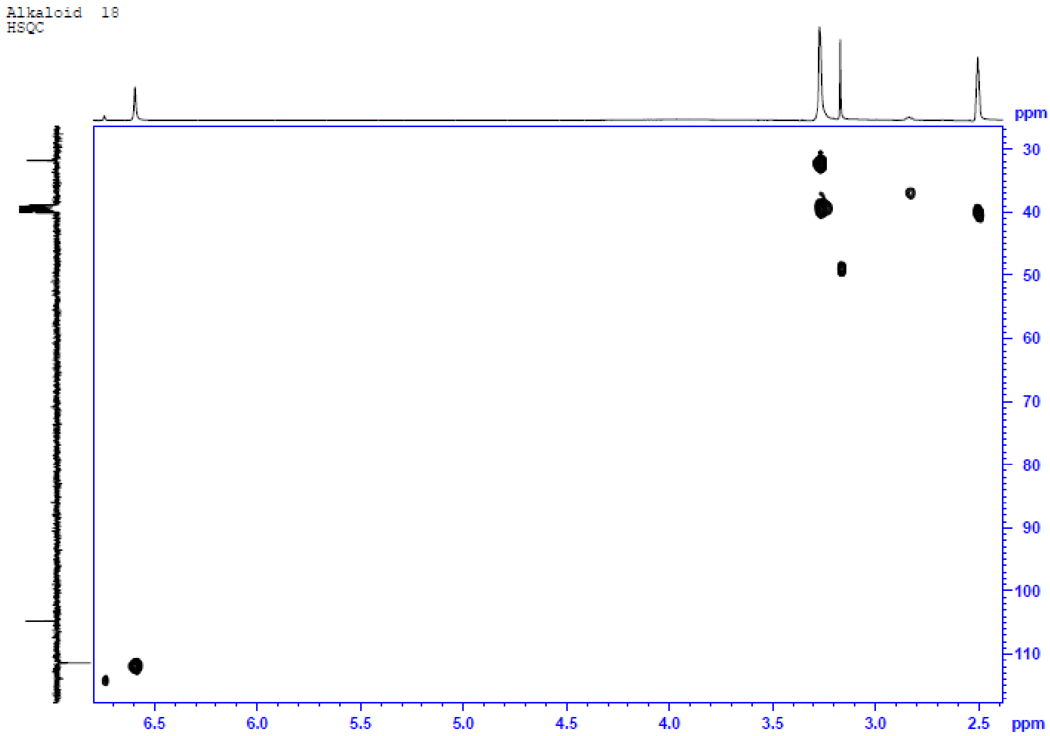

HSQC spectrum of **18** (DMSO-d_6, 400MHz)

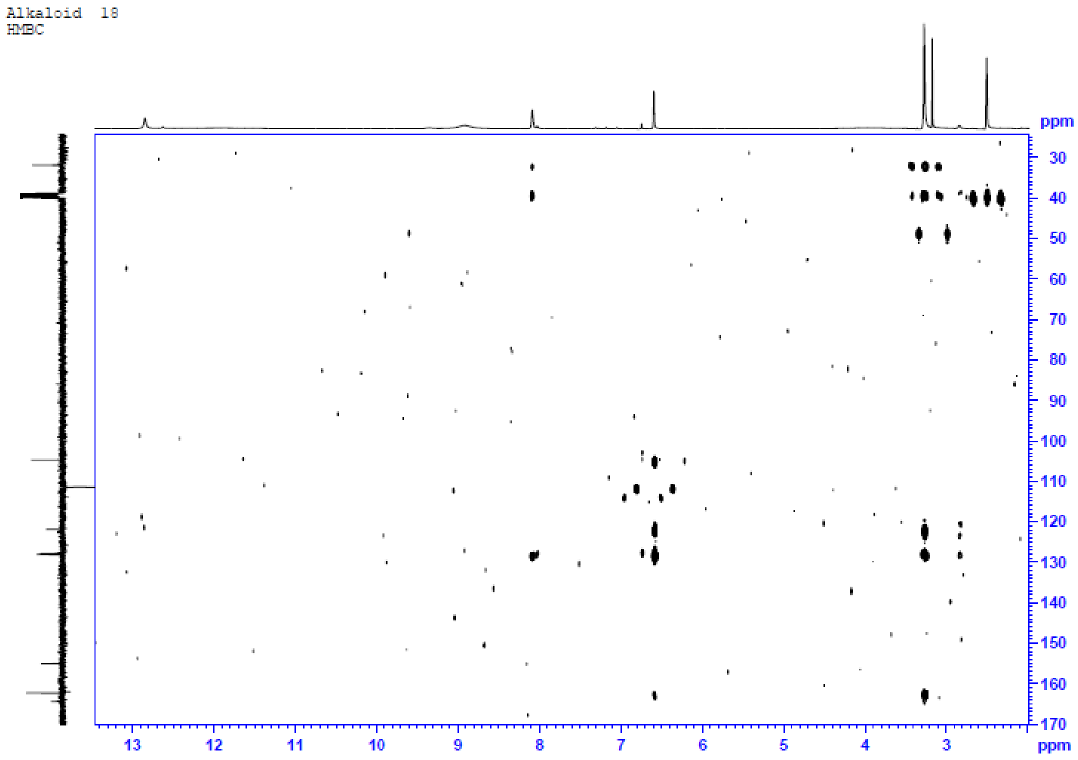

HMBC spectrum of **18** (DMSO-d_6, 400MHz)

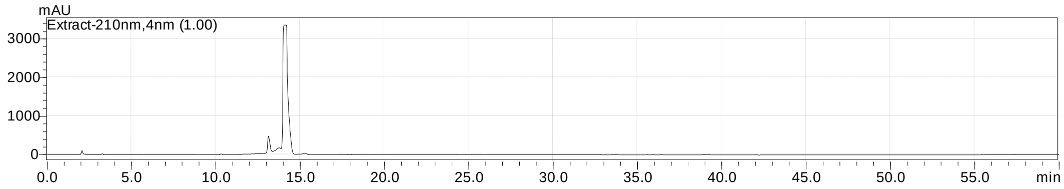

The DAD-HPLC of **19** (0-40min, 5%-100%MeOH-H_2O)

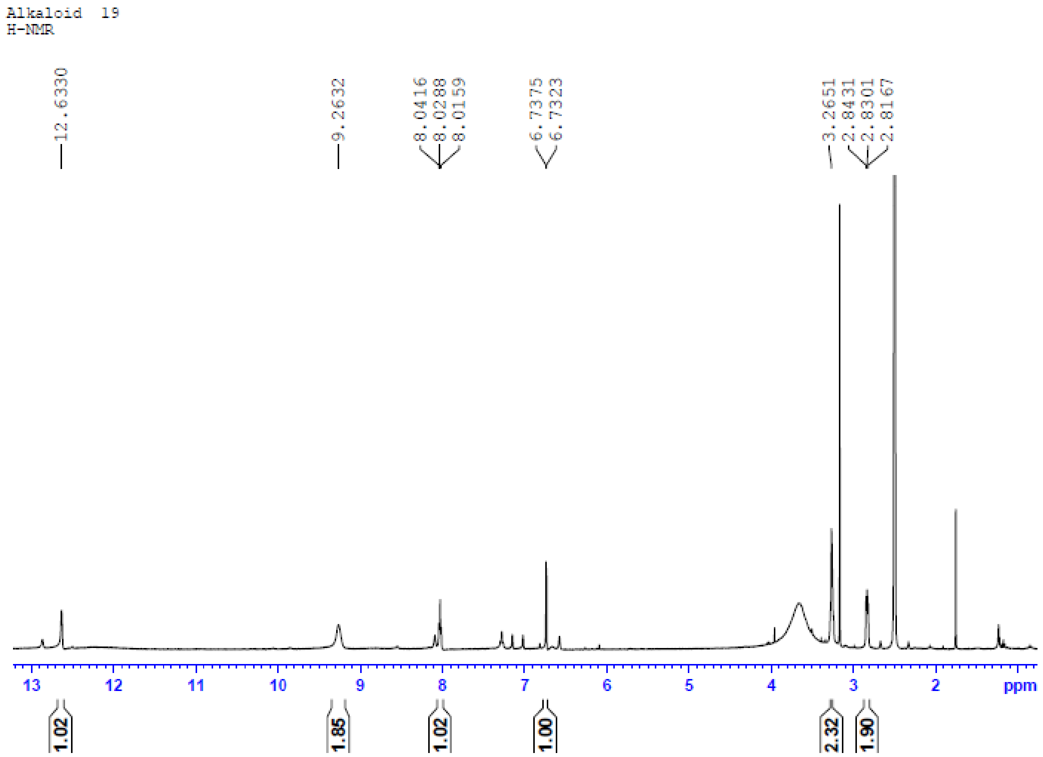

^{1}H-NMR spectrum of **19** (DMSO-d_6, 400MHz)

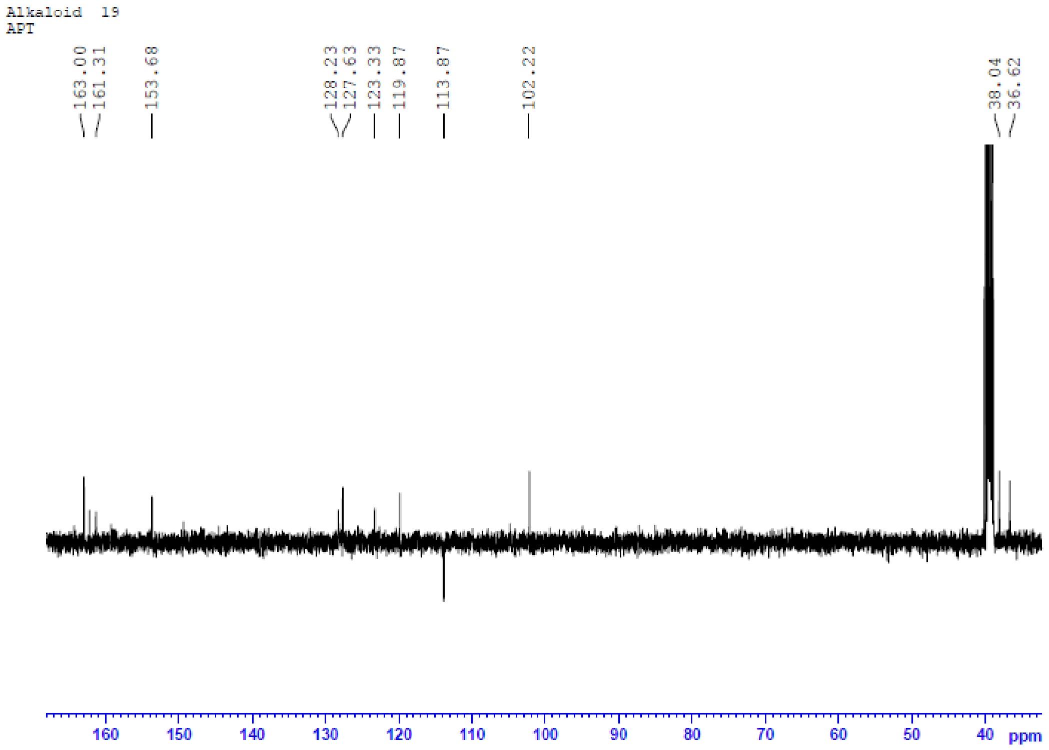

^{13}C-NMR spectrum of **19** (DMSO-d_6, 100MHz)

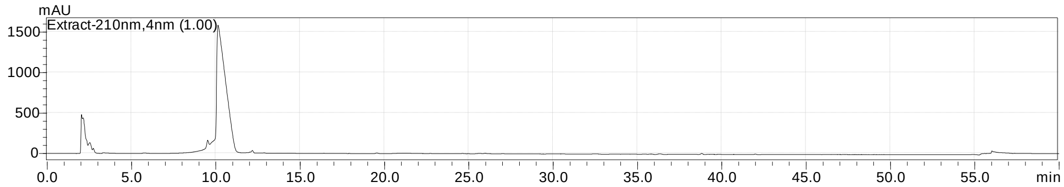

The DAD-HPLC of **20** (0-40min, 5%-100%MeOH-H_2O)

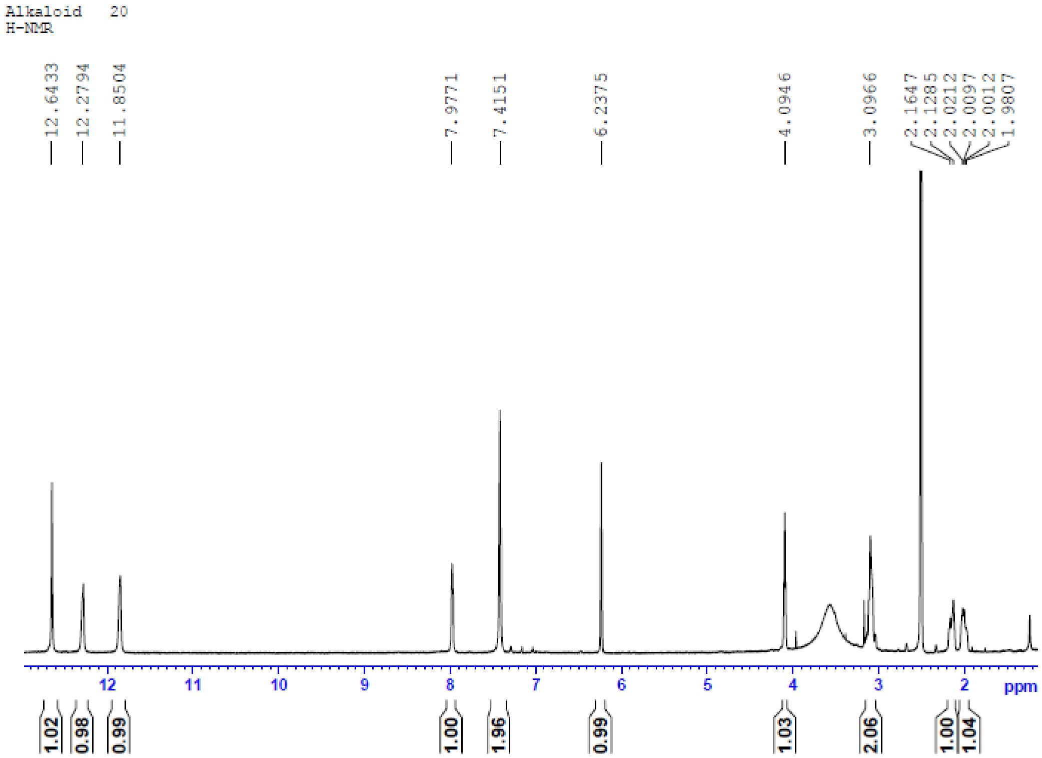

^{1}H-NMR spectrum of **20** (DMSO-d_6, 400MHz)

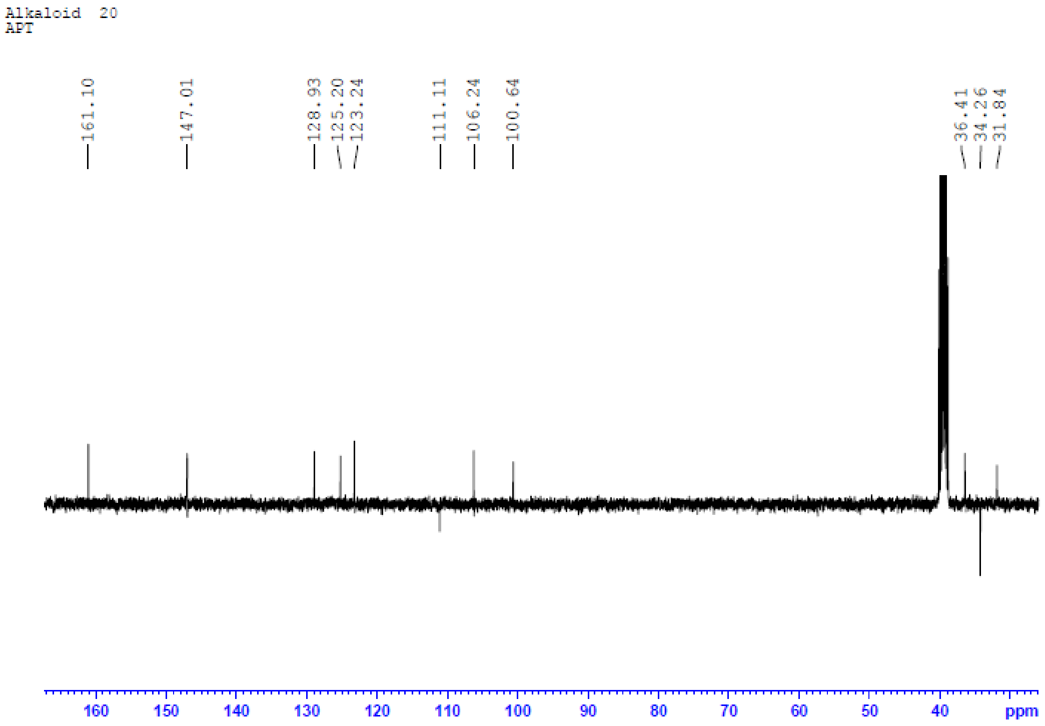

^{13}C-NMR spectrum of **20** (DMSO-d_6, 100MHz)

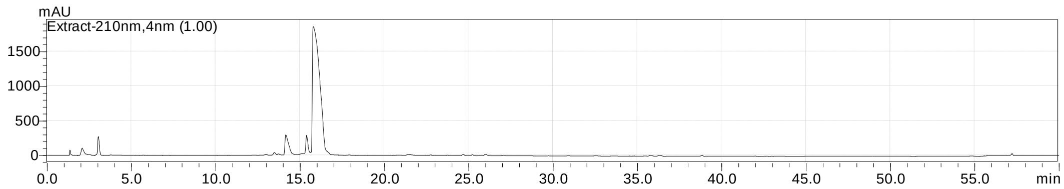

The DAD-HPLC of **21** (0-40min, 5%-100%MeOH-H_2O)

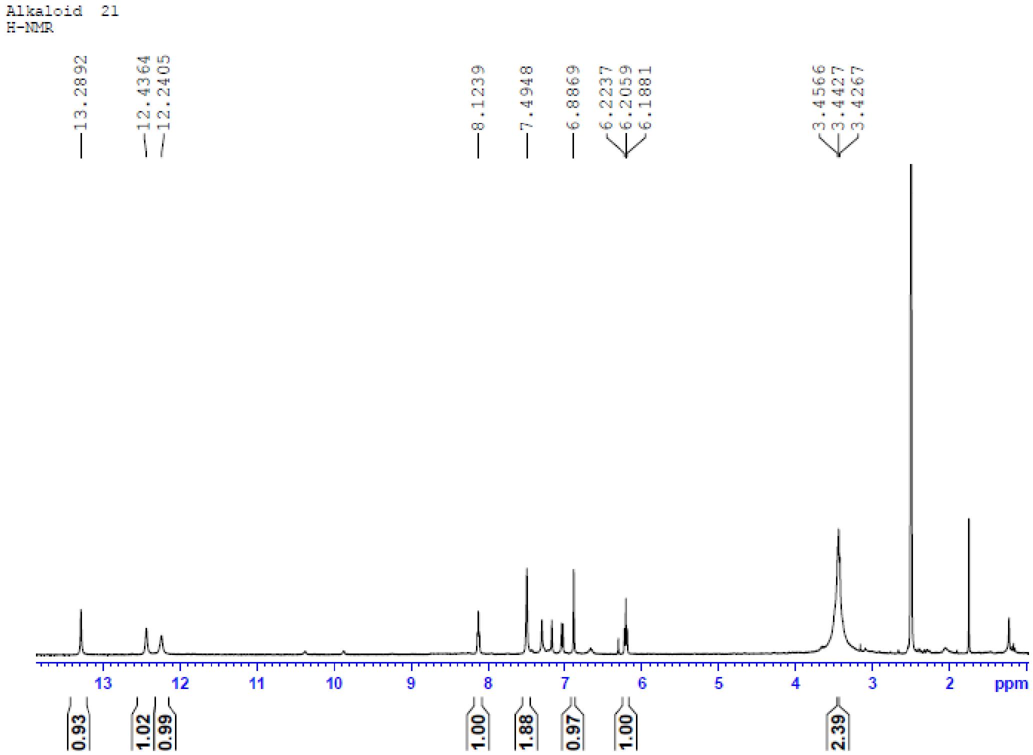

^{1}H-NMR spectrum of **21** (DMSO-d_6, 400MHz)

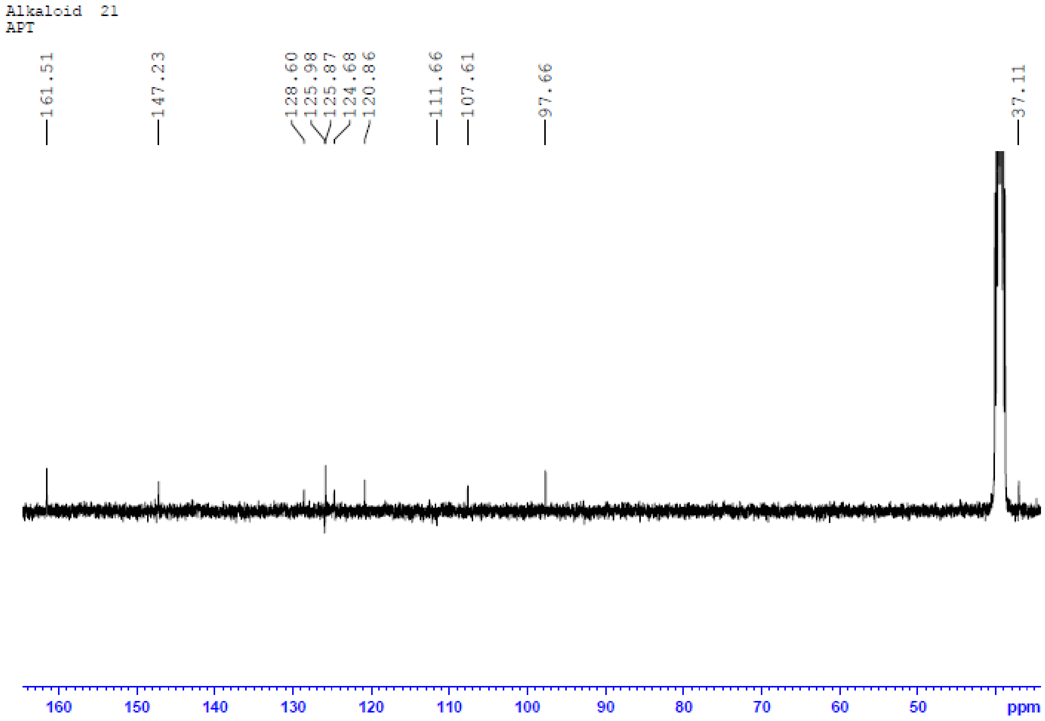

^{13}C-NMR spectrum of **21** (DMSO-d_6, 100MHz)

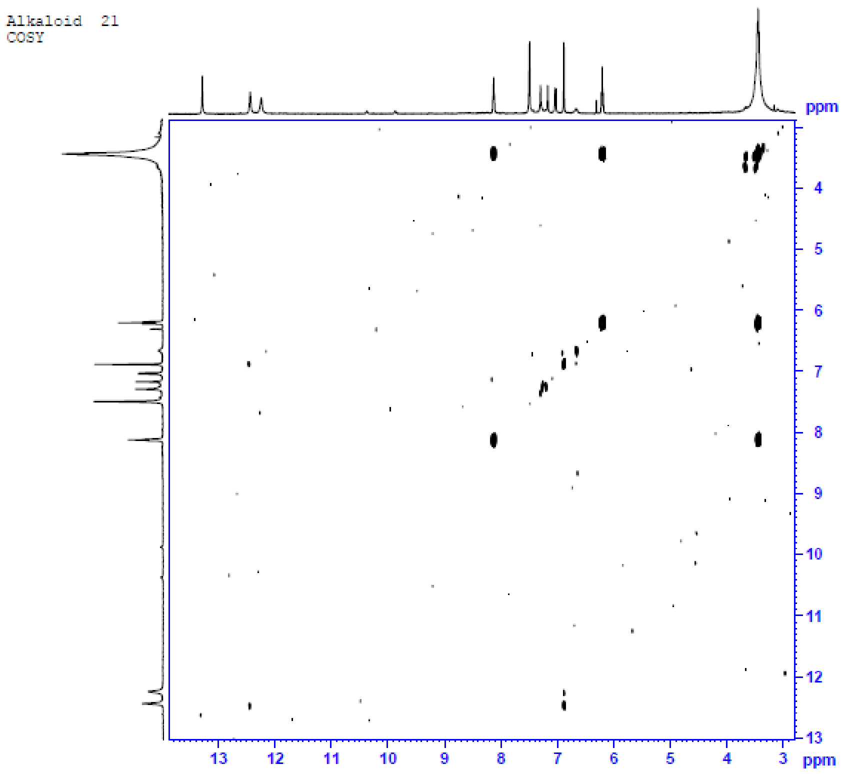

^{1}H-^{1}H COSY spectrum of **21** (DMSO-d_6, 400MHz)

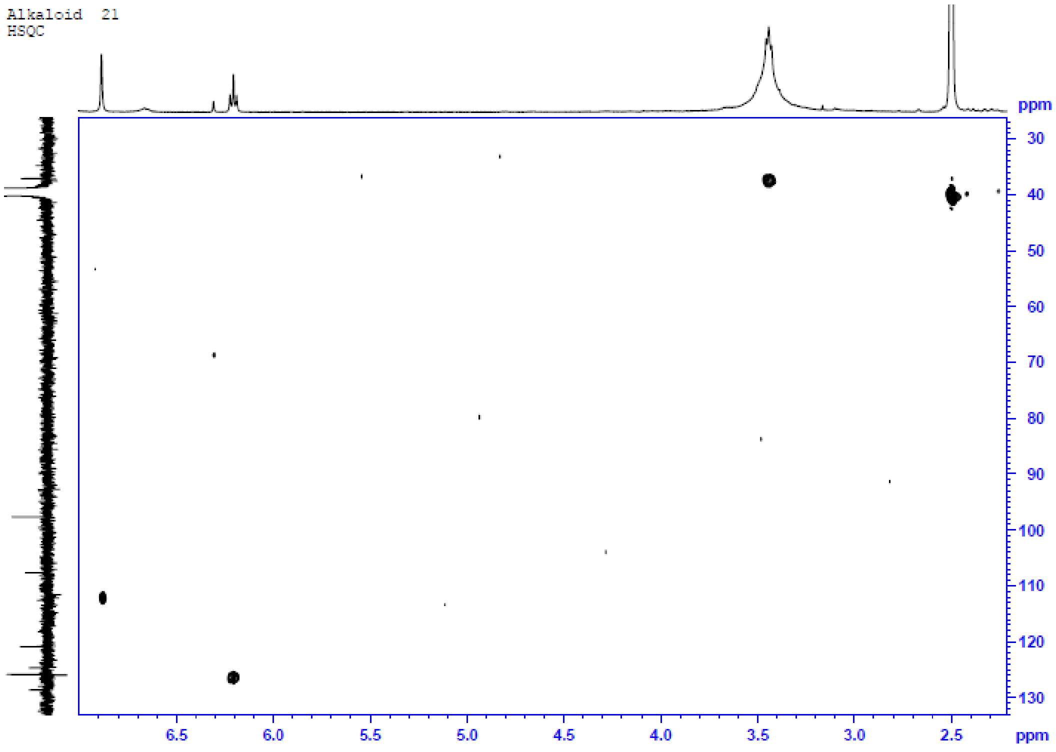

HSQC spectrum of **21** (DMSO-d_6, 400MHz)

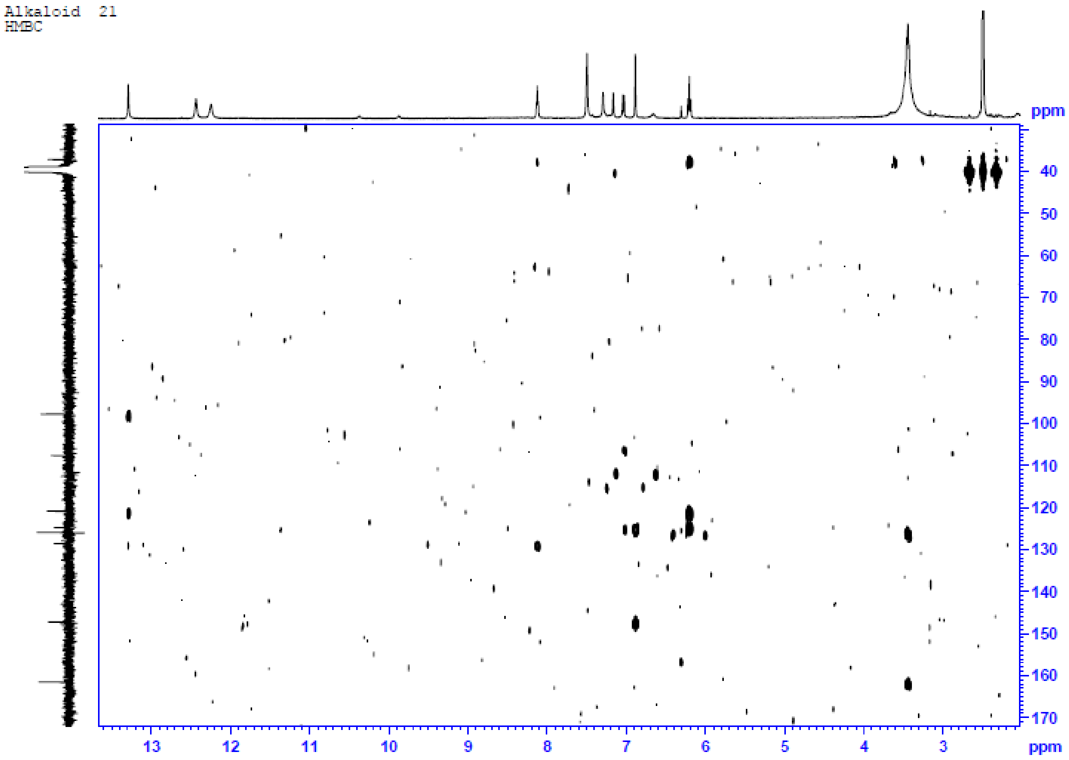

HMBC spectrum of **21** (DMSO-d_6, 400MHz)

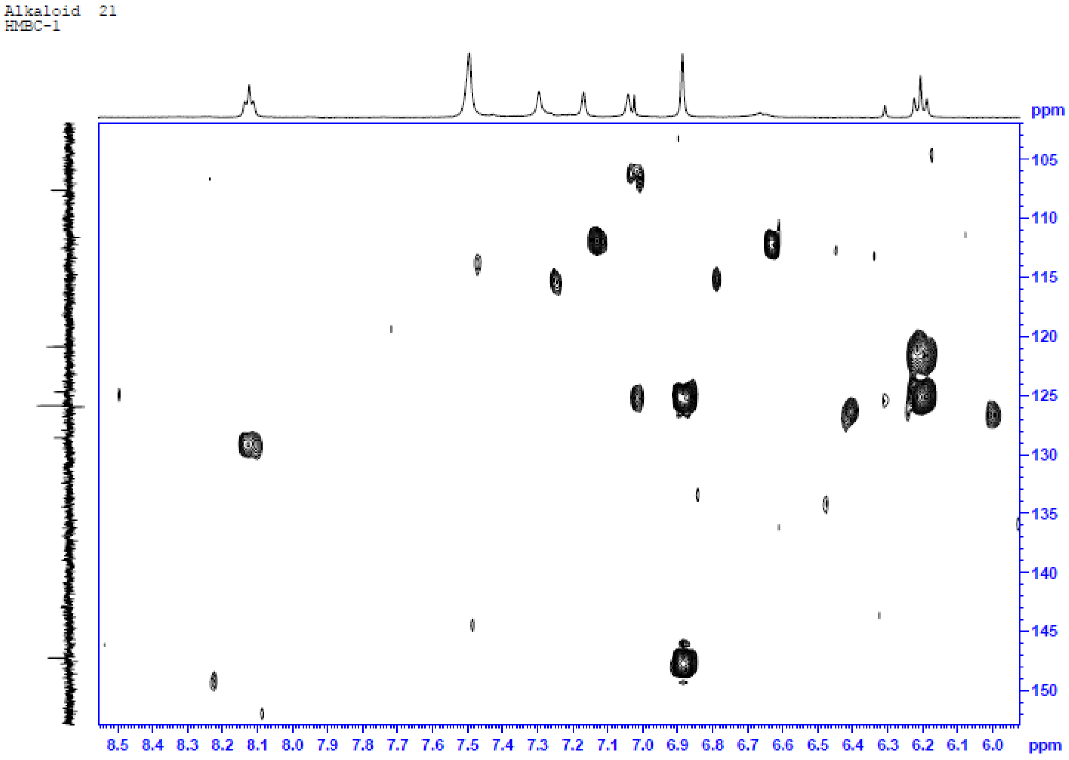

HMBC spectrum of **21** (DMSO-d_6, 400MHz), expansion-1

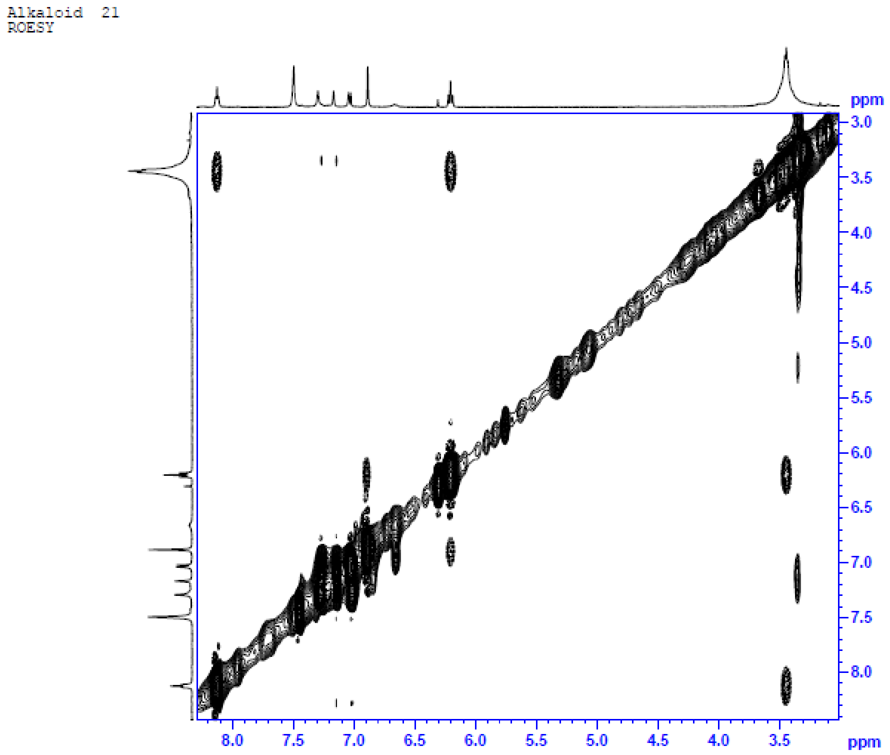

ROESY spectrum of **21** (DMSO-d_6, 400MHz)

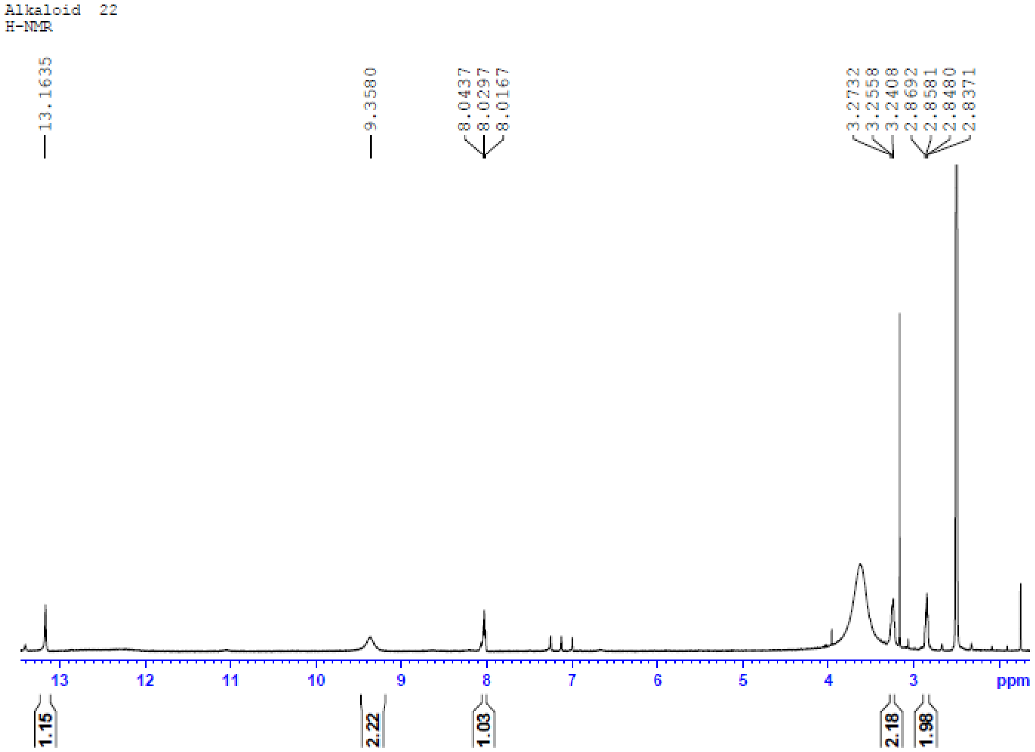

^{1}H-NMR spectrum of **22** (DMSO-d_6, 400MHz)

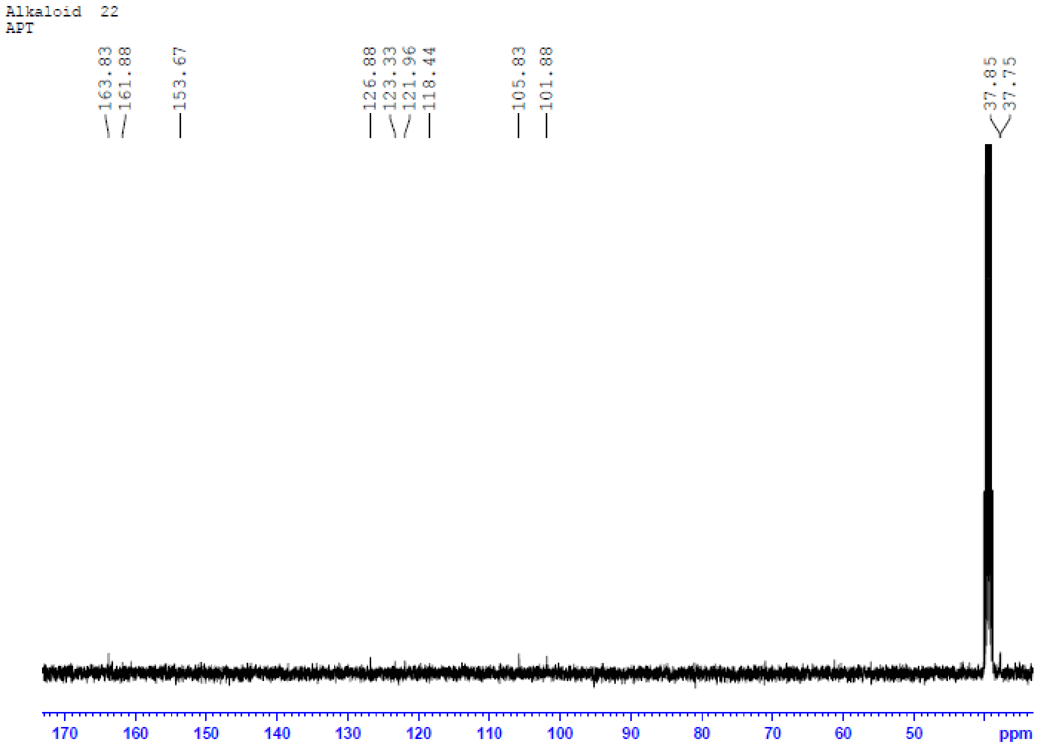

^{13}C-NMR spectrum of **22** (DMSO-d_6, 100MHz)

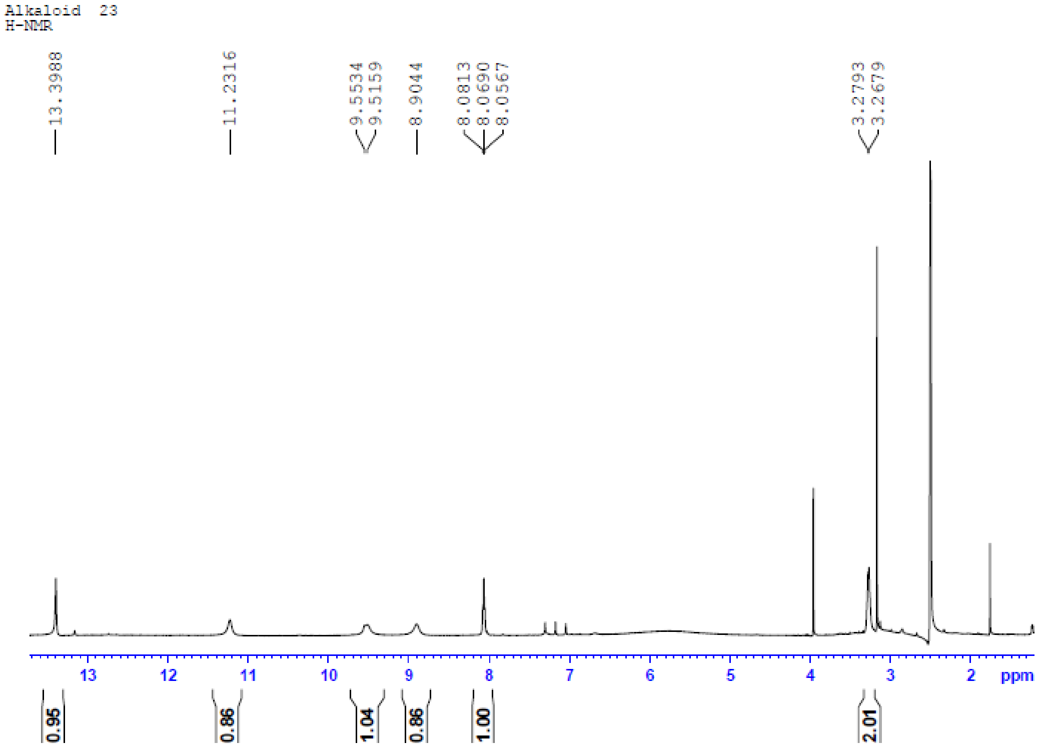

^{1}H-NMR spectrum of **23** (DMSO-d_6, 400MHz)

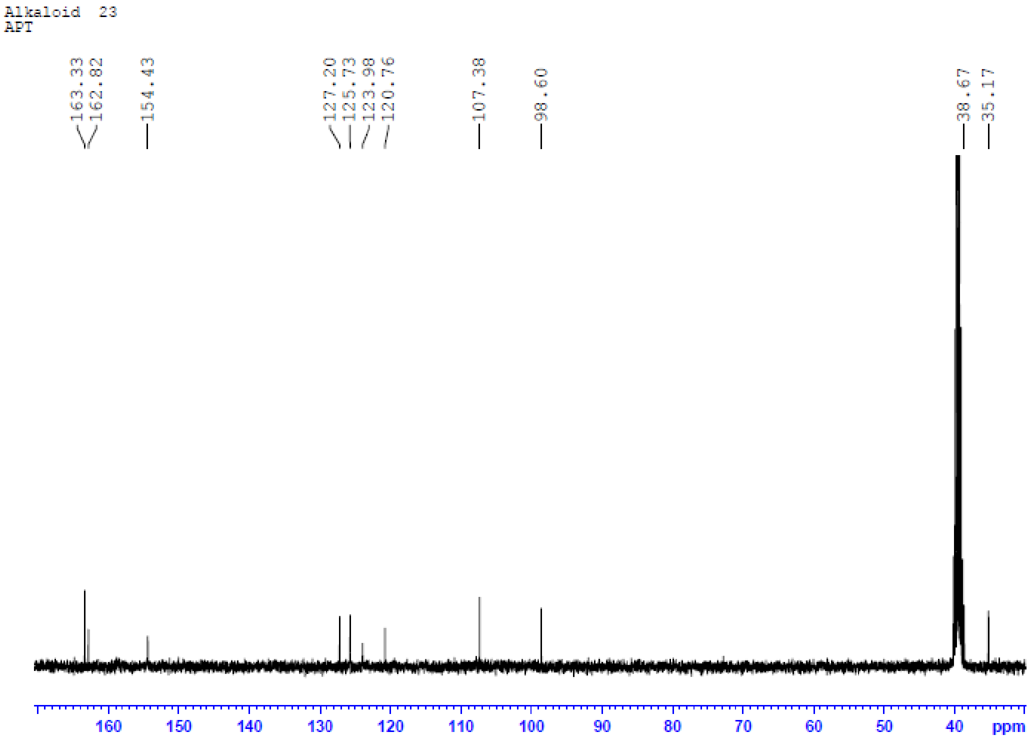

^{13}C-NMR spectrum of **23** (DMSO-d_6, 100MHz)

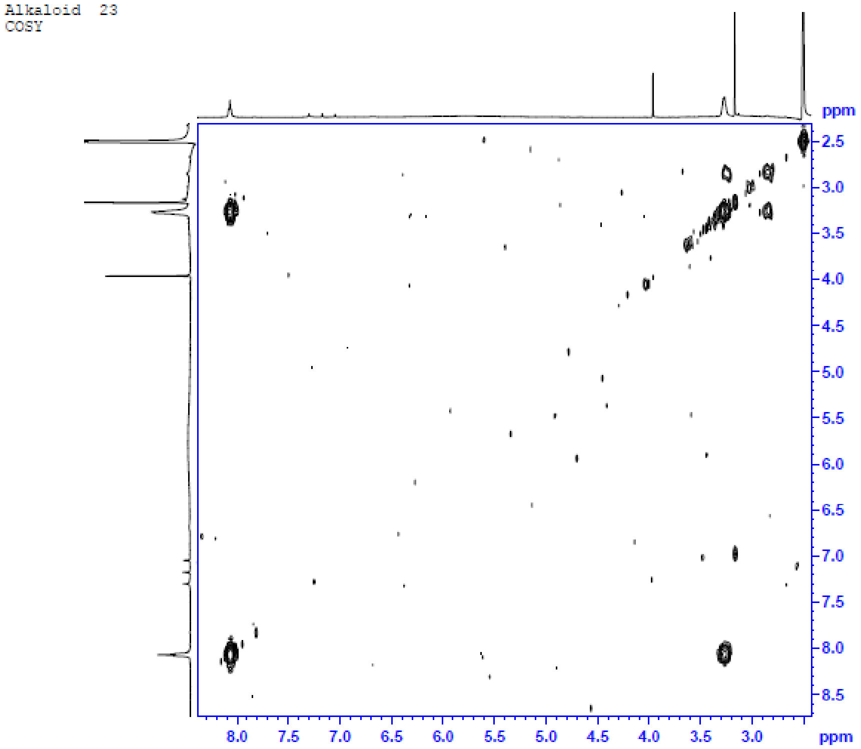

^{1}H-^{1}H COSY spectrum of **23** (DMSO-d_6, 400MHz)

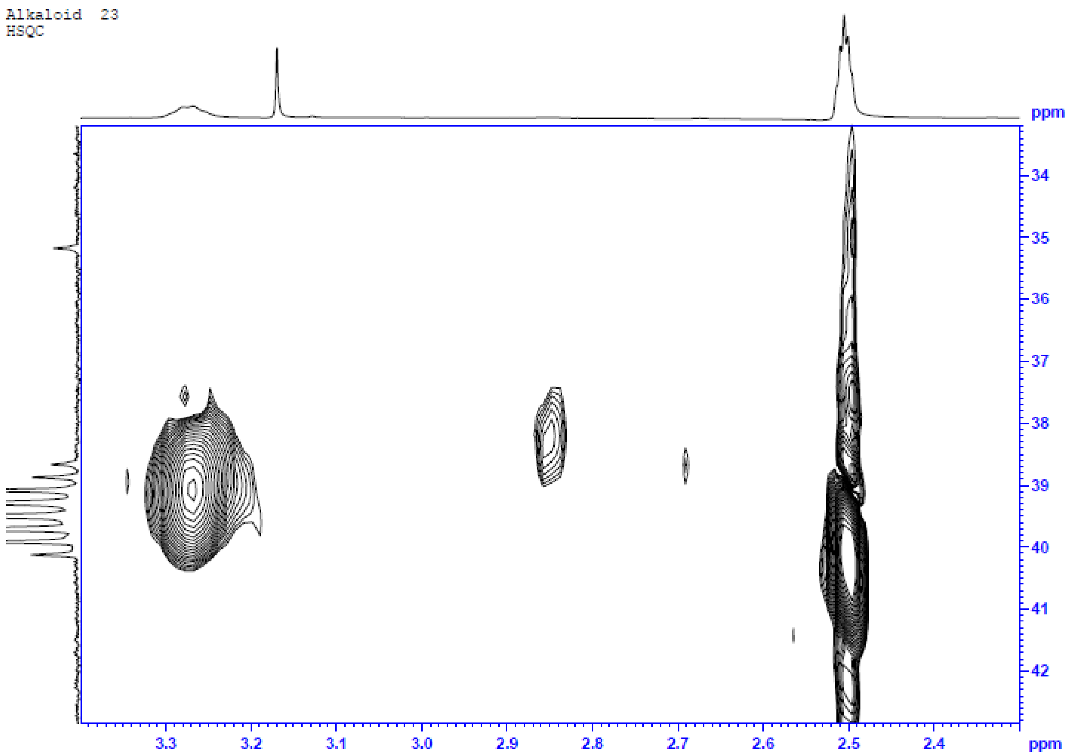

HSQC spectrum of **23** (DMSO-d_6, 400MHz)

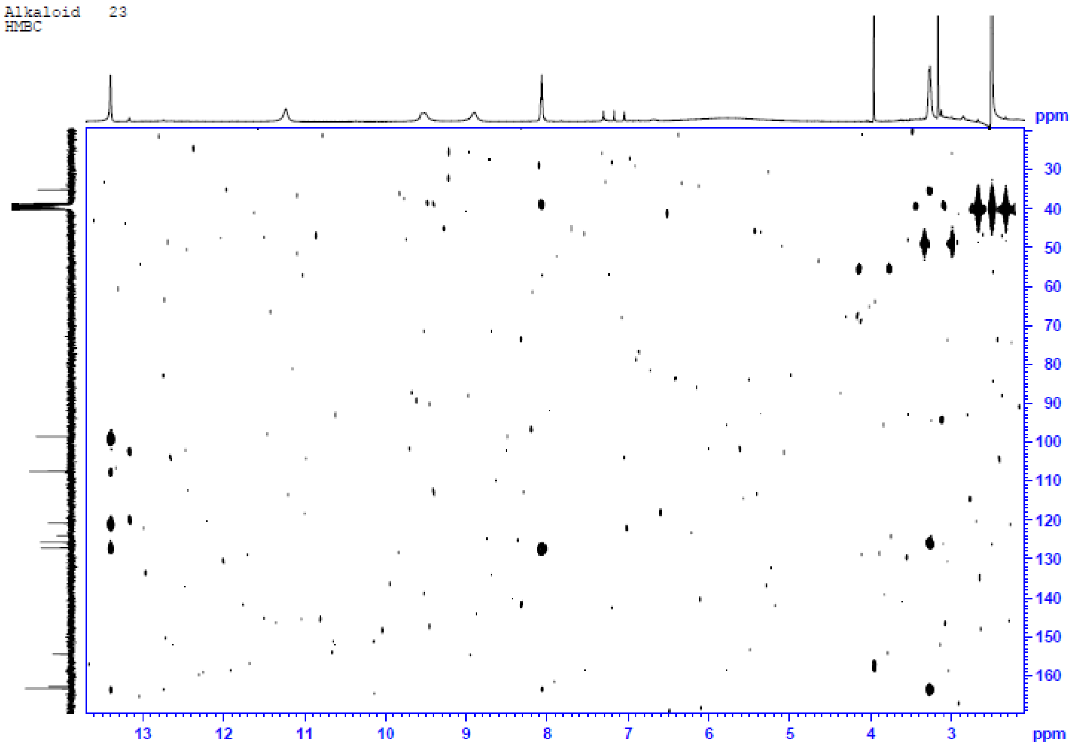

HMBC spectrum of **23** (DMSO-d_6, 400MHz)

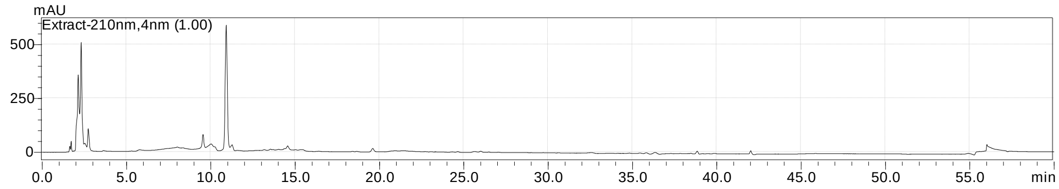

The DAD-HPLC of **24** (0-40min, 5%-100%MeOH-H_2O)

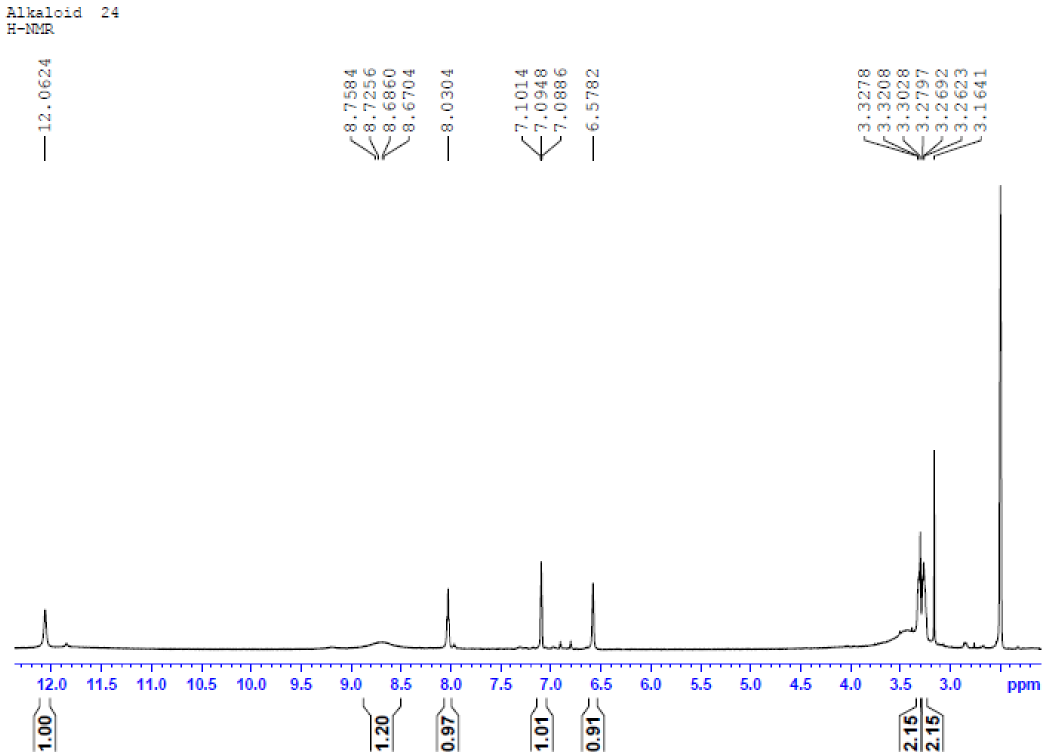

^{1}H-NMR spectrum of **24** (DMSO-d_6, 400MHz)

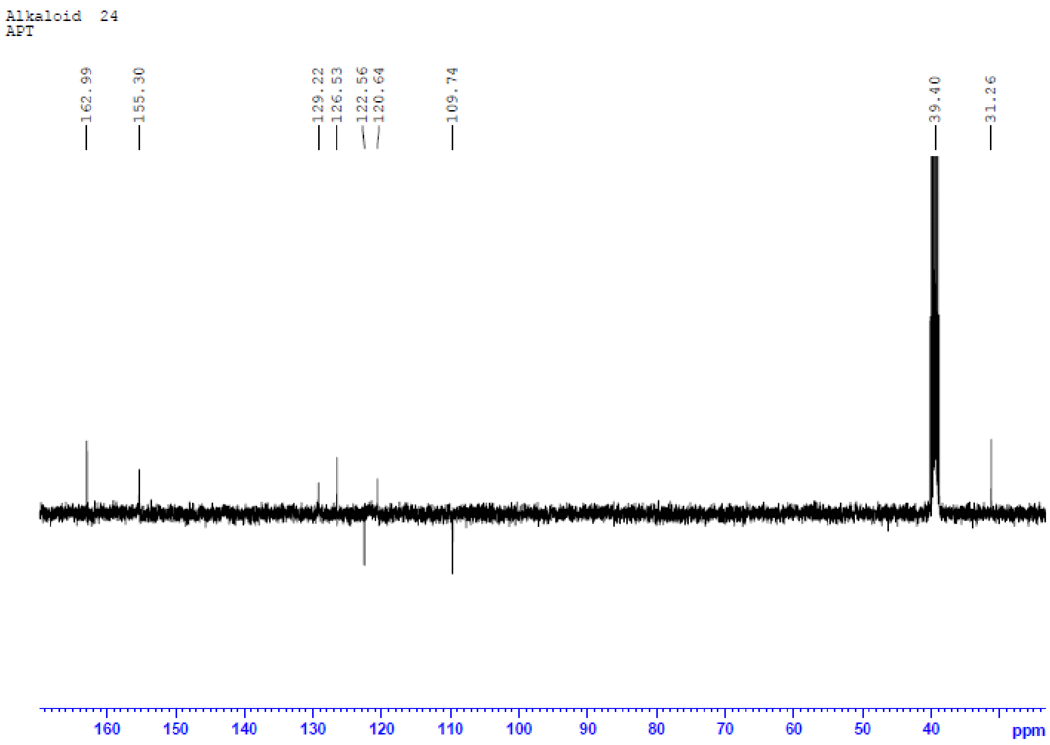

^{13}C-NMR spectrum of **24** (DMSO-d_6, 100MHz)

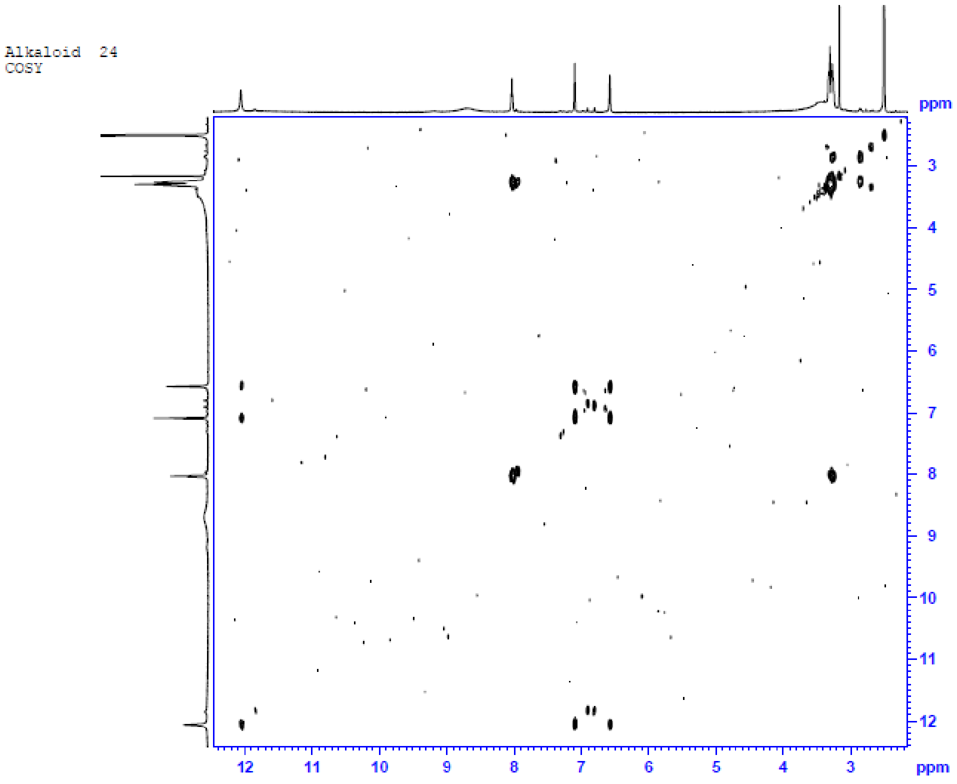

^{1}H-^{1}H COSY spectrum of **24** (DMSO-d_6, 400MHz)

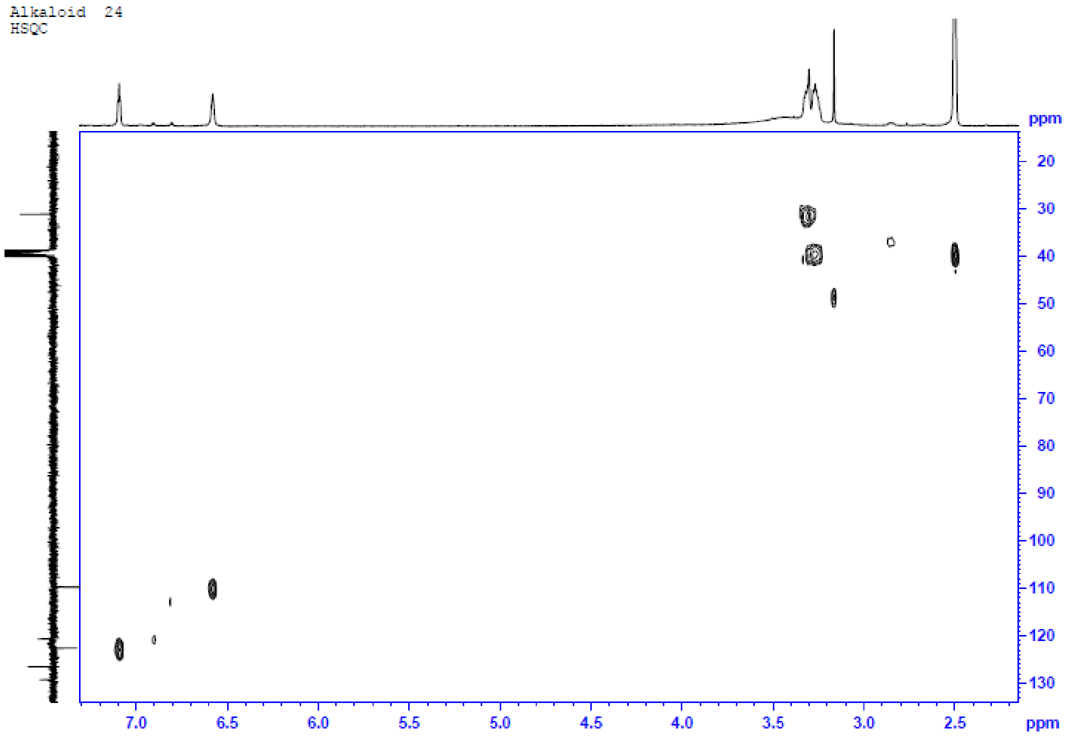

HSQC spectrum of **24** (DMSO-d_6, 400MHz)

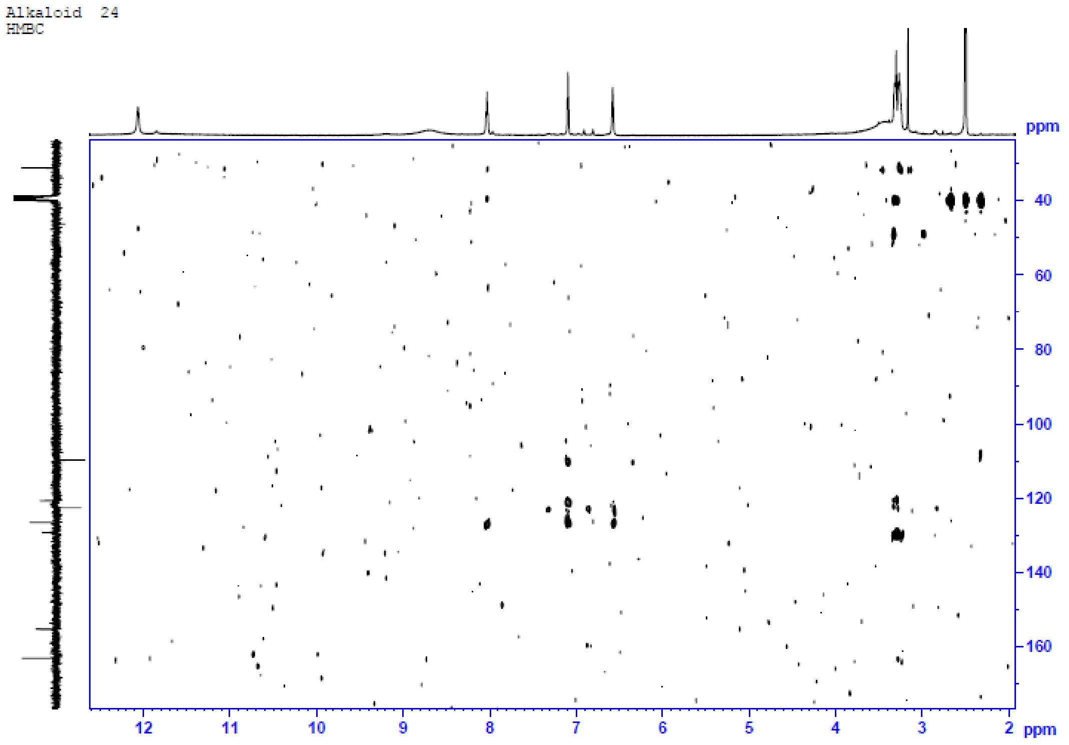

HMBC spectrum of **24** (DMSO-d_6, 400MHz)

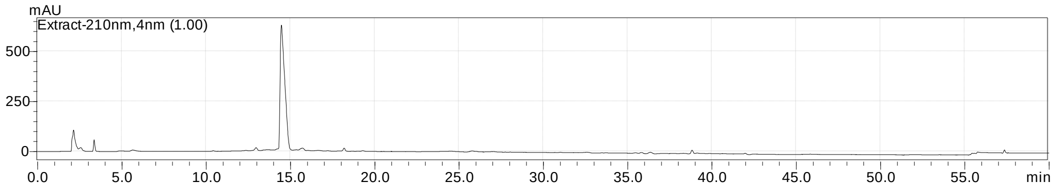

The DAD-HPLC of **25** (0-40min, 5%-100%MeOH-H_2O)

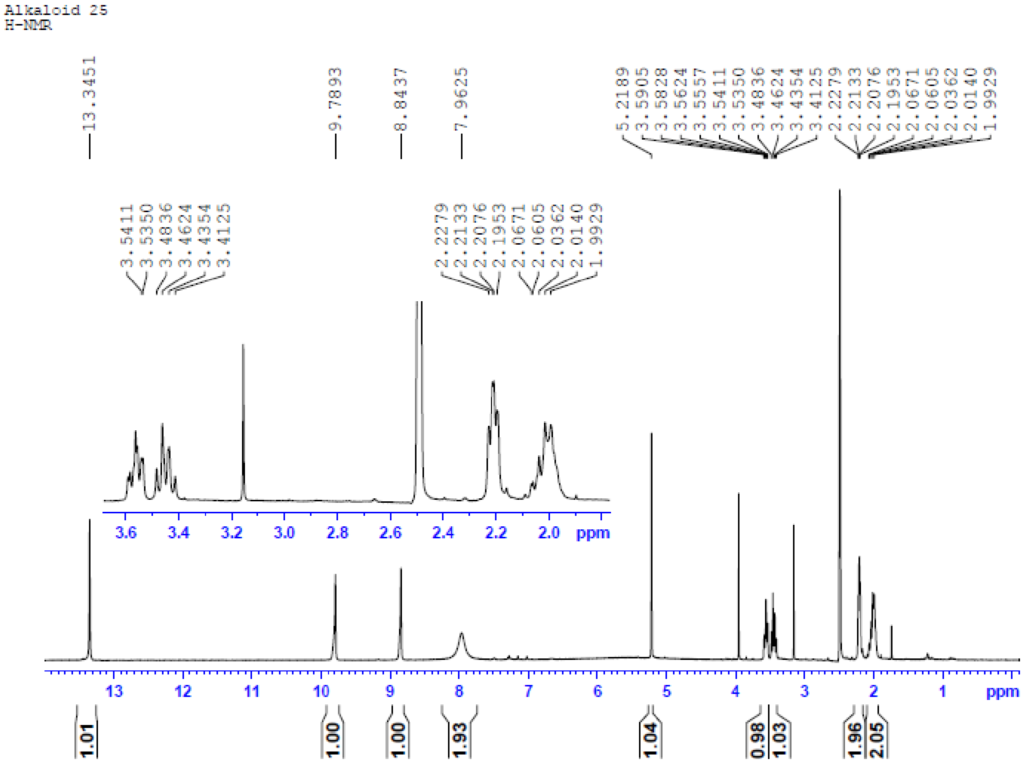

^{1}H-NMR spectrum of **25** (DMSO-d_6, 400MHz)

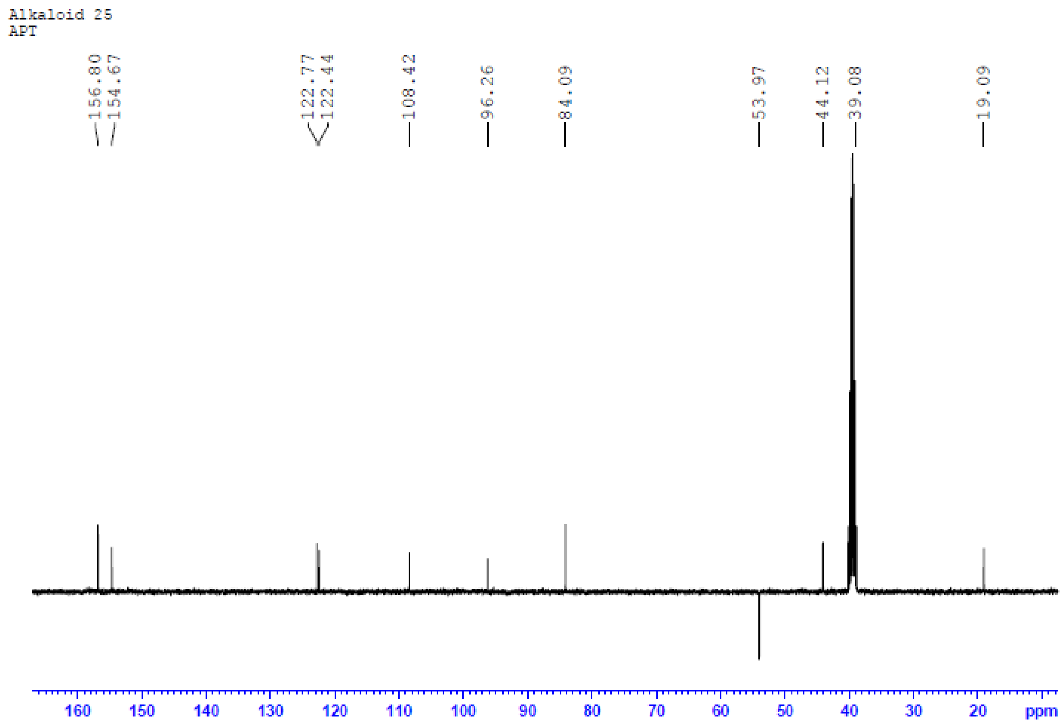

^{13}C-NMR spectrum of **25** (DMSO-d_6, 100MHz)

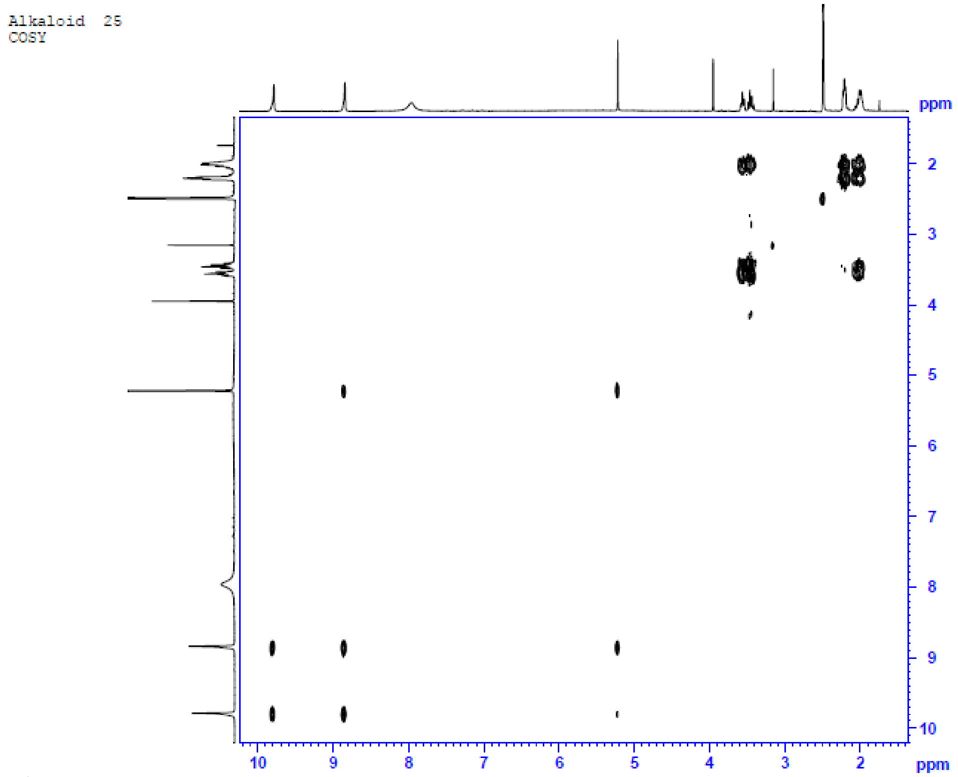

^{1}H-^{1}H COSY spectrum of **25** (DMSO-d_6, 400MHz)

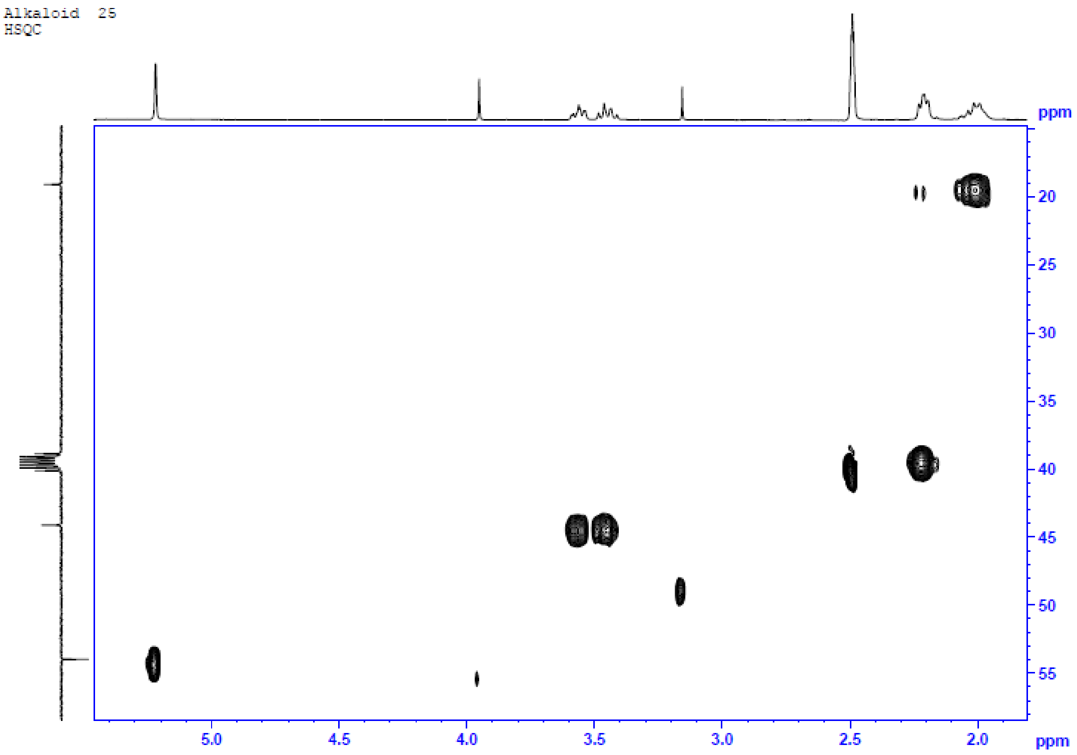

HSQC spectrum of **25** (DMSO-d_6, 400MHz)

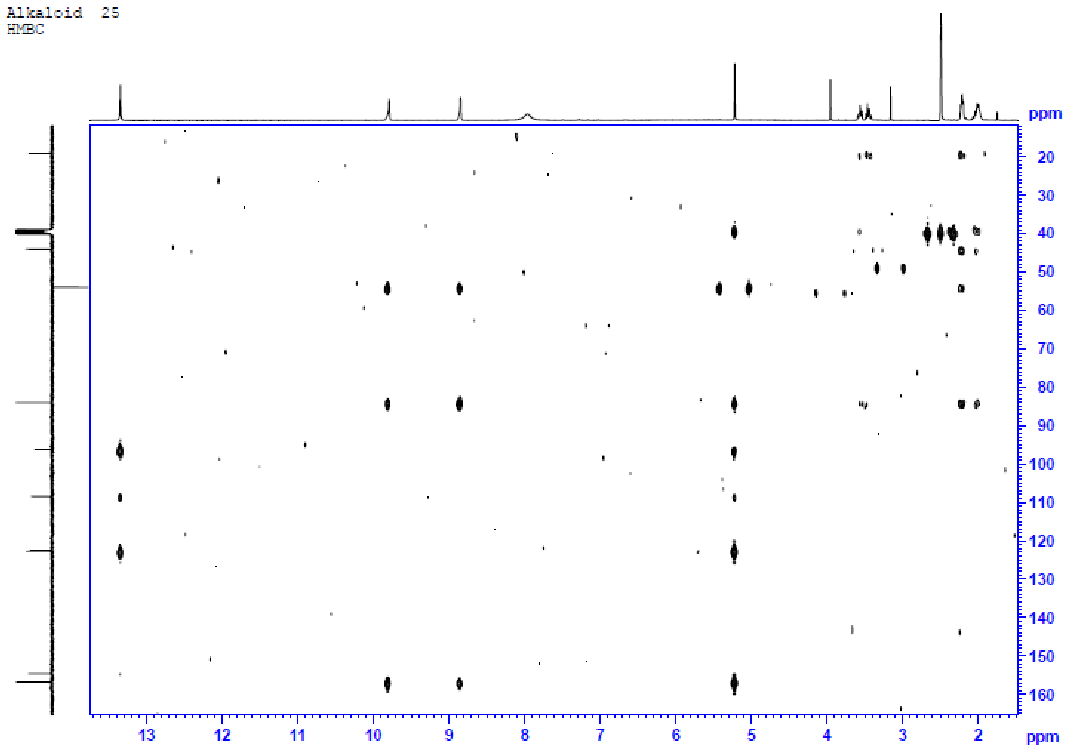

HMBC spectrum of **25** (DMSO-d_6, 400MHz)

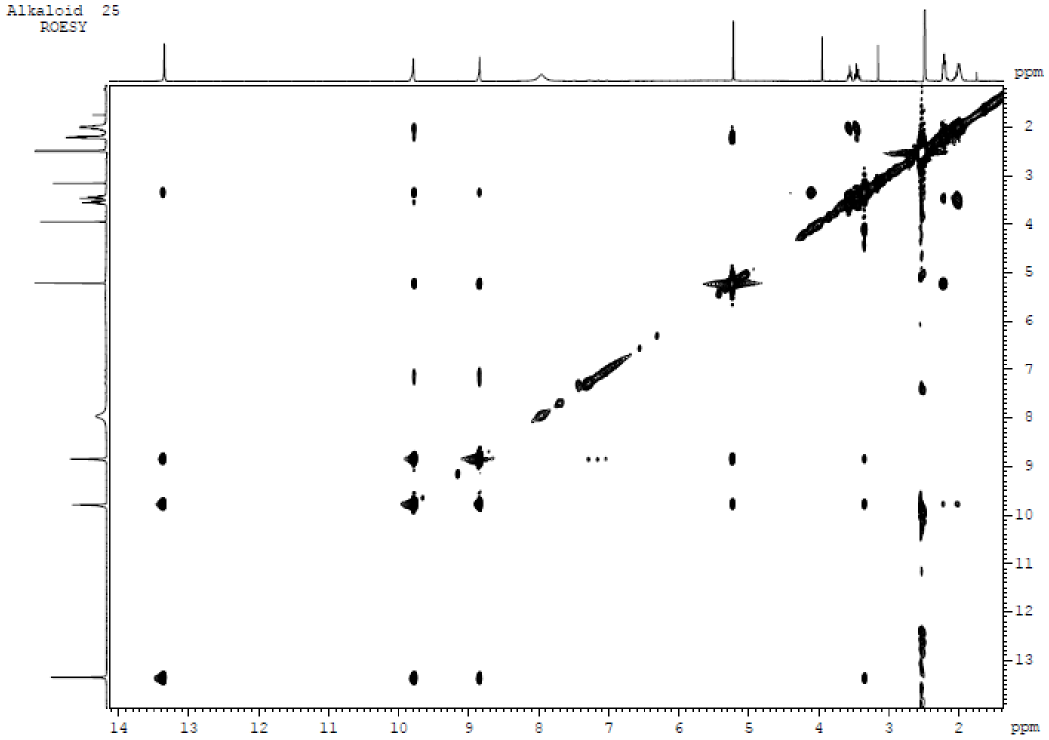

ROESY spectrum of **25** (DMSO-d_6, 400MHz)

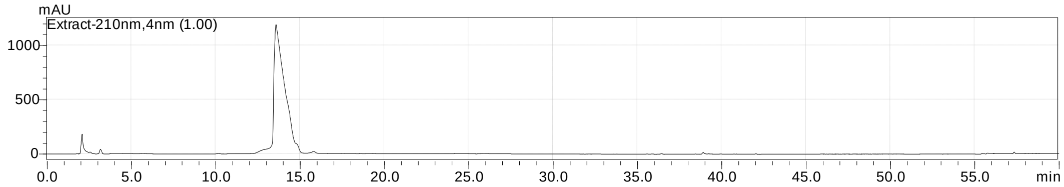

The DAD-HPLC of **26** (0-40min, 5%-100%MeOH-H_2O)

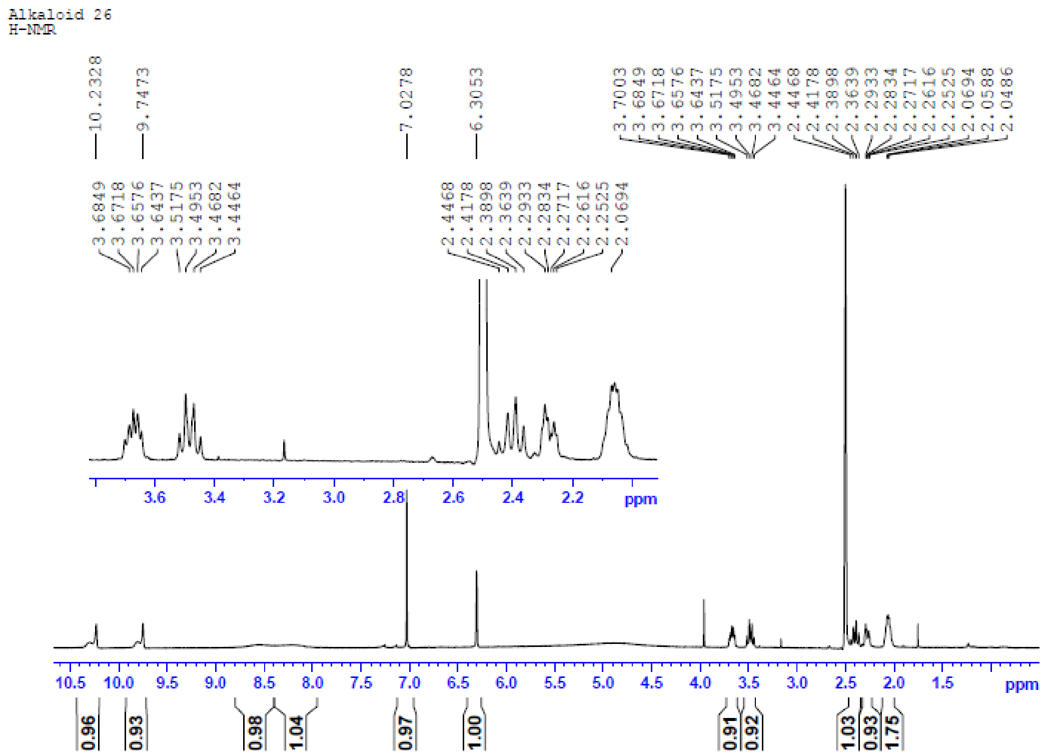

^{1}H-NMR spectrum of **26** (DMSO-d_6, 400MHz)

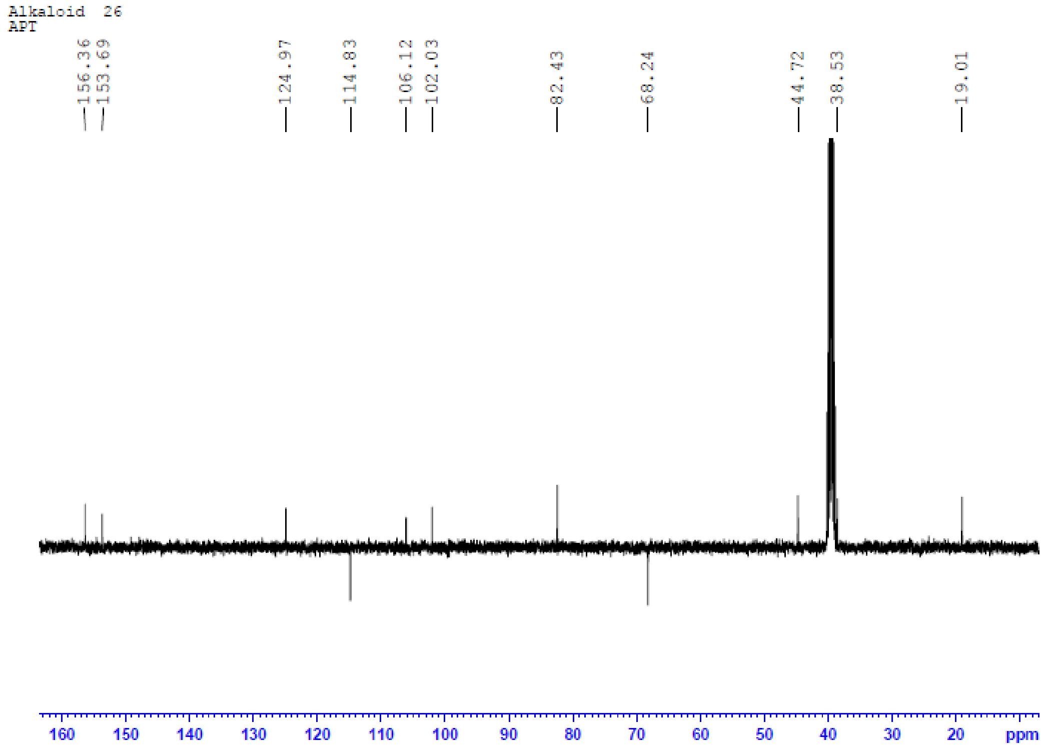

^{13}C-NMR spectrum of **26** (DMSO-d_6, 100MHz)

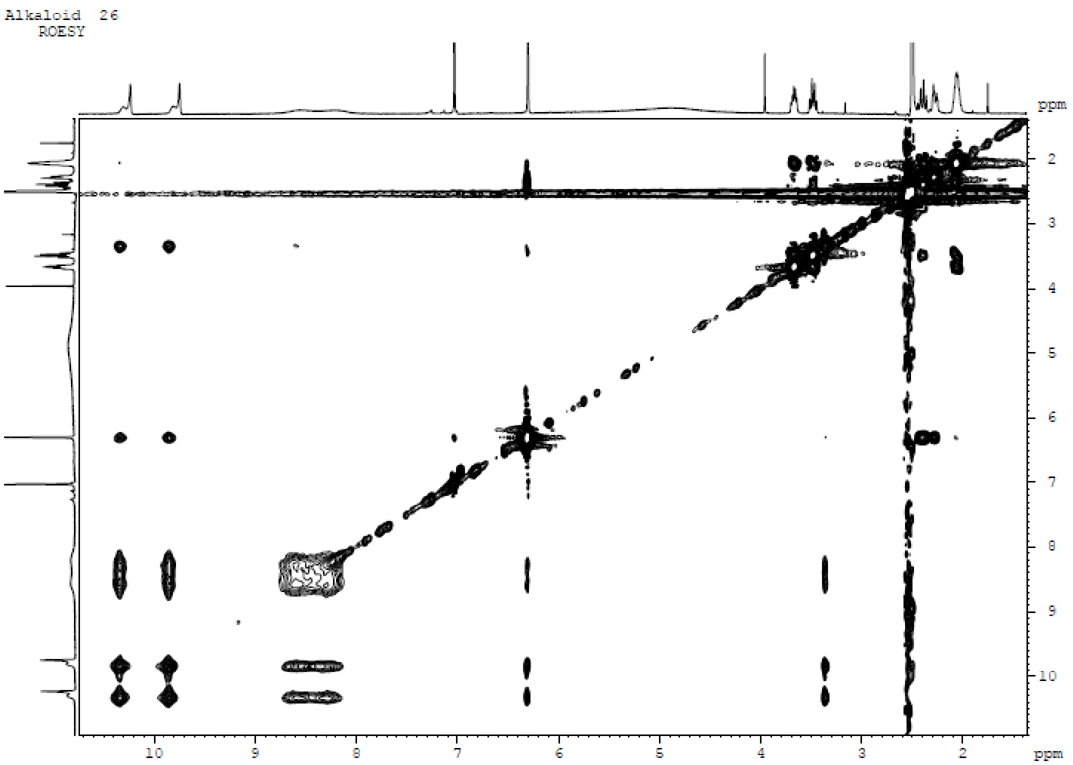

ROESY spectrum of **26** (DMSO-d_6, 400MHz)

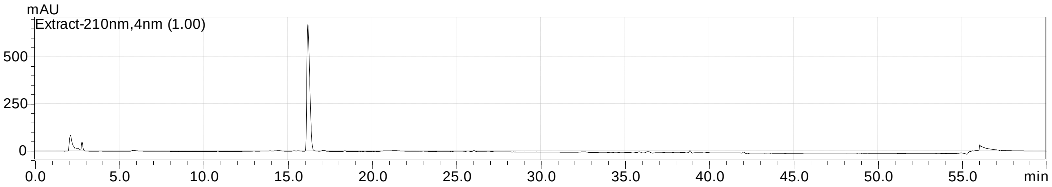

The DAD-HPLC of **27** (0-40min, 5%-100%MeOH-H_2O)

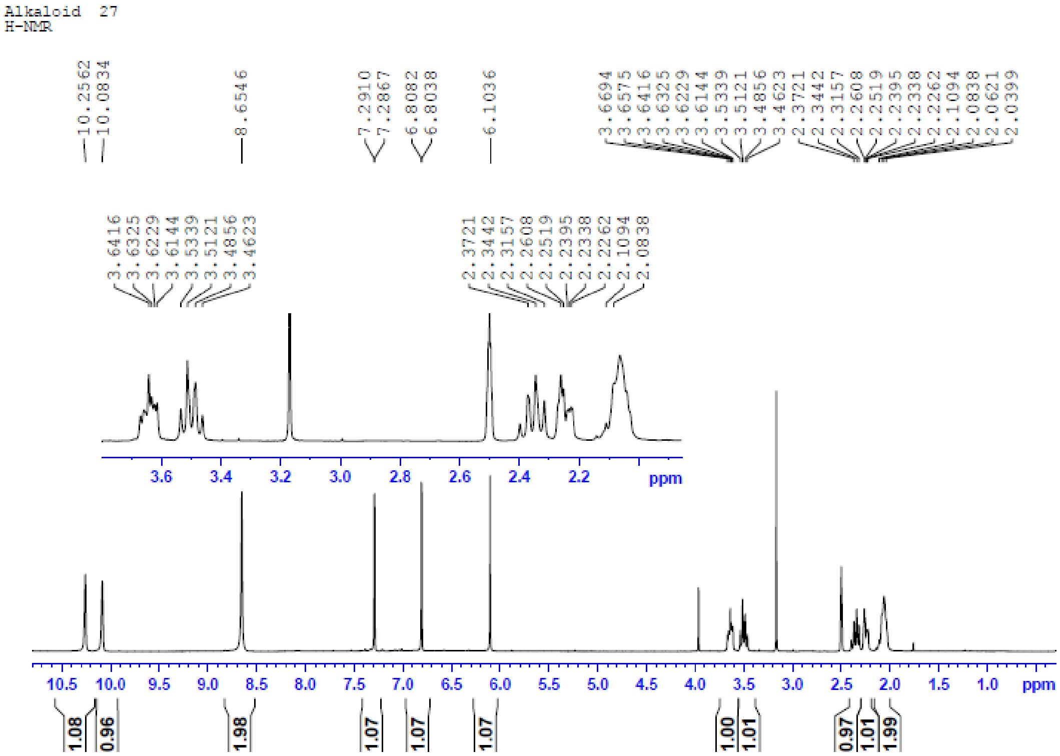

^{1}H-NMR spectrum of **27** (DMSO-d_6, 400MHz)

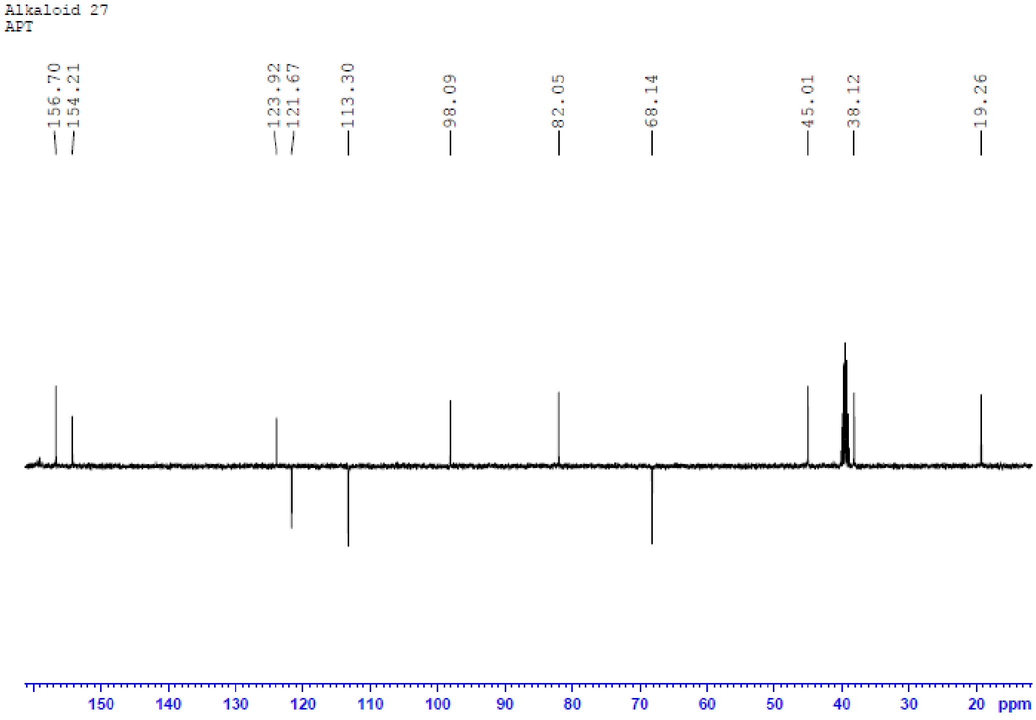

^{13}C-NMR spectrum of **27** (DMSO-d_6, 100MHz)

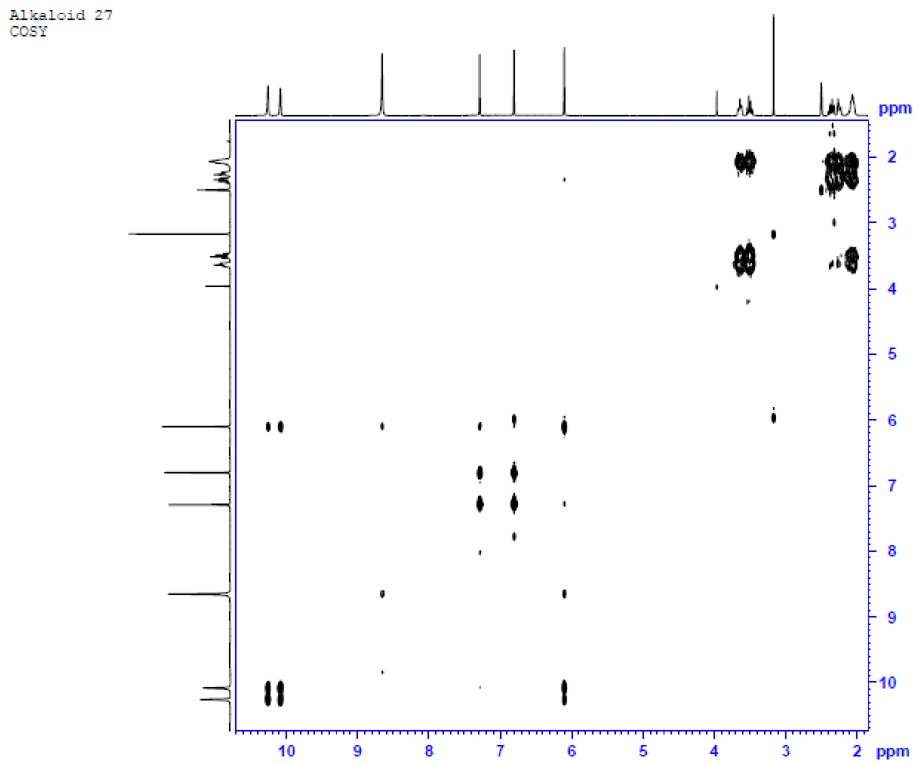

^{1}H-^{1}H COSY spectrum of **27** (DMSO-d_6, 400MHz)

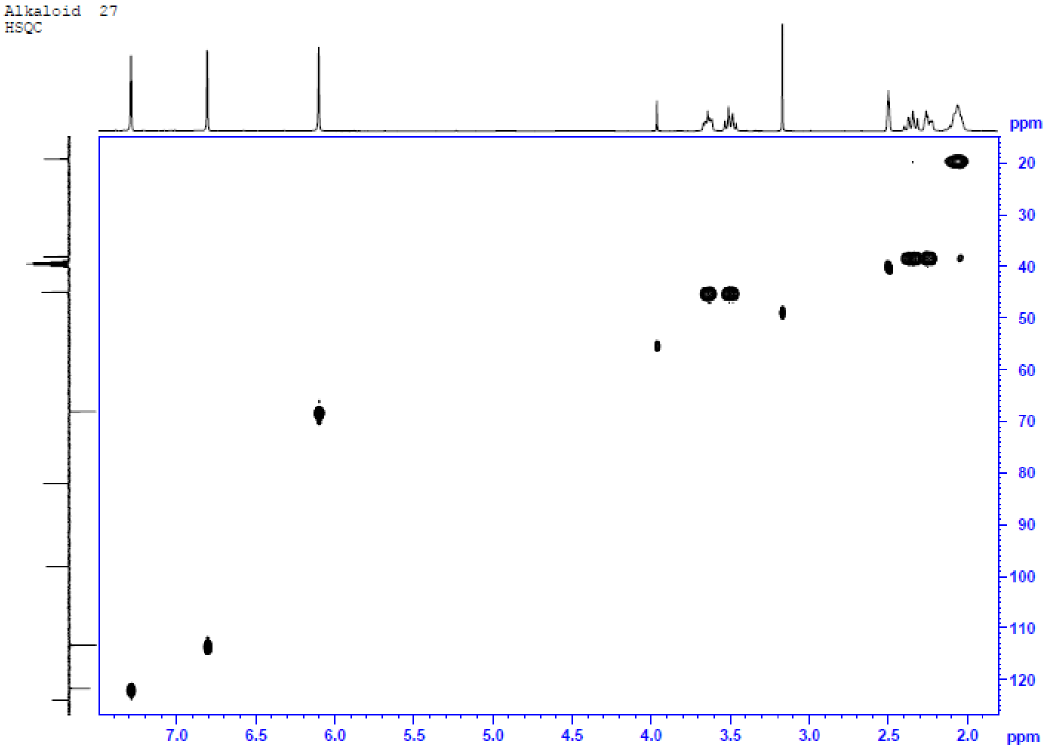

HSQC spectrum of **27** (DMSO-d_6, 400MHz)

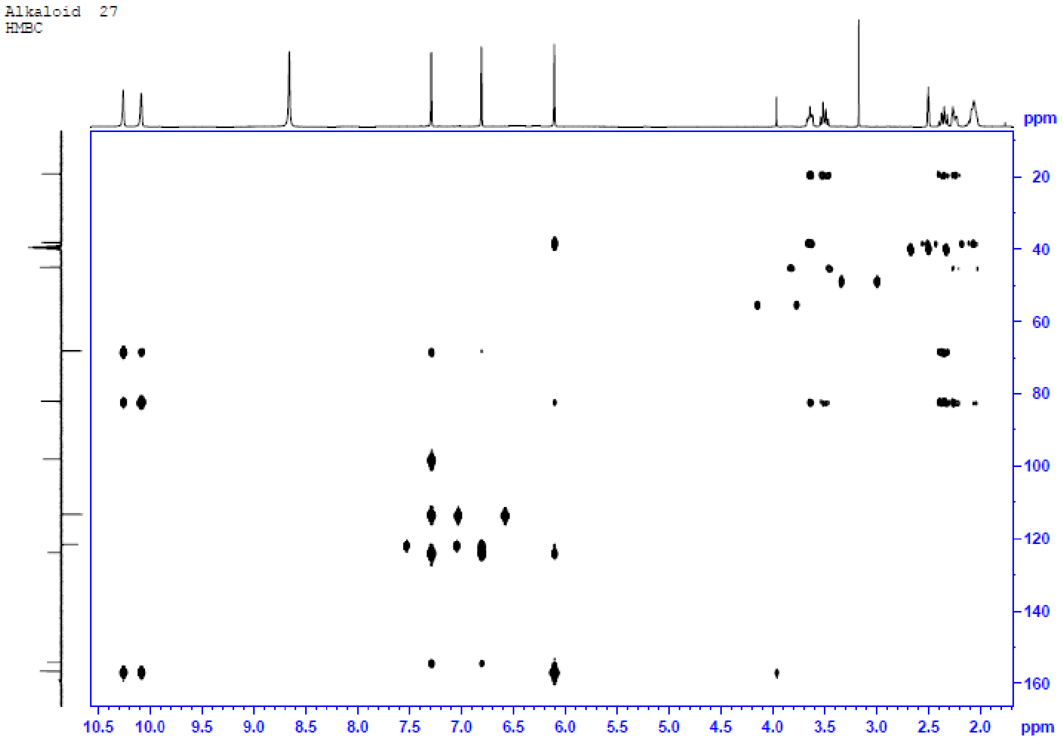

HMBC spectrum of **27** (DMSO-d_6, 400MHz)

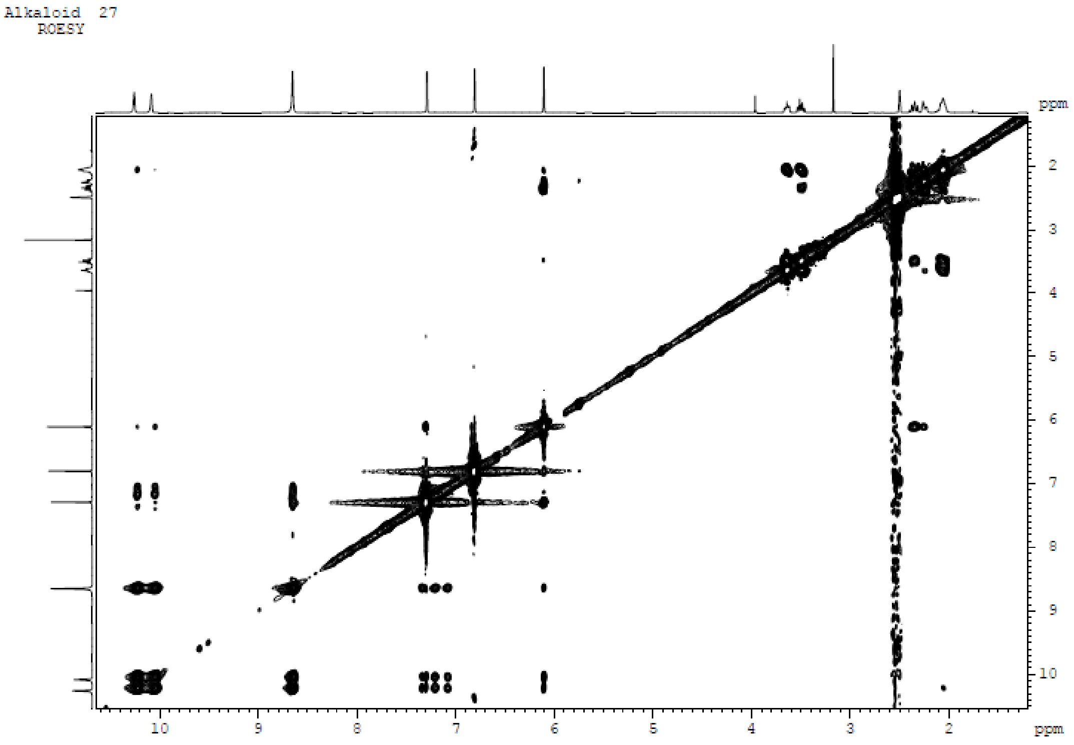

ROESY spectrum of **27** (DMSO-d_6, 400MHz)

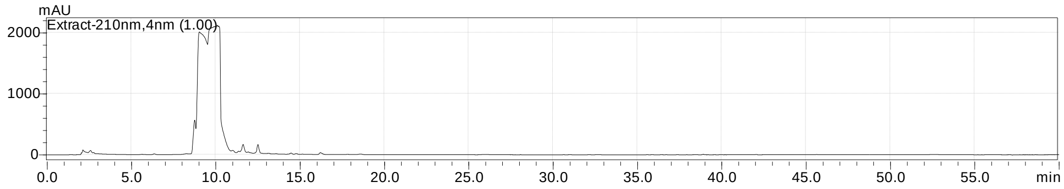

The DAD-HPLC of **28** (0-40min, 5%-100%MeOH-H_2O)

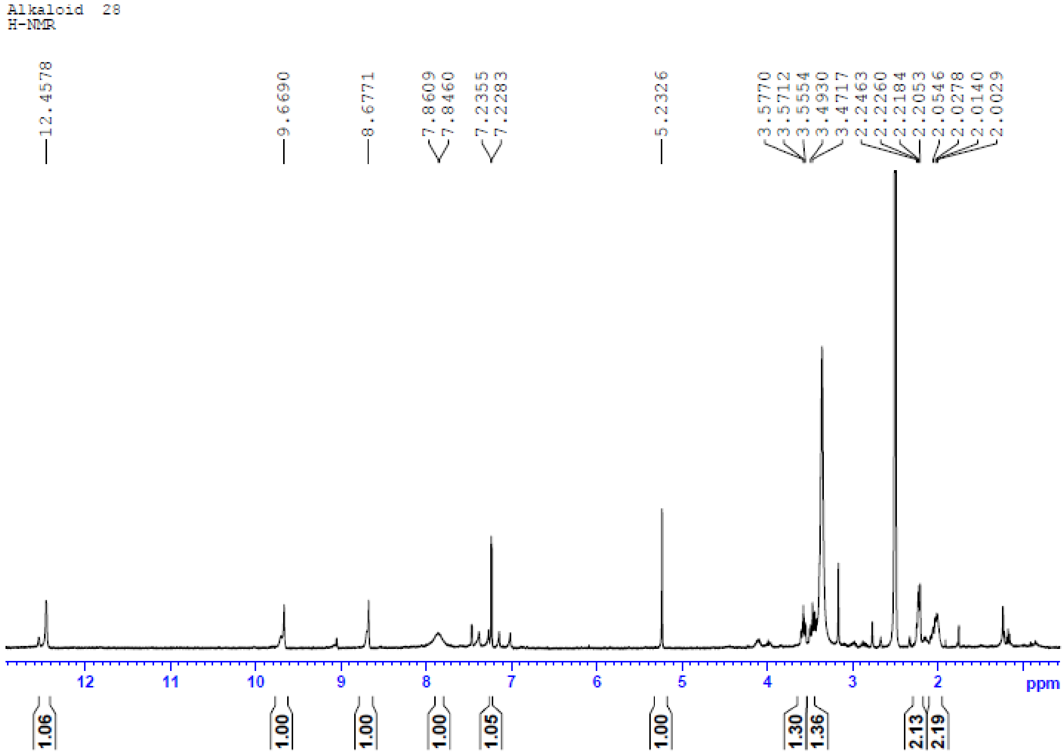

^{1}H-NMR spectrum of **28** (DMSO-d_6, 400MHz)

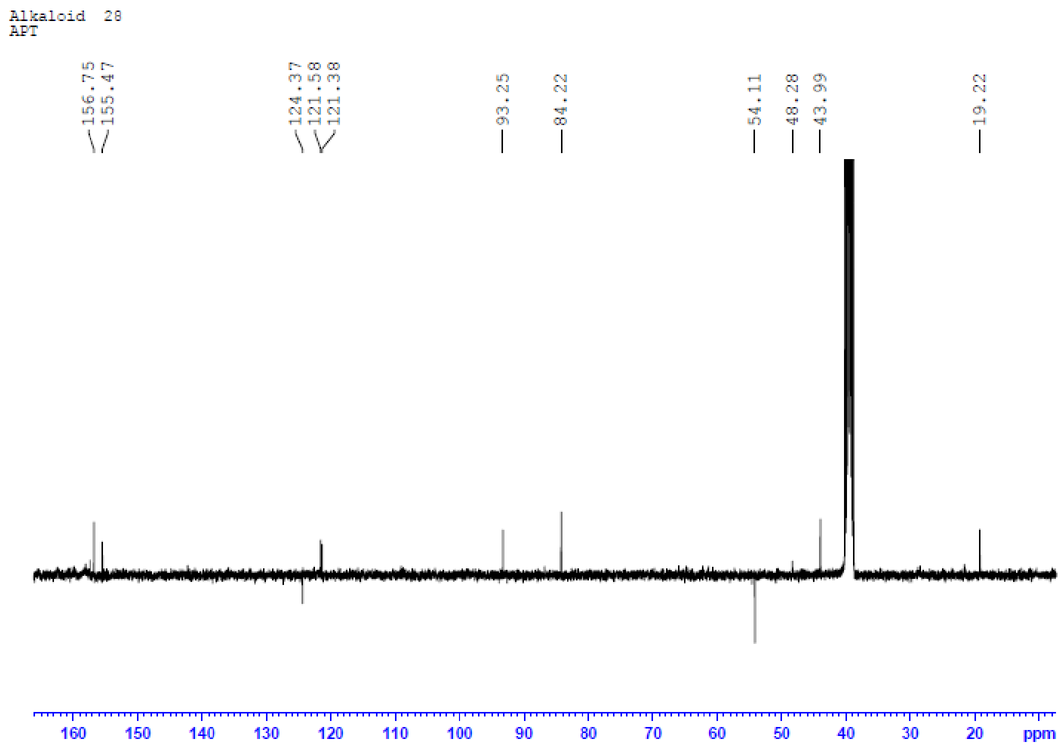

^{13}C-NMR spectrum of **28** (DMSO-d_6, 100MHz)

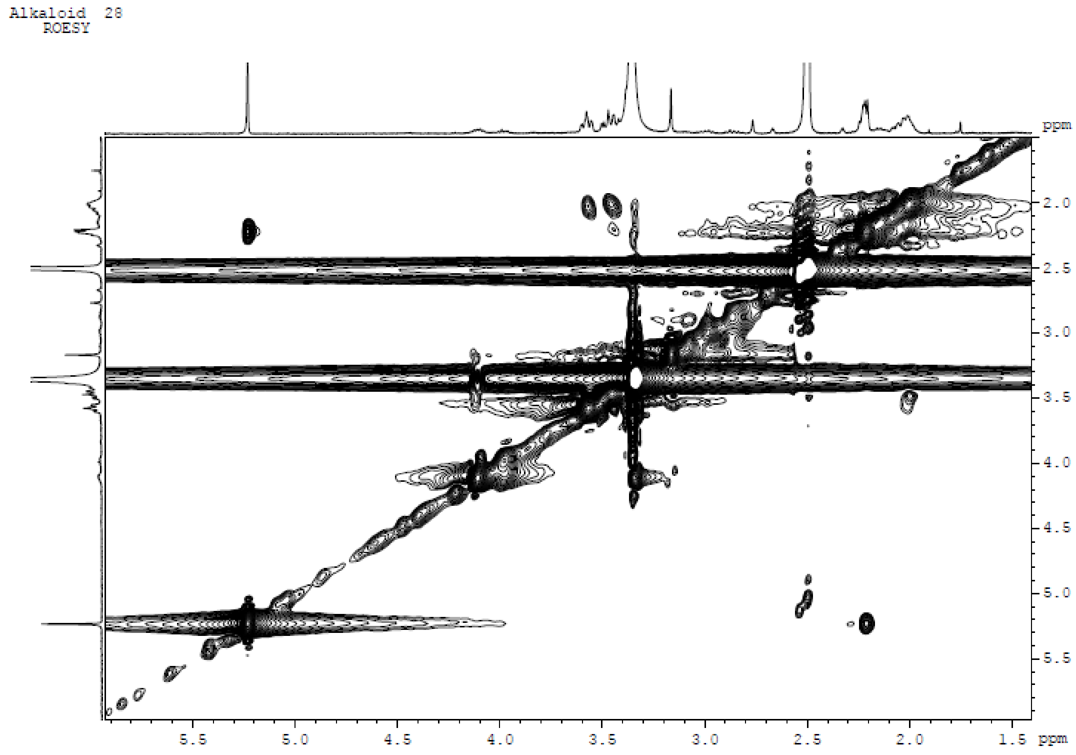

ROESY spectrum of **28** (DMSO-d_6, 400MHz)

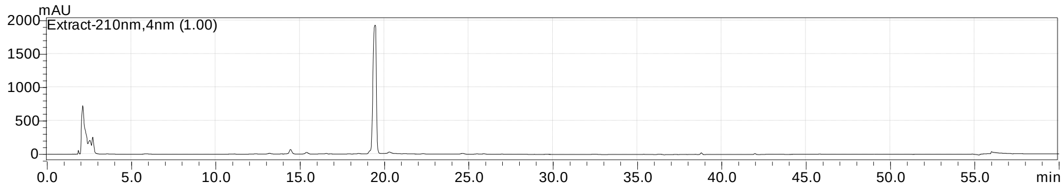

The DAD-HPLC of **29** (0-40min, 5%-100%MeOH-H_2O)

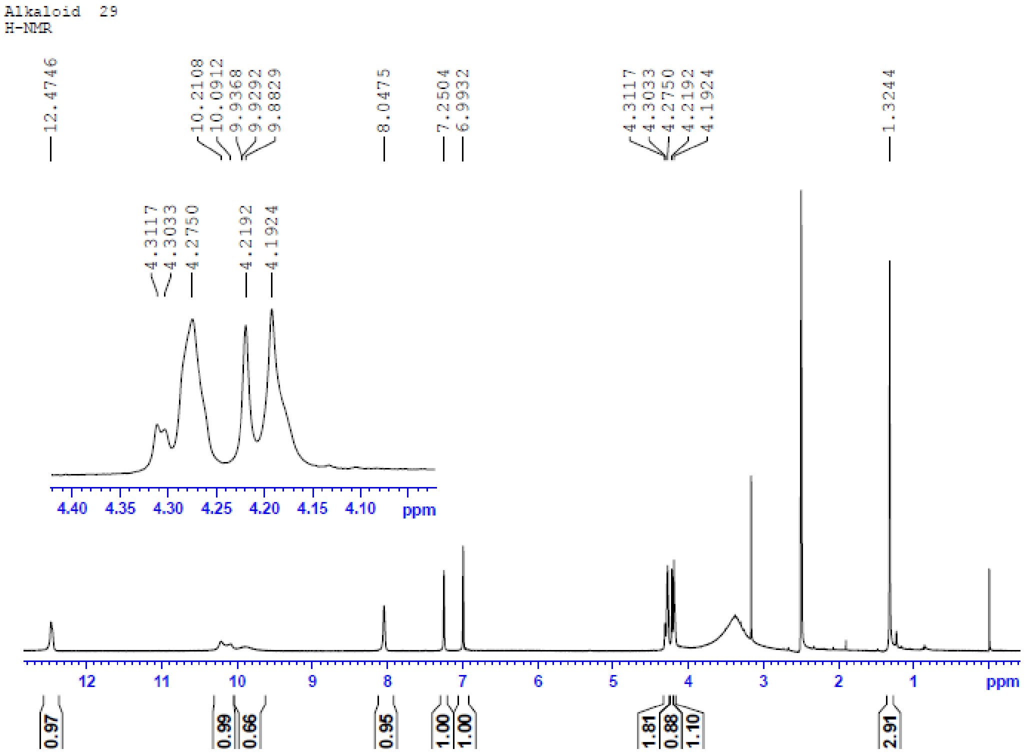

^{1}H-NMR spectrum of **29** (DMSO-d_6, 400MHz)

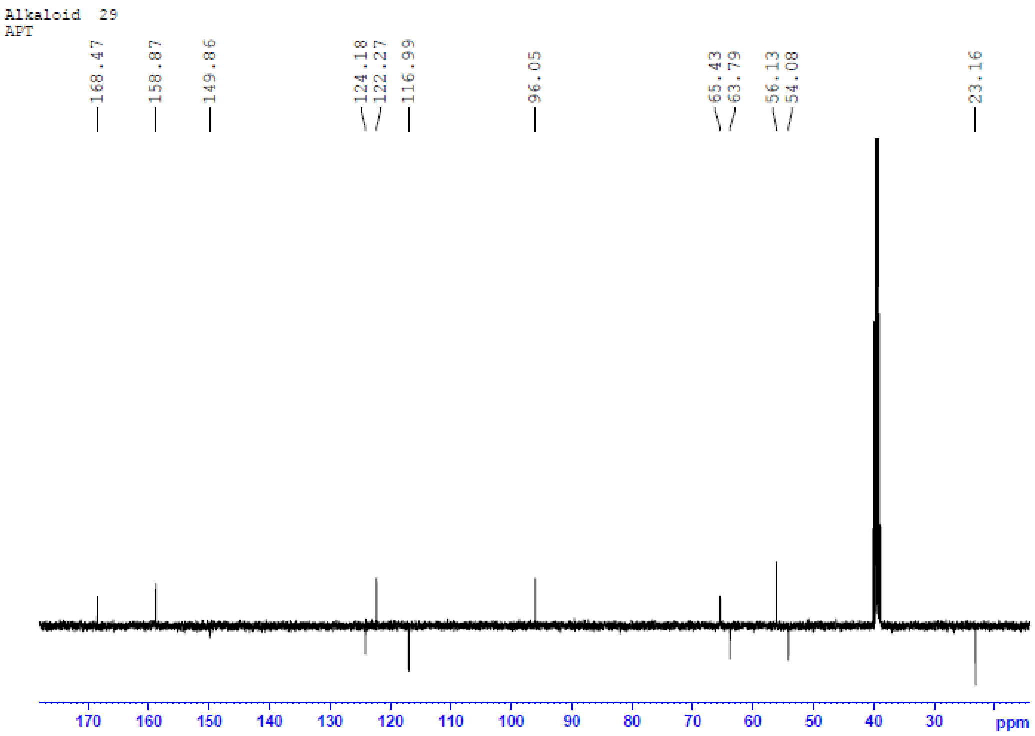

^{13}C-NMR spectrum of **29** (DMSO-*d*$_6$, 100MHz)

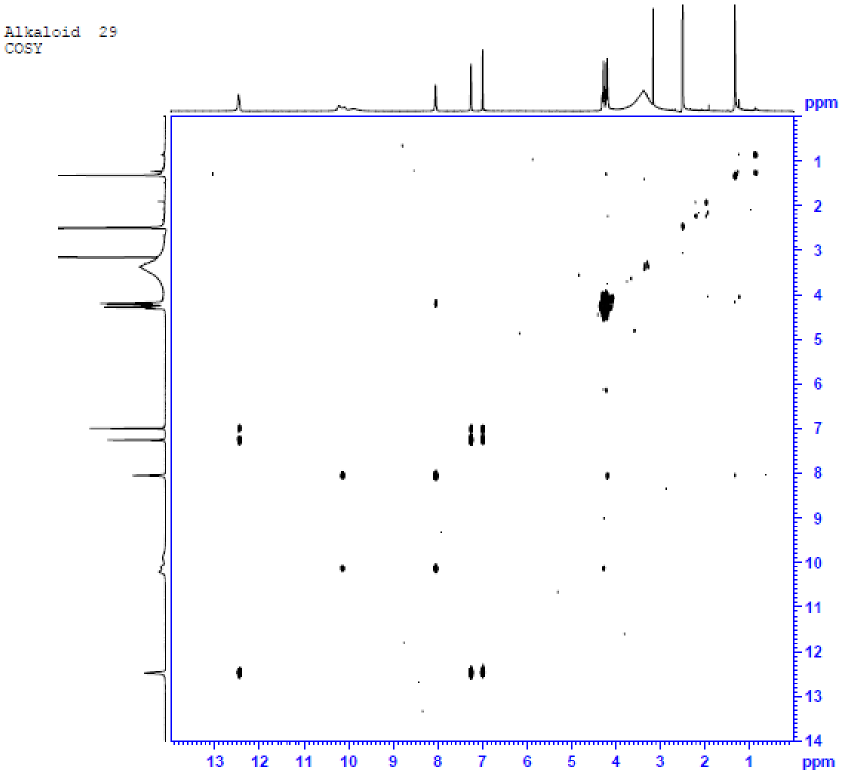

^{1}H-^{1}H COSY spectrum of **29** (DMSO-*d*$_6$, 400MHz)

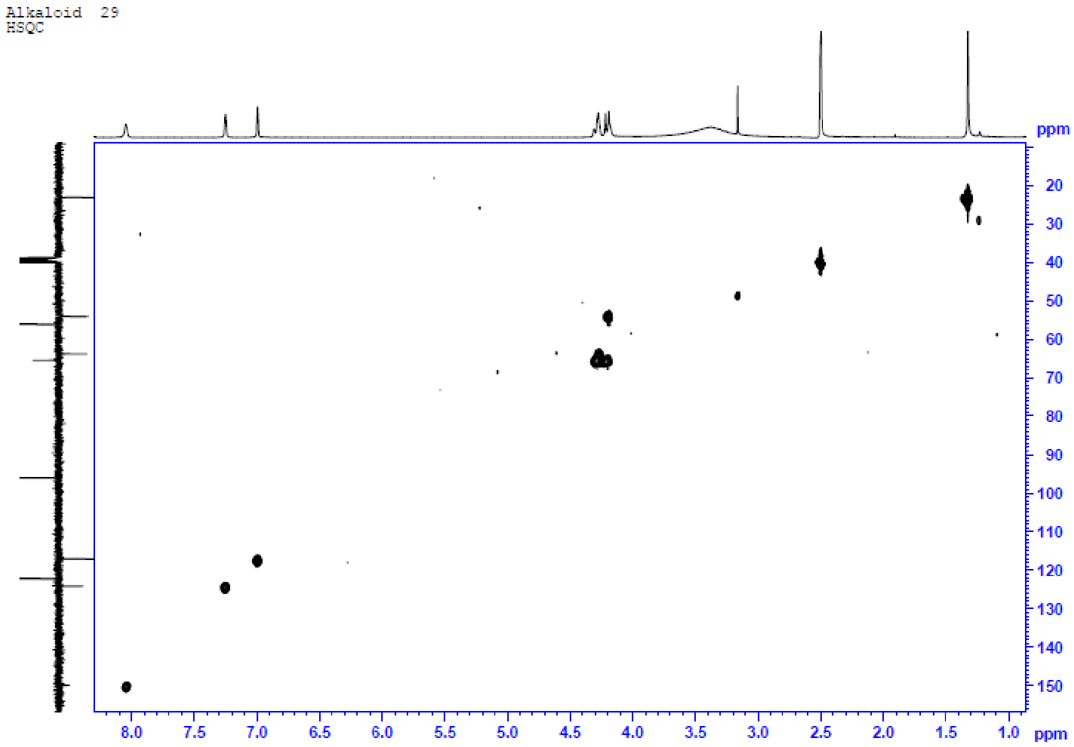

HSQC spectrum of **29** (DMSO-d_6, 400MHz)

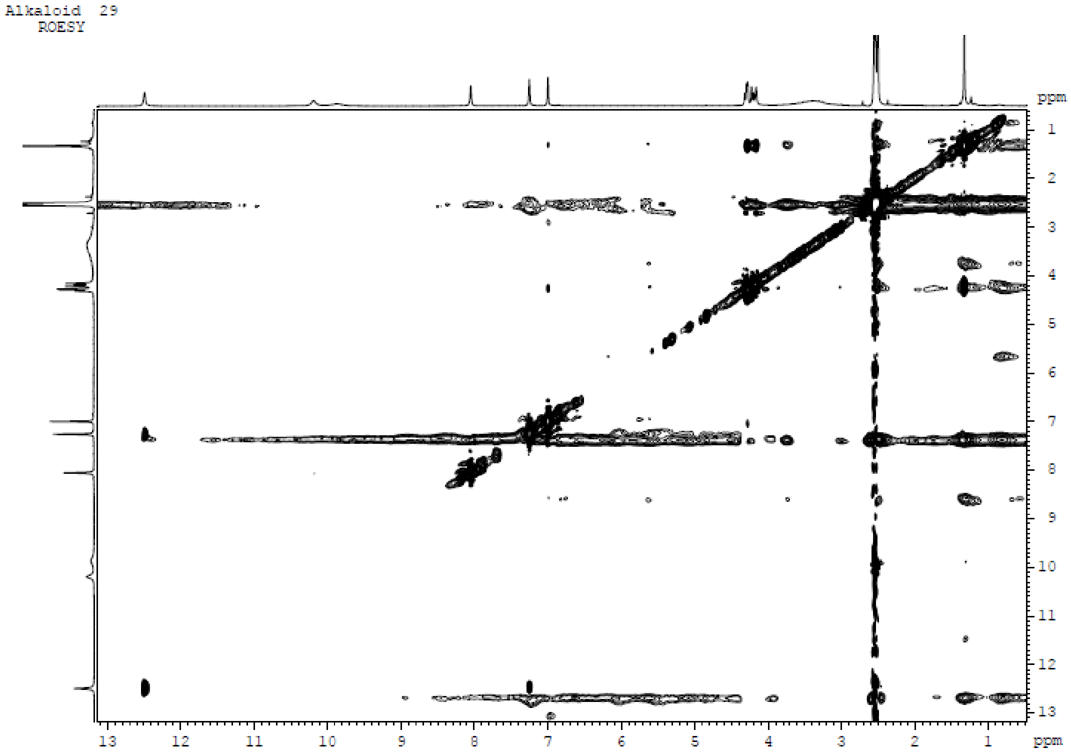

ROESY spectrum of **29** (DMSO-d_6, 400MHz)

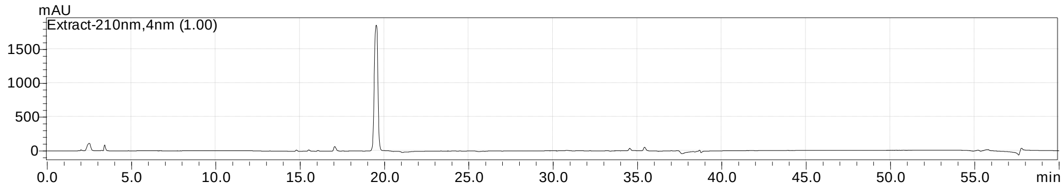

The DAD-HPLC of **30** (0-40min, 5%-100%MeOH-H_2O)

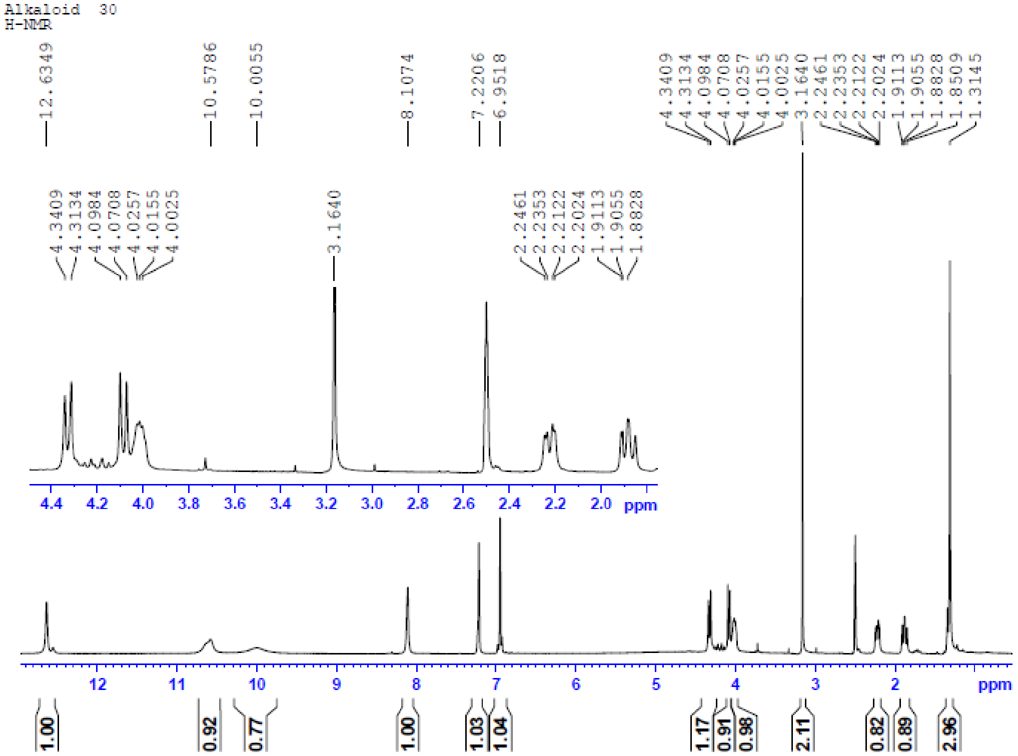

^{1}H-NMR spectrum of **30** (DMSO-d_6, 400MHz)

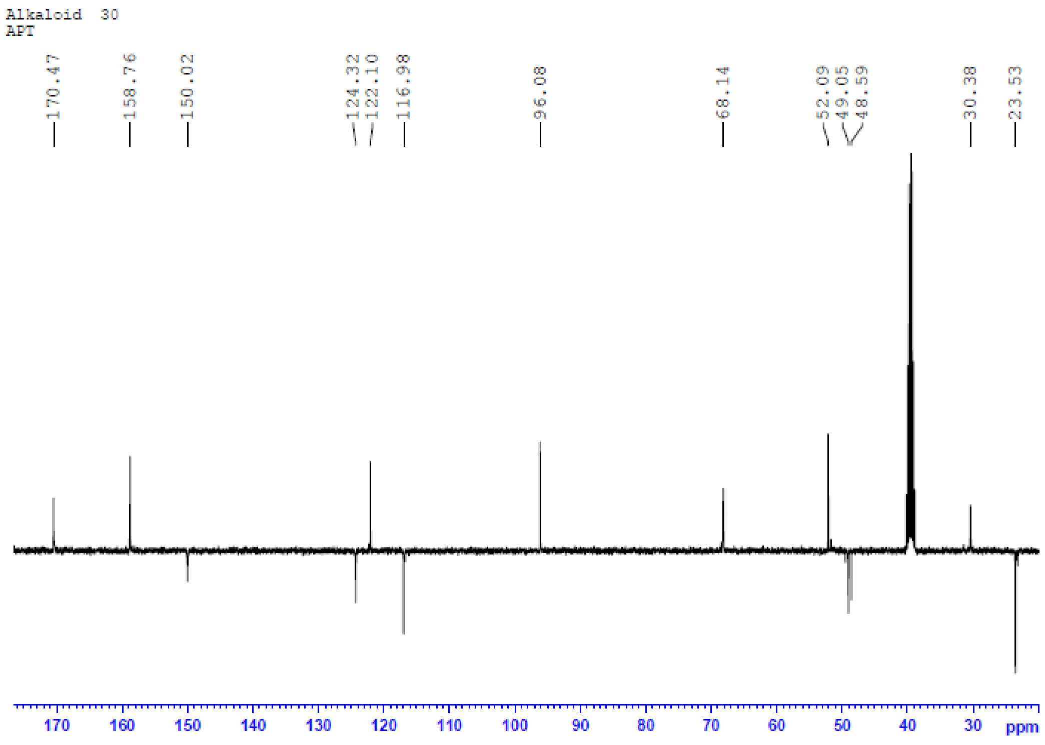

^{13}C-NMR spectrum of **30** (DMSO-d_6, 100MHz)

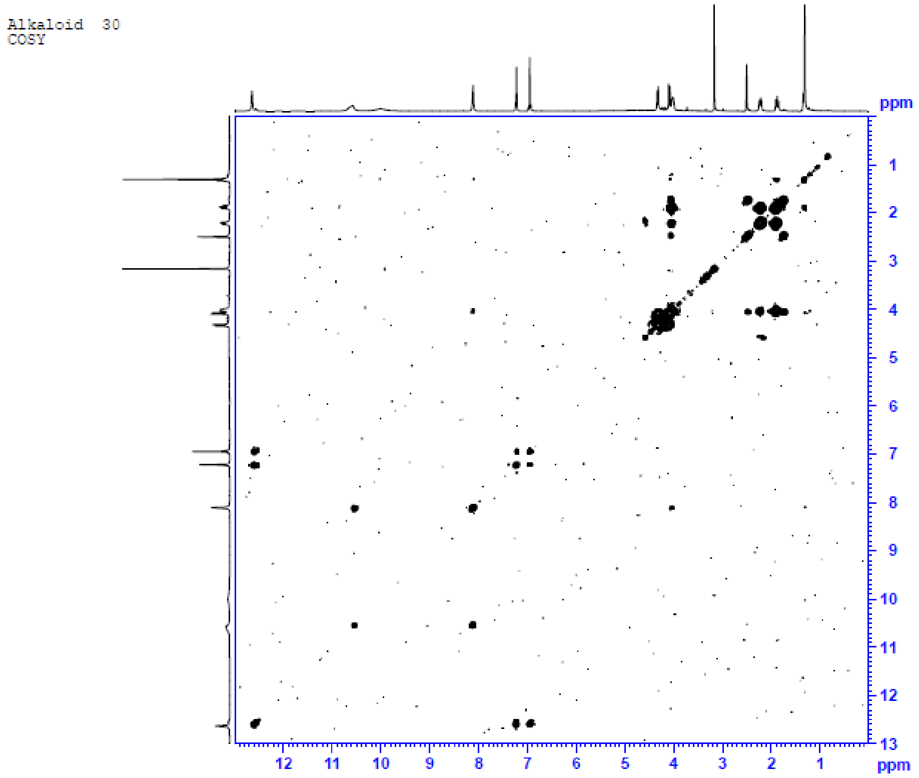

^{1}H-^{1}H COSY spectrum of **30** (DMSO-d_6, 400MHz)

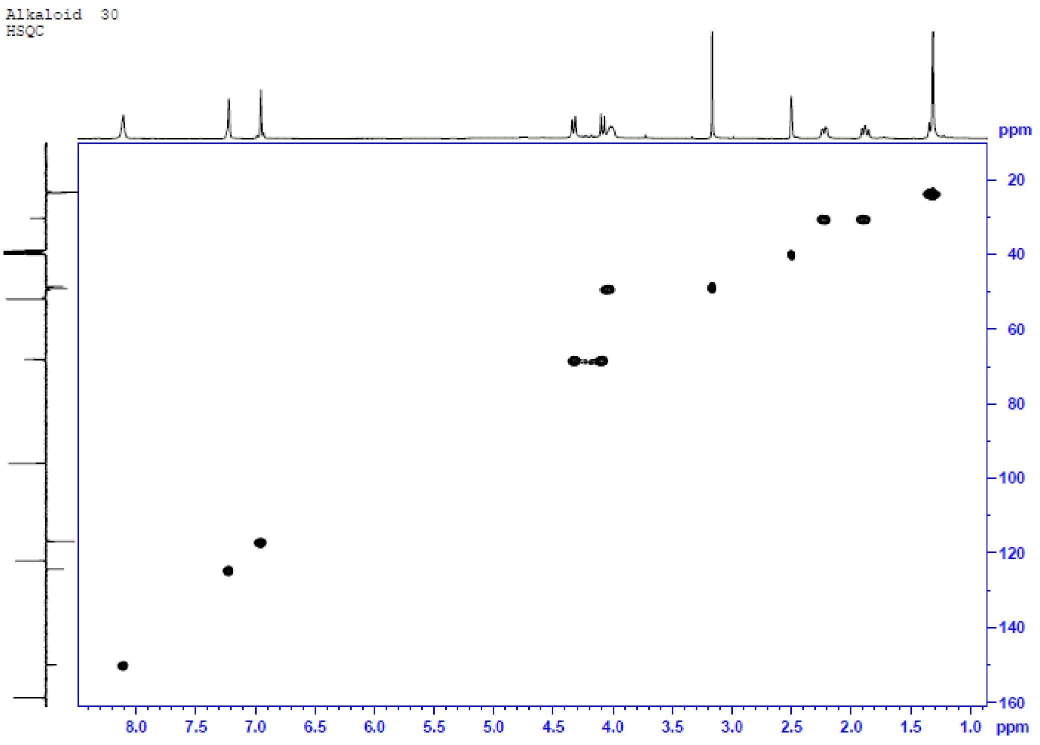

HSQC spectrum of **30** (DMSO-d_6, 400MHz)

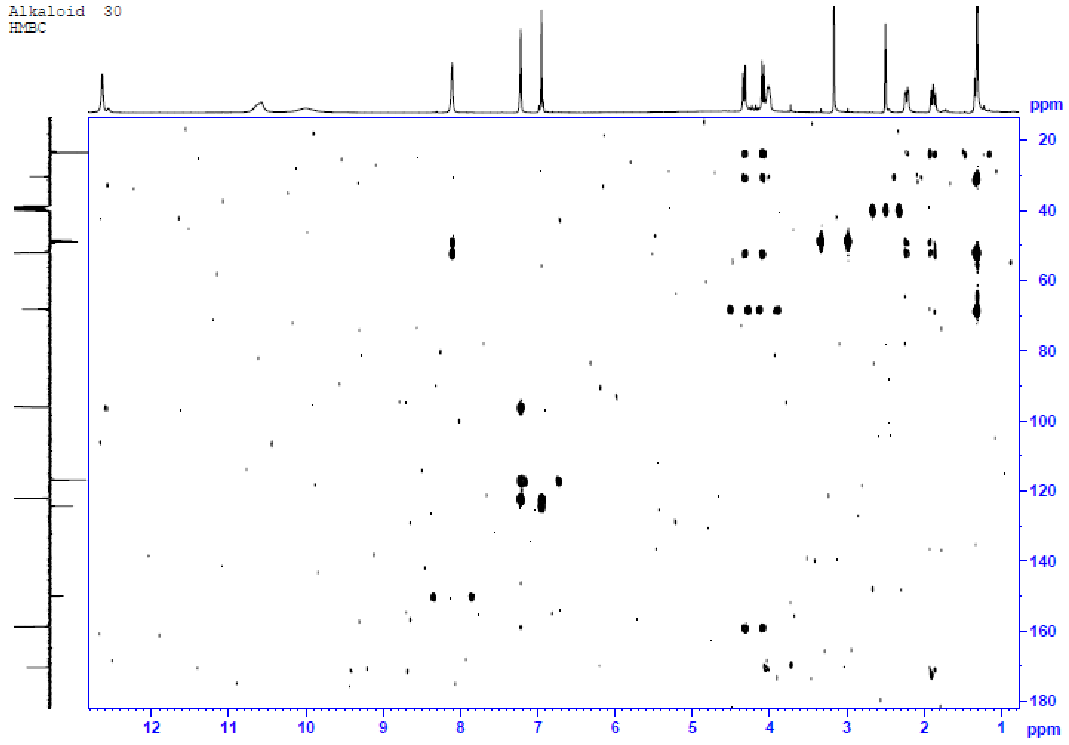

HMBC spectrum of **30** (DMSO-d_6, 400MHz)

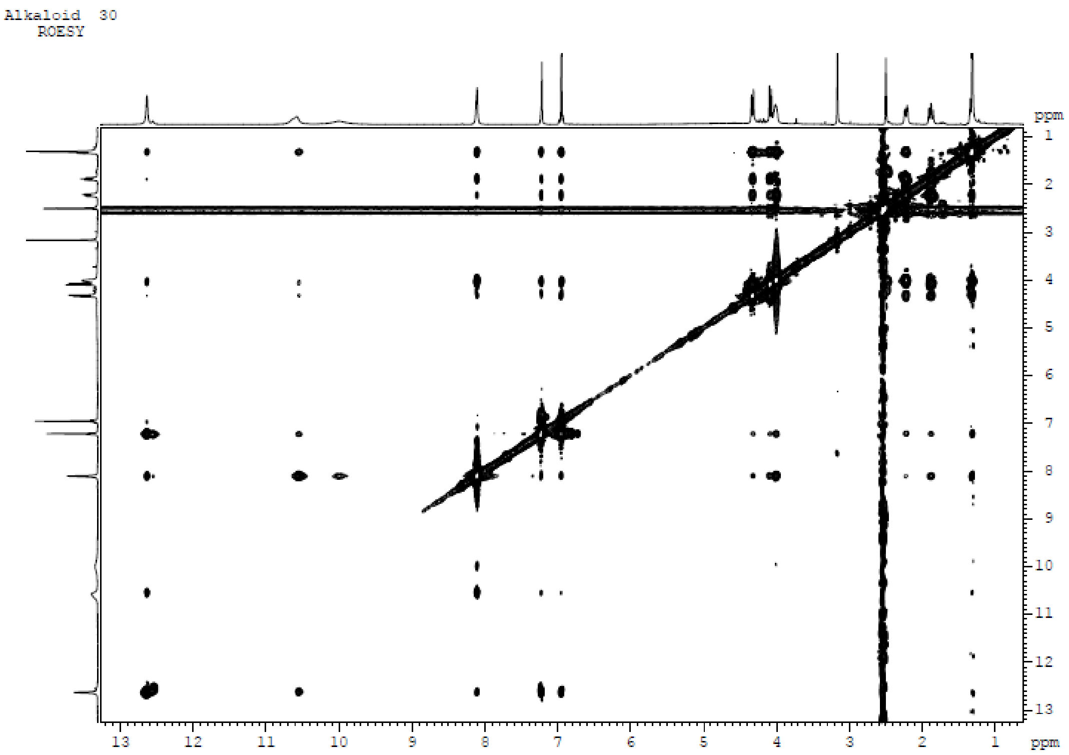

ROESY spectrum of **30** (DMSO-d_6, 400MHz)

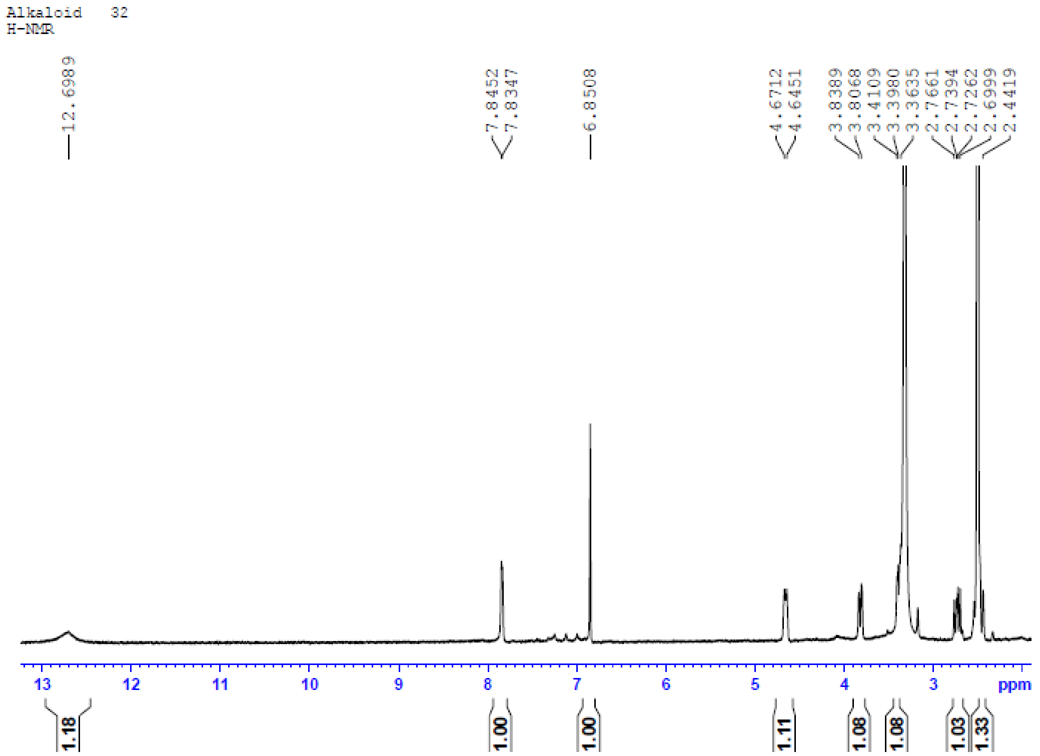

^{1}H-NMR spectrum of **32** (DMSO-d_6, 400MHz)

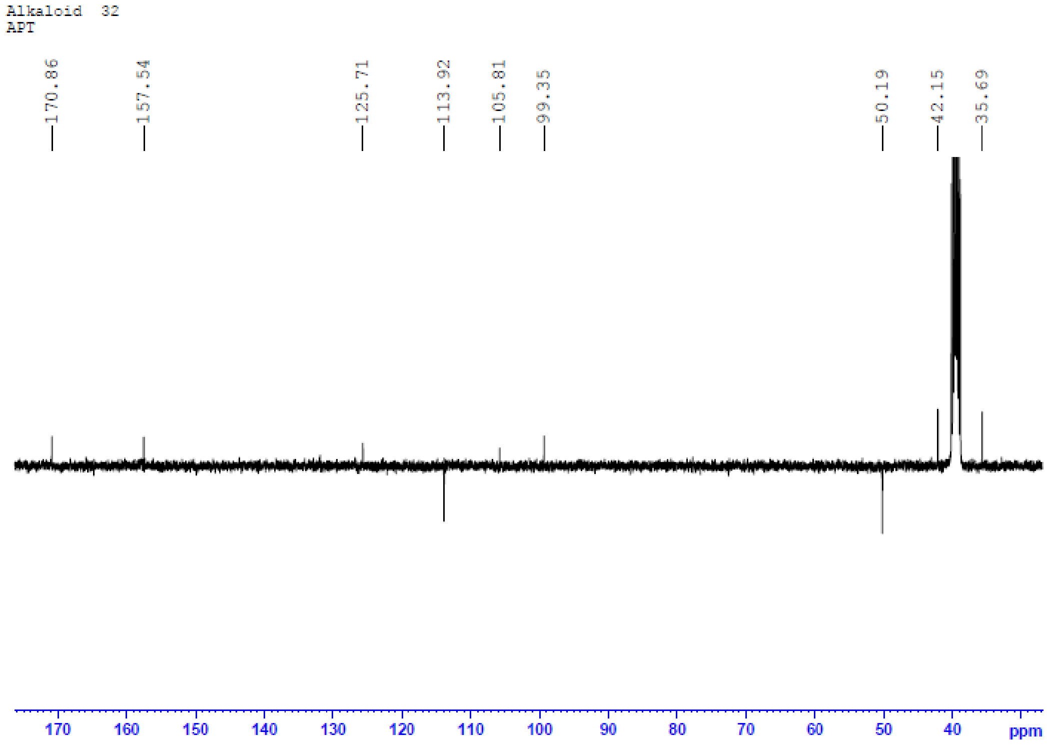

^{13}C-NMR spectrum of **32** (DMSO-d_6, 100MHz)

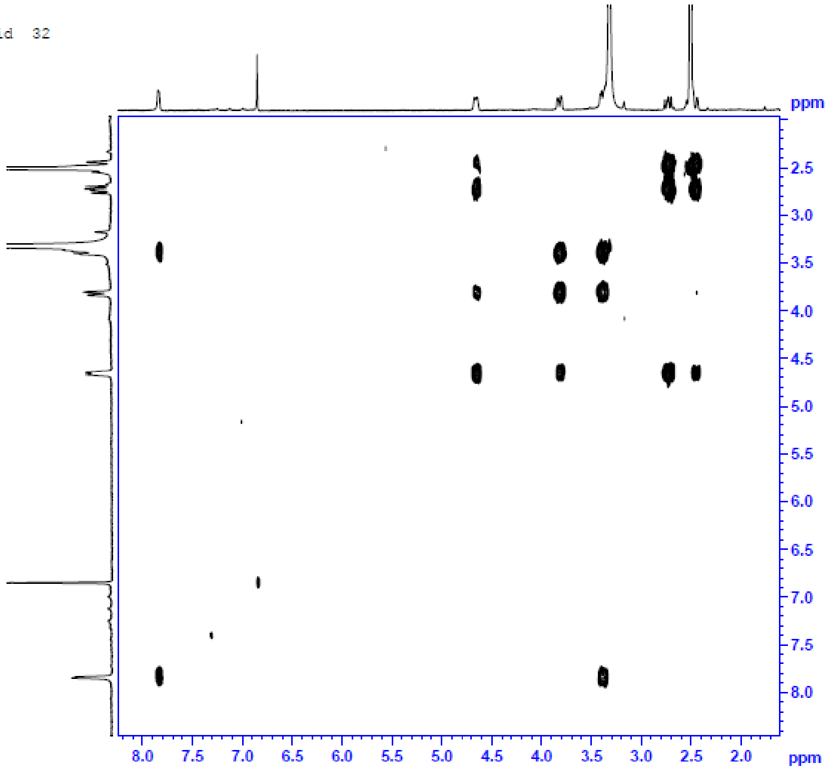

^{1}H-^{1}H COSY spectrum of **32** (DMSO-d_6, 400MHz)

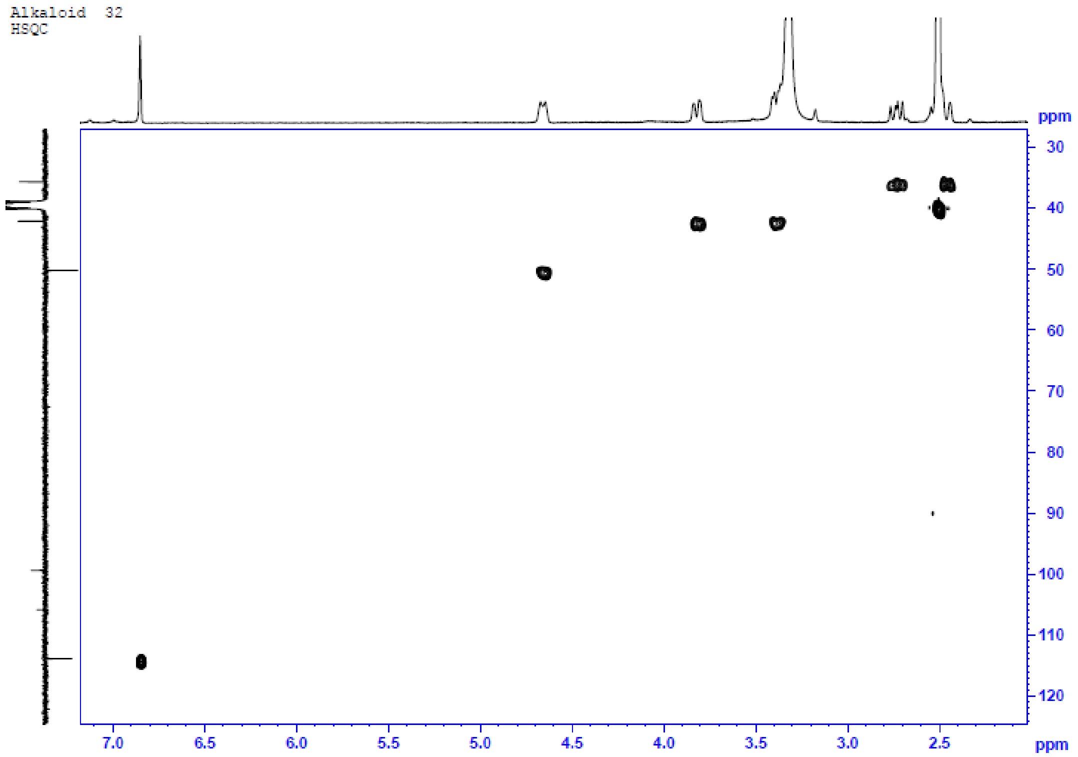

HSQC spectrum of **32** (DMSO-d_6, 400MHz)

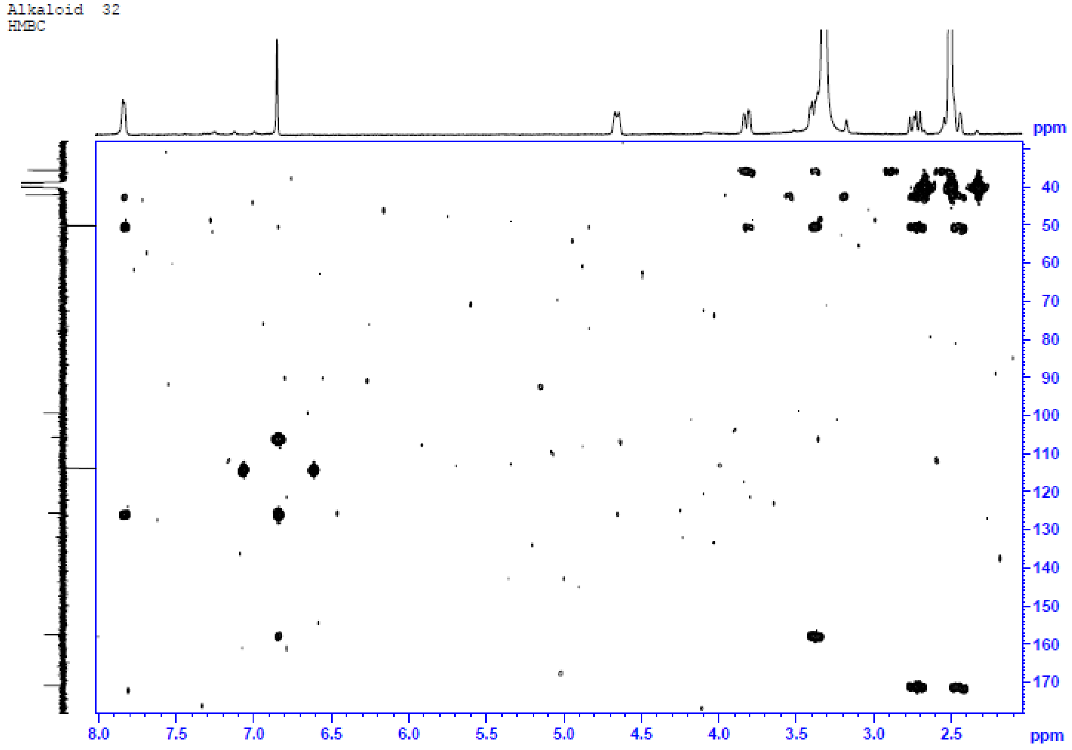

HMBC spectrum of **32** (DMSO-d_6, 400MHz)

Table S1. Antimicrobial effects of alkaloids

No.	MIC (μg/mL)						
	S. aureus ATCC 25923	*S. haemolyticus* ATCC 29970	*B. subtilis* ATCC 11562	*X. vesicatoria* ATCC 11633	*P. lachrymans* ATCC 11633	*A. tumefaciens* ATCC 11158	*R. solanacearum* ATCC 11696
**1**	>128	>128	>128	>128	>128	>128	>128
**2/3**	>128	>128	>128	>128	>128	>128	>128
**4/5**	>128	>128	>128	>128	>128	>128	>128
**6**	>128	>128	>128	>128	>128	>128	>128
**7**	>128	>128	>128	>128	>128	>128	>128
**8**	>128	>128	>128	>128	>128	>128	>128
**9**	>128	>128	>128	>128	128	>128	128
**10**	32	128	64	128	64	128	64
**11**	>128	>128	>128	128	>128	128	>128
**12**	32	>128	>128	>128	128	>128	>128
**13**	>128	>128	>128	>128	>128	>128	>128
**14**	>128	>128	>128	>128	>128	>128	>128
**15**	>128	>128	>128	>128	>128	>128	>128
**16**	>128	>128	>128	>128	128	128	128
**17**	>128	>128	>128	>128	>128	>128	>128
**18**	>128	>128	>128	>128	>128	>128	>128
**19**	>128	>128	>128	>128	>128	>128	>128
**20**	>128	>128	>128	>128	>128	>128	>128
**21**	>128	>128	>128	>128	>128	>128	>128
**22**	>128	>128	>128	>128	>128	>128	>128
**23**	>128	>128	>128	>128	>128	>128	>128
**24**	>128	>128	>128	>128	>128	>128	>128
**25**	>128	>128	>128	>128	>128	>128	>128
**26**	>128	>128	>128	>128	>128	>128	>128
**27**	>128	>128	>128	>128	>128	>128	>128
**28**	>128	>128	>128	>128	>128	>128	>128
**29**	>128	>128	>128	>128	>128	>128	>128
**30**	>128	>128	>128	>128	>128	>128	>128
**31**	>128	>128	>128	>128	>128	>128	>128
**32**	>128	>128	>128	>128	>128	>128	>128

Gram positive strains: *S. aureus*; *S. haemolyticus*; *B. subtilis*; Gram negative strains: *X. vesicatoria*, *P. lachrymans*, *A. tumefaciens*, *R. solanacearum.*
